# Inhibition of lysine acetyltransferase KAT6 in ER^+^HER2^−^ metastatic breast cancer: a phase 1 trial

**DOI:** 10.1038/s41591-024-03060-0

**Published:** 2024-06-01

**Authors:** Toru Mukohara, Yeon Hee Park, David Sommerhalder, Kan Yonemori, Erika Hamilton, Sung-Bae Kim, Jee Hyun Kim, Hiroji Iwata, Toshinari Yamashita, Rachel M. Layman, Monica Mita, Timothy Clay, Yee Soo Chae, Catherine Oakman, Fengting Yan, Gun Min Kim, Seock-Ah Im, Geoffrey J. Lindeman, Hope S. Rugo, Marlon Liyanage, Michelle Saul, Christophe Le Corre, Athanasia Skoura, Li Liu, Meng Li, Patricia M. LoRusso

**Affiliations:** 1 National Cancer Center Hospital East, Kashiwa, Japan; 2 Samsung Medical Center, Sungkyunkwan University School of Medicine, Seoul, Republic of Korea; 3 NEXT Oncology, San Antonio, TX USA; 4 National Cancer Center Hospital, Tokyo, Japan; 5 Sarah Cannon Research Institute, Nashville, TN USA; 6 Asan Medical Center, University of Ulsan College of Medicine, Seoul, Republic of Korea; 7 Seoul National University Bundang Hospital, Seoul National University College of Medicine, Seongnam, Republic of Korea; 8 Nagoya City University, Graduate School of Medical Sciences, Nagoya, Japan; 9 Kanagawa Cancer Center, Yokohama, Japan; 10 The University of Texas MD Anderson Cancer Center, Houston, TX USA; 11 Hoag Family Cancer Institute, Newport Beach, CA USA; 12 Saint John of God Subiaco Hospital, Perth, Western Australia Australia; 13 Kyungpook National University Chilgok Hospital, School of Medicine, Kyungpook National University, Daegu, Republic of Korea; 14 Western Health, Sunshine Hospital, St Albans, Victoria Australia; 15 Swedish Cancer Institute, First Hill-True Family Women’s Cancer Center, Seattle, WA USA; 16 Yonsei University College of Medicine, Seoul, Republic of Korea; 17 Seoul National University Hospital, Seoul National University College of Medicine, Cancer Research Institute, Seoul National University, Seoul, Republic of Korea; 18 Peter MacCallum Cancer Centre and Walter and Eliza Hall Institute of Medical Research, Melbourne, Victoria Australia; 19 University of California, San Francisco, CA USA; 20 Pfizer, San Diego, CA USA; 21 Pfizer, Collegeville, PA USA; 22 Pfizer, San Francisco, CA USA; 23 Yale School of Medicine, New Haven, CT USA

**Keywords:** Drug development, Target validation

## Abstract

Inhibition of histone lysine acetyltransferases (KATs) KAT6A and KAT6B has shown antitumor activity in estrogen receptor-positive (ER^+^) breast cancer preclinical models. PF-07248144 is a selective catalytic inhibitor of KAT6A and KAT6B. In the present study, we report the safety, pharmacokinetics (PK), pharmacodynamics, efficacy and biomarker results from the first-in-human, phase 1 dose escalation and dose expansion study (*n* = 107) of PF-07248144 monotherapy and fulvestrant combination in heavily pretreated ER^+^ human epidermal growth factor receptor-negative (HER2^−^) metastatic breast cancer (mBC). The primary objectives of assessing the safety and tolerability and determining the recommended dose for expansion of PF-07248144, as monotherapy and in combination with fulvestrant, were met. Secondary endpoints included characterization of PK and evaluation of antitumor activity, including objective response rate (ORR) and progression-free survival (PFS). Common treatment-related adverse events (any grade; grades 3–4) included dysgeusia (83.2%, 0%), neutropenia (59.8%, 35.5%) and anemia (48.6%, 13.1%). Exposure was approximately dose proportional. Antitumor activity was observed as monotherapy. For the PF-07248144–fulvestrant combination (*n* = 43), the ORR (95% confidence interval (CI)) was 30.2% (95% CI = 17.2–46.1%) and the median PFS was 10.7 (5.3–not evaluable) months. PF-07248144 demonstrated a tolerable safety profile and durable antitumor activity in heavily pretreated ER^+^HER2^−^ mBC. These findings establish KAT6A and KAT6B as druggable cancer targets, provide clinical proof of concept and reveal a potential avenue to treat mBC. clinicaltrial.gov registration: NCT04606446.

## Abstract (web summary)

In this phase 1 trial, treatment of patients with ER^+^HER2^−^ metastatic breast cancer with a selective catalytic inhibitor of the lysine acetyltransferase KAT6 shows a manageable safety profile and encouraging preliminary clinical efficacy.

## Main

Epigenetic regulators control gene transcription and fundamental cellular processes and are potential cancer drivers^1^. Histone acetylation and deacetylation are dynamic and reversible processes catalyzed by two classes of enzymes: histone acetyltransferase (HAT) and histone deacetylase (HDAC). Several HDAC inhibitors have been approved for the treatment of hematological malignancies, including vorinostat, belinostat, romidepsin and panobinostat^2–4^. However, HDAC inhibitors have shown limited success in the treatment of solid tumors owing to a lack of cancer specificity or satisfactory therapeutic window.

A reversed biological process of histone deacetylation is histone acetylation, which is catalyzed by HATs. *KAT6A*, and its paralog, *KAT6B*, encode KATs that regulate lineage-specific gene transcription via H3K23 acetylation (H3K23Ac)^5–7^. *KAT6A* was first identified as part of a chromosomal translocation t(8:16)(p11;p13) with CREB-binding protein in a subset of acute myeloid leukemia (AML)^8,9^. The functions of KAT6A have been interrogated in breast cancer preclinical models because it is located within the 8p11-p12 amplicon that is found amplified in 12–15% of breast cancers^10–12^. An orally bioavailable KAT6A and 6B inhibitor that selectively inhibits the catalytic activity of KAT6A and KAT6B was recently shown to elicit on-target antitumor activity in vivo in ER^+^ breast cancer and other cancer types in preclinical studies^13^. However, epigenetic drugs have faced challenges as a result of the complexity of epigenetic regulation, intrinsic heterogeneity of human cancer and potential off-target effects. No KAT6 inhibitor had been evaluated in the clinic.

PF-07248144 is a potent and selective catalytic KAT6A and KAT6B inhibitor developed for clinical investigation. The purpose of this ongoing open-label, multicenter, phase 1 clinical study was to evaluate the safety, tolerability and clinical activity of PF-07248144 as monotherapy or as combined therapy in patients with solid tumors, with a focus on locally advanced or metastatic ER^+^HER2^−^ breast cancer, whose disease progressed after CDK4/6 inhibitors and endocrine therapies.

## Results

**Fig. 1:**
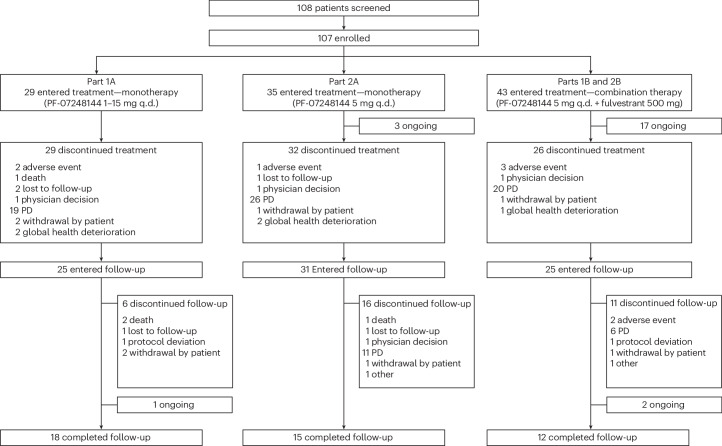
Patient disposition. CONSORT diagram.

**Extended Data Fig. 1:**
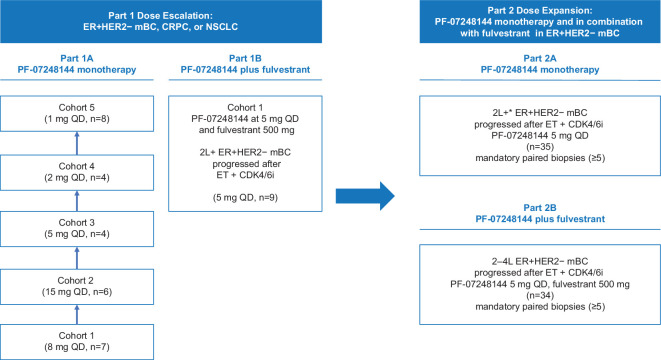
Study design and enrollment status. *3L+ for patients enrolled in the Republic of Korea. 2L, with at least one prior line of treatment; 3L+, as third line and above; CRPC, castration-resistant prostate cancer; ER, estrogen receptor; ET, endocrine therapy; HER2, human epidermal growth factor receptor 2; mBC, locally advanced or metastatic breast cancer; NSCLC, non-small cell lung cancer; QD, once a day.

This report focuses on the 29 patients in part 1A who received PF-07248144 monotherapy once daily (q.d.) at five dose levels (1 mg (*n* = 8), 2 mg (*n* = 4), 5 mg (*n* = 4), 8 mg (*n* = 7) and 15 mg (*n* = 6)), the 35 patients in part 2A who received PF-07248144 at the recommended dose for expansion (RDE) of 5 mg q.d. as monotherapy, and the 43 patients who received PF-07248144 at the RDE of 5 mg q.d. in combination with 500 mg of fulvestrant in parts 1B and 2B. Between 16 November 2020 and 30 September 2023 (study ongoing), 108 patients were screened and 107 patients were enrolled and received at least one dose of PF-07248144 (Fig. 1 and Extended Data Fig. 1).

### PF-07248144 dose escalation (part 1)

In the dose escalation part of the study, 29 patients (mBC, *n* = 12; castration-resistant prostate cancer (CRPC), *n* = 15; non-small cell lung cancer (NSCLC, *n* = 2)) were enrolled in part 1A and received PF-07248144 q.d. monotherapy at five dose levels (1 mg (*n* = 8), 2 mg (*n* = 4), 5 mg (*n* = 4), 8 mg (*n* = 7) and 15 mg (*n* = 6)). The median (range) age was 68.0 years (48.0–90.0 years), 13 (44.8%) patients were female, 11 (37.9%) patients were white, 11 (37.9%) were Asian and 3 (10.3%) patients were Black or African American. Among the patients with mBC (*n* = 12), all received prior CDK4/6 inhibitors and endocrine therapy (ET) in the advanced/metastatic setting. The median (range) prior lines of systemic therapy in the advanced/metastatic setting were 5.0 (1–14) (Table 1).

**Table 1:** Baseline characteristics and prior lines of therapy

	Monotherapy PF-07248144 1-15 mg q.d. dose escalation part 1A (*N* = 29)	Monotherapy PF-07248144 5 mg q.d. part 2A (*N* = 35)	Combination PF-07248144 5 mg q.d. + fulvestrant 500 mg part 1B + part 2B (*N* = 43)
Age, median (range) (years)	68.0 (48.0–90.0)	57.0 (39.0–76.0)	55.0 (24.0–76.0)
Female, *n* (%)	13 (44.8)	35 (100.0)	42 (97.7)
Race, *n* (%)			
White	11 (37.9)	9 (25.7)	13 (30.2)
Black or African American	3 (10.3)	0	1 (2.3)
Asian	11 (37.9)	26 (74.3)	29 (67.4)
Not reported	4 (13.8)	0	0
Primary cancer diagnosis, *n* (%)			
Breast cancer (ER^+^HER2^−^)	12 (41.4)	35 (100)	43 (100)
NSCLC	2 (6.9)	N/A	N/A
CRPC	15 (51.7)	N/A	N/A
ECOG PS, *n* (%)			
0	13 (44.8)	18 (51.4)	17 (39.5)
1	16 (55.2)	17 (48.6)	26 (60.5)
**ER**^+^**HER2**^−^ **mBC prior lines of therapy**			
Number of patients	12	35	43
Systemic therapy in any setting, median (range)	7.0 (1–14)	7.0 (2–13)	2.0 (1–10)
Systemic therapy in advanced/metastatic setting, median (range)	5.0 (1–14)	5.0 (1–13)	1.0 (1–6)
Prior CDK4/6 inhibitors, *n* (%)	12 (100)	35 (100)	43 (100)
Prior CDK4/6 inhibitors >12 months	7 (58.3)	19 (54.3)	33 (76.7)
Prior palbociclib, *n* (%)	8 (66.7)	31 (88.6)	32 (74.4)
Prior abemaciclib, *n* (%)	4 (33.3)	4 (11.4)	9 (20.9)
Prior ribociclib, *n* (%)	1 (8.3)	3 (8.6)	5 (11.6)
Prior ET in advanced/metastatic setting, median (range)	2.0 (1–6)	2.0 (1–7)	1.0 (1–3)
Prior ET, *n* (%)	12 (100)	35 (100)	43 (100)
Prior aromatase inhibitors, *n* (%)	10 (83.3)	30 (85.7)	38 (88.4)
Prior fulvestrant, *n* (%)	11 (91.7)	28 (80.0)	5 (11.6)
Prior chemotherapies in advanced/metastatic setting, median (range)	2.5 (0–6)	1.0 (0–8)	0.0 (0–3)
Prior chemotherapy, *n* (%)	11 (91.7)	26 (74.3)	12 (27.9)
Prior other targeted therapies (mTOR and PIK), *n* (%)	7 (58.3)	14 (40.0)	2 (4.7)
*ESR1* mutant, *n* (%)	5/12 (41.7)	22/35 (62.9)	24/42 (57.1)

mTOR, mechanistic target of rapamycin; PIK, phosphoinositide kinase.

In part 1A (*n* = 29), three dose-limiting toxicities (DLTs) (2 Grade (G) 3 neutropenia and 1 G3 anemia) were observed (PF-07248144 1, 2 and 8 mg q.d.) (Extended Data Table 1a). According to the DLT rates at different dose levels and the Bayesian logistic regression model (Extended Data Table 1b), the maximum tolerated dose (MTD) for PF-07248144 was not reached; 5 mg q.d. was identified as the RDE for PF-07248144 monotherapy based on clinical safety, PK exposure, potent blood PD inhibition (target engagement) and preliminary antitumor activity. Treatment-related adverse events (TRAEs) G ≥ 3 observed in >1 patient were anemia (7 (24.1%)), neutropenia (5 (17.2%)), and leukopenia (2 (6.9%)). No febrile neutropenia adverse events (AEs) were reported. At the highest dose level (15 mg), one patient with NSCLC had G5 pneumonitis considered by the investigator as related to study treatment. No other pneumonitis of any grade was reported (*n* = 107). TRAEs led to dose interruptions and reductions in 41.4% and 34.5% of the patients, respectively. TRAEs leading to dose reduction in more than one patient were anemia (17.2%), neutropenia (13.8%) and dysgeusia (6.9%). Three (10.3%) patients discontinued treatment due to treatment-emergent adverse events (TEAEs) (G3 neutropenia, 8 mg q.d.; G5 pneumonitis, 15 mg q.d.; G3 anemia, 15 mg q.d.) (Table 2 and Extended Data Table 2).

**Table 2:** Most frequent TRAEs by preferred terms in ≥10% of patients of any treatment group

	Monotherapy PF-07248144 1-15 mg q.d. dose escalation part 1A (*N* = 29)		Monotherapy PF-07248144 5 mg q.d. part 2A (*N* = 35)		Combination PF-07248144 5 mg q.d. + fulvestrant 500 mg part 1B + part 2B (*N* = 43)	
By preferred term, *n* (%) of patients	All grade	Grade ≥3	All grade	Grade ≥3	All grade	Grade ≥3
With any adverse event	27 (93.1)	13 (44.8)	33 (94.3)	19 (54.3)	43 (100.0)	25 (58.1)
Dysgeusia^a^	23 (79.3)	0	30 (85.7)^a^	0	36 (83.7)^a^	0
Anemia	18 (62.1)	7 (24.1)	16 (45.7)	3 (8.6)	18 (41.9)	4 (9.3)
Neutropenia^b^	12 (41.4)	5 (17.2) Grade 4: 0 (0.0)	24 (68.6)	14 (40.0) Grade 4: 2 (5.7)	28 (65.1)	19 (44.2) Grade 4: 1 (2.3)
Thrombocytopenia	10 (34.5)	1 (3.4)	13 (37.1)	0	5 (11.6)	1 (2.3)
Aspartate aminotransferase increased	9 (31.0)	0	8 (22.9)	0	3 (7.0)	0
Leukopenia	9 (31.0)	2 (6.9)	16 (45.7)	4 (11.4)	14 (32.6)	5 (11.6)
Diarrhea	8 (27.6)	1 (3.4)	5 (14.3)	0	7 (16.3)	0
Alanine aminotransferase increased	5 (17.2)	0	6 (17.1)	2 (5.7)	6 (14.0)	0
Decreased appetite	5 (17.2)	0	2 (5.7)	0	6 (14.0)	1 (2.3)
Fatigue	4 (13.8)	0	6 (17.1)	0	18 (41.9)	1 (2.3)
Electrocardiogram QT prolonged	3 (10.3)	0	4 (11.4)	1 (2.9)	3 (7.0)	0
Hypomagnesemia	3 (10.3)	0	0	0	2 (4.7)	0
Lymphopenia	3 (10.3)	1 (3.4)	1 (2.9)	0	1 (2.3)	0
Nausea	3 (10.3)	0	3 (8.6)	0	6 (14.0)	0
Vomiting	3 (10.3)	0	0	0	1 (2.3)	0
Stomatitis	1 (3.4)	0	5 (14.3)	0	7 (16.3)	1 (2.3)

Preferred terms included in hematologic TEAEs are provided in the footnote of Extended Data Table 2.

^a^Dysgeusia (n = 78; at PF-07248144 5 mg QD monotherapy or combined with fulvestrant): 51/78 (65.4%) were Grade 1 and 15/78 (19.2%) were Grade 2.

^b^For neutropenia: no febrile neutropenia reported (n = 107)

The RDE of 5 mg q.d. was explored in combination with fulvestrant in the part 1B dose escalation (*n* = 9). The safety profile of patients in part 1B (*n* = 9) was consistent with those in part 1A; two DLTs (2 G3 neutropenia) were observed in part 1B (Extended Data Table 1). Collective safety, efficacy, PK and pharmacodynamic data supported 5 mg q.d. as the RDE for PF-07248144 + fulvestrant.

**Extended Data Fig. 2:**
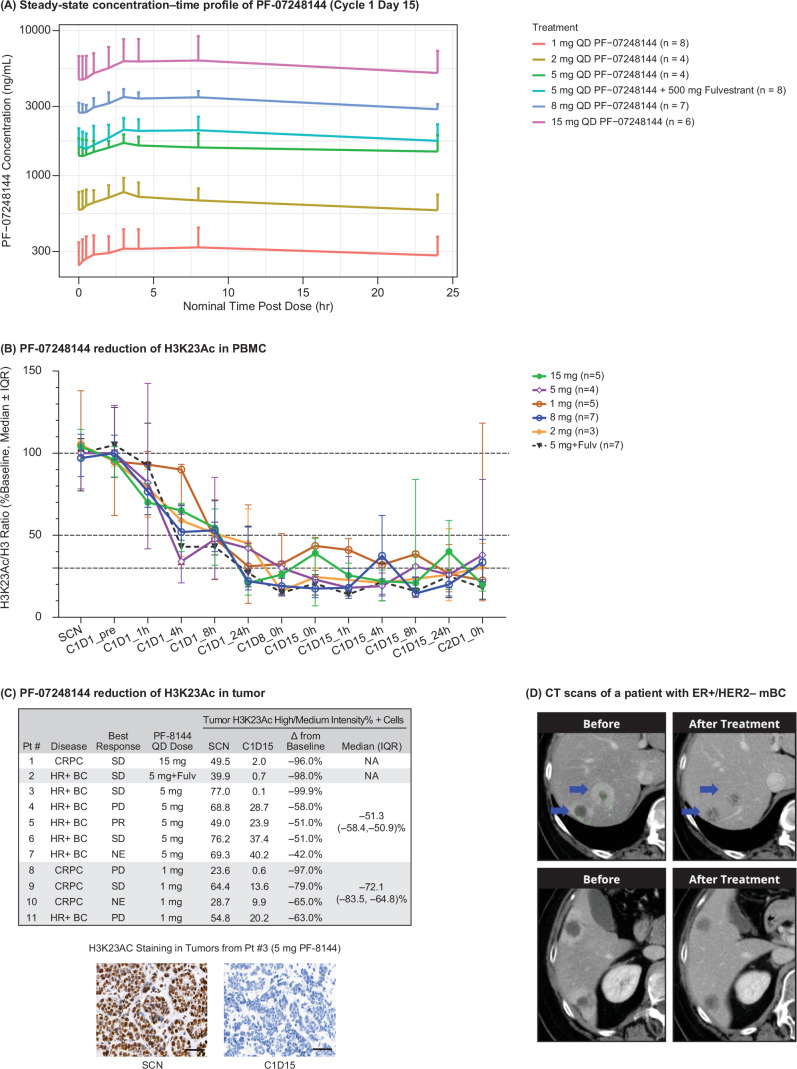
Pharmacokinetics and pharmacodynamics of PF-0728144 and tumor size change from baseline over time. (**a**) Steady-state (C1D15) concentration-time profiles are provided as mean + standard deviation for each cohort. (**b**) H3K23Ac PD biomarker modulation in PBMC by single and multiple doses of PF-07248144 in part 1A (monotherapy) and part 1B (in combination with fulvestrant). Data are presented as median ± interquartile range (IQR). (**c**) H3K23Ac PD biomarker modulation by PF-07248144 after 14 days of treatment in tumor. High, medium, and low intensity H3K23Ac staining positive tumor cells from the entire tissue were evaluated from semi-quantitative image analysis. Percentage of high and medium intensity positive tumor cells were used for tumor H3K23Ac PD changes. Insert representative tumor area images of the IHC staining at 20X for the baseline (SCN) and on-treatment (C1D15) samples from patient #3: brown stain indicates the presence of H3K23AC antigen, while the blue counterstain highlights cell nuclei. The scale bar is 50 µm. (**d**) The patient had ER + /HER2– mBC and *PIK3CA H1047R* mutation and achieved confirmed PR (−39.72%) after PF-07248144 8 mg QD monotherapy with the duration of response of 15.7 months. (Images courtesy of Dr Toru Mukohara). BC, breast cancer; C, cycle; CRPC, castration-resistant prostate cancer; D, day; ER, estrogen receptor; Fulv, fulvestrant; HER2, human epidermal growth factor receptor 2; IQR, interquartile range; mBC, advanced/metastatic breast cancer; NA, not applicable; NE, not evaluable; PD, progressive disease; PF-8144, PF-07248144; PMBC, peripheral blood mononuclear cell; PR; partial response; pre, predose; Pt, patient; QD, once a day; SCN, screening; SD, stable disease.

PF-07248144 PK was linear between 1- and 15-mg q.d. regimens and a steady state was achieved by C1D15 (Extended Data Fig. 2a). After q.d. oral administration (at 1, 2, 5, 8 and 15 mg as monotherapy) or 5 mg in combination with fulvestrant, PF-07248144 was consistently absorbed, with a median steady-state time to peak drug concentration (*T*_max_) of 3 h. Across the dose range (1–15 mg), PF-07248144 steady-state area under the curve (AUC) during the 24-h dosing interval (AUC_24_ on C1D15) and highest drug concentration (*C*_max_) increased in an approximately dose-proportional manner, the steady-state concentrations were near or above effective concentration (*C*_eff_) targets (defined from preclinical models) and the interpatient variability of PF-07248144 was low to moderate, with the geometric coefficient of variation ranging from 12% to 45% for C1D15 AUC_24_ and from 13% to 43% for C1D15 *C*_max_. PF-07248144 accumulated after repeated daily oral dosing with a median effective half-life (*t*_1/2,eff_) across the evaluated doses ranging from 47.1 h to 60.6 h and the median drug accumulation ratio (*R*_ac_) ranging from 3.36 to 4.16 (Extended Data Table 3).

Pharmacodynamic assessments showed that H3K23Ac median reduction from baseline ≥70% in peripheral blood mononuclear cells (PBMCs) was achieved at a steady state on C1D15 at all evaluated doses of PF-07248144 (Extended Data Fig. 2b). Baseline and on-treatment paired biopsies were available from patients receiving PF-07248144 q.d. at doses of 1 mg (*n* = 4) and 15 mg (*n* = 1) monotherapy for 14 d. Tumor H3K23Ac levels were reduced from baseline in patients treated with 1 mg (median change from baseline −72.1%, interquartile range (IQR) −83.5% to −64.8%; *n* = 4) and 15 mg q.d. (change from baseline −97%, *n* = 1) of PF-07248144 (Extended Data Fig. 2c). The H3K23Ac decrease data in PBMCs and tumors from patients dosed at ≥1 mg of PF-07248144 demonstrated a strong KAT6 target inhibition effect.

**Extended Data Fig. 3:**
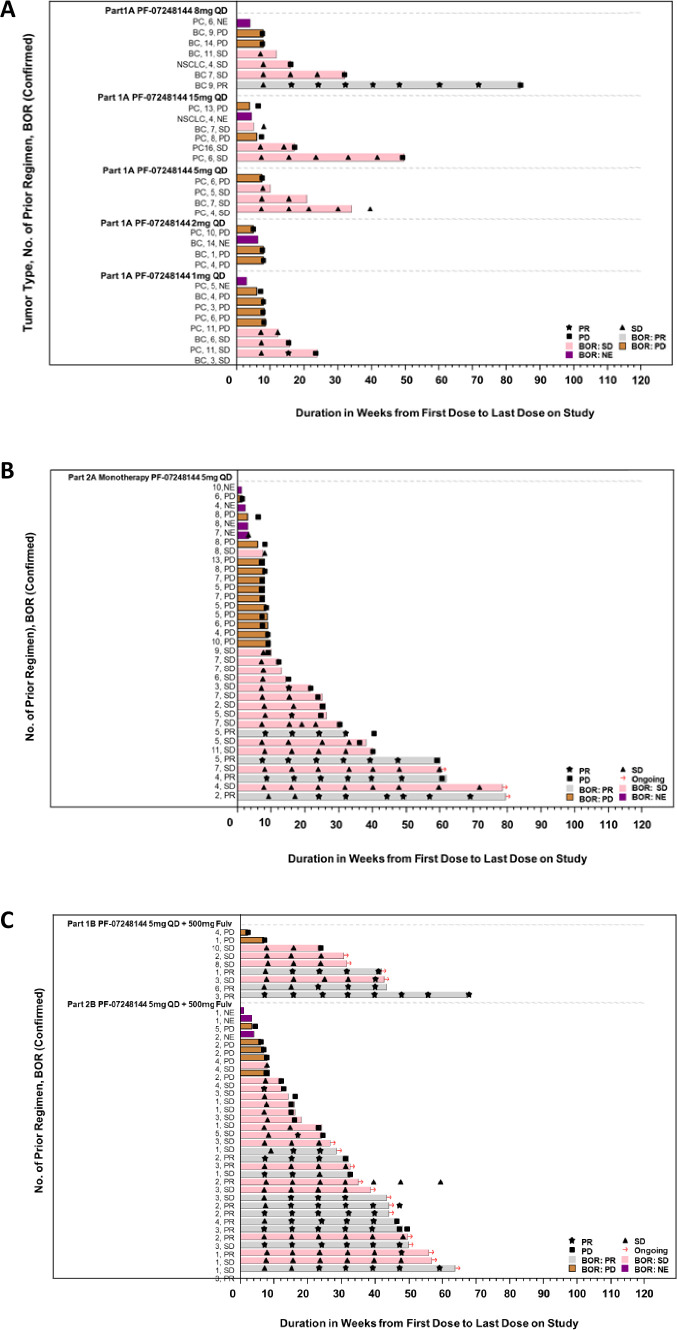
Treatment duration. (**a**) Part 1A dose escalation. (**b**) Part 2A PF-07248144 5 mg QD monotherapy. (**c**) Parts 1B and 2B PF-07248144 5 mg QD plus fulvestrant 500 mg. * All patients in part 2A who received PF-07248144 5 mg QD monotherapy and all patients in part 1B and 2B who received PF-07248144 5 mg QD plus fulvestrant 500 mg had ER + HER2− breast cancer. Patients with BC indicate patients with ER + HER2 − BC. BC, breast cancer; BOR, best of response; ER, estrogen receptor; Fulv, fulvestrant; HER2, human epidermal growth factor receptor 2; NE, not evaluable; NCSLC, non-small cell lung cancer; PC, prostate cancer; PD, progressive disease; PR, partial response; QD, once a day; SD, stable disease.

**Extended Data Fig. 4:**
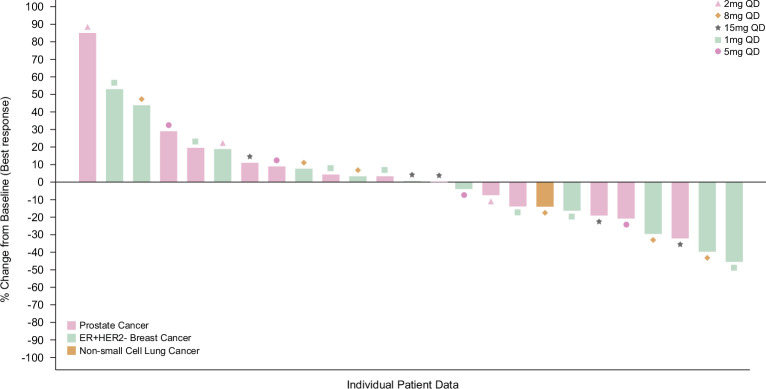
Waterfall plot of tumor size change from baseline of part 1A dose escalation. All patients in part 1A received PF-07248144 monotherapy. ER, estrogen receptor; HER2, human epidermal growth factor receptor 2; PD, progressive disease; PR, partial response; QD, once a day; SD, stable disease.

Preliminary clinical efficacy evaluation showed that confirmed and durable partial response (PR) was observed in one patient with ER^+^HER2^−^ mBC in part 1A, with a 15.7-month duration (8 mg q.d.). Stable disease (SD) was observed in 12 (of 29) patients in part 1A, of whom 6 had ER^+^HER2^−^ mBC, 5 had CRPC and 1 had NSCLC (Extended Data Figs. 2d, 3a and 4).

### PF-07248144 monotherapy (part 2A, 5 mg q.d.) and in combination with fulvestrant (parts 1B and 2B, 5 mg q.d.)

Thirty-five patients with ER^+^HER2^−^ mBC received PF-07248144 as monotherapy at 5 mg q.d., the monotherapy RDE. The median (range) age was 57.0 (39.0–76.0) years, 35 (100%) were female, 9 (25.7%) were white and 26 (74.3%) were Asian (Table 1).

Forty-three patients with ER^+^HER2^−^ mBC received PF-07248144 RDE, 5 mg q.d., in combination with fulvestrant 500 mg (ref. ^14^). The median age was 55.0 (24.0–76.0) years, 42 (97.7%) patients were female, 13 (30.2%) patients were white, 1 (2.3%) patient was Black or African American and 29 (67.4%) were Asian (Table 1).

In the monotherapy group, the median (range) prior lines of systemic therapy in the advanced/metastatic setting were 5 (1–13); 35 (100%) patients received prior CDK4/6 inhibitors and 19 (54.3%) had a duration of CDK4/6 inhibitor treatment of >12 months; 31 patients (88.6%) received prior palbociclib, 4 (11.4%) received prior abemaciclib and 3 (8.6%) received prior ribociclib. The median (range) prior lines of ET in the advanced/metastatic setting were 2 (1–7). Thirty patients (85.7%) received prior aromatase inhibitors, 28 (80.0%) received prior fulvestrant, 26 (74.3%) received prior chemotherapy and 14 (40%) received prior targeted therapies. Of the 35 patients, 22 (62.9%) had one or more detectable *ESR1* mutations in circulating tumor (ct)DNA at baseline (Table 1).

In the combination group, the median (range) prior lines of systemic therapy in the advanced/metastatic setting were 1 (1–6). All patients (*n* = 43, 100%) had received prior CDK4/6 inhibitors, 33 (76.7%) with a duration of CDK4/6 inhibitor treatment >12 months, 32 (74.4%) of whom received prior palbociclib, 9 (20.9%) prior abemaciclib and 5 (11.6%) prior ribociclib. All 43 (100%) patients received prior lines of ET in the advanced/metastatic setting and the median (range) was 1 (1–3); 38 (88.4%) received prior aromatase inhibitors and 5 (11.6%) prior fulvestrant. Twelve (27.9%) patients received prior chemotherapy and two (4.7%) prior targeted therapies. Of the 43 patients, 42 had *ESR1* assayed at baseline; 24 of 42 (57.1%) of these patients had one or more detectable *ESR1* mutations in ctDNA at baseline (Table 1).

#### Safety

In the monotherapy group (*n* = 35), 34 (97.1%) patients reported at least one all-causality TEAE of any grade. The most frequent all-causality TEAEs of any grade were dysgeusia (30 (85.7%)), neutropenia (24 (68.6%)), anemia (18 (51.4%)) and leukopenia (16 (45.7%)). Twenty-two patients (62.9%) reported ≥G3 TEAEs. The most frequent (≥10%) TEAEs ≥G3 were neutropenia (14 (40.0%)), leukopenia and an increase in alanine aminotransferase (ALT) (4 (11.4%) each) (Extended Data Table 2).

Any grade TRAEs were reported in 33 (94.3%) patients. The most frequent TRAEs of any grade were dysgeusia (30/35 (85.7%); G1 26 (74.3%), G2 4 (11.4%)), neutropenia (24 (68.6%)), anemia and leukopenia (16 (45.7%) each). TRAEs ≥G3 were reported in 19 (54.3%) patients. TRAEs ≥G3 in one or more patients included neutropenia (G3, 12 (34.3%); G4, 2 (5.7%)), leukopenia (G3, 4 (11.4%)) and anemia (G3, 3 (8.6%)) and increase in ALT (G3, 2 (5.7%)) (Table 2). No G5 TRAEs were reported. Neutropenia was reversible and well managed with dose modifications; no febrile neutropenia was reported. TRAEs led to dose interruptions and reductions in 57.1% and 45.7% of patients, respectively. The TRAE leading to dose reduction in more than one patient was neutropenia (40.0%). One (2.9%) patient discontinued owing to TEAEs (G2 neutropenia). No events of dysgeusia led to dose reductions or treatment discontinuation.

In the combination group (*n* = 43), all patients (100%) reported at least one all-causality TEAE of any grade. The most frequent all-causality TEAEs of any grade were dysgeusia (37 (86.0%)), neutropenia (28 (65.1%)), fatigue (24 (55.8%)) and anemia (19 (44.2%)). Twenty-seven (62.8%) reported TEAEs ≥G3 and the most frequent (≥10%) TEAEs ≥G3 were neutropenia (19 (44.2%)), leukopenia and anemia (5 (11.6%) each) (Extended Data Table 2).

Any grade TRAEs were reported in 43 (100.0%) patients. The most frequent TRAEs of any grade were dysgeusia (36 (83.7%); G1: 25 (58.1%), G2: 11 (25.6%)), neutropenia (28 (65.1%)), anemia and fatigue (18 (41.9%) each). TRAEs ≥G3 were reported in 25 (58.1%) patients. TRAEs ≥G3 in more than one patient included neutropenia (G3: 18 (41.9%); G4: 1 (2.3%)), leukopenia (G3: 5 (11.6%)) and anemia (G3: 4 (9.3%)) (Table 2). No G5 TRAEs were reported. Neutropenia events were reversible and well managed with dose modification; no febrile neutropenia AEs were reported. TRAEs led to dose interruptions and reductions in 44.2% and 53.5%, respectively. TEAEs leading to dose reduction in more than one patient were neutropenia (39.5%) and anemia (4.7%). Three (7.0%) patients discontinued owing to TEAEs (G2 anemia, G3 myocardial injury/G3 troponin increase and underlying disease progression). No events of dysgeusia led to dose modifications or treatment discontinuation.

#### Efficacy

**Fig. 2:**
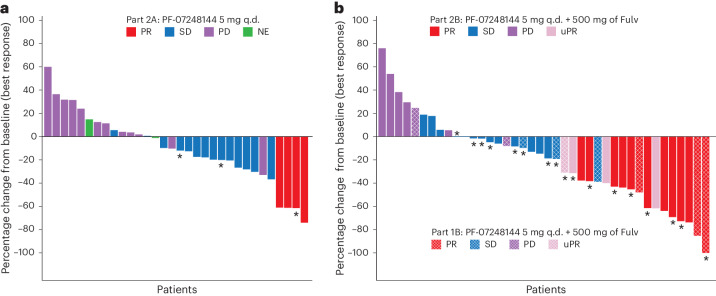
Waterfall plot of tumor size change from baseline. **a**, Waterfall plot of patients in part 2A (PF-07248144 5 mg q.d.). **b**, Waterfall plot of patients in parts 1B and 2B (PF-07248144 5 mg q.d. + fulvestrant 500 mg). Largest decrease or smallest increase represents the best response to treatment. uPR, unconfirmed PR (considered as SD in Table 3). * indicates ongoing.

**Extended Data Fig. 5:**
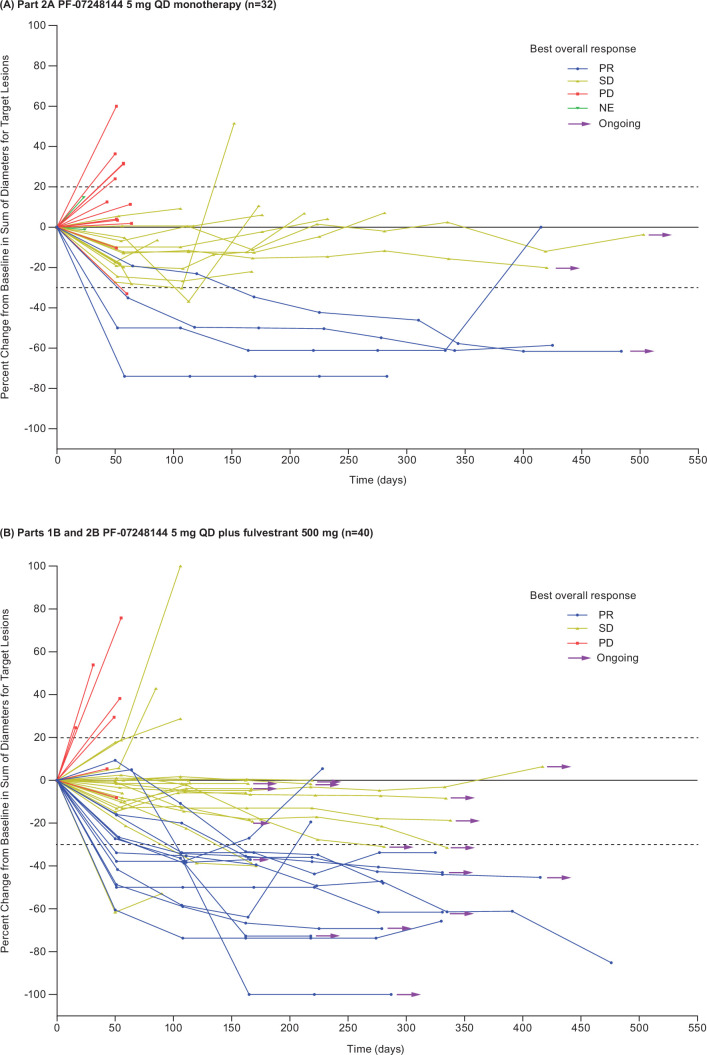
Spider plot of tumor size change from baseline over time in patients with target lesions at baseline and at least one adequate post-baseline assessment. (**a**) Part 2A PF-07248144 5 mg QD monotherapy (n = 32). (**b**) Parts 1B and 2B PF-07248144 5 mg QD plus fulvestrant 500 mg (n = 40). NE, not evaluable; PD, progressive disease; PR, partial response; SD, stable disease.

In the monotherapy group, with a median (range) duration of follow-up of 15.9 (13.8–not evaluable (NE)) months, 4 confirmed partial tumor responses (PRs) were observed out of 35 patients. The ORR was 11.4% (95% CI: 3.2–26.7%), the median (range) duration of response (DOR) was 12.0 (7.4–NE) months and the clinical benefit rate (CBR) was 31.4% (95% CI: 16.9–49.3%). The median PFS (mPFS) was 3.3 (95% CI: 2.0–5.8) months (Table 3, Fig. 2a and Extended Data Figs. 3b and 5a).

**Table 3:** Clinical efficacy of PF-07248144

	PF-07248144 5 mg q.d. Part 2A (*N* = 35)	Combination PF-07248144 5 mg q.d. + fulvestrant 500 mg Parts 1B + 2B (*N* = 43)								
	*N* = 35	Total *N* = 43	2L *N* = 23	3L+ *N* = 20	Fulv treated *N* = 5	Fulv naive *N* = 38	*ESR1* MT *N* = 24	*ESR1* WT *N* = 18	*PIK3CA/AKT1/ PTEN* MT *N* = 19	*PIK3CA/AKT1/ PTEN* WT *N* = 23
Objective response (CR + PR), *n* (%)	4 (11.4)	13 (30.2)	5 (21.7)	8 (40.0)	3 (60.0)	10 (26.3)	8 (33.3)	5 (27.8)	5 (26.3)	8 (34.8)
95% CI^a^	3.2–26.7	17.2–46.1	7.5–43.7	19.1–63.9	14.7–94.7	13.4–43.1	14.5–52.2	7.1–48.5	6.5–46.1	15.3–54.2
Median duration of response (95% CI)^b^	12.0 (7.4–NE)	9.2 (7.2–NE)	NE (5.5–NE)	9.2 (7.2–NE)	N/A	N/A	9.2 (5.8–NE)	NE (NE–NE)	7.2 (5.5–NE)	NE (9.2–NE)
Disease control (CR + PR + SD + non-CR/non-PD), *n* (%)	18 (51.4)	33 (76.7)	16 (69.6)	17 (85.0)	5 (100.0)	28 (73.7)	21 (87.5)	12 (66.7)	13 (68.4)	20 (87.0)
95% CI^a^	34.0–68.6	61.4–88.2	47.1– 86.8	62.1–96.8	47.8–100.0	56.9–86.6	67.6–97.3	41.0–86.7	43.4–87.4	66.4–97.2
CBR, *n* (%)	11 (31.4)	22 (51.2)	10 (43.5)	12 (60.0)	4 (80.0)	18 (47.4)	12 (50.0)	10 (55.5)	9 (47.4)	13 (56.5)
95% CI^a^	16.9–49.3	35.5–66.7	23.2– 65.5	36.1– 80.9	28.4–99.5	31.0–64.2	29.1–70.9	30.8–78.5	24.4–71.1	34.5–76.8
mPFS, 95% CI^b^	3.3 (2.0–5.8)	10.7 (5.3–NE)	NE (3.5–NE)	10.7 (5.5–NE)	N/A	N/A	10.7 (5.5–NE)	NE (3.5–NE)	7.2 (2.8–NE)	10.8 (5.6–NE)

^a^Clopper–Pearson method used.

^b^Brookmeyer and Crowley method used.

2L, with at least one prior line of treatment; 3L+, as third line and above; Fulv, fulvestrant; N/A, not available; NE, not evaluable.

**Fig. 3:**
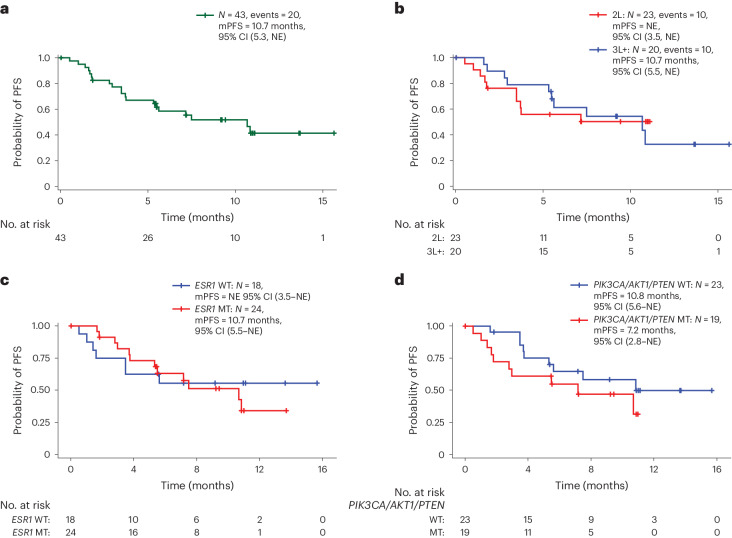
Kaplan–Meier plot of PFS based on investigator response. **a**, Patients in parts 1B and 2B (PF-07248144 5 mg q.d. + fulvestrant 500 mg). **b**, Subgroup analysis by prior lines of therapies: 2L versus 3L+ in patients in parts 1B and 2B (PF-07248144 5 mg q.d. + fulvestrant 500 mg). **c**, Subgroup analysis by *ESR1* mutation status: MT versus WT in patients in parts 1B and 2B (PF-07248144 5 mg q.d. + fulvestrant 500 mg). **d**, Subgroup analysis by *PIK3CA/AKT1/PTEN* mutation status: MT versus WT patients in parts 1B and 2B (PF-07248144 5 mg q.d. + fulvestrant 500 mg).

In the combination group, with a median (range) duration of follow-up of 9.2 (7.2–11.0) months, 13 confirmed PRs were observed out of 43. The ORR (95% CI) was 30.2% (17.2–46.1%) and the median (range) DOR 9.2 (7.2–NE) months. The CBR was 51.2% (95% CI: 35.5–66.7%) and the mPFS 10.7 months (*n* = 43; 95% CI: 5.3–NE months) (Table 3, Figs. 2b and 3a, and Extended Data Figs. 3c and 5b).

To further explore the antitumor activity, exploratory analyses by prior lines of systemic therapies in the post-CDK4/6 inhibitor setting and by prior exposure to fulvestrant treatment were performed.

Twenty-three patients received PF-07248144 and fulvestrant as second-line systemic therapy in the advanced/metastatic setting post-CDK4/6 inhibitors. The ORR was 21.7% (95% CI: 7.5–43.7%) and the CBR 43.5% (95% CI: 23.2–65.5%). With a median duration of follow-up of 9.4 (7.2–11.0) months, the mPFS was not reached. Twenty patients received PF-07248144 and fulvestrant as the third line and above (3L+) systemic therapy after CDK4/6 inhibitors; the ORR was 40.0% (95% CI: 19.1–63.9%) and the CBR 60.0% (95% CI: 36.1–80.9%). With a median duration of follow-up of 9.2 (9.2–13.7) months, the mPFS was 10.7 months (95% CI: 5.5–NE months) (Table 3 and Fig. 3b).

Five patients received prior fulvestrant in the advanced/metastatic setting. There were three confirmed PRs with an ORR of 60% (95% CI: 14.7–94.7%). Thirty-eight patients were fulvestrant naive; the ORR was 26.3% (95% CI: 13.4–43.1%) (Table 3).

To explore the efficacy by gene mutation status with approved targeted therapies, post hoc analyses based on ctDNA mutation profiling, including *ESR1* and *PIK3CA/PTEN/AKT1* genes, was investigated in plasma samples collected at baseline.

The biomarker analysis cohort included 42 of 43 patients with baseline ctDNA results in the combination group. Of the patients, 57% (24 of 42) and 45.2% (19 of 42) had one or more gene mutations in *ESR1* and *PIK3CA/PTEN/AKT1* genes, respectively, at baseline. Durable antitumor activity of PF-07248144 in combination with fulvestrant was observed in patients regardless of mutation status in *ESR1* and/or *PIK3CA/PTEN/AKT1* (Table 3 and Fig. 3c,d).

In the *ESR1* mutant subgroup (*n* = 24), the ORR was 33.3% (95% CI: 14.5–52.2), the median (range) DOR was 9.2 (5.8–NE) months, the CBR 50.0% (95% CI: 29.1–70.9%) and mPFS 10.7 (95% CI: 5.5–NE) months. In the *ESR1* wild-type (WT) subgroup (*n* = 18), ORR was 27.8% (95% CI: 7.1–48.5%), CBR 55.5% (95% CI: 30.8–78.5%) and the mPFS was not reached (Table 3 and Fig. 3c).

In the *PIK3CA/PTEN/AKT1* mutant subgroup (*n* = 19), the ORR was 26.3% (95% CI: 6.5–46.1%), the median (range) DOR 7.2 (5.5–NE), the CBR 47.4% (95% CI: 24.4–71.1%) and mPFS 7.2 (95% CI: 2.8–NE) months. In the *PIK3CA/PTEN/AKT1* WT subgroup (*n* = 23), the ORR was 34.8% (95% CI: 15.3–54.2%), CBR 56.5% (95% CI: 34.5–76.8%) and mPFS 10.8 (95% CI: 5.6–NE) months (Table 3 and Fig. 3d).

#### PK and pharmacodynamics

**Extended Data Fig. 6:**
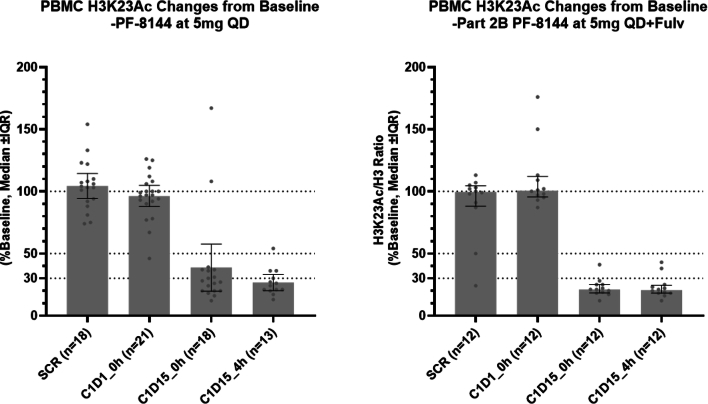
PBMC H3K23AC from part 2A and part 2B. Patients with ER + HER2− mBC. In part 2A, patients were treated with PF-07248144 5 mg QD monotherapy. In part 2B, patients were treated with PF-07248144 5 mg QD plus fulvestrant 500 mg. Data are presented as median (column) ± interquartile ranges (solid lines on each column). C, cycle; D, day; ER, estrogen receptor; Fulv, fulvestrant; HER2, human epidermal growth factor receptor 2; mBC, advanced/ metastatic breast cancer; PMBC, peripheral blood mononuclear cell; QD, once a day; SCR, screening; IQR, interquartile ranges.

Steady-state exposure to PF-07248144 at 5 mg q.d. was similar between PF-07248144 monotherapy and fulvestrant combination therapy (Extended Data Table 3). The median H3K23Ac PD reduction >70% was achieved in blood from patients treated with 5 mg q.d. monotherapy and fulvestrant combination at C1D15 (Extended Data Fig. 6). A strong H3K23Ac reduction was also observed from patients treated with 5 mg q.d. of PF-07248144 monotherapy (median change from baseline: −51.3%, interquartile range (IQR): −58.4%, −50.9%; *n* = 5) and fulvestrant combination (change from baseline: −98%; *n* = 1) in the tumor (Extended Data Fig. 2c).

#### Changes of ctDNA and *ESR1* mutations in plasma in patients treated with PF-07248144 + fulvestrant

**Extended Data Fig. 7:**
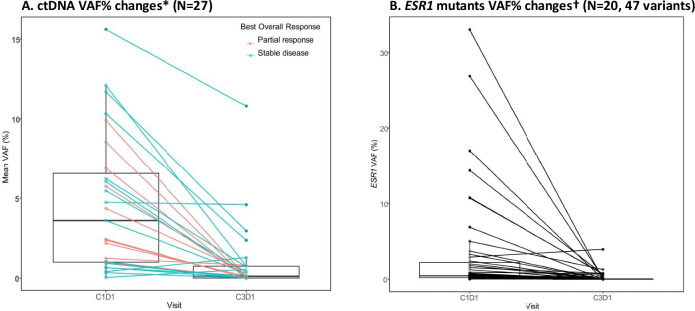
ctDNA analysis of patients in part 1B and part 2B treated with PF-07248144 5 mg QD plus fulvestrant 500 mg. * Changes of mean VAF of all detectable mutants. † Changes of single VAF of *ESR1* mutants. Multiple mutants present from individual samples. VAF, variant allele frequency. Data are presented as median (middle line in the box) and interquartile ranges (top and bottom lines in the box).

To explore how ctDNA and *ESR1* mutation changes after treatment, plasma samples were collected from patients who received PF-07248144 5 mg q.d. in combination with fulvestrant before treatment (C1D1) and 8 weeks after treatment (C3D1). The ctDNA (mutation burden) change was evaluable in 27 patients. Median (IQR) change of ctDNA mean variant allele frequency (VAF) was −95% (−100%, −77%). In addition, *ESR1* VAF change was evaluable in 47 *ESR1* variants detected in 20 patients. PF-07248144 fulvestrant combination reduced *ESR1* VAF to almost undetectable in most patients. Median (IQR) change of *ESR1* VAF was −100% (−100%, −100%) (Extended Data Fig. 7).

## Discussion

Epigenetic targeting represents one of the most promising approaches in both treatment and reversibility of drug resistance^15^. However, epigenetic drugs have faced challenges owing to the complexity of epigenetic regulation and intrinsic heterogeneity of human cancer, as well as off-target effects and unfavorable drug properties. A deeper understanding of the target biology, including the precise impact of histone modification on transcription, has been necessary. Development and refinement of selective and potent epigenetic drugs with good PK exposure and effective target pharmacodynamic engagement have also been required to overcome these challenges.

Several HDAC inhibitors have been approved for the treatment of hematological malignancies. However, HDAC inhibitors have shown limited success in the treatment of solid tumors. In hormone receptor-positive (HR^+^)/HER2^−^ mBC, although there was early enthusiasm for HDAC inhibitors^16^, two pivotal clinical trials of HDAC inhibitors showed divergent results^17^. In the E2112 phase 3 trial, for patients with HR^+^/HER2^−^ mBC whose disease progressed after aromatase inhibitors, the HDAC inhibitor entionostat plus ET demonstrated an mPFS of 3.3 months. The most common G3 and G4 AEs were hematological toxicities^17^. Additional research has suggested that HDAC inhibitors could promote breast cancer metastasis in preclinical models^18^.

A reversed biological process of histone deacetylation is histone acetylation which is catalyzed by HATs. KAT6A and KAT6B are KATs that regulate lineage-specific gene transcription via H3K23Ac. *KAT6A* was first identified as a recurrent cytogenetic translocation (t8;16)(p11;p13) in a subset of AML. *KAT6A* and *KAT6B* have not been established as major drivers in tumorigenesis as a result of the lack of high-frequency somatic oncogenic mutations. Although early preclinical data suggested that inhibition of the histone acetyltransferases KAT6A and 6B induces senescence and arrests tumor growth in cell lines and animal models^7,13^, it is difficult to predict the translatability in the clinical setting, given the complexity and heterogeneity of human cancer.

PF-07248144 is a potent and selective catalytic KAT6A and KAT6B inhibitor. In the present study, we reported the safety, efficacy, PK/pharmacodynamic and biomarker results of the phase 1 study of PF-07248144 as monotherapy and in combination with fulvestrant. We have demonstrated a tolerable safety profile of PF-07248144 and promising and durable clinical efficacy, especially in combination with fulvestrant in heavily pretreated ER^+^HER2^−^ mBC.

Patients with HR^+^/HER2^−^ mBC who have progressed after the first-line CDK4/6 inhibitors and ET represent a high unmet medical need. For all comers without biomarker selection, recent trials demonstrated that standard-of-care fulvestrant monotherapy provided modest clinical benefit with a CBR of 13.7% and mPFS of 2 months^19,20^.

For biomarker subgroups with available therapies: in patients with *ESR1* mutations, the oral selective ER downregulator elacestrant was recently approved based on the positive result of the phase 3 EMERALD trial with an mPFS of 3.8 (95% CI: 2.2–7.3) months^20,21^. In patients with *PIK3CA* mutations in the post-CDK4/6 inhibitor setting, the phase 2 Bylieve trial evaluated the safety and efficacy of α-selective phosphoinositide 3-kinase inhibitor alpelisib in combination with fulvestrant, and demonstrated an ORR of 17% (95% CI: 11–25%), CBR of 46% (95% CI: 36–55%) and mPFS of 7.3 (95% CI: 5.6–8.3) months^22^. In patients with *PIK3CA/AKT1/PTEN* alterations, the AKT inhibitor capivasertib with fulvestrant was recently approved, based on the positive result of the phase 3 CAPItello-291 where the mPFS was 7.3 (95% CI: 5.5–9.0) months. In the post-CDK4/6 inhibitor setting, the mPFS was 5.5 (95% CI: 3.9–6.8) months^23^.

Cross-trial comparison with phase 1 data could be challenging, given the small sample size, nonrandomization, baseline patient characteristics, prior line therapies and other prognostic factors. However, as an early signal finding in the post-CDK4/6 inhibitor setting in the phase 1 study, the KAT6 inhibitor PF-07248144 + fulvestrant (*n* = 43) demonstrated an ORR of 30.2% (95% CI: 17–46.1%), median DOR of 9.2 months (7.2 months, NE), CBR of 51.2% (95% CI: 35.5–66.7%) and mPFS of 10.7 months (5.3 months, NE). Consistent antitumor activity was observed in patients with 2L and 3L+, fulvestrant-treated and fulvestrant-naive, and all biomarker subgroups, regardless of *ESR1*, *PIK3CA*, *AKT1* and *PTEN* mutation status. These findings provide clinical proof of concept that KAT6A and 6B are druggable targets in the clinic and open the potential as a new class of epigenetic drugs for the treatment of ER^+^HER2^−^ mBC.

Although, with PF-07248144 monotherapy, target tumor lesion reductions were observed in more than half of the patients, the observed ORR, CBR and PFS were higher in those patients treated in combination with fulvestrant. One possibility for this difference is that patients who received monotherapy had more prior lines of systemic therapy in the metastatic setting. However, in the fulvestrant combination arm, subgroup analysis suggested that there were consistent antitumor effects between patients treated in 2L (*n* = 23) and 3L+ (*n* = 20). In fact, the observed ORR and CBR were numerically higher in 3L+ when combined with fulvestrant (ORR = 40% (95% CI: 19.1–63.9%); CBR = 60% (95% CI: 36.1–80.9%)). In addition, in five patients pretreated with fulvestrant, three confirmed responses were observed when rechallenged with fulvestrant in combination. This finding may suggest that PF-07248144 in combination with fulvestrant can overcome endocrine resistance, resensitize ET and create potential efficacy synergy, consistent with preclinical observations^13^.

In mBC, ctDNA testing has been used for treatment tailoring, tracking mechanisms of drug resistance and predicting disease response^24^. Plasma ctDNA levels largely depend on tumor burden and tumor cell turnover. Early ctDNA reduction has been reported to have the potential as an early response biomarker and in predicting clinical outcomes for fulvestrant in combination with a CDK4/6 inhibitor^25^. We evaluated ctDNA and *ESR1* mutational burden changes from baseline after 8 weeks of drug treatment. Consistent with the broad and durable antitumor activity observed, PF-07248144 5 mg q.d. + fulvestrant reduced ctDNA in 92.6% (25 of 27) of the evaluable patients (Extended Data Fig. 7). This was further supported by the clearance of *ESR1* mutant(s) in most patients treated with PF-07248144 5 mg q.d. + fulvestrant, whereas only weak or moderate *ESR1* variant frequency reduction has been reported with fulvestrant alone^26^. These preliminary biomarker data provide more evidence for the efficacy of PF-07248144 in combination with fulvestrant and valuable insights to further understand the mechanism of action by KAT6 inhibition in ER^+^HER2^−^ mBC. Further investigation is ongoing to test whether ctDNA changes correlate with PFS and if any clonal mutations emerge when patients progress in this study.

This phase 1 study also revealed remarkable inhibition of KAT6A and 6B catalytic activity from both blood PBMCs and on-treatment tumor samples in patients with cancer, indicating an on-target effect. Preclinical research showed that KAT6A and 6B inhibition downregulates ER signaling, inhibits the cell cycle and induces cell senescence^7,13^. Modulation of proteins such as ER and cyclin D1 and/or whole transcriptome gene expression analysis on tumor paired biopsies is ongoing to assess how PF-07248144 regulates the tumor microenvironment and specific signaling pathways. Furthermore, comprehensive genome and transcriptome analysis of baseline archival tumor tissue is under way to identify potential, translatable, predictive clinical biomarkers. As a result of the limited sample size for evaluable patients with both biomarker and efficacy in the phase 1 study, window-of-opportunity trials and future biomarker-driven studies will be critical to understand the mechanism of action of PF-07248144 in the clinical setting.

At the RDE dose of 5 mg q.d. (*n* = 78), the most frequent TRAE was dysgeusia (84.6%), with the majority as G1 (65.4%). No treatment discontinuation was reported as a result of dysgeusia. Dysgeusia was reported as a frequent TEAE in taxane- or platinum-based chemotherapy ranging from 42% to 93%^27–29^. Dysgeusia has been reported as a frequent TEAE in immunotherapy T cell engagers^30^, other epigenetic inhibitors^31,32^ and HDAC inhibitors^33^. Preliminary preclinical research has shown that epigenetic changes of gene clusters in the taste bud might alter taste^34^. However, the exact mechanism of action of dysgeusia related to KAT6 inhibition remains unknown and requires further investigation. It is interesting that patients have reported symptom improvement or resolution after prolonged treatment interruption or discontinuation, suggesting a reversible process.

The interpretation of the results in the present study is limited by its single-arm design, lack of direct comparison with other treatment options, the small sample size in biomarker subgroups, different patient baseline characteristics and prior line of therapies, as well as tumor heterogeneity. Nevertheless, PF-07248144 presented meaningful and durable clinical responses with predictable PK and a manageable safety profile for patients with HR^+^/HER2^−^ mBC. The data support further exploration of combination therapy strategies with PF-07248144 in later-phase larger studies.

In summary, the findings from this phase 1 study establish KAT6A and KAT6B as druggable cancer targets and provide clinical proof of concept in treating HR^+^/HER2^−^ mBC. This agent and drug class may open a previously unknown avenue to treat HR/HER2^−^ BC and could also have profound implications to stimulate both scientific and clinical research on targeting new epigenetic machinery in solid tumors.

## Methods

### Study design

The present study was conducted in accordance with the Declaration of Helsinki and the Council for International Organizations of Medical Sciences International Ethical Guidelines. It followed all applicable guidelines, laws and regulations. The protocol was approved by the ethics committee or the institutional review board (IRB). All patients provided written informed consent.

The following independent ethics committee or IRB provided approval of the study: Bellberry Human Research Ethics Committee, Eastwood, South Australia, Australia; Royal Melbourne Hospital Human Research Ethics Committee, Parkville, Victoria, Australia; St John of God Health Care Human Research Ethics Committee, Perth, Western Australia, Australia; National Cancer Center IRBc, Chuo-ku, Tokyo, Japan; Kanagawa Cancer Center IRB, Yokohama, Kanagawa, Japan; Aichi Cancer Center Hospital IRB, Nagoya, Aichi, Japan; Seoul National University Bundang Hospital IRB, Seongnam, Gyeonggi-do, Republic of Korea; IRB of Samsung Medical Center, Seoul, Seoul-Teukbyeolsi (Seoul), Republic of Korea; Seoul National University Hospital IRB/IEC, Seoul, Seoul-Teukbyeolsi (Seoul), Republic of Korea; Asan Medical Center IRB, Seoul, Republic of Korea; Severance Hospital, Yonsei University Health System IRB, Seoul, Seoul-Teukbyeolsi (Seoul), Republic of Korea; IRB of Kyungpook National University Chilgok Hospital, Daegu, Taegu-Kwangyǒkshi, Republic of Korea; Western IRB, Puyallup, Washington, USA; Salus IRB, Austin, TX, USA; Advarra, Inc, Columbia, MA, USA; UCSF Human Research Protection Program, San Francisco, CA, UA; U.T. M.D. Anderson Cancer Center IRB, Houston, TX, USA; University of Louisville IRB no. 1, Biomedical, Louisville, KY, USA.

The present study had dose escalation (parts 1A and 1B) and dose expansion (parts 2A and 2B) parts (Extended Data Fig. 1), in which PF-07248144 was administered orally q.d. on a continuous basis in 28-day cycles. In part 1A, patients received escalating doses of PF-07248144 monotherapy at 1–15 mg. In part 1B, patients received PF-07248144 at dose levels of 1–5 mg plus fulvestrant administered intramuscularly at 500 mg (ref. ^14^). Dose escalation, identification of the MTD and the RDE were guided by a Bayesian logistic regression model. In parts 2A and 2B, patients received PF-07248144 monotherapy and PF-07248144 + fulvestrant, respectively, at the RDE determined in the dose escalation parts.

Enrollment of participating patients started in November 2020 and is ongoing; the data cutoff was 30 September 2023. Data were collected by clinical investigative sites as described in the study-specific clinical protocol. Sex was collected and data are included in Table 1. Predefined analysis based on sex was not conducted, in accordance with protocol and statistical analysis plan of this phase 1 study.

The present study is registered at clinicaltrial.gov (registration: NCT04606446). The study protocol and statistical analysis plan are available as Supplementary Information.

### Patients

Patients were aged ≥18 years with an Eastern Cooperative Oncology Group (ECOG) performance status (PS) of 0 or 1 and adequate bone marrow, renal and liver function.

Part 1A included patients with ER^+^HER2^−^ locally advanced BC or mBC, CRPC or NSCLC that was resistant to or intolerant of standard therapy or for whom no standard therapy was available. Parts 1B and 2A included patients with ER^+^HER2^−^ mBC (2L+) whose disease had progressed after at least one prior line of CDK4/6 inhibitor and one prior line of ET in the advanced or metastatic setting. Part 2B included patients with ER^+^HER2^−^ mBC (2–4L) whose disease had progressed after at least one prior line of CDK4/6 inhibitor and one prior line of ET in the advanced or metastatic setting. Patients should not have received more than three prior lines of systemic therapies including up to one line of cytotoxic chemotherapy for visceral disease in the advanced or metastatic setting; prior fulvestrant treatment was allowed but not required.

Patients did not receive compensation for participation in the present study.

#### Inclusion criteria

Patients were eligible if they met all the following criteria: (1) adults aged ≥18 years (aged ≥20 years in Japan; aged ≥19 years in the Republic of Korea); (2) for part 1A (monotherapy dose escalation), patients were required to have histological or cytological diagnosis of locally advanced or metastatic ER^+^HER2^−^ breast cancer, locally advanced or metastatic CRPC or locally advanced or metastatic NSCLC that was intolerant of or resistant to standard therapy or for which no standard therapy was available; (3) for part 1B (combination dose escalation), patients were required to have histological or cytological diagnosis of locally advanced or metastatic ER^+^HER2^−^ breast cancer; eligible patients must have progressed after at least one prior line of treatment with an ET and CDK4/6 inhibitor in the advanced or metastatic setting; (4) for parts 1A and 1B, intolerance or progression on prior therapies must have been documented for study enrollment; (5) for part 2A (ER^+^HER2^−^ breast cancer 2L+, monotherapy), patients were required to have histological or cytological diagnosis of locally advanced or metastatic ER^+^HER2^−^ breast cancer. Eligible patients must have progressed after at least one prior line of CDK4/6 inhibitor and at least one prior line of ET; (6) for part 2B (ER^+^HER2^−^ breast cancer 2–4L, combination with fulvestrant), patients were required to have histological or cytological diagnosis of advanced or metastatic ER^+^HER2^−^ breast cancer. Eligible patients must have progressive disease (PD) after at least one prior line of a CDK4/6 inhibitor and at least one prior line of ET; in addition, eligible patients must not have received more than three prior lines of systemic therapies including up to one line of cytotoxic chemotherapy for visceral disease in advanced or metastatic setting, although eligible patients may have but were not required to have prior treatment with fulvestrant; (7) patients with ER^+^HER2^−^ advanced BC or mBC must have documentation of an ER^+^ tumor (≥1% positive stained cells) based on the most recent tumor biopsy (unless non-measurable disease where the most recent documentation will be provided), utilizing an assay consistent with local standards; (8) patients with ER^+^HER2^−^ advanced BC or mBC must have documentation of a HER2^−^ tumor that was determined as immunohistochemistry (IHC) score 0/1+ or negative by in situ hybridization (FISH/CISH/SISH/DISH) defined as a HER2:CEP17 ratio <2 or for single probe assessment a HER2 copy number <4; (9) female patients with ER^+^HER2^−^ advanced BC or mBC considered to be of childbearing potential (or have tubal ligations only) must be willing to undergo medically induced menopause by treatment with the approved luteinizing hormone-releasing hormone agonist, such as goserelin, leuprolide or equivalent agents, to induce chemical menopause; (10) female patients with ER^+^HER2^−^ advanced BC or mBC of nonchildbearing potential must meet at least one of the following criteria of achieving postmenopausal status, defined as follows: cessation of regular menses for at least 12 consecutive months with no alternative pathological or physiological cause (a serum follicle-stimulating hormone level confirming the postmenopausal state), have undergone a documented hysterectomy and/or bilateral oophorectomy, and with medically confirmed ovarian failure; (11) patients must have at least one measurable lesion as defined by RECIST v.1.1 (Response Evaluation Criteria in Solid Tumors) that has not been previously irradiated; (12) Eastern Cooperative Oncology Group performance status (ECOG PS) 0 or 1; (13) adequate bone marrow function, including absolute neutrophil count ≥ 1,500 mm^−3^ or ≥1.5 × 10^9^ l^−1^, platelets ≥100,000 mm^−3^ or ≥100 × 10^9^ l^−1^ and hemoglobin ≥9 g dl^−1^; (14) adequate renal function, including serum creatinine ≤1.5× upper limit of normal (ULN) or estimated creatinine clearance glomerular filtration rate ≥ 60 ml min^−1^ (≥50 ml min^−1^ for part 2 dose expansion was acceptable) as calculated using the standard method for the institution; in equivocal cases, a 24-h urine collection test can be used to estimate the creatinine clearance more accurately; (15) adequate liver function, including total serum bilirubin ≤1.5× ULN unless the patient had documented Gilbert’s syndrome, aspartate aminotransaminase (AST) and ALT ≤ 2.5× ULN, or AST and ALT ≤ 3.0× ULN if there was liver involvement by the tumor for part 1 dose escalation or AST and ALT ≤ 5.0× ULN if there was liver involvement by the tumor for part 2 dose expansion; (16) resolved acute effects of any prior therapy to baseline severity or Common Terminology Criteria for Adverse Events (CTCAE) grade ≤1 except for AEs not constituting a safety risk by investigator judgment; (17) patients who were willing and able to comply with all scheduled visits, treatment plans, laboratory tests, lifestyle considerations and other study procedures; and (18) patients who were capable of giving signed informed consent.

#### Exclusion criteria

Patients were excluded from the study if any of the following criteria applied: (1) patients with known symptomatic brain metastases requiring steroids; patients with previously diagnosed brain metastases were eligible if they had completed their treatment and had recovered from the acute effects of radiotherapy or surgery before study entry, had discontinued corticosteroid treatment for these metastases for at least 3 weeks and were neurologically stable for 2 months (requires magnetic resonance imaging (MRI) confirmation); (2) patients with advanced/metastatic, symptomatic, visceral spread, who were at risk of life-threatening complications in the short term (including patients with massive uncontrolled effusions (pleural, pericardial, peritoneal), pulmonary lymphangitis and >50% liver involvement); (3) patients with any other active malignancy within 3 years before enrollment, except for adequately treated basal cell or squamous cell skin cancer, or carcinoma in situ; other indolent cancers that did not interfere with assessment of primary cancer under study may be allowed with prior sponsor approval; (4) major surgery within 3 weeks before study entry; (5) radiotherapy within 3 weeks before study entry; (6) systemic anti-cancer therapy within 3 weeks before study entry; if the last immediate anti-cancer treatment contained an antibody-based agent(s) (approved or investigational), then an interval of 28 d or 5 half-lives (whichever is shorter) of the agent(s) before receiving the study intervention treatment was required; (7) prior irradiation to >25% of the bone marrow; (8) patients with active, uncontrolled bacterial, fungal or viral infection, including (but not limited to) hepatitis B virus, hepatitis C virus, known human immunodeficiency virus (HIV) or acquired immune deficiency syndrome (AIDS)-related illness; HIV-seropositive patients who were healthy and low risk for AIDS-related outcomes could be considered eligible; (9) eligibility criteria for patients who were HIV-positive should be evaluated and discussed with the sponsor’s medical monitor and were to be based on current and past CD4 and T cell counts, history (if any) of AIDS-defining conditions (for example, opportunistic infections) and status of HIV treatment; in addition, the potential for drug–drug interactions was to be taken into consideration; in equivocal cases, with positive serology, those patients with a negative viral load were potentially eligible provided that the other entry criteria were met; (10) unmanageable ascites (limited medical treatment to control ascites was permitted, but all patients with ascites require review by the sponsor’s medical monitor); (11) baseline 12-lead ECG demonstrating clinically relevant abnormalities that may affect participant safety or interpretation of study results (for example, baseline corrected QT interval >470 ms, complete left bundle-branch block, signs of an acute myocardial infarction, ST changes suggestive of active myocardial ischemia, second- or third-degree atrioventricular (AV) block, or serious bradyarrhythmias or tachyarrhythmias); (12) any of the following in the previous 6 months: myocardial infarction, long Q–T syndrome, torsades de pointe, clinically important atrial or ventricular arrhythmias (including sustained ventricular tachyarrhythmia and ventricular fibrillation), serious conduction system abnormalities (for example, bifascicular block, third-degree AV block), unstable angina, coronary/peripheral artery bypass graft, symptomatic congestive heart failure, New York Heart Association class III or IV, cerebrovascular accident, transient ischemic attack, symptomatic pulmonary embolism and/or other clinically important episode of thromboembolic disease and ongoing cardiac dysrhythmias of National Cancer Institute CTCAE ≥ grade 2; for grade 2 atrial fibrillation, may be considered eligible with sponsor approval (for example, if improved to grade 1 with nonurgent medical intervention or chronic grade 2 atrial fibrillation with good rate control with nonurgent medical intervention); if a patient has a cardiac rhythm device/pacemaker placed and corrected QT interval (Fridericia method) >470 ms, the patient could be considered eligible; patients with cardiac rhythm device/pacemaker must be discussed in detail with the sponsor’s medical monitor to judge eligibility; (13) therapeutic anticoagulation; (14) hypertension that could not be controlled by optimal medical therapy (for example, ≥160/100 mmHg); (15) participation in other studies involving investigational drug(s) within 3 weeks before study entry; participation in long-term follow-up of other studies was allowed if no procedures that may interfere with the interpretation of study results were to be performed; (16) known or suspected hypersensitivity or severe allergy to active ingredient/excipients of study drug(s); (17) prior treatment with study drug(s); (18) active inflammatory gastrointestinal (GI) disease, refractory and unresolved chronic diarrhea or previous gastric resection, lap-band surgery or other GI conditions and surgeries that may significantly alter the absorption of PF-07248144 tablets; gastroesophageal reflux disease under treatment was allowed; (19) positive serum or urine pregnancy test (for females of childbearing potential) at screening; (20) other medical or psychiatric condition including recent (within the past year) or active suicidal ideation/behavior or laboratory abnormality that may increase the risk of study participation or, in the investigator’s judgment, make the participant inappropriate for the study; and (21) investigator site staff or Pfizer employees directly involved in the conduct of the study, site staff otherwise supervised by the investigator and their respective family members.

### Objectives and assessments

The primary objective was to assess safety per CTCAE v.5.0 and tolerability for both dose escalation and expansion parts. The PK profile of PF-07248144 was a secondary objective for both parts, whereas antitumor activity per RECIST v.1.1 was an exploratory objective for the dose escalation part and a secondary objective for the dose expansion parts. Other exploratory objectives included pharmacodynamics and potential predictive biomarkers for both parts. Additional planned secondary endpoints not reported in the present study included single-dose PK.

Assessments for safety included dose-limiting toxicity (DLTs; dose escalation part only), TEAEs and TRAEs, as well as laboratory abnormalities.

Blood samples for PK analyses were collected on cycle 1 day 1 (C1D1), C1D8, C1D15, cycles ≥2 day 1 and end of treatment (EOT) in part 1, and on C1D1, C1D15, day 1 cycles 2–4 and EOT in part 2. Concentrations used to generate PK parameters were quantified using validated bioanalytical methods.

Antitumor activity was assessed by investigator based on RECIST v.1.1 including best overall response (BOR), ORR (that is, defined as the proportion of patients with a BOR of complete response (CR) or PR), DOR, disease control rate (DCR, including CR, PR, SD and non-CR/non-PD), CBR (that is, defined as the proportion of patients with a BOR of CR, PR or SD lasting for at least 24 weeks) and PFS. Tumor assessments (computed tomography scans/MRI) were performed every 8 weeks (±7 d) from C1D1 for the first 48 weeks, then every 12 weeks (±7 d) and at EOT.

For pharmacodynamic and biomarker assessments, PBMC samples were collected from all patients during screening, and on C1D1, C1D8 (part 1 only), C1D15, C2D1 and C3D1 and at EOT. Data reported were from screening and C1D1 predose as baseline and C1D1 postdosing and C1D8, C1D15 and C2D1 as on-treatment time points. The H3K23Ac pharmacodynamic biomarker of KAT6 modulation in pre- and on-treatment PBMC samples and tumor biopsies was evaluated by Meso Scale Discovery (MSD) and IHC assays, respectively. The MSD assay was developed to measure H3K23Ac and total histone H3 levels in histone extracts from PBMCs with mouse anti-histone H3 at 2 µg ml^−1^ (Active Motif, cat. no. 39763) as a coating antibody, and rabbit anti-acetyl-histone H3 (Lys23) at 1:10,000 dilution (Millipore Sigma, cat. no. 07-355) and rabbit anti-histone H3 antibody at 1:10,000 dilution (Abcam, cat. no. 1791) as detection antibodies. Tumor H3K23Ac IHC assay was established with rabbit anti-acetyl-histone H3 (Lys23) (clone D6Y7M) antibody at 1:30 dilution (Cell Signaling Technology, cat. no. 14932). Fresh tumor biopsies were collected from baseline (during screening) and on C1D15 in selected patients.

Plasma for cell-free DNA (cfDNA) was collected during screening, and on C1D1, C1D15, C3D1 and at EOT. Data reported were from C1D1 (or screening if C1D1 was not available) as baseline and C3D1 as an on-treatment time point. CtDNA and gene mutations were evaluated by Guardant360 assay (74 gene-panel based, Guardant Health Inc.). Gene mutation status was classified as mutant (MT) or WT. MT was defined as having any missense, nonsense or frameshift mutations or splice-site alterations. WT was defined as having no mutations detected. VAFs were defined as percentages of the variant MT reads over the total number of reads from cfDNA in a sample. Percentage change in ctDNA from baseline was analyzed only for patients with detected mutations either at baseline and/or on-treatment. Mean VAF and percentage change in mean VAF values were provided by Guardant Health Inc.

### Statistical analyses

There was no formal hypothesis testing in the present study.

In the dose escalation parts (parts 1A and 1B) of the present study, patients were to receive escalating doses of PF-07248144 monotherapy (part 1A) or PF-07248144 + fulvestrant (part 1B) until determination of the MTD/RDE. For each dose level tested, it was estimated that each cohort would consist of at least three participants evaluable for DLT assessment (compliance with at least 75% of the planned doses or occurrence of DLT). The total sample size could not be estimated accurately at the start of the study because it depended on the number of doses to be tested until the determination of the MTD/RDE and on the number of participants who would meet the definition of being DLT evaluable.

For the dose expansion parts (part 2A monotherapy, part 2B combination therapy), there was no formal hypothesis testing. A sample size of at least 30 participants in each part was deemed sufficient to monitor clinical activity. Assuming a noninformative prior (that is, Jeffreys prior) if four or more participants had a tumor response out of 30, this would translate into a posterior probability (beta binomial) of >0.746 that the true response rate was not inferior to 10%.

In the dose escalation part, two-parameter and five-parameter Bayesian logistic regression models (BLRMs) were used to model the dose/DLT relationship of PF-07248144 monotherapy (part 1A) and combination therapy (part 1B). A weekly informative prior was used for the two-parameter BLRM to reflect the uncertainty about the dose/DLT relationship of PF-07248144 monotherapy before study start. An informative prior was formed using the accumulated DLT data from PF-07248144 monotherapy from part 1A to set the five-parameter BLRM for PF-07248144 + fulvestrant. The BLRM along with the Escalation With Overdose Control principle was used to guide the dose escalation/de-escalation after accrual of data from each cohort of patients. This design ensured that no dose was administered to patients if the risk of excessive toxicity (DLT rate >33%) was >25%.

The MTD was identified based on the DLT. The criterion to estimate the MTD was based on a >50% posterior probability of the risk of DLT being in the target toxicity interval (0.16, 0.33) and a <25% posterior probability of the risk of DLT being in the overtoxic interval (0.33, 1.00).

The RDE was identified based on safety, efficacy and PK findings. No formal set of criteria was used to determine the RDEs for PF-07248144 monotherapy and PF-07248144 + fulvestrant. The totality of the data (that is, clinical activity, safety, tolerability, biomarkers, PK) was reviewed by the sponsor and the study investigators to identify the dose that provided the best benefit risk for patients.

The 95% CI for ORR, DCR and CBR was based on the Clopper–Pearson method. The Kaplan–Meier method was used to analyze all time to event endpoints, and the 95% CI for mPFS and mDOR was based on the Brookmeyer and Crowley method. Safety data were summarized descriptively. No formal interim analysis was conducted.

Biomarker analyses were performed on patients with reported cfDNA results. To calculate percentage change in *ESR1* VAF, values were shifted by the VAF threshold for positivity (0.001%) divided by 2 to enable percentage change in patients with no detected *ESR1* mutation at baseline, but with detected *ESR1* mutation on-treatment. If patients had multiple *ESR1* mutations, each individual *ESR1* VAF value was included in the analysis.

Summary statistics were generated using SAS v.9.4 software and R v.4.2.1 (2022-06-23).

### Reporting summary

Further information on research design is available in the Nature Portfolio Reporting Summary linked to this article.

## Online content

Any methods, additional references, Nature Portfolio reporting summaries, source data, extended data, supplementary information, acknowledgements, peer review information; details of author contributions and competing interests; and statements of data and code availability are available at 10.1038/s41591-024-03060-0.

### Supplementary information

Supplementary Information (1) Redacted study protocol. (2) Redacted statistical analysis plan.

Reporting Summary

## Figures and Tables

**Extended Data Table 1:**
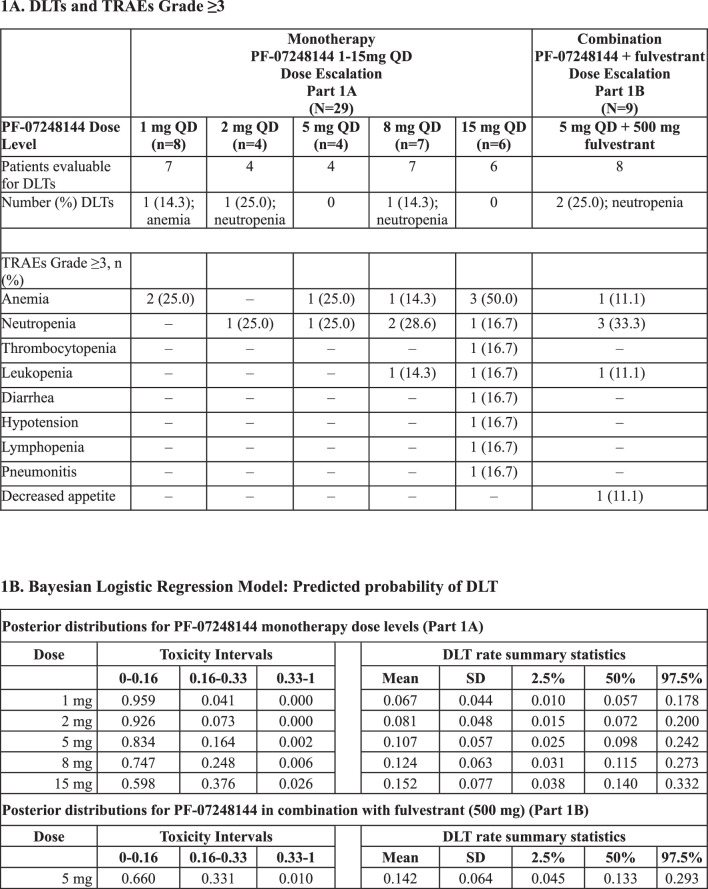
DLTs and TRAEs (Grade ≥3) by dose level in dose escalation

[[footnote for extended Table 1A]] A patient was classified as DLT evaluable if they experienced a DLT or received >75% of the planned doses and had received all scheduled safety assessments during the DLT window.

MedDRA v.26.1 coding dictionary applied. Some are cluster terms. The cluster term ‘anemia’ included the preferred terms ‘anemia’ and ‘hemoglobin decrease’, ‘thrombocytopenia’ included ‘thrombocytopenia’ and ‘platelet count decreased’, ‘leukopenia’ included ‘leukopenia’ and ‘white blood cell count decreased’, ‘lymphopenia’ included ‘lymphopenia’ and ‘lymphocyte count decreased’, ‘neutropenia included ‘neutropenia’ and ‘neutrophil count decreased’.

**Extended Data Table 2:**
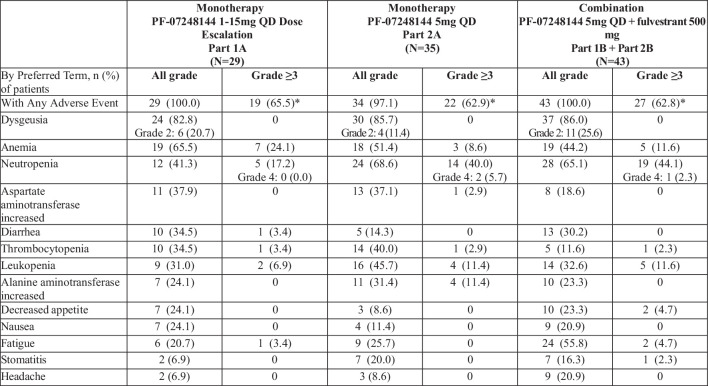
TEAEs by preferred terms in ≥20% of patients in any treatment group—safety analysis set

*Part 1A: 2 grade 4 (1 hyperkalemia and 1 hypercalcemia), 1 grade 5 pneumonitis. Part 2A: 2 grade 4 neutropenia; 1 grade 5 disease progression. Part 1B and part 2B: 1 grade 4 neutropenia.

**Extended Data Table 3:**
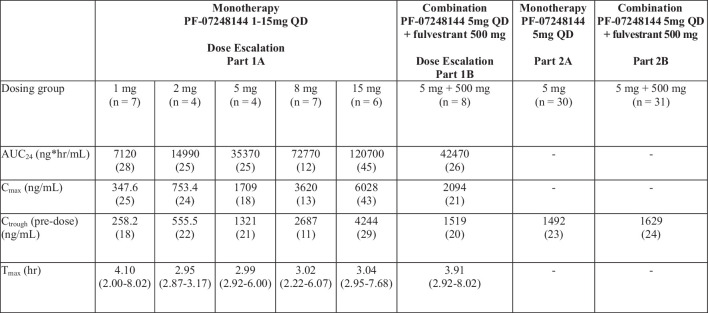
PK parameter summary on cycle 1 day 15

Geometric means (geometric %CV) are presented for all summarized parameters except median (range) for *T*_max_.

## Data Availability

Upon request, and subject to review, Pfizer will provide the data that support the findings of the present study. Subject to certain criteria, conditions and exceptions, Pfizer may also provide access to the related individual de-identified participant data. See https://www.pfizer.com/science/clinical-trials/trial-data-and-results for more information.
