# Author Correction: Inhibition of lysine acetyltransferase KAT6 in ER^+^HER2^−^ metastatic breast cancer: a phase 1 trial

**DOI:** 10.1038/s41591-024-03129-w

**Published:** 2024-06-24

**Authors:** Toru Mukohara, Yeon Hee Park, David Sommerhalder, Kan Yonemori, Erika Hamilton, Sung-Bae Kim, Jee Hyun Kim, Hiroji Iwata, Toshinari Yamashita, Rachel M. Layman, Monica Mita, Timothy Clay, Yee Soo Chae, Catherine Oakman, Fengting Yan, Gun Min Kim, Seock-Ah Im, Geoffrey J. Lindeman, Hope S. Rugo, Marlon Liyanage, Michelle Saul, Christophe Le Corre, Athanasia Skoura, Li Liu, Meng Li, Patricia M. LoRusso

**Affiliations:** 1 National Cancer Center Hospital East, Kashiwa, Japan; 2 Samsung Medical Center, Sungkyunkwan University School of Medicine, Seoul, Republic of Korea; 3 NEXT Oncology, San Antonio, TX USA; 4 National Cancer Center Hospital, Tokyo, Japan; 5 Sarah Cannon Research Institute, Nashville, TN USA; 6 Asan Medical Center, University of Ulsan College of Medicine, Seoul, Republic of Korea; 7 Seoul National University Bundang Hospital, Seoul National University College of Medicine, Seongnam, Republic of Korea; 8 Nagoya City University, Graduate School of Medical Sciences, Nagoya, Japan; 9 Kanagawa Cancer Center, Yokohama, Japan; 10 The University of Texas MD Anderson Cancer Center, Houston, TX USA; 11 Hoag Family Cancer Institute, Newport Beach, CA USA; 12 Saint John of God Subiaco Hospital, Perth, Western Australia Australia; 13 Kyungpook National University Chilgok Hospital, School of Medicine, Kyungpook National University, Daegu, Republic of Korea; 14 Western Health, Sunshine Hospital, St Albans, Victoria Australia; 15 Swedish Cancer Institute, First Hill-True Family Women’s Cancer Center, Seattle, WA USA; 16 Yonsei University College of Medicine, Seoul, Republic of Korea; 17 Seoul National University Hospital, Seoul National University College of Medicine, Cancer Research Institute, Seoul National University, Seoul, Republic of Korea; 18 Peter MacCallum Cancer Centre and Walter and Eliza Hall Institute of Medical Research, Melbourne, Victoria Australia; 19 University of California, San Francisco, CA USA; 20 Pfizer, San Diego, CA USA; 21 Pfizer, Collegeville, PA USA; 22 Pfizer, San Francisco, CA USA; 23 Yale School of Medicine, New Haven, CT USA

**Keywords:** Drug development, Target validation

Correction to: *Nature Medicine* 10.1038/s41591-024-03060-0, published online 1 June 2024

In the version of the article initially published, in Extended Data Fig. 1, under “Part 1B”, “5 mg QD” originally read “3 dose levels” and “n=9” was missing from that box. There was originally an additional “Cohort 1 (5 mg QD, n=9)” box that has now been removed. Under “Part “2A”, the text “fulvestrant 500 mg” originally preceded “(n=35)”. In Extended Data Fig. 5, “(confirmed)” has been removed from both graph legends, which originally read “Best overall response (confirmed)”. In Extended Data Table 1, the title of Table 1B has been corrected from “PF-07248144 dose and DLT relationship” to “Bayesian Logistic Regression Model: Predicted probability of DLT”. These corrections have been made to the HTML and PDF versions of the article.

