# Supplementary material for: Inhibition of lysine acetyltransferase KAT6 in ER+HER2− metastatic breast cancer: a phase 1 trial
Source: Nat Med. 2024 Jun 1;30(8):2242–50. doi: 10.1038/s41591-024-03060-0 (PMC11333285; doi:10.1038/s41591-024-03060-0)
Supplement: Supplementary file 1 — (1) Redacted study protocol. (2) Redacted statistical analysis plan. [file 41591_2024_3060_MOESM1_ESM.pdf]

nature medicine

Supplementary information https://doi.org/10.1038/s41591-024-03060-0

Inhibition of lysine acetyltransferase KAT6 in ER^{+}HER2^{-} metastatic breast cancer: a phase 1 trial

In the format provided by the authors and unedited

A PHASE 1 DOSE ESCALATION AND EXPANSION STUDY TO EVALUATE SAFETY, TOLERABILITY, PHARMACOKINETIC, PHARMACODYNAMIC, AND ANTI-TUMOR ACTIVITY OF PF-07248144 IN PARTICIPANTS WITH ADVANCED OR METASTATIC SOLID TUMORS

**Study Intervention Number:**	PF-07248144
**Study Intervention Name:**	N/A
**US IND Number:**	149778
**EudraCT Number:**	N/A
**Protocol Number:**	C4551001
**Phase:**	1
**Short Title:** Phase 1 Study of PF-07248144 in Advanced or Metastatic Solid Tumors	

[redacted]

090177e19bd138e0\Approved\Approved On: 14-Nov-2022 20:57 (GMT)

DOCUMENT HISTORY

Document	Version Date
Amendment 4	14 November 2022
Amendment 3	14 Dec 2021
Amendment 2	03 Aug 2021
Amendment 1	11 Sep 2020
Original protocol	13 Jul 2020

This amendment incorporates all revisions to date, including amendments made at the request of country health authorities and IRBs/ ECs and any protocol administrative change letter(s).

Protocol Amendment Summary of Changes Table

Amendment 4 (14 November 2022)

Overall Rationale for the Amendment: The primary purpose of this amendment is to include the triplet combination (PF-07248144 + PF-07220060 + fulvestrant) dose escalation and dose expansion to evaluate the clinical safety and provide preliminary proof of concept for the combination in this ongoing C4551001 clinical trial.

PF-07248144 is a selective KAT6 inhibitor and PF-07220060 is a selective CDK4 inhibitor. Extensive preclinical data summarized in Section 4.2 of this amendment demonstrates the potential for synergistic efficacy of this combination in heavily pretreated ER+ breast cancer. Furthermore, the safety and preliminary efficacy of each drug as monotherapy and in combination with fulvestrant have already been characterized in the respective trials C4551001 [redacted]. Monotherapy and fulvestrant combination MRD/RDEs for each have been identified, and the planned starting dose of each in combination is below the MTD/RDE for each agent. These data are summarized in this amendment (Section 2.2.4 and Section 2.2.8) and indicate a favorable benefit to risk. [redacted] They are monitorable with the current protocol-required safety and laboratory tests, and reversible and manageable with dose modification (Section 6.6 - Dose Modification, Table 11 and Table 12).

[redacted]

[redacted]

090177e19bd138e0\Approved\Approved On: 14-Nov-2022 20:57 (GMT)

Section # and Name	Description of Change	Brief Rationale	Substantial or Nonsubstantial
1. Protocol summary; (1.1 Synopsis, 1.2 Schema and 1.3 Schedule of activities); 2.2.8 Overview of PF-07220060; 2.3 Benefit/Risk Assessment; 3. Objectives and endpoints; 4.1 Overall Design; 4.2. Scientific rationale for study design (4.2.2, 4.2.3, and 4.2.7); 4.3. Justification for dose (4.3.2, 4.3.3, 4.3.4, 4.3.5, and 4.3.7) 5. Study population (5.1 and 5.2); 6. Study Intervention (6.1 and 6.1.1.5); 6.2. Preparation/Handling/Storage/ Accountability; 6.5 Concomitant Therapy (6.5.2, 6.5.9 and 6.5.10); 6.6 Dose Modification (6.6.1 and 6.6.2); 8.5 Pharmocokinetics (8.5.1); 8.6 Pharmacodynamics; 8.8 Biomarkers (8.8.1, 8.8.2); 9.2. Sample Size Determination (9.2.1 and 9.2.2); 9.4 Statistical Analysis (9.4.1, 9.4.1.1, 9.4.2, 9.4.3, and 9.4.3.2); 9.4.5.1 Electrocardiogram Analyses; Section 10.5 Appendix 5: Genetics; Section 10.8.1 Japan Specific Requirements; Section 10.14 Appendix 14. Prohibited or Cautioned Concomitant Medications which may result in Pharmacokinetically Driven Drug-Drug Interaction (DDI); Section 10.15 Appendix 15 Prohibited or Cautioned Concomitant Medications which may result in	[redacted]	[redacted].	Substantial

090177e19bd138e0\Approved\Approved On: 14-Nov-2022 20:57 (GMT)

Section # and Name	Description of Change	Brief Rationale	Substantial or Nonsubstantial
[redacted]			
1. Protocol Summary (1.1, 1.2, 1.3); 3. Objectives and Endpoints; 5.1 inclusion Criteria	[redacted]	[redacted]	[redacted]
	Removed requirement for fulvestrant naïve status from Part 2B dose expansion. Updated inclusion criterion #3 for Part 2B	Clinical efficacy of PF-07248144 has been observed in patients who received prior fulvestrant resistant setting during fulvestrant combination dose escalation (Part 1B).	Substantial
2.3. Benefit/Risk Assessment (2.3.1 and 2.3.3)	[redacted]	[redacted]	[redacted]
4.3 Justification for dose (4.3.2, 4.3.3, 4.3.4, and 4.3.5)	[redacted]		[redacted]

090177e19bd138e0\Approved\Approved On: 14-Nov-2022 20:57 (GMT)

Section # and Name	Description of Change	Brief Rationale	Substantial or Nonsubstantial
	[redacted]		
5.1 Inclusion Criteria; 5.2 Exclusion Criteria	[redacted]	[redacted]	[redacted]
	[redacted]	[redacted]	[redacted]
	Inclusion criterion # 3: Added Part 2A with prior changes from PACL (12-May-2022). Participant population for Korea in Part 2A (dose expansion) will remain with 3L+	Korean MFDS requested that Part 2A participant population for Korea will remain with 3L+ as it was approved for C4551001 protocol amendment 1, dated 11 September 2020, in the initial IND.	Substantial
	Exclusion criterion # 1 Replaced 4 weeks with 3 weeks and 3 months with 2 months. Revised to have discontinued corticosteroid treatment for these metastases for at least 3 weeks and are neurologically stable for 2 months (requires MRI confirmation).	To ease the requirement for stable brain metastasis based on clinical investigators' feedback to start study treatment in participants with high unmedt medical need sooner with a minimal safety risk.	Substantial

090177e19bd138e0\Approved\Approved On: 14-Nov-2022 20:57 (GMT)

Section # and Name	Description of Change	Brief Rationale	Substantial or Nonsubstantial
	Exclusion criterion # 3<br>Added the following sentence: "Other indolent cancers that do not interfere with assessment of primary cancer under study may be allowed with prior sponsor approval".	To clarify that participants with other indolent cancers that do not interfere with the assessment of primary cancer under study may be allowed with prior sponsor approval	Substantial
	Exclusion criterion # 4:<br>Replaced 4 weeks with 3 weeks for participants with major surgery	To shorten the exclusion period for prior surgery based on clinical investigators' feedback to start study treatment in participants with high unmet medical need sooner with a minimal safety risk.	Substantial
	Exclusion criterion # 5<br>Replaced 4 weeks with 3 weeks for participants with radiation therapy	To shorten the exclusion period for prior radiation therapy based on clinical investigators' feedback to start study treatment in participants with high unmet medical need sooner with a minimal safety risk.	Substantial
	Exclusion criterion # 11<br>Revise to "For Grade 2 atrial fibrillation, may be considered eligible with sponsor approval (e.g if improved to Grade 1 with non-urgent medical intervention or chronic Grade 2 atrial fibrillation with good rate control with non-urgent medical intervention)."	To ease the exclusion criteria for stable and well-managed grade 2 atrial fibrillation based on clinical investigators' feedback to include participants with high unmed medical need with a low safety risk.	Substantial
	[redacted]	[redacted]	[redacted]

090177e19bd138e0\Approved\Approved On: 14-Nov-2022 20:57 (GMT)

Section # and Name	Description of Change	Brief Rationale	Substantial or Nonsubstantial
	[redacted]		
	Exclusion criterion # 14: Replaced "4 weeks" with "3 weeks" as a duration of participation prior to study entry in other studies involving investigational drug(s).	Based on investigators' request, reducing the washout period to 3 weeks may only carry very small and manageable risk but allow participants to receive potential treatment earlier and reduce prolonged treatment holiday and risk of disease progression.	Substantial
	Exclusion criterion #15: Known or suspected hypersensitivity or severe allergy to active ingredient/excipients of study intervention(s).<br><br>Addition of exclusion criterion # 16: Prior treatment with study intervention(s).	Clarified exclusion criterion #15 and added exclusion criterion #16 to distinguish between "active ingredients/excipients of study intervention(s)" and "study intervention(s)."	Substantial
6.6. Dose Modification (6.6.1 and 6.6.2)	[redacted]	[redacted]	[redacted]

090177e19bd138e0\Approved\Approved On: 14-Nov-2022 20:57 (GMT)

Section # and Name	Description of Change	Brief Rationale	Substantial or Nonsubstantial
	[redacted]		
[redacted]		[redacted]	[redacted]
Global	Other editorial and administrative items were clarified as well.	Administrative corrections and clarifications were made throughout the protocol to improve internal consistency	Non-substantial

090177e19bd138e0\Approved\Approved On: 14-Nov-2022 20:57 (GMT)

TABLE OF CONTENTS

LIST OF TABLES 17
LIST OF FIGURES 18
1. PROTOCOL SUMMARY 19
1.1. Synopsis 19
1.2. Schema 30
1.3. Schedules of Activities 31
2. INTRODUCTION 48
2.1. Study Rationale 48
2.2. Background 48
[redacted]
2.2.4. Clinical Overview for Study C4551001 51
2.2.4.1. Overview of Clinical Pharmacology for PF-07248144....................52
[redacted]
2.2.6. Overview of Fulvestrant 53
[redacted]
2.3. Benefit/Risk Assessment 55
2.3.1. Risk Assessment 56
2.3.2. Benefit Assessment 60
2.3.3. Overall Benefit/Risk Conclusion 60
3. OBJECTIVES AND ENDPOINTS 61
4. STUDY DESIGN 66
4.1. Overall Design 66
4.2. Scientific Rationale for Study Design 69
4.2.1. Preclinical rationale for PF-07248144....................69
[redacted]

090177e19bd138e0\Approved\Approved On: 14-Nov-2022 20:57 (GMT)

4.2.4. Estrogen Receptor (ER) Positive Breast Cancer 73
4.2.5. Prostate Cancer 73
4.2.6. NSCLC 73
4.2.7. Background and Rationale for Biomarker Assessment 74
4.3. Justification for Dose 74
[redacted]
4.3.3. Dose Increment 77
4.3.4. Criteria for Dose Escalation 78
4.3.5. Dose Limiting Toxicity Definition 78
4.3.6. Maximum Tolerated Dose Definition 80
4.3.7. Recommended Phase 2 Dose and Recommended Dose for Expansion Definition 80
[redacted]
4.4. End of Study Definition 82
5. STUDY POPULATION 82
5.1. Inclusion Criteria 82
5.2. Exclusion Criteria 85
5.3. Lifestyle Considerations 88
[redacted]
5.3.2. Contraception 89
5.4. Screen Failures 89
6. STUDY INTERVENTION 89
6.1. Study Intervention(s) Administered 90
6.1.1. Administration 90
6.1.1.1. PF-07248144 90
6.1.1.2. Fulvestrant 91
[redacted]
6.1.1.6. [redacted]
6.2. Preparation/Handling/Storage/Accountability 93

[redacted]

6.2.1. Preparation and Dispensing 94
6.3. Measures to Minimize Bias: Randomization and Blinding....94
6.3.1. Allocation to Study Intervention 94
6.4. Study Intervention Compliance....95
6.5. Concomitant Therapy....95
6.5.1. [redacted]
6.5.2. Fulvestrant [redacted]
6.5.3. Other Antitumor/Anticancer or Experimental Drugs 97
6.5.4. Supportive Care 97
6.5.5. Hematopoietic Growth Factors....97
6.5.6. Anti-Diarrheal, Anti-Emetic Therapy....98
6.5.7. Anti-inflammatory Therapy....98
6.5.8. Corticosteroids....98
6.5.9. Surgery....98
6.5.10. Rescue Medicine....98
6.6. Dose Modification....98
6.6.1. Dosing Interruptions 99
6.6.2. Dose Reductions 100
6.7. Intervention After the End of the Study....104
7. DISCONTINUATION OF STUDY INTERVENTION AND PARTICIPANT DISCONTINUATION/WITHDRAWAL....104
7.1. Discontinuation of Study Intervention 104
7.1.1. Request to Continue Study Intervention....105
7.2. Participant Discontinuation/Withdrawal From the Study 105
7.2.1. Withdrawal of Consent 106
7.3. Lost to Follow-up 106
8. STUDY ASSESSMENTS AND PROCEDURES....107
8.1. Efficacy Assessments....108
8.1.1. Tumor Response Assessments....108
8.1.1.1. Determination of Radiographic Progression in Bone 108

090177e19bd138e0\Approved\Approved On: 14-Nov-2022 20:57 (GMT)

8.1.1.2. Prostate-Specific Antigen 108
8.2. Safety Assessments 109
8.2.1. Physical Examinations 109
8.2.2. Vital Signs 109
8.2.3. Electrocardiograms 109
8.2.4. Clinical Safety Laboratory Assessments 110
8.2.5. Pregnancy Testing 111
8.3. Adverse Events and Serious Adverse Events 111
8.3.1. Time Period and Frequency for Collecting AE and SAE Information 112
8.3.1.1. Reporting SAEs to Pfizer Safety 112
8.3.1.2. Recording Nonserious AEs and SAEs on the CRF 113
8.3.2. Method of Detecting AEs and SAEs 113
8.3.3. Follow-up of AEs and SAEs 113
8.3.4. Regulatory Reporting Requirements for SAEs 113
8.3.5. Exposure During Pregnancy or Breastfeeding, and Occupational Exposure 114
8.3.5.1. Exposure During Pregnancy 114
8.3.5.2. Exposure During Breastfeeding 116
8.3.5.3. Occupational Exposure 116
8.3.6. Cardiovascular and Death Events 116
8.3.7. Disease-Related Events and/or Disease Related Outcomes Not Qualifying as AEs or SAEs 117
8.3.8. Adverse Events of Special Interest 117
8.3.8.1. Lack of Efficacy 117
8.3.9. Medical Device Deficiencies 117
8.3.10. Medication Errors 117
8.4. Treatment of Overdose 118
8.5. Pharmacokinetics 119
8.5.1. Plasma for Pharmacokinetic Analysis of PF-07248144 [redacted]

090177e19bd138e0\Approved\Approved On: 14-Nov-2022 20:57 (GMT)

[redacted]
8.7. Genetics 121
8.7.1. Specified Genetics 122
8.7.2. Banked Biospecimens for Genetics 122
[redacted]
8.9. Immunogenicity Assessments 124
8.10. Health Economics 124
9. STATISTICAL CONSIDERATIONS 124
9.1. Statistical Hypotheses 124
9.2. Sample Size Determination 124
9.2.1. Part 1 Dose Escalation 125
9.2.2. Part 2 Dose Expansion 125
9.3. Analysis Sets 125
9.4. Statistical Analyses 126
9.4.1. Maximum Tolerated Dose Determination 126
9.4.1.1. Stopping Criteria 128
9.4.2. Efficacy Analysis 128
9.4.3. Pharmacokinetic Analysis 129
[redacted]
9.4.5. Safety Analyses 131
9.4.5.1. Electrocardiogram Analyses 132

090177e19bd138e0\Approved\Approved On: 14-Nov-2022 20:57 (GMT)

9.4.5.2. Adverse Events 133
9.4.5.3. Laboratory Test Abnormalities 133
9.4.6. Other Analyse(s) 133
9.5. Interim Analyses 133
9.6. Data Monitoring Committee or Other Independent Oversight Committee 134
10. SUPPORTING DOCUMENTATION AND OPERATIONAL CONSIDERATIONS 134
10.1. Appendix 1: Regulatory, Ethical, and Study Oversight Considerations 134
10.1.1. Regulatory and Ethical Considerations 134
10.1.1.1. Reporting of Safety Issues and Serious Breaches of the Protocol or ICH GCP 134
[redacted]
10.1.3. Informed Consent Process 135
10.1.4. Data Protection 136
[redacted]
10.2. Appendix 2: Clinical Laboratory Tests 142
10.3. Appendix 3: Adverse Events: Definitions and Procedures for Recording, Evaluating, Follow-up, and Reporting 143
10.3.1. Definition of AE 143
10.3.2. Definition of SAE 144
10.3.3. Recording/Reporting and Follow-up of AEs and/or SAEs 146
10.3.4. Reporting of SAEs 149
10.4. Appendix 4: Contraceptive Guidance 150
10.4.1. Male Participant Reproductive Inclusion Criteria 150
10.4.2. Female Participant Reproductive Inclusion Criteria 150
10.4.3. Woman of Childbearing Potential 151
10.4.4. Contraception Methods 152
10.5. Appendix 5: Genetics 154

090177e19bd138e0\Approved\Approved On: 14-Nov-2022 20:57 (GMT)

10.6. Appendix 6: Liver Safety: Suggested Actions and Follow-up Assessments 155
10.7. Appendix 7: ECG Findings of Potential Clinical Concern 157
10.8. Appendix 8: Country-Specific Requirements. 159
10.8.1. Japan Specific Requirements 159
10.8.1.1. Japan Participation, Enrollment and General Safety Monitoring 159
10.8.1.2. Modification and Addition of Exclusion Criteria 160
[redacted]
10.8.1.4. Genetics and Biomarkers 161
[redacted]
10.8.3. Korean Specific Requirements Inclusion Criterion #3 166
Part 2A (Dose Expansion, monotherapy) prior lines of therapy 166
10.9. Appendix 9: Detailed Dose Escalation/DeEscalation Scheme for BLRM Design 166
[redacted]
10.10. Appendix 10. Bone Marrow Reserve in Adults 170
10.11. Appendix 11: RECIST (Response Evaluation Criteria In Solid Tumors) version 1.1 Guidelines 172
10.12. Appendix 12: ECOG Performance Status*[35] 177
10.13. Appendix 13: Alternative Measures During Public Emergencies....................178
10.13.1. Eligibility 178
10.13.2. Telehealth Visits 178
10.13.3. Alternative Facilities for Safety Assessments 179
10.13.3.1. Laboratory Testing 179
10.13.3.2. Imaging..179
10.13.3.3. Electrocardiograms..179
10.13.3.4. Study Intervention 180
10.13.3.5. Home Health Visits 180
10.13.4. Adverse Events and Serious Adverse Events 181

090177e19bd138e0\Approved\Approved On: 14-Nov-2022 20:57 (GMT)

10.13.5. Efficacy Assessments 181
10.13.6. Independent Oversight Committees 181
[redacted]
10.16. Appendix 16: Abbreviations 189
10.17. Appendix 17: Protocol Amendment History....196
11. REFERENCES 204

090177e19bd138e0\Approved\Approved On: 14-Nov-2022 20:57 (GMT)

LIST OF TABLES

Table 1. Schedule of Activities: Dose Escalation (Parts 1A, 1B, 1C and 1D) 31

Table 2. Schedule of Activities: Pharmacokinetic Sampling, Pharmacodynamic/other Biomarker Sampling, and ECG Assessments (for Parts 1A, 1B [redacted]

Table 3. Schedule of Activities: Dose Expansion (Part 2) 41

Table 4. Schedule of Activities: Pharmacokinetic Sampling, Pharmacodynamic/other Biomarker Sampling (for Parts 2A [for participants not participating in food effect substudy], 2B [redacted]

Table 5. Schedule of Activities: Pharmacokinetic Sampling Schedule in Participants in the Food Effect Subset of Part 2A (Monotherapy Dose Expansion)..47

Table 6. PF-07248144 as a Single Agent [redacted]

Table 7. Simulated Incidence of Grade 3/4 Neutropenia for PF-07248144 (monotherapy)...76

[redacted]

Table 13. Safety Laboratory Tests 142

[redacted]

090177e19bd138e0\Approved\Approved On: 14-Nov-2022 20:57 (GMT)

LIST OF FIGURES

[redacted] [redacted]

090177e19bd138e0\Approved\Approved On: 14-Nov-2022 20:57 (GMT)

1. PROTOCOL SUMMARY

1.1. Synopsis

Short Title: Phase 1 Study of PF-07248144 in Advanced or Metastatic Solid Tumors

Background

PF-07248144 is a potent and selective catalytic inhibitor of KAT6 histone acetyltransferases, KAT6A and KAT6B as evidenced by its potent biochemical inhibition of both enzymes. KAT enzymes perform important regulatory functions in cancer and are frequently altered by mutations, translocations, and amplifications. In vitro and in vivo data provide the rationale to investigate KAT6A/6B (KAT6A and KAT6B) as therapeutic targets and to evaluate the safety and potential clinical benefits of PF-07248144 in participants with ER+HER2- breast cancer, CRPC, or NSCLC by inhibiting KAT6A/6B enzymes.

Rationale

The purpose of this FIH study is to evaluate the safety, tolerability, and potential clinical benefits of PF-07248144 as monotherapy or in combination in participants with locally advanced or metastatic ER+HER2- breast cancer, CRPC, or NSCLC whose disease progressed on or who are intolerant to standard therapy.

090177e19bd138e0\Approved\Approved On: 14-Nov-2022 20:57 (GMT)

090177e19bd138e0\Approved\Approved On: 14-Nov-2022 20:57 (GMT)

Objectives and Endpoints

**Part 1: PF-07248144 Monotherapy (Part 1A) and Combination Dose Escalation (Parts 1B[redacted]**	
**Primary Objectives:**	**Primary Endpoints:**
**Part 1A: Monotherapy Dose Escalation**<br><br>• To assess safety and tolerability of escalating dose levels of PF-07248144 in successive cohorts of participants with locally advanced or metastatic ER+ HER2 breast cancer, CRPC, or NSCLC to determine the monotherapy MTD and to select the monotherapy RDE.<br><br>**Part 1B: Combination Dose Escalation**<br><br>• To assess safety and tolerability of PF-07248144 in combination with fulvestrant in participants with locally advanced or metastatic ER+HER2 breast cancer who have progressed after at least 1 prior line of treatment with an endocrine therapy and CDK4/6 inhibitor to determine the combination MTD and to select the combination RDE.<br><br>[redacted]<br><br>[redacted]<br><br>[redacted]<br><br>[redacted]	**Parts 1A, 1B[redacted]**<br><br>• DLTs.<br><br>• AEs as characterized by type, frequency, severity (as graded by NCI CTCAE version 5.0), timing, seriousness, and relationship to study therapy.<br><br>• Laboratory abnormalities as characterized by type, frequency, severity (as graded by NCI CTCAE version 5.0), and timing.

[redacted]

**Part 1: PF-07248144 Monotherapy (Part 1A) and Combination Dose Escalation (Parts 1B [redacted])**	
**Secondary Objective:**	**Secondary Endpoint(s):**
• To evaluate the single- and multiple-dose PK of PF-07248144 when given as monotherapy (Part 1A), in combination with fulvestrant (Part 1B); [redacted]<br>• [redacted]<br>[redacted]	• PK parameters of PF-07248144:<br>• [redacted]<br>[redacted]
**Exploratory Objectives:**	**Exploratory Endpoints:**
• To evaluate the anti-tumor activity of PF-07248144 as a monotherapy<br>• To evaluate the anti-tumor activity of PF-07248144 in combination with: fulvestrant (Part 1B [redacted]	• BOR as assessed by investigator based on RECIST v1.1.<br>• DOR and CBR as assessed by investigator based on RECIST v1.1.<br>• PFS as assessed by investigator based on RECIST v1.1.
**Part 1A CRPC Participants Only**<br>• To monitor and document new bone lesion(s) for CRPC.<br>• To monitor and document biochemical response for CRPC.	• Number of new bone lesions as assessed by radionuclide bone scan based on PCWG3.[1]<br>• PSA levels based on PCWG3.
• To evaluate PD effects following PF-07248144 treatment as a single agent (Part 1A) and in combination with fulvestrant (Part 1B), [redacted]	• [redacted]

090177e19bd138e0\Approved\Approved On: 14-Nov-2022 20:57 (GMT)

**Part 1: PF-07248144 Monotherapy (Part 1A) and Combination Dose Escalation (Parts 1B [redacted]**	
• To explore potential predictive biomarkers of clinical response, mechanisms of action, and mechanisms of resistance to PF-07248144 as a single agent (Part 1A) and in combination with fulvestrant (Part 1B), [redacted]	• [redacted]<br>• [redacted] analyses of [redacted].<br>• Changes in peripheral blood biomarkers of [redacted] hat may be related to response or resistance to treatment.

090177e19bd138e0\Approved\Approved On: 14-Nov-2022 20:57 (GMT)

Part 2: PF-07248144 Monotherapy (Part 2A) and Combinations in Dose Expansion (Parts 2B [redacted])	
**Primary Objectives:**	**Primary Endpoints:**
**Part 2A: ER+HER2- breast cancer 2L+, monotherapy**<br>• To assess safety and tolerability of PF-07248144 monotherapy at the RDE from Part 1A in participants with advanced or metastatic 2L+ ER+HER2- breast cancer who have progressed after at least 1 prior line of CDK4/6 inhibitor and 1 line of endocrine therapy.<br>**Part 2B: 2-4L ER+HER2- breast cancer, combination**<br>• To assess safety and tolerability of PF-07248144 at the RDE in combination with fulvestrant (determined in Part 1B) in participants with advanced or metastatic ER+HER2- breast cancer whose disease has progressed after 1 line of a CDK4/6 inhibitor and at least1 line of endocrine therapy and who must not have received more than 3 lines of systemic therapies including up to 1 line of cytotoxic chemotherapy for visceral disease in advanced or metastatic setting.<br>[redacted]<br>[redacted]	• AEs as characterized by type, frequency, severity (as graded by NCI CTCAE version 5.0), timing, seriousness, and relationship to study therapy.<br>• Laboratory abnormalities as characterized by type, frequency, severity (as graded by NCI CTCAE version 5.0), and timing.

090177e19bd138e0\Approved\Approved On: 14-Nov-2022 20:57 (GMT)

Part 2: PF-07248144 Monotherapy (Part 2A) and Combinations in Dose Expansion (Parts 2B [redacted]	
**Secondary Objectives:**	**Secondary Endpoints:**
• To evaluate antitumor activity of PF-07248144 monotherapy and in combination with fulvestrant (Part 2B) [redacted]	• BOR, DOR, and CBR as assessed by investigator based on RECIST v1.1.<br>• PFS as assessed by investigator based on RECIST v1.1.<br>• TTP as assessed by investigator based on RECIST v1.1.<br>• Overall Survival.
• To evaluate PK of PF-07248144 monotherapy (at RDE from Part 1A) and in combination with fulvestrant (at combination RDE from Part 1B) [redacted]	• Monotherapy and combination cohorts: Trough concentrations of PF-07248144 for selected cycles.
• [redacted]	[redacted]
• To evaluate the effect of food on the PK of PF-07248144 administered (at Part 1A RDE) in a subset of participants in Part 2A (approximately 6 participants).<br>• To evaluate urine PK of PF-07248144 in a subset of participants in Part 2A (at least 6 participants).	• In a subset participants (approximately 6) in Part 2A, PK parameters (C_{max}, T_{max}, AUC_{last}, and as data permit, AUC_{inf}, CL/F, V_z/F, and $t_{1/2}$) of PF-07248144 given with and without food.<br>• The amount of PF-07248144 excreted in urine relative to dose administered (%) and renal clearance (CL_r).

090177e19bd138e0\Approved\Approved On: 14-Nov-2022 20:57 (GMT)

Part 2: PF-07248144 Monotherapy (Part 2A) and Combinations in Dose Expansion (Parts 2B [redacted]	
**Exploratory Objectives:**	**Exploratory Endpoints:**
• To evaluate PD effects of KAT6A/6B inhibition following PF-07248144 treatment as a single agent (Part 2A) and in combination with fulvestrant (2B) [redacted]<br><br>• To explore potential predictive biomarkers of clinical response, mechanisms of action, and mechanisms of resistance as a single agent (Part 2A) and in combination with fulvestrant (2B) [redacted]<br><br>• [redacted]	• [redacted]<br><br>• [redacted]<br><br>• [redacted]<br><br>• [redacted] mutations, [redacted] that may be related to response or resistance to treatment.<br><br>• [redacted]

Overall Design

This is an open-label, multi-center Phase 1 study in adult participants to evaluate safety, tolerability, PK, and PD of PF-07248144 in locally advanced or metastatic selected solid tumors (ER+HER2- breast cancer, CRPC, or NSCLC) and early signs of clinical activity of PF-07248144 as a single agent and in combination in ER+HER2- breast cancer. The overall study design is depicted in the schema (Section 1.2).

Part 1 dose escalation consists of: Part 1A, Part 1B, [redacted].

Part 1A contains dose escalation as monotherapy in participants with locally advanced or metastatic ER+HER2- breast cancer, CRPC, or NSCLC that are resistant or intolerant to standard therapy or for whom no standard therapy is available, to determine the MTD and select the RDE. Participants will receive escalating doses of PF-07248144. BLRM guided by EWOC principle will be used to determine the dose escalation process and the MTD. [redacted] The maximum allowable PF-07248144 dose increment is 100% unless the circumstances are as defined in Section 4.3.2. DLT will be assessed during Cycle 1 (the first 28 days). Each dose level group will be approximately 3 participants, with at least 1 DLT-evaluable participant per cohort in the first 2 cohorts and at least 2 DLT evaluable participants per dose level group in the remaining cohorts for Part 1A. Per BLRM design, expanding additional participants at lower dose levels is allowed to

090177e19bd138e0\Approved\Approved On: 14-Nov-2022 20:57 (GMT)

assess safety. [redacted]

In Part 1B, PF-07248144 in combination with fulvestrant will be evaluated for dose finding in participants with advanced or metastatic ER+HER2- breast cancer (2L+) whose disease has progressed after at least 1 line of treatment with an endocrine therapy and CDK4/6 inhibitor to determine the MTD and RDE for this combination. The definitions of RDE and RP2D are provided in Section 4.3.7. Combination RDE may be different from monotherapy RDE due to potential toxicity overlap or drug-drug interaction.

[redacted]

[redacted]

After the determination of the monotherapy RDE in Part 1A, PF-07248144 will be evaluated in a dose expansion cohort as a monotherapy in locally advanced or metastatic ER+HER2- breast cancer (2L+) who have progressed after at least 1 prior line of CDK4/6 inhibitor and 1 line of endocrine therapy (Part 2A). The definitions of RDE and RP2D are provided in Section 4.3.7. [redacted]

A food effect assessment will take place in a subset of participants (approximately 6) in Part 2A. Each participant will serve as his/her own control in which PF-07248144 will be administered in the morning under "fed conditions" on Cycle 1 Day -7 and under "fasted" conditions on Cycle 1 Day 1. Additional details are provided in Section 4.3.8.

PK and metabolite profiling in both urine and blood will be assessed in a subset of participants (approximately 6) in Part 2A. Both urine and blood samples should be collected from the same participants at screening and on Cycle 1 Day 15 (Sections 8.5.2 and 8.5.3).

After determination of the combination RDE from Part 1B, PF-07248144 in combination with fulvestrant will be evaluated in a dose-expansion combination cohort in participants with advanced or metastatic 2-4L ER+HER2- breast cancer whose disease has progressed after 1 line of a CDK4/6 inhibitor and 1 line of endocrine therapy and who must not have received more than 3 lines of systemic therapies in advanced or metastatic settings (Part 2B). Paired tumor biopsies will be required from at least 5 participants in Part 2B.

[redacted]

090177e19bd138e0\Approved\Approved On: 14-Nov-2022 20:57 (GMT)

[redacted]

Number of Participants

The total number of participants is estimated to be approximately 140 - 200.

Part 1 Dose Escalation: Approximately 70 participants will be enrolled in Part 1 including 25 to 30 participants in Part 1A, 6 to 9 participants in Part 1B, [redacted]

The actual number of participants enrolled will depend on the tolerability of PF-07248144 and the number of dose levels required to identify the MTD/RDE as a monotherapy or in combination.

Part 2: Dose Expansion: Approximately 90 -120 participants are expected to be enrolled in Part 2. Approximately 30 participants each will be enrolled in Parts 2A, 2B, [redacted] with up to an additional approximately 30 patients at an alternative dose(s).

All participants will undergo up to 28 days of screening prior to study entry. Eligible participants will then receive study intervention for up to 2 years, or until disease progression, unacceptable toxicities, a decision by the participant (withdrawal of consent or no longer willing to participate) or investigator to discontinue treatment, or study termination. Any additional treatment beyond 2 years shall be discussed and approved by the sponsor. After EOT, all participants will complete a 28 day post-treatment follow up visit for AEs. Participants in Part 2 dose expansion cohorts will be contacted by telephone approximately every 3 months for survival data collection until end of trial (2 years from last participant first dose), unless otherwise notified by the sponsor. During treatment with study intervention, all cycles will be 28 days in length.

Sample size determination is presented in Section 9.2.

Intervention Groups and Duration

Study intervention will be administered in 28-day cycles. Treatment will continue until PD, unacceptable toxicity, participant refusal, investigator decision, or study termination, whichever occurs first. Additional dosing frequency may be considered after the second dose level if supported by emerging PK, PD and clinical data.

The starting dose for PF-07248144 for this FIH study has been determined to be 8 mg PO QD, based on information derived from the 1 month repeat dose toxicology studies in rats and dogs.

BLRM is used for dose finding until reaching MTD. The definition of MTD is provided in Section 4.3.6.

090177e19bd138e0\Approved\Approved On: 14-Nov-2022 20:57 (GMT)

A dosing diary will be given to each participant to support at home dosing. The participant will be requested to note the date and time of the dose.

Dose modification information is provided in Section 6.6.

Data Monitoring Committee or Other Independent Oversight Committee: No

This is an open-label, non-randomized Phase 1 study. This study will not use a DMC. Discussions between the investigators and the sponsor regarding safety will occur in an ongoing manner at regular teleconferences and/or meetings to determine the safety profile and risk/benefit ratio and determine if further participant enrollment is appropriate. These individual and summary data would also include participants who are determined to be not applicable for DLT assessment.

Statistical Methods

There will be no formal hypothesis testing in this study.

Determination of MTD will be performed using a Per protocol analysis set (evaluable for MTD).

Bayesian adaptive approach:

The dose escalation in Part 1A, and dose finding in Part 1B, [redacted] of the study will be guided by a Bayesian analysis of Cycle 1 DLT data for PF-07248144 as a monotherapy (Part 1A) or in combination (Part 1B, [redacted]). A traditional 2-parameter BLRM will be used to model the dose/DLT relationship of PF-07248144 monotherapy. A more complex BLRM model specifically designed for combinations will be used to model the dose/DLT relationship of PF-07248144 given in combination with fulvestrant, [redacted]

Assessment of participant risk: After each cohort of participants, the posterior distribution for the risk of DLT for new participants at different doses of interest for PF-07248144 will be evaluated. The posterior distributions will be summarized to provide the posterior probability that the risk of DLT lies within the following intervals:

Under-dosing:	[0, 0.16]
Targeted dosing:	[0.16, 0.33]
Overdosing:	[0.33, 1]

The EWOC principle: Dosing decisions are guided by the escalation with overdose control principle. A dose may only be used for newly enrolled participants if the risk of excessive toxicity at that dose is less than 25%.

090177e19bd138e0\Approved\Approved On: 14-Nov-2022 20:57 (GMT)

Prior distributions:

Weakly informative prior distributions based on pre-clinical/expert opinion information will be chosen for the logistic parameters for prior distribution in Part 1A, see Appendix 9 (Section 10.9).

In case of change of the dosing regimen, DLT data accumulated during the dose escalation with the original regimen might be used to form a prior for further BLRM analysis.

A MAP approach might be used to derive the prior distribution for model parameters used in Part 1B, [redacted] based on the data collected in Part 1A and DLT data collected in clinical studies for fulvestrant, [redacted]

Efficacy analysis:

Tumor response will be presented in the form of participant data listings that include, but are not limited to tumor type, dose on Day 1, tumor response at each visit, clinical benefit response, and best overall response. Proportion of participants responding to the treatment will be presented for each dose level. Progression date, death date, date of first response and last tumor assessment date, and date of last contact will be listed. The Kaplan-Meier methods will be used to analyze all time to event endpoints.

090177e19bd138e0\Approved\Approved On: 14-Nov-2022 20:57 (GMT)

1.2. Schema

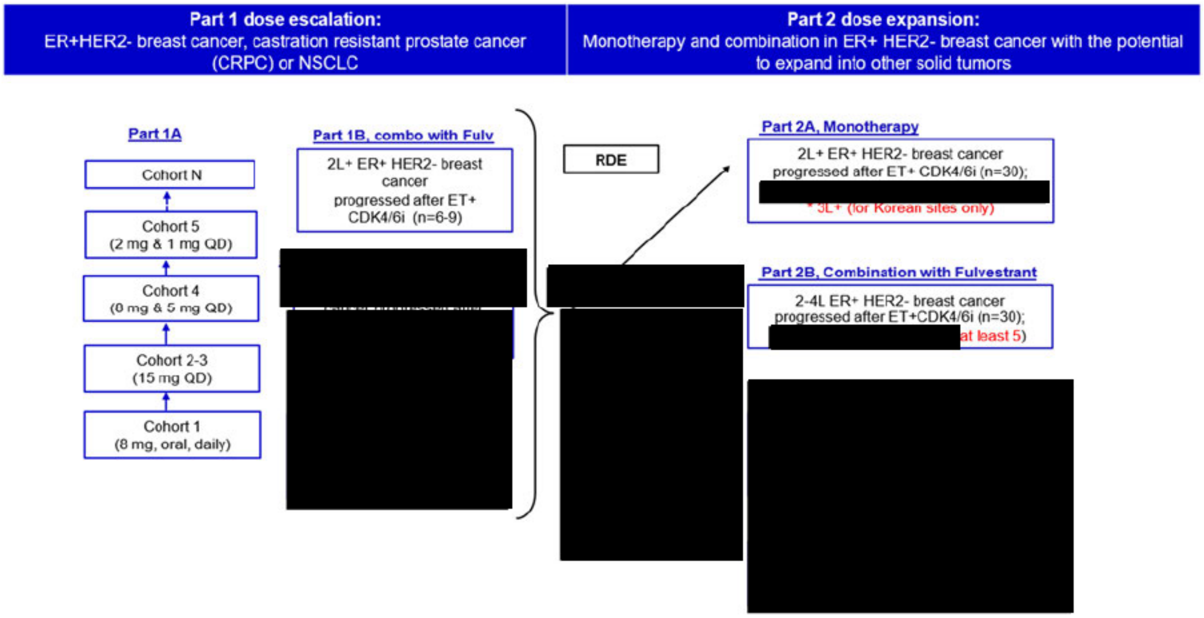

Abbreviation: RDE = recommended dose for expansion

* = More detailed information is provided in Appendix 8 (Section 10.8.3)

090177e19bd138e0\Approved\Approved On: 14-Nov-2022 20:57 (GMT)

090177e19bd138e0\Approved\Approved On: 14-Nov-2022 20:57 (GMT)

1.3. Schedules of Activities

The SoA tables provide an overview of the protocol visits and procedures. Refer to the STUDY ASSESSMENTS AND PROCEDURES of the protocol for detailed information on each assessment required for compliance with the protocol.

The investigator may schedule visits (unplanned visits) in addition to those listed in the SoA tables, in order to conduct evaluations or assessments required to protect the well-being of the participant.

DOSE ESCALATION

Table 1. Schedule of Activities: Dose Escalation (Parts 1A, 1B, [redacted]

		Cycle 1 (28 days)				Cycle 2 (28 days)		Cycles 3+, (every 28 days)			
**Visit Identifier**	**Screening (≤28 days prior to Cycle 1, Day1)** [a]	**Day 1**	**Day 8**	**Day 15**	**Day 22**	**Day 1**	**Day 15**	**Day 1**	**Day 15**	**EOT** [b]	**Post-Treatment Follow-up (28 days after EOT)** [c]
**Visit Window**		±2D	±2D	±2D	±2D	±2D	±2D	±2D	±2D	±7D	+7D
Informed consent [d]	X										
Medical/Oncological history [e]	X										
Full physical examination [f]	X									X	
Brief physical examination [f]		X	X	X	X	X	X	X	X		X
Height	X										
Weight	X	X				X		X		X	X
Vital signs [g]	X	X	X	X	X	X	X	X	X	X	X
ECOG performance status [h]	X	X				X		X		X	X
Contraception check [i]	X	X				X		X		X	
Concomitant medication(s) [j]	X	X	X	X	X	X	X	X	X	X	X
**Laboratory**											
Hematology [k]	X	X	X	X	X	X	X	X	X	X	X
Blood chemistry [k]	X	X	X	X	X	X	X	X	X	X	X

Table 1. Schedule of Activities: Dose Escalation (Parts 1A, 1B, 1C and 1D)

		Cycle 1 (28 days)				Cycle 2 (28 days)		Cycles 3+, (every 28 days)			
**Visit Identifier**	**Screening (≤28 days prior to Cycle 1, Day1)** [a]	**Day 1**	**Day 8**	**Day 15**	**Day 22**	**Day 1**	**Day 15**	**Day 1**	**Day 15**	**EOT** [b]	**Post-Treatment Follow-up (28 days after EOT)** [c]
Coagulation [k]	X	X						X (every 8 weeks)		X	
Urinalysis [k]	X									X	
Pregnancy test [l]	X	X				X		X		X	
PSA (for prostate cancer only) [m]	X					X		X		X	
Viral disease screen (Hepatitis B, C, and HIV) [n]	X										
(Standard 12-lead) ECG	See PK, Biomarker, and ECG table below.										
**Registration and treatment**											
Registration [o]	X										
PF-07248144 administration [p]		Orally, QD continuously.									
[redacted]		[redacted]									
fulvestrant administration [r] (Parts 1B [redacted])		Intramuscularly, 500 mg on C1D1, C1D15, C2D1 and once monthly thereafter.									
[redacted]		[redacted]									
[redacted]		[redacted]									

[redacted]

090177e19bd138e0\Approved\Approved On: 14-Nov-2022 20:57 (GMT)

090177e19bd138e0\Approved\Approved On: 14-Nov-2022 20:57 (GMT)

Table 1. Schedule of Activities: Dose Escalation (Parts 1A, 1B, 1C and 1D)

		Cycle 1 (28 days)				Cycle 2 (28 days)		Cycles 3+, (every 28 days)			
**Visit Identifier**	**Screening (≤28 days prior to Cycle 1, Day1) [a]**	**Day 1**	**Day 8**	**Day 15**	**Day 22**	**Day 1**	**Day 15**	**Day 1**	**Day 15**	**EOT [b]**	**Post-Treatment Follow-up (28 days after EOT) [c]**
Tumor assessments CT/MRI Scans of chest, abdomen, pelvis, any clinically indicated sites of disease; clinical evaluation of superficial disease [t]	X	Performed every 8 weeks (±7 days) from C1D1 for the first 48 weeks and then every 12 weeks (±7 days).								X	
Radionuclide bone scan [u] (prostate cancer only)	X	Performed every 8 weeks (±7 days) up to 2 years and then every 12 weeks (±7 days) from C1D1.								X	
**Other clinical assessments**											
Adverse event monitoring [v]	Continuous.										
Pharmacokinetics blood sampling	See PK, Biomarker, and ECG table below.										
Pharmacodynamics blood and other biomarker sampling	See PK, Biomarker, and ECG table below.										
Pfizer Prep D1 Banked Biospecimen(s)	See PK, Biomarker, and ECG table below.										

a) **Screening:** To be obtained within 28 days prior to C1D1.

b) **EOT Visit:** Visit to be performed as soon as possible after the last dose of study intervention and prior to initiation of any new anti-tumor therapy. Obtain assessments if not completed in the last week on study (or within 6 weeks for tumor assessments).

c) **Post-Treatment Follow up:** At least 28 calendar days, and no more than 35 calendar days after discontinuation of study intervention, participants will return to undergo review of concomitant treatments, vital signs, and assessment for resolution of any treatment-related AEs. Participants continuing to experience treatment-related toxicity after the Post Treatment Follow-up Visit will continue to be followed at least every 28 days until resolution or determination, in the clinical judgment of the investigator, that no further improvement is expected; these may be conducted via telephone.

d) **Informed Consent:** Must be obtained prior to undergoing any study-specific procedures. In Japan, after completion of Cycle 1, participants will be asked to sign an additional consent document for confirmation of the participant's willingness to continue participation in this study before starting Cycle 2.

[redacted]

090177e19bd138e0\Approved\Approved On: 14-Nov-2022 20:57 (GMT)

Table 1. Schedule of Activities: Dose Escalation (Parts 1A, 1B, 1C and 1D)

		Cycle 1 (28 days)				Cycle 2 (28 days)		Cycles 3+, (every 28 days)			
**Visit Identifier**	**Screening (≤28 days prior to Cycle 1, Day1)** [a]	**Day 1**	**Day 8**	**Day 15**	**Day 22**	**Day 1**	**Day 15**	**Day 1**	**Day 15**	**EOT** [b]	**Post-Treatment Follow-up (28 days after EOT)** [c]

e) **Medical/Oncological History:** To include information on oncology disease including details of diagnosis and prior anticancer treatments (systemic treatment, prior surgery and radiotherapy, etc). When available, primary diagnosis history should also include known molecular characteristics of the participant's tumor including mutations, amplifications, etc.

f) **Physical Examination:** Full physical exam at Screening and EOT, brief physical exam at all other timepoints. A symptom-directed exam and an assessment for emergent toxicities or changes from prior visits conducted by a physician, trained physician's assistant, or nurse practitioner, as acceptable according to local regulation.

g) **Vital Signs:** Include oral temperature, pulse rate (PR), respiration rate, blood pressure (BP), and SpO_2. BP and PR to be recorded in the sitting position or semi-recumbent position (the same position should be maintained throughout the study) after approximately 5 minutes of rest.

h) **ECOG Performance Status:** ECOG performance scale is available in Section 10.12.

i) **Contraception Check:** The investigator or his or her designee, in consultation with the participant, will confirm that the participant has selected an appropriate method of contraception for the individual participant (and his or her partner[s]) from the permitted list of contraception methods and will confirm that the participant has been instructed in its consistent and correct use. The investigator or designee will inform the participant of the need to use highly effective contraception consistently and correctly and document the conversation and the participant's affirmation in the participant's chart (participants need to affirm their consistent and correct use of at least 1 of the selected methods of contraception). In addition, the investigator or designee will instruct the participant to call immediately if the selected contraception method is discontinued or if pregnancy is known or suspected in the participant or partner.

j) **Concomitant Treatments:** All concomitant medications and nondrug supportive interventions should be recorded on the CRF.

k) **Hematology, Blood Chemistry, Coagulation, and Urinalysis:** No need to repeat on C1D1 if baseline assessment is performed within 7 days prior to that date (Section 10.2 for a complete list of clinical laboratory tests).

l) **Pregnancy Test:** Pregnancy tests may be urine or serum tests, but must have a sensitivity of at least 25 mIU/mL. Pregnancy tests will be performed in WOCBP. Following a negative pregnancy test result at screening, appropriate contraception must be commenced and a second negative pregnancy test result will be required at the baseline visit prior to the participant's receiving the study intervention. Pregnancy tests will also be done whenever 1 menstrual cycle is missed during the active treatment period (or when potential pregnancy is otherwise suspected) and at the end of the study. Pregnancy tests may also be repeated if requested by IRBs/ECs or if required by local regulations (see Section 10.2).

m) **PSA:** For prostate cancer only, PSA to be performed every 4 weeks from C1D1 (±2 days) until EOT.

n) **Viral Disease Screening Tests**: HBsAg, , HBcAb, anti-HBs, HCVAb, and HIV to be conducted by local laboratory where required by local regulations or if warranted by participant history.

o) **Registration:** Participant enrollment number and dose level allocation assigned by Pfizer Inc.

[redacted]

090177e19bd138e0\Approved\Approved On: 14-Nov-2022 20:57 (GMT)

Table 1. Schedule of Activities: Dose Escalation (Parts 1A, 1B, 1C and 1D)

		Cycle 1 (28 days)				Cycle 2 (28 days)		Cycles 3+, (every 28 days)			
**Visit Identifier**	**Screening (≤28 days prior to Cycle 1, Day1)** [a]	**Day 1**	**Day 8**	**Day 15**	**Day 22**	**Day 1**	**Day 15**	**Day 1**	**Day 15**	**EOT** [b]	**Post-Treatment Follow-up (28 days after EOT)** [c]

p) PF-07248144 will be administered as monotherapy, QD for the first 2 dose groups and most likely for all subsequent dose groups in 28 day cycles on a continuous basis. Additional dosing frequency such as intermittent dosing may be considered after the second dose group in the study if supported by emerging clinical data. Treatment will continue until PD, unacceptable toxicity, or participant refusal, whichever occurs first.

q) [redacted]

r) **Parts 1B** [redacted] **Only.** fulvestrant to be administered in combination with PF-07248144 (Part 1B) [redacted] Detailed administration information can be found in the fulvestrant (Faslodex) USPI, [2] and country-specific label

s) [redacted]

t) **Tumor Assessments:** Tumor assessments will include all known or suspected disease sites. Imaging may include chest, abdomen, and pelvis CT or MRI scans (RECIST version 1.1). Brain scans and bone scans will be performed at baseline if disease is suspected and on study as appropriate to follow disease. Tumor assessment(s) should be repeated at the end of treatment visit if more than 6 weeks have passed since the last evaluation.

u) **Radionuclide Bone Scan (prostate cancer only):** For CRPC, whole body 99mTcmethylene diphosphonate radionuclide bone scintigraphy should be performed per the SoA. The bone scan will assess 5 regions of the skeleton, including skull, thorax, spine, pelvis, and extremities. Radiographic progression for bone disease is defined as the appearance of 1 or more metastatic lesions on bone scan. Confirmation with a second imaging modality (plain film, CT, or MRI) will be required when bone lesions are found in a single region on the bone scan. Appearance of metastatic lesions in 2 or more of the 5 regions on a bone scan will not require confirmation with a second imaging modality. PET is not an evaluable imaging modality for this study.

v) **Adverse Event Assessments:** AEs should be documented and recorded at each visit using the NCI CTCAE version 5.0. The time period for actively eliciting and collecting AEs and SAEs ("active collection period") for each participant begins from the time the participant provides informed consent through and including a minimum of 28 calendar days after the last study intervention administration. If the participant begins a new anticancer therapy, the period for recording non serious AEs on the CRF ends at the time the new treatment is started. However, any SAEs occurring during the active collection period must still be reported to Pfizer Safety and recorded on the CRF, irrespective of any intervening treatment.

Table 2. Schedule of Activities: Pharmacokinetic Sampling, Pharmacodynamic/other Biomarker Sampling, and ECG Assessments (for Parts 1A, 1B[redacted]

Visit Identifier	Screening (≤28 days prior to C1D1)	Cycle 1																						Cycle 2+		EOT	Post-Treatment Follow-up (28 days after EOT)
		Day 1										Day 8		Day 15										Day 1			
Hours Before/After Dose		pre	0	0.25 h	0.5 h	1 h	2 h	3 h	4 h	8 h	24 h	pre	0	pre	0	0.25 h	0.5 h	1 h	2 h	3 h	4 h	8 h	24 h	pre	0		
Visit Window		- 6h		±1.5m	±3 m	±6 m	±12 m	±18 m	±24 m	±2 h	-2.5 h	-30 m		-30 m		±1.5 m	±3 m	±6 m	±12 m	±18 m	±24 m	±2 h	- 2.5 h	- 30 m			
Study intervention administration**			X										X		X										X		
PF-07248144 PK blood plasma sampling (Parts 1A and 1B) [a,*]		X		X	X	X	X	X	X	X	X	X		X		X	X	X	X	X	X	X	X	X[a]		X	
[redacted]		[redacted]				[redacted]	[redacted]		[redacted]	[redacted]		[redacted]		[redacted]		[redacted]	[redacted]	[redacted]	[redacted]	[redacted]	[redacted]	[redacted]	[redacted]	[redacted]		[redacted]	

090177e19bd138e0\Approved\Approved On: 14-Nov-2022 20:57 (GMT)

Table 2. Schedule of Activities: Pharmacokinetic Sampling, Pharmacodynamic/other Biomarker Sampling, and ECG Assessments (for Parts 1A, 1B, 1C and 1D)

Visit Identifier	Screening (≤28 days prior to C1D1)	Cycle 1																						Cycle 2+		EOT	Post-Treatment Follow-up (28 days after EOT)
		Day 1										Day 8		Day 15										Day 1			
Hours Before/After Dose		pre	0	0.25 h	0.5 h	1 h	2 h	3 h	4 h	8 h	24 h	pre	0	pre	0	0.25 h	0.5 h	1 h	2 h	3 h	4 h	8 h	24 h	pre	0		
Visit Window		-6h		±1.5m	±3 m	±6 m	±12 m	±18 m	±24 m	±2 h	-2.5 h	-30 m		-30 m		±1.5 m	±3 m	±6 m	±12 m	±18 m	±24 m	±2 h	-2.5 h	-30 m			
[redacted]		[redacted]				[redacted]	[redacted]		[redacted]	[redacted]		[redacted]		[redacted]		[redacted]	[redacted]	[redacted]	[redacted]	[redacted]	[redacted]	[redacted]	[redacted]	[redacted]		[redacted]	
[redacted]		[redacted]												[redacted]										[redacted]		[redacted]	
[redacted]	[redacted]	[redacted]												[redacted]										[redacted]		[redacted]	

090177e19bd138e0\Approved\Approved On: 14-Nov-2022 20:57 (GMT)

Table 2. Schedule of Activities: Pharmacokinetic Sampling, Pharmacodynamic/other Biomarker Sampling, and ECG Assessments (for Parts 1A, 1B, [redacted]

Visit Identifier	Screening (≤28 days prior to C1D1)	Cycle 1																						Cycle 2+		EOT	Post-Treatment Follow-up (28 days after EOT)
		Day 1										Day 8		Day 15										Day 1			
Hours Before/After Dose		pre	0	0.25 h	0.5 h	1 h	2 h	3 h	4 h	8 h	24 h	pre	0	pre	0	0.25 h	0.5 h	1 h	2 h	3 h	4 h	8 h	24 h	pre	0		
Visit Window		-6h		±1.5m	±3 m	±6 m	±12 m	±18 m	±24 m	±2 h	-2.5 h	-30 m		-30 m		±1.5 m	±3 m	±6 m	±12 m	±18 m	±24 m	±2 h	-2.5 h	-30 m			
[redacted]	[redacted]	[redacted]				[redacted]			[redacted]	[redacted]	[redacted]	[redacted]		[redacted]				[redacted]			[redacted]	[redacted]	[redacted]	[redacted]		[redacted]	
[redacted]	[redacted]																										
[redacted]		[redacted]												[redacted]										[redacted]			
Mandatory archival tumor tissue [i]	X																										
[redacted]	[redacted]																			[redacted]						[redacted]	

090177e19bd138e0\Approved\Approved On: 14-Nov-2022 20:57 (GMT)

090177e19bd138e0\Approved\Approved On: 14-Nov-2022 20:57 (GMT)

Table 2. Schedule of Activities: Pharmacokinetic Sampling, Pharmacodynamic/other Biomarker Sampling, and ECG Assessments (for Parts 1A, 1B, [redacted]

Visit Identifier	Screening (≤28 days prior to C1D1)	Cycle 1																						Cycle 2+		EOT	Post-Treatment Follow-up (28 days after EOT)
		Day 1										Day 8		Day 15										Day 1			
Hours Before/After Dose		pre	0	0.25 h	0.5 h	1 h	2 h	3 h	4 h	8 h	24 h	pre	0	pre	0	0.25 h	0.5 h	1 h	2 h	3 h	4 h	8 h	24 h	pre	0		
Visit Window		-6h		±1.5m	±3 m	±6 m	±12 m	±18 m	±24 m	±2 h	-2.5 h	-30 m		-30 m		±1.5 m	±3 m	±6 m	±12 m	±18 m	±24 m	±2 h	-2.5 h	-30 m			
Pfizer Prep D1 banked blood [k]	X																										
Standard 12-lead ECG [l]	X	X				X	X					X		X				X	X					X		X	

* **NOTE:** All Japanese participants in Parts 1A, 1B, [redacted] should be hospitalized for PK sampling for at least 2 days of the first cycle of dosing of PF-07248144 [redacted]

** **NOTE:** Sample time windows apply to PF-07248144 [redacted] dosing only.

a. **Parts 1A and 1B PK Sampling:** Blood collections for PK sampling of PF-07248144 on C1D1, predose (within 6 hours prior to the morning dose of PF-07248144), 0.25, 0.5, 1, 2, 3, 4, 8, and 24 hours after the morning dose on C1D1; Cycle 1 Day 8, pre dose (within 30 minutes prior to the morning dose of PF-07248144); C1D15 predose (within 30 minutes prior to the morning dose of PF-07248144), 0.25, 0.5, 1, 2, 3, 4, 8, and 24 hours after the morning dose of PF-07248144 on C1D15; Cycles ≥2, Day 1 predose (within 30 minutes prior to the morning dose); EOT .

b. [redacted]

c. [redacted]

d. [redacted]

e. **Biomarker Assessments:** Detailed instructions for sample collection, processing, and shipment will be provided in the Laboratory Manual. All biomarker sampling should be within the protocol-specified window or the same as PK sampling.

Table 2. Schedule of Activities: Pharmacokinetic Sampling, Pharmacodynamic/other Biomarker Sampling, and ECG Assessments (for Parts 1A, 1B, [redacted]

Visit Identifier	Screening (≤28 days prior to C1D1)	Cycle 1																						Cycle 2+		EOT	Post-Treatment Follow-up (28 days after EOT)
		Day 1										Day 8		Day 15										Day 1			
Hours Before/After Dose		pre	0	0.25 h	0.5 h	1 h	2 h	3 h	4 h	8 h	24 h	pre	0	pre	0	0.25 h	0.5 h	1 h	2 h	3 h	4 h	8 h	24 h	pre	0		
Visit Window		-6h		±1.5m	±3 m	±6 m	±12 m	±18 m	±24 m	±2 h	-2.5 h	-30 m		-30 m		±1.5 m	±3 m	±6 m	±12 m	±18 m	±24 m	±2 h	-2.5 h	-30 m			

f. [redacted]

g. [redacted]

[redacted]

i. **Archival Tumor Tissue**: Archived FFPE specimen from a recurrent tumor or distant metastasis other than to bone will be required and collected for all participants. If the archived FFPE specimen is not available or sufficient, a fresh biopsy (before C1D1 treatment) will be mandatory. Additional information can be found in Section 8.8.1 and the Laboratory Manual.

j. [redacted]

k. **Pfizer Prep D1 Banked Blood**: If not collected on the designated collection day, collect at the next available time point when biospecimens are being collected in conjunction with a participant visit.

l. **Standard 12 Lead ECG:** ECGs will be collected at times specified in the Schedule of Activities. The Screening ECG will be a single 12lead ECG. At all other times, at each time point, 3 consecutive 12lead ECGs (triplicate) will be performed approximately 2 minutes apart to determine mean QTcF interval. All 12lead ECGs should be confirmed by a qualified individual at the institution. ECGs on Day 1 of each cycle ≥2 will be collected prior to dosing. For all participants on Cycle 1, Days 1 and 15: at predose, 1 hr and 2 hr postdose, Cycle 1, Day 8: predose, for Cycles ≥2, Day 1: predose, and at the EOT visit. When coinciding with blood sample draws for PK, the ECG assessment should preferably be performed prior to blood sample collection, such that the blood sample is collected at the nominal time. If the mean QTcF is prolonged (≥45 msec from the baseline or >500 msec), the ECGs should be reevaluated by a qualified individual at the institution for confirmation. Additional triplicate ECGs may be performed as clinically indicated.

090177e19bd138e0\Approved\Approved On: 14-Nov-2022 20:57 (GMT)

090177e19bd138e0\Approved\Approved On: 14-Nov-2022 20:57 (GMT)

DOSE EXPANSION (Part 2)

Table 3. Schedule of Activities: Dose Expansion (Part 2)

		Cycle 1 (28 days)		Cycle 2 (28 days)		Cycles 3+, (every 28 days)				
**Visit Identifier**	**Screening (≤28 days prior to Cycle 1, Day 1)[a]**	**Day 1**	**Day 15**	**Day 1**	**Day 15**	**Day 1**	**Day 15**	**(EOT)[b]**	**Post-Treatment Follow-up (28 days after EOT)[c]**	**Long-Term Follow-up**
**Visit Window**		±2D	±2D	±2D	±2D	±2D	±2D	±7D	+7D	
Informed consent[d]	X									
Medical/Oncological history[e]	X									
Full Physical examination[f]	X							X		
Brief physical examination[f]		X	X	X	X	X	X		X	
Height	X									
Weight	X	X		X		X		X	X	
Vital signs[g]	X	X	X	X	X	X	X	X	X	
ECOG Performance status[h]	X	X		X		X		X	X	
Contraception check[i]	X	X		X		X		X		
Concomitant medication(s)[j]	X	X	X	X	X	X	X	X	X	
**Laboratory**										
Hematology[k]	X	X	X	X	X	X	X	X	X	
Blood chemistry[k]	X	X	X	X	X	X	X	X	X	
Coagulation[k]	X	X				X (every 8 weeks)		X		
Urinalysis[k]	X							X		
Pregnancy test[l]	X	X		X		X		X		
(Standard 12-lead) ECG[m]	X	X	X	X		X		X		
Viral disease screen (Hepatitis B, C, and HIV)[n]	X									
**Registration and treatment**										
Registration[o]	X									

[redacted]

090177e19bd138e0\Approved\Approved On: 14-Nov-2022 20:57 (GMT)

Table 3. Schedule of Activities: Dose Expansion (Part 2)

		Cycle 1 (28 days)		Cycle 2 (28 days)		Cycles 3+, (every 28 days)				
**Visit Identifier**	**Screening (≤28 days prior to Cycle 1, Day 1)[a]**	**Day 1**	**Day 15**	**Day 1**	**Day 15**	**Day 1**	**Day 15**	**(EOT)[b]**	**Post-Treatment Follow-up (28 days after EOT)[c]**	**Long-Term Follow-up**
**Visit Window**		±2D	±2D	±2D	±2D	±2D	±2D	±7D	+7D	
PF-07248144 administration		Orally, QD continuously.								
[redacted]		[redacted]								
fulvestrant administration[p] (Part 2B [redacted])		Intramuscularly, 500 mg on C1D1, C1D15, C2D1 and once monthly thereafter.								
Tumor assessments: CT/MRI Scans of chest, abdomen, pelvis, any clinically indicated sites of disease; clinical evaluation of superficial disease[q]	X	Performed every 8 weeks (±7 days) from C1D1 for the first 48 weeks and then every 12 weeks (±7 days) thereafter for up to 2 years.						X		
**Other clinical assessments**										
Survival follow-up[r]									X	X
Adverse event monitoring[s]	Continuous.									
Pharmacokinetics blood sampling	See Pharmacokinetic and Biomarker Sampling table below.									
Biomarker Sampling	See Pharmacokinetic and Biomarker Sampling table below .									

a. **Screening:** To be obtained within 28 days prior to C1D1.
b. **EOT Visit:** Visit to be performed as soon as possible after the last dose of study intervention and prior to initiation of any new anti-tumor therapy. Obtain assessments if not completed in the last week on study (or within 6 weeks for tumor assessments).
c. **Post-Treatment Follow up:** At least 28 calendar days, and no more than 35 calendar days after discontinuation of study intervention, participants will return to undergo review of concomitant treatments, vital signs, and assessment for resolution of any treatment related AEs. Participants continuing to experience treatment-related toxicity after Post-Treatment Follow-up Visit will continue to be followed at least every 28 days until resolution or determination, in the clinical judgment of the investigator, that no further improvement is expected; these may be conducted via telephone.
d. **Informed Consent:** Must be obtained prior to undergoing any study specific procedures.
e. **Medical/Oncological History:** To include information on oncology disease including details of diagnosis and prior anticancer treatments (systemic treatment, prior surgery and radiotherapy, etc). When available primary diagnosis history should also include known molecular characteristics of the participant's tumor including mutations, amplifications, etc.

090177e19bd138e0\Approved\Approved On: 14-Nov-2022 20:57 (GMT)

Table 3. Schedule of Activities: Dose Expansion (Part 2)

		Cycle 1 (28 days)		Cycle 2 (28 days)		Cycles 3+, (every 28 days)				
**Visit Identifier**	**Screening (≤28 days prior to Cycle 1, Day 1)[a]**	**Day 1**	**Day 15**	**Day 1**	**Day 15**	**Day 1**	**Day 15**	**(EOT)[b]**	**Post-Treatment Follow-up (28 days after EOT)[c]**	**Long-Term Follow-up**
**Visit Window**		±2D	±2D	±2D	±2D	±2D	±2D	±7D	+7D	

f. **Physical Examination:** Full physical exam at Screening and End of Treatment, brief physical exam at all other timepoints. A symptom-directed exam and an assessment for emergent toxicities or changes from prior visits conducted by a physician, trained physician's assistant or nurse practitioner, as acceptable according to local regulation.

g. **Vital Signs:** Include oral temperature, pulse rate (PR), respiration rate, blood pressure (BP), and SpO2. BP and PR to be recorded in the sitting position or semi-recumbent position (same position should be maintained throughout the study) after approximately 5 minutes of rest.

h. **ECOG Performance Status**: ECOG performance scale is available in Section 10.12.

i. **Contraception check:** The investigator or his or her designee, in consultation with the participant, will confirm that the participant has selected an appropriate method of contraception for the individual participant [and his or her partner(s)] from the permitted list of contraception methods and will confirm that the participant has been instructed in its consistent and correct use. The investigator or designee will inform the participant of the need to use highly effective contraception consistently and correctly and document the conversation and the participant's affirmation in the participant's chart (participants need to affirm their consistent and correct use of at least 1 of the selected methods of contraception). In addition, the investigator or designee will instruct the participant to call immediately if the selected contraception method is discontinued or if pregnancy is known or suspected in the participant or partner.

j. **Concomitant Treatments:** All concomitant medications and nondrug supportive interventions should be recorded on the CRF.

k. **Hematology, Blood Chemistry, Coagulation, and Urinalysis:** No need to repeat on C1D1 if baseline assessment performed within 7 days prior to that date (see Section 10.2 for a complete list of clinical laboratory tests).

l. **Pregnancy Test:** Pregnancy tests may be urine or serum tests, but must have a sensitivity of at least 25 mIU/mL. Pregnancy tests will be performed in WOCBP. Following a negative pregnancy test result at screening, appropriate contraception must be commenced and a second negative pregnancy test result will be required at the baseline visit prior to the participant's receiving the study treatment. Pregnancy tests will also be done whenever 1 menstrual cycle is missed during the active treatment period (or when potential pregnancy is otherwise suspected) and at the end of the study. Pregnancy tests may also be repeated if requested by IRBs/ECs or if required by local regulations.

m. **Standard 12 Lead ECG**: ECGs will be collected at times specified in the SoA. The Screening ECG will be a single 12-lead ECG. At all other times, at each time point, 3 consecutive 12 lead ECGs (triplicate) will be performed approximately 2 minutes apart to determine mean QTcF interval. ECG on Days 1 and 15 of cycle 1 will be collected prior to dosing. ECGs on Day 1 of each cycle ≥2 will be collected prior to dosing, and at the EOT visit. All 12 lead ECGs should be confirmed by a qualified individual at the institution. If the mean QTcF is prolonged (≥45 msec from the baseline or >500 msec), the ECGs should be reevaluated by a qualified individual at the institution for confirmation. Additional triplicate ECGs may be performed as clinically indicated. For the food effect substudy, additional ECGs should be collected at Cycle 1 Day -7: at predose, 1 hr and 2 hr postdose. After predose, a window of ±10% time is allowable.

n. **Viral Disease Screening Tests:** HbsAg, HBcAb, anti-HBs, HCVAb, and HIV to be conducted by local laboratory where required by local regulations or if warranted by participant history.

o. **Registration:** Participant enrollment number and dose level allocation assigned by Pfizer Inc.

p. **For Part 2B** [redacted]**:** fulvestrant to be administered in combination with PF-07248144 (Part 2B) [redacted] Detailed administration information can be found in the fulvestrant (Faslodex) USPI,[2] and country-specific label.

090177e19bd138e0\Approved\Approved On: 14-Nov-2022 20:57 (GMT)

Table 3. Schedule of Activities: Dose Expansion (Part 2)

		Cycle 1 (28 days)		Cycle 2 (28 days)		Cycles 3+, (every 28 days)				
**Visit Identifier**	**Screening (≤28 days prior to Cycle 1, Day 1)[a]**	**Day 1**	**Day 15**	**Day 1**	**Day 15**	**Day 1**	**Day 15**	**(EOT)[b]**	**Post-Treatment Follow-up (28 days after EOT)[c]**	**Long-Term Follow-up**
**Visit Window**		**±2D**	**±2D**	**±2D**	**±2D**	**±2D**	**±2D**	**±7D**	**+7D**	

q. **Tumor Assessments:** Tumor assessments will include all known or suspected disease sites. Imaging may include chest, abdomen, and pelvis computed tomography or MRI scans (RECIST version 1.1). Brain scans and bone scans will be performed at baseline if disease is suspected and on study as appropriate to follow disease. Tumor assessment should be repeated at the EOT visit if more than 6 weeks have passed since the last evaluation.

r. **Survival Follow up:** Participants will be contacted by telephone approximately every 3 months for survival data collection until end of trial (2 years from last participant first dose), unless otherwise notified by the Sponsor.

s. **Adverse Event Assessments:** AEs should be documented and recorded at each visit using the NCI CTCAE version 5.0. The time period for actively eliciting and collecting AEs and SAEs ("active collection period") for each participant begins from the time the participant provides informed consent through and including a minimum of 28 calendar days after the last study intervention administration. If the participant begins a new anticancer therapy, the period for recording non serious AEs on the CRF ends at the time the new treatment is started. However, any SAEs occurring during the active collection period must still be reported to Pfizer Safety and recorded on the CRF, irrespective of any intervening treatment.

[redacted]

Table 4. Schedule of Activities: Pharmacokinetic Sampling, Pharmacodynamic/other Biomarker Sampling (for Parts 2A [for participants not participating in food effect substudy], 2B [redacted]

Visit Identifier	Screening (≤28 days prior to C1D1)	Cycle 1 Day 1		Cycle 1 Day 15			Cycle 2+ Day 1		EOT	Post-Treatment Follow-up (28 days after EOT)
**Hours Before/After Dose**		**pre**	**0**	**pre**	**0**	**4h**	**pre**	**0**		
**Visit Window**		**-6h**		**-30m**		**±24m**	**-30 m**			
Study intervention administration**			X		X			X		
PF-07248144 PK blood plasma sampling (for participants not participating in the food effect substudy) [a]		X		X		X	X (Cycles 2 - 4 only)		X	
[redacted]		[redacted]		[redacted]		[redacted]	[redacted]		[redacted]	
PF-07248144 urine collection for PK and metabolite profiling (subset of participants in Part 2A, same participants as the blood sample for metabolite profiling subset) [b]	X			See footnote b						
Blood Sample for metabolite profiling (subset of participants in Part 2A, same participants as the PF-07248144 urine collection for PK and metabolite profiling subset) [c]	X			See footnote c						
[redacted]	[redacted]	[redacted]		[redacted]			[redacted]		[redacted]	
[redacted]	[redacted]	[redacted]		[redacted]		[redacted]	[redacted]			
[redacted]	[redacted]									
[redacted]		[redacted]		[redacted]			[redacted]			
Mandatory archival tumor tissue [i]	X									
[redacted]	[redacted]					[redacted]			[redacted]	
Pfizer Prep D1 banked blood [k]	X									

090177e19bd138e0\Approved\Approved On: 14-Nov-2022 20:57 (GMT)

090177e19bd138e0\Approved\Approved On: 14-Nov-2022 20:57 (GMT)

Table 4. Schedule of Activities: Pharmacokinetic Sampling, Pharmacodynamic/other Biomarker Sampling (for Parts 2A [for participants not participating in food effect substudy], 2B [redacted])

Visit Identifier	Screening (≤28 days prior to C1D1)	Cycle 1					Cycle 2+		EOT	Post-Treatment Follow-up (28 days after EOT)
		Day 1		Day 15			Day 1			
**Hours Before/After Dose**		**pre**	**0**	**pre**	**0**	**4h**	**pre**	**0**		
**Visit Window**		**-6h**		**-30m**		**±24m**	**-30 m**			

**** NOTE:** Sample time windows apply to PF-07248144 [redacted] dosing only.

a. **Part 2 (Non-food effect) PK Sampling:** Blood collections for PK sampling at predose time point (within 6 hours prior to the morning dose of PF-07248144) on Cycle 1 Day 1, predose on Cycle 1 Day 15, and for Cycles ≥2 Day 1 predose (within 30 minutes prior to the morning dose), 4 hours postdose on C1D15 (± 24 minutes); EOT and Post-Treatment Follow-Up. This PK sampling is in Part 2A, 2B [redacted] participants except for those participating in food effect subset.

b. **Urine Collection for PF-07248144 PK and Metabolite Profiling:** Urine will be collected at screening and for 24 hours after PF-07248144 dosing for PK and metabolite ID on Cycle 1 Day 15 over the following intervals: 0 to 4 hrs, 4 to 8 hrs, 8 to 12 (-3 hr window), and 12 to 24 hrs postdose. These urine samples for PK and metabolite profiling will be collected from at least 6 participants in Part 2A. Urine collection for PF-07248144 PK and metabolite profiling will be collected from the same subset of participants from whom blood samples for metabolite profiling are collected in Part 2A.

c. **Blood Samples for PF-07248144 Metabolite Profiling**: Blood samples will be collected for metabolite profiling at screening (or prior to first dose administration) and on Cycle 1 Day15 predose (within 30 minutes prior to the morning dose of PF-07248144), 0.25, 0.5, 1, 2, 3, 4, 8, and 24 hours after the morning dose of PF-07248144 on C1D15). These blood samples for metabolite profiling will be collected from at least 6 participants in Part 2A. Blood samples for metabolite profiling will be collected from the same subset of participants from whom urine samples are collected in Part 2A.

d. [redacted]

e. **Biomarker Assessments:** Detailed instructions for sample collection, processing, and shipment will be provided in the Laboratory Manual. All biomarker sampling should be within the protocol-specified window or the same as PK sampling.

f. [redacted]

g. [redacted]

[redacted]

i. **Archival Tumor Tissue**: Archived FFPE specimen from a recurrent tumor or distant metastasis other than to bone will be required and collected for all participants. If the archived FFPE specimen is not available or sufficient, a fresh biopsy (before C1D1 treatment) will be mandatory. Additional information can be found in Section 8.8.1 and the Laboratory Manual.

j. [redacted]

k. **Pfizer Prep D1 Banked Blood**: If not collected on the designated collection day, collect at the next available time point when biospecimens are being collected in conjunction with a participant visit.

090177e19bd138e0\Approved\Approved On: 14-Nov-2022 20:57 (GMT)

Table 5. Schedule of Activities: Pharmacokinetic Sampling Schedule in Participants in the Food Effect Subset of Part 2A (Monotherapy Dose Expansion)

**Visit Identifier**	**Screening (≤28 Days)**	**Cycle 1 Only**				**Cycle 2 and Beyond**	**End of Treatment**
**Study Day**		**Day -7**	**Day 1**	**Day 15**		**Day 1**	
**Hours Before/After Dose**				**predose**	**4hr**	**predose**	
**Visit Window**				**-30 m**	**±24 m**	**-30 m**	
Blood samples for PF-07248144 PK[a]		X	X	X	X	X	X
Meal Administration[b]		X					

a. **PK Sampling:** The food effect assessment will take place in a subset (approximately 6) of participants enrolled in Part 2A (expansion monotherapy). Each participant will serve as his/her own control in which PF-07248144 will be administered in the morning under "fed conditions" on Cycle 1 Day -7 and- under "fasted" conditions on Cycle 1 Day 1 (see Section 6.1.1.6). Blood collections for PK sampling of PF-07248144 will occur at predose (within 6 hours prior to the morning dose of PF-07248144); 0.25, 0.5, 1, 2, 3, 4, 8, and 24 hours after the morning dose- on Cycle 1 Day -7 and Cycle 1 Day 1; and at predose (within 30 minutes prior to the morning dose) and 4 hours post dose on Cycle 1 Day 15; and predose (within 30 minutes prior to the morning dose) Cycles ≥2 Day 1 and at the EOT visit. The visit windows for PK samples collected on Cycle 1 Day -7 and Cycle 1 Day 1 will be the same as the visit windows used in Part 1A Cycle 1 Day 1 PK sampling visit windows.

b. **Meal Administration:** For all participants in the food effect subset, on Cycle 1 Day -7, a test breakfast meal will be provided and must be consumed over 30 minutes. PF-07248144 will be administered with approximately 8 oz (240 mL) of water 30 minutes after the start of the meal. No additional food will be allowed until at least 4 hours post-dose. The test meal to be consumed will be a high fat (approximately 50% of total caloric content of the meal) and high calorie (approximately 800-1000 calories) meal. This test meal should derive approximately 150, 250, and 500-600 calories from protein, carbohydrate, and fat, respectively.

[redacted]

2. INTRODUCTION

PF-07248144 is an orally available small-molecule inhibitor of human KAT6 histone acetyltransferases, KAT6A and KAT6B that is being investigated in participants with locally advanced or metastatic ER+HER2- breast cancer, CRPC, or NSCLC whose disease progressed on or who are intolerant to standard therapy.

2.1. Study Rationale

PF-07248144 has demonstrated potent cellular activity in ER+ breast cancer, CRPC and NSCLC preclinical models and tolerable safety profile in nonclinical toxicology studies, which justify further clinical development.

The purpose of this FIH clinical study is to evaluate the safety, tolerability, and potential clinical benefits of PF-07248144 as monotherapy and combination in participants with locally advanced or metastatic ER+HER2- breast cancer, CRPC, or NSCLC whose disease progressed on or who are intolerant to standard therapy.

2.2. Background

PF-07248144 is a potent and selective catalytic inhibitor of KAT6 histone acetyltransferases, KAT6A and KAT6B as evidenced by its potent biochemical inhibition of both enzymes. Histone acetylation is a reversible protein modification essential for chromatin organization and function [3]. Acetylation of histones is catalyzed by KATs or other HATs using AcCoA as the co-factor[4,5]. KAT6A (also known as MOZ or MYST3) and KAT6B (also known as MORF) are part of MYST family of HATs composed of 5 HATs: KAT5, KAT6A, KAT6B, KAT7, KAT8[6]. KAT6A and KAT6B are paralog genes, which are responsible for acetylation of H3K23, and their acetyltransferase functions are involved in fundamental cellular processes, including gene transcription, cellular senescence, tissue development, and maintenance of normal hematopoietic stem cells[7-9]. KAT6A was first identified as part of a chromosomal translocation t(8;16)(p11;p13) with CREBBP in AML[10,-11]. Subsequently, additional KAT6A and KAT6B translocations, generating fusions with other HATs such as EP300, NCOA2, and NCOA3 have also been identified in AML[7, 10]. Experimentally, KAT6A enzymatic activity is critical for the oncogenic function of KAT6A fusion proteins in leukemogenesis[12].

KAT6A was also identified as part of the recurrently amplified 8p11-12 region found in 10 to 15% of breast cancers[13] and shown to be a significant dependency in 8p11 amplified breast cancer cell lines overexpressing KAT6A, thus highlighting its oncogenic function[14, 15]. KAT6A shRNA-mediated knockdown reduced ESR1 mRNA and ERα protein levels in ER+ breast cancer cells. KAT6A is a direct transcriptional regulator of ESR1 and is localized to the gene promoter directly regulating its transcription. Overexpression of ESR1 partially rescued the growth defect caused by shRNA depletion of KAT6A[14]. These findings indicated an important role of KAT6A in gene regulation of ESR1 required for growth of ER+ breast cancer cells. Chromosome 8p11-12 amplifications and KAT6A over-expression were also observed in additional tumor types including prostate cancer, ovarian cancer, uterine cervix cancer, lung adenocarcinoma, colon and rectal adenocarcinomas, and medulloblastoma.[16-18]

090177e19bd138e0\Approved\Approved On: 14-Nov-2022 20:57 (GMT)

These results support the treatment potential of KAT6A and KAT6B inhibitors in multiple disease settings, including breast, prostate and non-small cell lung cancer.

2.2.1. Nonclinical Pharmacology

PF-07248144 is a potent and selective catalytic inhibitor of KAT6 histone acetyltransferases, KAT6A and KAT6B. [redacted]

Overall, the above background and preclinical data provide the rationale to investigate KAT6A/6B as therapeutic targets and to evaluate the safety and potential clinical benefits of PF-07248144 as monotherapy or in combination in participants with ER+HER2- breast cancer, CRPC, and NSCLC by inhibiting KAT6A/6B enzymes.

[redacted]

[redacted]

090177e19bd138e0\Approved\Approved On: 14-Nov-2022 20:57 (GMT)

[redacted]

090177e19bd138e0\Approved\Approved On: 14-Nov-2022 20:57 (GMT)

[redacted]

[redacted]

[redacted]

2.2.4. Clinical Overview for Study C4551001

The purpose of this ongoing Phase 1 clinical study C4551001 is to evaluate the safety, tolerability, and potential clinical activity of PF-07248144 as monotherapy or combination in participants with locally advanced or metastatic ER+HER2- breast cancer, CRPC, or NSCLC whose disease progressed on or who are intolerant to standard therapy.

As of 12 September 2022, a total of 66 participants have been treated across the study. 21 participants were treated in monotherapy dose escalation cohorts, including 9 participants with ER+ HER2- breast cancer, 10 with CRPC, and 2 with NSCLC.

In monotherapy dose escalation part 1A, 6 participants were treated with 15 mg QD, 7 participants with 8 mg QD, 4 participants with 5 mg QD, and 4 participants with 2 mg QD respectively. In fulvestrant combination dose escalation Part 1B, 4 participants with ER+ HER2- breast cancer were treated with 5 mg QD. For monotherapy dose expansion Part 2A, 33 participants with ER+ HER2- breast cancer were treated with 5 mg QD. For fulvestrant combination dose expansion Part 2B, 8 participants with ER+ HER2- breast cancer were treated with 5 mg QD.

3 DLTs were reported during the study. Of these, 1 DLT was reported at 2 mg QD (monotherapy escalation), 1 DLT was reported at 8 mg QD (monotherapy escalation), and 1 DLT was reported at 5 mg QD + fulvestrant (combination escalation). All 3 DLTs were Grade 3 neutropenia (decreased neutrophil count) and resolved with dose modification.

Among 59 participants (evaluable for safety analysis) treated with PF-07248144 in this study (escalation and expansion), 52 (88.1%) experienced at least 1 treatment-related AE.

The most common treatment-related AEs occurring in $\geq$ 20% participants were: dysgeusia in (N= 41; 69.5%), neutrophil count decreased/neutropenia in (N= 33; 55.9%), anemia (N= 24; 40.7%), white blood cell count decreased (N= 20; 33.9%), platelet count decreased/thrombocytopenia (N= 16; 27.1%), diarrhea (N= 13; 22%), and fatigue (N= 12; 20.3%)

090177e19bd138e0\Approved\Approved On: 14-Nov-2022 20:57 (GMT)

Grade ≥3 treatment-related AEs in more than one participant included neutrophil count decreased/neutropenia (N=17; 28.8%), anemia (N=6; 10.2%), and white blood cell count decreased (N= 4; 6.8%).

[redacted]

[redacted]

[redacted]

As of 12 September 2022, encouraging early signs of clinical activity in ER+ HER2- breast cancer participants were observed in monotherapy dose escalation (Part 1A), monotherapy dose expansion (Part 2A) as well as in combination dose escalation with fulvestrant (Part 1B). Of these early signs of clinical activity, several PRs were confirmed based on the investigators' assessment.

RDE for monotherapy was identified as 5 mg QD based on the overall assessment of clinical safety and PK/PD. RDE for combination with fulvestrant was also identified as 5 mg QD.

Please refer to the PF-07248144 IB for further information.

2.2.4.1. Overview of Clinical Pharmacology for PF-07248144

Following QD oral administration alone (at 2, 5, 8, and 15 mg) or 5 mg in combination with fulvestrant, PF-07248144 was rapidly absorbed, with a median Tmax of 3 hours based on preliminary data. Across the dose range of 2 to 15 mg, PF-07248144 steady-state AUC during the 24-hr dosing interval (AUC_{24} on Cycle 1 Day 15) and C_{max} increased with dose in an approximately dose-proportional manner. [redacted]

090177e19bd138e0\Approved\Approved On: 14-Nov-2022 20:57 (GMT)

Please refer to the PF-07248144 IB for further information.

[redacted]

2.2.6. Overview of Fulvestrant

Fulvestrant is an ER antagonist indicated for the treatment of: 1) HR+HER2- advanced breast cancer in postmenopausal women not previously treated with endocrine therapy 2) HR+ advanced breast cancer in postmenopausal women with disease progression following endocrine therapy and 3) HR+HER2- advanced or metastatic breast cancer in combination with palbociclib in women with disease progression after endocrine therapy.

[redacted]

[redacted]

[redacted]

[redacted]

090177e19bd138e0\Approved\Approved On: 14-Nov-2022 20:57 (GMT)

[redacted]

[redacted]

[redacted]

[redacted]

[redacted]

[redacted]

[redacted]

[redacted]

090177e19bd138e0\Approved\Approved On: 14-Nov-2022 20:57 (GMT)

[redacted]

2.3. Benefit/Risk Assessment

C4551001 is an ongoing FIH Phase 1 clinical study for PF-07248144. A safety summary as of the data cutoff of 12 September 2022 is provided in Section 2.2.4. Risk and mitigation strategy is summarized in Section 2.3.1. More detailed information about the known and expected benefits and risks, and reasonably expected AEs of PF-07248144 [redacted] may be found in their IBs, which are the SRSDs [redacted] in this study. Detailed information about the known and expected benefits and risks for fulvestrant may be found in the fulvestrant (Faslodex) USPI,[2] which will be the SRSD for this study.

[redacted]

[redacted]

[redacted]

090177e19bd138e0\Approved\Approved On: 14-Nov-2022 20:57 (GMT)

2.3.1. Risk Assessment

Potential Risk of Clinical Significance	Summary of Data/Rationale for Risk	Mitigation Strategy
**Study Intervention(s)/Procedures**		
**PF-07248144**		
[redacted]	The potential key risks are based on AEs reported in studies with PF-07248144 in rats and dogs.	Frequent clinical laboratory assessments including hematology (complete blood counts and differentials), coagulation, and clinical chemistry (Section 10.2).<br><br>AEs will be monitored on an ongoing basis.<br><br>PF-07248144 doses may be interrupted or reduced based on toxicities observed (See Table 10 and Table 11).<br><br>Effective contraception methods will be used in all participants (see Section 10.4).
[redacted]		
[redacted]	[redacted]	[redacted]

[redacted]

090177e19bd138e0\Approved\Approved On: 14-Nov-2022 20:57 (GMT)

Potential Risk of Clinical Significance	Summary of Data/Rationale for Risk	Mitigation Strategy
	**Study Intervention(s)/Procedures**	
		• [redacted]
	**Fulvestrant**	
Potential risks associated with fulvestrant include:<br>• Increased exposure in participants with hepatic impairment.<br>• Injection site reactions, including risk of bleeding.<br>• Embryo-fetal toxicity.	The potential risks are based on the USPI.	AEs and clinical laboratory results will be monitored on an ongoing basis.<br>• LFT abnormality and hepatic impairment: Clinical signs of hepatic impairment, such as fatigue and jaundice and laboratory results will be monitored.<br>• Injection site reactions: Careful monitoring at the time of injection will be conducted.<br>• Embryo-Fetal Toxicity: Effective contraception methods to be taken in female and male participants.
	[redacted]	
[redacted]	[redacted]	[redacted]

090177e19bd138e0\Approved\Approved On: 14-Nov-2022 20:57 (GMT)

Potential Risk of Clinical Significance	Summary of Data/Rationale for Risk	Mitigation Strategy
	**Study Intervention(s)/Procedures**	
		• [redacted]
	[redacted]	
[redacted]	[redacted]	[redacted]
[redacted]<br>[redacted]<br>[redacted]		[redacted]
[redacted]		[redacted]
[redacted]		[redacted]
[redacted]		[redacted]

090177e19bd138e0\Approved\Approved On: 14-Nov-2022 20:57 (GMT)

Potential Risk of Clinical Significance	Summary of Data/Rationale for Risk	Mitigation Strategy
	**Study Intervention(s)/Procedures**	
• metabolism and nutrition disorders (decreased [redacted]		[redacted]
[redacted]		[redacted]
[redacted]		[redacted]
	**Study Procedures**	
[redacted] for at least 5 participants enrolled in Part 2A, 5 in Part 2B, [redacted]	[redacted] threatening or fatal in rare cases.	[redacted]

090177e19bd138e0\Approved\Approved On: 14-Nov-2022 20:57 (GMT)

2.3.2. Benefit Assessment

This is a FIH study to evaluate PF-07248144. The study intervention should be used with appropriate caution typical for an investigational drug. Participant benefits include contributing to the process of developing new therapies in oncology areas of unmet need, receiving study intervention that may have clinical utility, and receiving medical evaluations and assessments associated with the study procedures.

2.3.3. Overall Benefit/Risk Conclusion

[redacted] n addition, PF-07248144 has displayed a tolerable safety profile and encouraging early signs of clinical activity (eg, PRs) as a monotherapy and in combination with fulvestrant during Phase 1 dose escalation.

Considering the measures taken to minimize risk to participants in this study, the potential risks identified in association with PF-07248144 are exceeded by the anticipated benefits that may be afforded to participants with locally advanced or metastatic ER+HER2- breast cancer, CRPC, and NSCLC.

090177e19bd138e0\Approved\Approved On: 14-Nov-2022 20:57 (GMT)

3. OBJECTIVES AND ENDPOINTS

Part 1: PF-07248144 Monotherapy (Part 1A) and Combination Dose Escalation (Parts 1B, [redacted]	
**Primary Objectives:**	**Primary Endpoints:**
**Part 1A: Monotherapy Dose Escalation**<br>• To assess safety and tolerability of escalating dose levels of PF-07248144 in successive cohorts of participants with locally advanced or metastatic ER+ HER2 breast cancer, CRPC, or NSCLC to determine the monotherapy MTD and to select the monotherapy RDE.<br>**Part 1B: Combination Dose Escalation**<br>• To assess safety and tolerability of PF-07248144 in combination with fulvestrant in participants with locally advanced or metastatic - ER+HER2 breast cancer who have progressed after at least 1 prior line of treatment with an endocrine therapy and CDK4/6 inhibitor to determine the combination MTD and to select the combination RDE.<br>[redacted]	**Parts 1A, 1B,** [redacted]<br>• DLTs.<br>• AEs as characterized by type, frequency, severity (as graded by NCI CTCAE version 5.0), timing, seriousness, and relationship to study therapy.<br>• Laboratory abnormalities as characterized by type, frequency, severity (as graded by NCI CTCAE version 5.0), and timing.

090177e19bd138e0\Approved\Approved On: 14-Nov-2022 20:57 (GMT)

**Part 1: PF-07248144 Monotherapy (Part 1A) and Combination Dose Escalation (Parts 1B, [redacted])**	
**Secondary Objective:**	**Secondary Endpoint(s):**
• To evaluate the single- and multiple-dose PK of PF-07248144 when given as monotherapy (Part 1A), in combination with fulvestrant (Part 1B); [redacted]<br><br>[redacted]<br><br>[redacted]	• PK parameters of PF-07248144:<br><br>• Single Dose: C_{max}, T_{max}, AUC_{last}, and as data permit, AUC_{inf}, CL/F, V_z/F, and $t_{1/2}$.<br><br>• Multiple Dose: $C_{max,ss}$, $T_{max,ss}$, $AUC_{\tau,ss}$, $C_{min,ss}$, CL_{ss}/F, and as data permit, V_{ss}/F, $t_{1/2}$, and R_{ac} ($AUC_{\tau,ss}/AUC_{\tau,sd}$).<br><br>• [redacted]<br><br>[redacted]
**Exploratory Objectives:**	**Exploratory Endpoints:**
• To evaluate the anti-tumor activity of PF-07248144 as monotherapy<br><br>• To evaluate the anti-tumor activity of PF-07248144 in combination with: fulvestrant (Part 1B); [redacted]	• BOR as assessed by investigator based on RECIST v1.1.<br><br>• DOR and CBR as assessed by investigator based on RECIST v1.1.<br><br>• PFS as assessed by investigator based on RECIST v1.1.
[redacted]	[redacted]
• To evaluate PD effects following PF-07248144 treatment as a single agent (Part 1A) and in combination with fulvestrant (Part 1B), [redacted]	• Changes from [redacted] in [redacted]

090177e19bd138e0\Approved\Approved On: 14-Nov-2022 20:57 (GMT)

**Part 1: PF-07248144 Monotherapy (Part 1A) and Combination Dose Escalation (Parts 1B, 1[redacted])**	
• To explore potential predictive biomarkers of clinical response, mechanisms of action, and mechanisms of resistance to PF-07248144 as a single agent (Part 1A) and in combination with fulvestrant (Part 1B), [redacted]	• [redacted].<br>• [redacted] analyses of [redacted]<br>• Changes in [redacted] that may be related to response or resistance to treatment.

090177e19bd138e0\Approved\Approved On: 14-Nov-2022 20:57 (GMT)

Part 2: PF-07248144 Monotherapy (Part 2A) and Combinations in Dose Expansion (Parts 2B [redacted]	
**Primary Objectives:**	**Primary Endpoints:**
**Part 2A: ER+HER2- breast cancer 2L+, monotherapy**<br><br>• To assess safety and tolerability of PF-07248144 monotherapy at the RDE from Part 1A in participants with advanced or metastatic 2L+ ER+HER2- breast cancer who have progressed after at least 1 prior line of CDK4/6 inhibitor and 1 line of endocrine therapy.<br><br>**Part 2B: 2-4L ER+HER2- breast cancer, combination**<br><br>• To assess safety and tolerability of PF-07248144 at the RDE in combination with fulvestrant (determined in Part 1B) in participants with advanced or metastatic ER+HER2- breast cancer whose disease has progressed after 1 line of a CDK4/6 inhibitor and at least 1 line of endocrine therapy and who must not have received more than 3 lines of systemic therapies including up to 1 line of cytotoxic chemotherapy for visceral disease in advanced or metastatic setting.<br><br>[redacted]<br><br>[redacted]	• AEs as characterized by type, frequency, severity (as graded by NCI CTCAE version 5.0), timing, seriousness, and relationship to study therapy.<br><br>• Laboratory abnormalities as characterized by type, frequency, severity (as graded by NCI CTCAE version 5.0), and timing.

090177e19bd138e0\Approved\Approved On: 14-Nov-2022 20:57 (GMT)

Part 2: PF-07248144 Monotherapy (Part 2A) and Combinations in Dose Expansion (Parts 2B [redacted]	
**Secondary Objectives:**	**Secondary Endpoints:**
• To evaluate antitumor activity of PF-07248144 monotherapy and in combination with fulvestrant (Part 2B) [redacted]	• BOR, DOR, and CBR as assessed by investigator based on RECIST v1.1.<br>• PFS as assessed by investigator based on RECIST v1.1.<br>• TTP as assessed by investigator based on RECIST v1.1.<br>• Overall Survival.
• To evaluate PK of PF-07248144 monotherapy (at RDE from Part 1A) and in combination with fulvestrant (at combination RDE from Part 1B) [redacted]	• Monotherapy and combination cohorts: Trough concentrations of PF-07248144 for selected cycles.
• [redacted]	[redacted]
• To evaluate the effect of food on the PK of PF-07248144 administered (at Part 1A RDE) in a subset of participants in Part 2A (approximately 6 participants).<br>• To evaluate urine PK of PF-07248144 in a subset of participants in Part 2A (at least 6 participants).	• In a subset, participants (approximately 6) in Part 2A, PK parameters (C_{max}, T_{max}, AUC_{last}, and as data permit, AUC_{inf}, CL/F, V_z/F, and $t_{1/2}$) of PF-07248144 given with and without food.<br>• The amount of PF-07248144 excreted in urine relative to dose administered (%) and renal clearance (CL_r).

090177e19bd138e0\Approved\Approved On: 14-Nov-2022 20:57 (GMT)

Part 2: PF-07248144 Monotherapy (Part 2A) and Combinations in Dose Expansion (Parts 2B [redacted])	
**Exploratory Objectives:**	**Exploratory Endpoints:**
• To evaluate PD effects of KAT6A/6B inhibition following PF-07248144 treatment as a single agent (Part 2A) and in combination with fulvestrant (2B) [redacted].<br>• To explore potential predictive biomarkers of clinical response, mechanisms of action, and mechanisms of resistance as a single agent (Part 2A) and in combination with fulvestrant (2B) [redacted]).<br>• To evaluate the metabolic profiling of PF-07248144 in a subset of participants in Part 2A (approximately 6 participants).	• Changes from [redacted], [redacted] in [redacted]<br>• [redacted].<br>• [redacted] analyses of [redacted] and their relationship to clinical response.<br>• [redacted] that may be related to response or resistance to treatment.<br>• [redacted]

4. STUDY DESIGN

4.1. Overall Design

This is an open-label, multi-center Phase 1 study in adult participants to evaluate safety, tolerability, PK, and PD of PF-07248144 in locally advanced or metastatic selected solid tumors (ER+HER2- breast cancer, CRPC, or NSCLC) and early signs of clinical activity of PF-07248144 as a single agent and in combination in ER+HER2- breast cancer. The overall study design is depicted in the schema (Section 1.2).

Part 1 dose escalation consists of: Part 1A, Part 1B[redacted]

Part 1A contains dose escalation as monotherapy in participants with locally advanced or metastatic ER+HER2- breast cancer, CRPC, or NSCLC that are resistant or intolerant to standard therapy or for whom no standard therapy is available, to determine the MTD and select the RDE. Participants will receive escalating doses of PF-07248144. BLRM guided by EWOC principle will be used to guide dose escalation process and determine the MTD. [redacted] Maximum allowable PF-07248144 dose increment is 100% unless in circumstance defined in Section 4.3.3. DLT will be assessed during Cycle 1 (the first 28 days with the inclusion of C2D1 laboratory assessments). Each dose level group will be approximately 3 participants, with at least 1 DLT-evaluable participant per cohort in the first 2 cohorts and at least 2 DLT evaluable participants per dose level group in the remaining cohorts for Part 1A. Per BLRM design, expanding additional

090177e19bd138e0\Approved\Approved On: 14-Nov-2022 20:57 (GMT)

participants at lower dose levels is allowed to assess safety. Additional dosing frequency such as intermittent dosing may be considered if supported by emerging clinical data.

In Part 1B, PF-07248144 in combination with fulvestrant, will be evaluated for dose finding in participants with locally advanced or metastatic ER+HER2- breast cancer (2L+) who have progressed after at least 1 line of treatment with an endocrine therapy and CDK4/6 inhibitor to determine the MTD and RDE for this combination. The definitions of RDE and RP2D are provided in Section 4.3.7. Combination RDE may be different from monotherapy RDE due to potential toxicity overlap or drug-drug interaction.

[redacted]

[redacted]

[redacted] Treatment will continue until PD, unacceptable toxicity, or participant refusal, whichever occurs first.

BLRM specifically developed for double and triple combinations will be used for dose finding in Part 1B, [redacted] PF-07248144 may start at 1 dose level below the monotherapy RDE (RDE-1) with fixed doses of fulvestrant [redacted] The definitions of MTD and RDE are provided in Section 4.3.6 and 4.3.7. In addition, depending on the safety findings in Part 1A, and whether significant overlapping toxicities are expected in combination, the starting dose of PF-07248144 in combination can be further modified to a lower dose. The sponsor may choose to advance a lower dose to start the combination dose finding Part 1B, [redacted] prior to the monotherapy MTD being reached. This decision will be made based on emerging preliminary safety, PK, PD, and/or activity data during Part 1A. At least 2 DLT evaluable participants will be required for dose level groups in Part 1B, [redacted]

After the determination of the monotherapy expansion RDE in Part 1A, PF-07248144 will be evaluated in a dose expansion cohort as a monotherapy in locally advanced or metastatic ER+HER2- breast cancer (2L+) who have progressed after at least 1 prior line of CDK4/6 inhibitor and 1 line of endocrine therapy (Part 2A). The definitions of RDE and RP2D are provided in Section 4.3.7. [redacted]

A food effect assessment will take place in a subset of participants (approximately 6) in Part 2A. Each participant will serve as his/her own control in which PF-07248144 will be

090177e19bd138e0\Approved\Approved On: 14-Nov-2022 20:57 (GMT)

administered in the morning under "fed conditions" on Cycle 1 Day -7 and under "fasted" conditions on Cycle 1 Day 1. Additional details are provided in Section 4.3.8.

PK and metabolite profiling in both urine and blood will be assessed in a subset of participants (approximately 6) in Part 2A. Both urine and blood samples should be collected from the same participants at screening and on Cycle 1 Day 15 (Sections 8.5.2 and 8.5.3).

After determination of the combination RDE from Part 1B, PF-07248144 in combination with fulvestrant will be evaluated in a dose-expansion combination cohort in participants with advanced or metastatic 2-4L ER+HER2- breast cancer whose disease has progressed after at least 1 prior line of a CDK4/6 inhibitor and at least 1 prior line of endocrine therapy and who must not have received more than 3 lines of systemic therapies in advanced or metastatic setting (Part 2B). Paired [redacted]

[redacted]

Furthermore, to better characterize safety, PK and potential efficacy, each dose expansion may investigate more than one dose level(s) of the study intervention(s) in up to an additional 30 participants. The dose levels to be evaluated will be informed by available data from dose escalation (including safety, PK/PD, and activity) and will not exceed the monotherapy MTD unless the observed exposure in combination significantly lower than monotherapy.

[redacted] the MTD and/or RDE based on emerging and available preliminary clinical data, including safety/tolerability, laboratory, PK, and PD findings. [redacted] from the MTD and/or RDE of the QD/BID regimen and satisfying EWOC criteria. PF-07248144 [redacted], if indicated based on emerging clinical data.

[redacted]

Number of Participants

The total number of participants is estimated to be approximately 140 to 200

090177e19bd138e0\Approved\Approved On: 14-Nov-2022 20:57 (GMT)

Part 1 Dose Escalation: Approximately 70 participants will be enrolled in Part 1 including 25 to 30 participants in Part 1A, 6 to 9 participants in Part 1B, [redacted]

The actual number of participants enrolled will depend on the tolerability of PF-07248144 and the number of dose levels required to identify the MTD/RDE as monotherapy or in combination.

Part 2: Dose Expansion: Approximately 90-120 participants are expected to be enrolled in Part 2. Approximately 30 participants each will be enrolled in Parts 2A, 2B [redacted], with up to an additional approximately 30 patients at an alternative dose(s) as discussed above.

All participants will undergo up to 28 days of screening prior to study entry. Eligible participants will then receive study intervention for up to 2 years, or until disease progression, unacceptable toxicities, a decision by the participant (withdrawal of consent or no longer willing to participate) or investigator to discontinue treatment, or study termination. Any additional treatment beyond 2 years shall be discussed and approved by the sponsor. After EOT, all participants will complete a 28 day post-treatment follow up visit for AEs. Participants in Part 2 dose expansion cohorts will be contacted by telephone approximately every 3 months for survival data collection until end of trial (2 years from last participant first dose), unless otherwise notified by the sponsor.

A participant is considered to have completed the study if he/she has completed all phases of the study including the last scheduled procedure shown in the schedule of activities, including overall survival (OS). OS follow-up should not continue after end of the study (Section 4.4).

During treatment with study intervention, all cycles will be 28 days in length. Every effort should be made to administer study intervention on the planned dose and schedule. In the event of significant toxicities, dosing may be interrupted, modified, or discontinued (Section 6.6).

The biomarker studies will be used to help understand the in vivo mechanism of action of the agent(s) studied as well as potential mechanisms of resistance. The studies may help in the future development of PF-07248144 as a single agent, or in combination with other compounds, and may provide information on tumor sub types that may respond to the study intervention.

4.2. Scientific Rationale for Study Design

4.2.1. Preclinical rationale for PF-07248144

PF-07248144 displayed potent cellular effects as measured by its ability to reduce H3K23Ac levels (on-target PD marker) and anti-proliferation in ER + HER2- breast cancer cell lines. Furthermore, single agent anti-tumor activities were confirmed [redacted]

090177e19bd138e0\Approved\Approved On: 14-Nov-2022 20:57 (GMT)

[redacted]

[redacted] n addition, [redacted] herefore, the use of a highly effective method of contraception is required (see Appendix 4: Section 10.4).

4.2.2. Preclinical rationale for PF-07248144 (KAT6 [redacted]

KAT6A and KAT6B acetyltransferases are involved in regulation of fundamental cellular process including gene transcription, cellular senescence, tissue differentiation and stem cell maintenance[7,8]. In ER+ breast cancer, KAT6A is a direct transcriptional regulator of ESR1 and promotes growth of ER+ breast cancer cells by driving ER signaling[15]. KAT6A can also promote cell proliferation by upregulating MYC transcriptional programs along with inhibition of cellular senescence via the INK4A-ARF pathway[9,21]. [redacted]

[redacted]

[redacted]

[redacted]

090177e19bd138e0\Approved\Approved On: 14-Nov-2022 20:57 (GMT)

[redacted]

[redacted]

[redacted]

[redacted]	[redacted]	[redacted]	[redacted]	[redacted]	[redacted]	[redacted]
[redacted]	[redacted]	[redacted]	[redacted]	[redacted]	[redacted]	[redacted]
[redacted]	[redacted]	[redacted]	[redacted]	[redacted]	[redacted]	[redacted]
[redacted]	[redacted]	[redacted]	[redacted]	[redacted]	[redacted]	[redacted]
[redacted]	[redacted]	[redacted]	[redacted]	[redacted]	[redacted]	[redacted]
[redacted]	[redacted]		[redacted]	[redacted]	[redacted]	[redacted]
[redacted]	[redacted]		[redacted]	[redacted]	[redacted]	[redacted]
[redacted]	[redacted]		[redacted]	[redacted]	[redacted]	[redacted]
[redacted]	[redacted]	[redacted]	[redacted]	[redacted]	[redacted]	[redacted]

[redacted]

[redacted]

[redacted]

[redacted]

090177e19bd138e0\Approved\Approved On: 14-Nov-2022 20:57 (GMT)

[redacted]

PF-07248144 (current study C4551001) has demonstrated a tolerable safety profile with reversible and manageable hematological toxicities (Section 2.2.4).

3 DLTs were reported (G3 neutropenia) during dose escalation and all were resolved with dose modification. Grade ≥3 treatment-related AEs in more than one participant include: neutrophil count decreased/neutropenia in 17 participants (28.8%); anemia in 6 participants (10.2%) and white blood cell count decreased in 4 participants (6.8%).

The dosing regimen of 5 mg QD was identified as the RDE for monotherapy and fulvestrant combination. Encouraging clinical responses (eg, PRs) were observed in monotherapy and the fulvestrant combination in heavily pretreated ER+ HER2- breast cancer.

[redacted]

[redacted]

[redacted]

[redacted]

[redacted]

[redacted]

090177e19bd138e0\Approved\Approved On: 14-Nov-2022 20:57 (GMT)

[redacted]

4.2.4. Estrogen Receptor (ER) Positive Breast Cancer

Breast cancer is the most common noncutaneous cancer in women with over 275,000 new cases expected to be diagnosed in the US in 2020[23]. Approximately 70-80% of new breast cancers express the estrogen and/or progesterone receptor. A more contemporary approach to treat ER-positive advanced or metastatic breast cancer involves combining novel agents with existing endocrine therapy to block pathways enabling partial or complete endocrine resistance. [redacted] Although patients with ER-positive tumors can benefit from hormonal therapy and CDK4/6 inhibitors, the majority of- these women develop resistance to endocrine therapy at some point during treatment. Therefore, there is a high unmet medical need to develop novel agents. [redacted] Together, these data provide rationale for exploring potential application of PF-07248144 as monotherapy and in combination with standard of care agents [redacted] in ER+HER2- breast cancer.

4.2.5. Prostate Cancer

Prostate cancer is the most common cancer in American men (aside from skin malignancies). The American Cancer Society estimates that in the US in 2020 there will be 191,930 newly diagnosed cases of prostate cancer and over 33,000 prostate cancer-related deaths[23]. The early stages of prostate cancer are localized to the prostate, often indolent in nature, and treatable by operative resection or radiotherapy. However, the majority of patients are at a high risk of disease recurrence; and, once metastatic, of disease progression. Prior to the recent approval of NHT (enzalutamide, abiraterone acetate/prednisone), the only approved therapies for CRPC were docetaxel, cabazitaxel and sipeleucel-T. The approval of NHTs in metastatic CRPC previously treated with docetaxel represented a therapeutic advance for these patients[24, 25,]. Given this disease biology, there is high unmet medical need for new therapies to provide long-term disease control while offering a better quality of life with manageable side effect profiles. Preclinically, PF-07248144 has demonstrated potent cellular activity in prostate cancer models [redacted], thus providing rationale for exploring potential application of PF-07248144 in prostate cancer.

4.2.6. NSCLC

The American Cancer Society estimates that there will be over 228,000 new cases of lung cancer diagnosed in the US in 2020, with over 135,000 deaths.[23] About 80-85% of lung cancers are histologically classified as NSCLC, which includes 2 major sub-types: 1) non-squamous carcinoma (including adenocarcinoma, large-cell carcinoma [rare], and other

090177e19bd138e0\Approved\Approved On: 14-Nov-2022 20:57 (GMT)

cell types) and 2) squamous cell (epidermoid) carcinoma. The 5-year survival rate of metastatic NSCLC is 4%. Pembrolizumb, an immune checkpoint inhibitor, is approved either as a monotherapy or in combination with chemotherapy for first-line treatment of NSCLC patients without a targetable oncogene driver (eg, EGFR, ALK, ROS1).[26] Treatment options are limited once a patient progresses on a PD-1/PD-L1 inhibitor (eg docetaxel, ramucirumab), thus there is still a high unmet medical need to find novel therapies to address this patient population. Preclinically, analysis of functional genomics data [redacted] providing rationale for potential exploration of PF-07248144 in NSCLC based on emerging data.

4.2.7. Background and Rationale for Biomarker Assessment

The objectives of the biomarker assessments in this study are: 1) to assess the pharmacodynamic effects of PF-07248144 as monotherapy, in combination with ET, [redacted] by characterizing modulation of PD biomarkers in both tumor tissue and peripheral blood. These [redacted] in tumor [redacted] and changes [redacted]; and 2) to explore potential predictive biomarker(s) and mechanisms of resistance to PF-07248144 therapy, when used as either a single agent, or in combination therapy.

The biomarker assessments may contribute to confirming target engagement, determining PD effects of PF-07248144, identifying those participants who are most likely to benefit from treatment, and identify potential resistance mechanisms to PF-07248144. These assessments may also be used to correlate the PD effect with PK parameters of PF-07248144, and to aid the selection of the RDE of PF-07248144. [redacted] as described in the SoA and the Laboratory Manual.

Banked Biospecimens will be collected and stored for further analyses which may, for example, provide greater understanding of the study intervention.

4.3. Justification for Dose

Doses presented are projected based on nonclinical data and may be modified based on emerging safety, tolerability, and PK data.

4.3.1. PF-07248144 Starting Dose for Monotherapy Dose Escalation

The selection of the starting dose for this FIH study is based on the preclinical toxicology results in accordance with ICH S9 Guidance entitled “Nonclinical Evaluation for Anticancer Pharmaceuticals”.

The starting dose for [redacted]

090177e19bd138e0\Approved\Approved On: 14-Nov-2022 20:57 (GMT)

[redacted]

The predicted apparent half-life by the PBPK modeling is approximately 12 hours at the dose of 8 mg, suggesting a QD dosing regimen. For additional information, please see the Investigator Brochure (IB). [redacted]

In summary, [redacted].

[redacted]

[redacted]

[redacted]

090177e19bd138e0\Approved\Approved On: 14-Nov-2022 20:57 (GMT)

[redacted]

[redacted]

[redacted]

[redacted]

[redacted]

[redacted] [redacted]

	[redacted]	[redacted]	[redacted]
[redacted]	[redacted]	[redacted]	[redacted]

[redacted]

090177e19bd138e0\Approved\Approved On: 14-Nov-2022 20:57 (GMT)

[redacted] [redacted]

[redacted]	[redacted]
[redacted]	[redacted]
[redacted]	[redacted]
[redacted]	[redacted]
[redacted]	[redacted]

[redacted] [redacted]

[redacted]	[redacted]
[redacted]	[redacted]
[redacted]	[redacted]
[redacted]	[redacted]

4.3.3. Dose Increment

In Part 1A, the maximum allowable dose increment of PF-07248144 is 100%. However, a maximum allowable dose-increment >100% (up to 150%) may be considered based on the emerging PK data.

[redacted]

Review of single and multiple dose PK data, together with Cycle 1 safety observations, from all available participants will be conducted prior to determining whether a >100% (up to 150%) maximum allowable dose increment will be implemented. This decision should be discussed and approved by both investigator and sponsor.

For combination dose escalation in Parts 1B [redacted] a maximum allowable dose increment of PF-0728144 [redacted]) to a dose level already determined to be safe as monotherapy may be allowed. For instance, a dose increment from 2 mg QD to 5 mg QD for PF-07248144 may be considered as guided by the BLRM with EWOC principle.

The dose of PF-07248144 [redacted] in combination, may not exceed the monotherapy MTD/RDE [redacted], unless emerging clinical data suggest combination may significantly reduce exposure [redacted] in

090177e19bd138e0\Approved\Approved On: 14-Nov-2022 20:57 (GMT)

which case further dose increase may be considered based on the consensus of investigators and Sponsor.

Not all the dose levels explored in the monotherapy studies may be tested in combination. Intermediate doses may be evaluated based on emerging clinical safety and PK/PD data.

4.3.4. Criteria for Dose Escalation

BLRM guided by the EWOC principle will be used in dose escalation. A traditional 2 -parameter BLRM will be used to model the dose/DLT relationship of PF-07248144 monotherapy and, BLRMs developed specifically for combinations will be used to model the dose/DLT relationship of PF-07248144 given in combination with fulvestrant (Part 1B), [redacted] Using DLT data at all tested dose levels and pre-specified prior distribution of model parameters, the posterior distribution for probability of having a DLT will be calculated for all dose levels and dose recommendations using EWOC will be provided.

Dose recommendation will be based on the probability that the true DLT rate for each dose lies in 1 of the following categories:

Under-dosing:	[0, 0.16]
Targeted dosing:	[0.16, 0.33]
Overdosing:	[0.33, 1]

A dose may only be used for newly enrolled participants if it satisfies EWOC criteria: the risk of excessive toxicity (overdosing) at that dose is less than 25%.

The maximum allowable PF-07248144 dose increment is 100% except as defined in Section 4.3.3.

Dose escalation will stop when stopping criteria are met (see Section 9.4.1.1). In an unlikely situation of observing DLT(s) at the starting dose level, lower dose levels might be considered. The dose level will be recommended by BLRM in such a way that EWOC criteria are fulfilled.

Intra-participant dose escalation will not be permitted in this study. In case of change of the dosing regimen, DLT data accumulated during the dose escalation with the original regimen might be used to form a prior for further BLRM analysis. Details about derivation of this prior using Meta-Analytic-Predictive (MAP) approach can be found in Section 10.9.

4.3.5. Dose Limiting Toxicity Definition

DLT evaluable: A participant is classified as DLT evaluable if the participant experiences a DLT or if the participant otherwise in the absence of a DLT receives at least 75% of the planned doses of PF-07248144 and has received all scheduled safety assessments during the

090177e19bd138e0\Approved\Approved On: 14-Nov-2022 20:57 (GMT)

DLT observation period. If a participant fails to meet these criteria, the participant is classified as not DLT-evaluable and may be replaced.

DLT observation period: For the purpose of dose escalation, the DLT observation period for monotherapy dose escalation (Part 1A) and combination dose escalation (Part 1B, [redacted]) will be the first cycle of treatment (within 28 days of the first dose) with inclusion of the Cycle 2 Day 1 (Day 29) laboratory assessments in each participant.

Significant AEs considered to be related to the study intervention or treatment under investigation that occur after the DLT observation period will be reviewed in the context of all safety data available. That review may result in re-evaluation of the dosing level or regimen.

Severity of AEs will be graded according to CTCAE version 5.0. For the purpose of dose escalation/finding, any of the following AEs occurring in the first cycle of treatment (28 days) which are attributable to one, the other, or all agents in the combination will be classified as DLTs:

Hematological Dose-Limiting Toxicities:

- Grade 4 neutropenia regardless of intervention is a DLT.
- Febrile neutropenia (defined as an ANC <1000/mm^3 with a single temperature of >38.3°C [101°F], or a sustained temperature of ≥38°C [100.4°F] for more than 1 hour) is a DLT.
- Grade 3 neutropenia with infection is a DLT (Note: isolated Grade 3 neutropenia without accompanying fever or infection as defined above is not a DLT).
- Grade 4 thrombocytopenia is a DLT.
- Grade 3 thrombocytopenia with bleeding or requiring platelet transfusion is a DLT.
- Grade 4 anemia is a DLT.
- Grade 3 anemia requiring blood transfusion is a DLT.

Non-Hematologic Dose-Limiting Toxicities:

Any Grade ≥3 non-hematologic possibly treatment related AE is a DLTs with the following clarifications:

- Grade ≥3 nausea, vomiting, or diarrhea lasting ≥3 days despite adequate antiemetic and other supportive care is a DLT.
- Grade ≥3 fatigue lasting ≥7 days is a DLT.

090177e19bd138e0\Approved\Approved On: 14-Nov-2022 20:57 (GMT)

- Confirmed DILI meeting Hy's law criteria is a DLT.
- For participants with Grade 2 hepatic transaminase or alkaline phosphatase levels at baseline as a result of liver metastasis or bone metastasis, AST or ALT >10 x ULN or AST or ALT >5 x ULN for ≥14 days will be considered a DLT
- Clinically important or persistent toxicities (eg, toxicities responsible for significant dose delay) that are not included in the above criteria may also be considered a DLT following review by the investigators and the Sponsor. All DLTs need to represent a clinically significant shift from baseline.
- Grade ≥3 QTc prolongation is a DLT.
- Grade ≥3 anaphylaxis is a DLT.

Any toxicity causing greater than 2 weeks of dose delay of either PF-07248144 [redacted] is a DLT. In addition, any Grade 5 AE (death) not clearly due to either the underlying disease or other etiologies is a DLT. *Note*: Participants deriving clinical benefit from study intervention who experience a DLT may continue on study at a reduced dose following recovery of the AE to Grade 1 or baseline, only after discussion between the Investigator and Sponsor.

Any dose reduction of either PF-07248144 [redacted] due to a treatment-related AE (per protocol) during the first 28 days will be qualified as participant experiencing DLT.

The following AEs will not be adjudicated as DLTs:

- Isolated Grade 3 laboratory abnormalities that are not associated with clinical sequelae and are corrected or resolved to Grade 1 or baseline with supplementation/appropriate management within 72 hours of their onset.

Adverse events consistent with the definition of a DLTobserved after the DLT observation period may also be considered in dose escalation decisions and the final determination of the MTD/RDE as monotherapy and in combination.

4.3.6. Maximum Tolerated Dose Definition

MTD is defined as a dose with true DLT rate from the target toxicity interval. The target interval for the DLT rate is defined as (0.16, 0.33).

4.3.7. Recommended Phase 2 Dose and Recommended Dose for Expansion Definition

The RP2D is the dose chosen for further investigation based on Phase 1 study results. The monotherapy RDE is the dose chosen for further investigation based on Part 1A dose escalation results. If the MTD proves to be clinically feasible for long term administration in a reasonable number of participants, then this dose becomes the RDE. However, RDE dose might be lower than the MTD. Safety, efficacy, and PK data as well potential exposure-response relationships will be considered in identifying the RDE.

090177e19bd138e0\Approved\Approved On: 14-Nov-2022 20:57 (GMT)

Similarly, based on the safety, tolerability and PK/PD data from Part 1B, [redacted], combination dose finding, [redacted] RDE will be selected. The final combination "Study" RP2D will be determined after the completion of combination expansion cohorts. Combination RDE may be different from monotherapy RDE due to potential toxicity overlap or drug-drug interaction.

Each dose expansion cohort may investigate more than one dose of each of the study interventions. All dose levels to be evaluated will be informed by available data from dose escalation (including safety, PK/PD, and efficacy) and will not exceed each agent's MTD. [redacted]

After dose expansion, the final "Study" RP2Ds will be determined by the sponsor based on the recommendation from investigators and study team. The determination of "Study" RP2D will be based on safety, tolerability and early signs of clinical efficacy and benefit from both Part 1 dose escalation and Part 2 expansion.

During escalation and prior to an MTD being reached, the sponsor may choose to advance a lower dose into expansion, particularly (but not exclusively) when considering combination cohorts. This decision will be made based on emerging preliminary safety, PK, PD, and/or efficacy data.

4.3.8. [redacted]

[redacted]

[redacted]

[redacted]

[redacted]

090177e19bd138e0\Approved\Approved On: 14-Nov-2022 20:57 (GMT)

[redacted]

4.4. End of Study Definition

The end of the study is defined as 2 years from the last participant's first dose unless otherwise notified by the sponsor.

5. STUDY POPULATION

This study can fulfill its objectives only if appropriate participants are enrolled. The following eligibility criteria are designed to select participants for whom participation in the study is considered appropriate. All relevant medical and nonmedical conditions should be taken into consideration when deciding whether a particular participant is suitable for this protocol.

Prospective approval of protocol deviations to recruitment and enrollment criteria, also known as protocol waivers or exemptions, is not permitted.

During the COVID-19 pandemic, please refer to Appendix 13 (Section 10.13) for additional eligibilities.

5.1. Inclusion Criteria

Participants are eligible to be included in the study only if all of the following criteria apply:

1. Adult participants age ≥18 years (please follow local regulatory requirements if the legal age of consent for study participation is less than 18 years old). For participants enrolled at clinical sites in Japan: adult participants age ≥20 years. For participants enrolled at clinical sites in South Korea: adult participants age ≥19 years.
    - Refer to Appendix 4 for reproductive criteria for male (Section 10.4.1) and female (Section 10.4.2) participants.
2. **Part 1 (Dose Escalation) tumor indications and prior lines of therapy**
    - **Part 1A (Monotherapy Dose Escalation):** Histological or cytological diagnosis of locally advanced or metastatic ER+HER2- breast cancer, locally advanced or metastatic CRPC, or locally advanced or metastatic NSCLC that is intolerant or resistant to standard therapy or for which no standard therapy is available.
    - **Part 1B (Combination Dose Escalation):** Histological or cytological diagnosis of locally advanced or metastatic ER+HER2- breast cancer. Participants must have progressed after at least 1 prior line of treatment with an endocrine therapy and CDK4/6 inhibitor in the advanced or metastatic setting.
    - [redacted]

[redacted]

090177e19bd138e0\Approved\Approved On: 14-Nov-2022 20:57 (GMT)

[redacted]

- [redacted]
- Intolerance or progression on prior therapies must be documented for study enrollment.

3. **Part 2 (Dose Expansion) tumor indications and prior lines of therapy**

    - **Part 2A (ER+HER2- breast cancer 2L+, monotherapy):** Histological or cytological diagnosis of locally advanced or metastatic ER+HER2- breast cancer. Participants must have progressed after at least 1 prior line of CDK4/6 inhibitor and at least 1 prior line of endocrine therapy. Additional Korea specific requirements are provided in Appendix 8 (Section 10.8.3).
    - **Part 2B (ER+HER2- breast cancer 2-4L, combination with fulvestrant):**
        - Histological or cytological diagnosis of advanced or metastatic ER+HER2- breast cancer. Participants must have progressive disease after at least 1 prior line of a CDK4/6 inhibitor and at least 1 prior line of endocrine therapy.
        - Participants must not have received more than 3 prior lines of systemic therapies including up to 1 line of cytotoxic chemotherapy for visceral disease in advanced or metastatic setting; Participants may have but are not required to have prior treatment with fulvestrant.
    - [redacted]
        - [redacted]
        - [redacted]

4. Participants with ER+HER2- advanced or metastatic breast cancer must have documentation of ER-positive tumor (≥1% positive stained cells) based on most recent tumor biopsy (unless non-measurable disease where most recent documentation will be provided) utilizing an assay consistent with local standards.

090177e19bd138e0\Approved\Approved On: 14-Nov-2022 20:57 (GMT)

5. Participants with ER+HER2- advanced or metastatic breast cancer must have documentation of HER2-negative tumor: HER2-negative tumor is determined as immunohistochemistry score 0/1+ or negative *by in situ* hybridization (FISH/CISH/SISH/DISH) defined as a HER2/CEP17 ratio <2 or for single probe assessment a HER2 copy number <4.

6. Female participants with ER+HER2- advanced or metastatic breast cancer considered to be of childbearing potential (or have tubal ligations only) must be willing to undergo medically induced menopause by treatment with the approved LHRH agonist such as goserelin, leuprolide or equivalent agents to induce chemical menopause.

7. Female participants with ER+HER2- advanced or metastatic breast cancer of nonchildbearing potential must meet at least 1 of the following criteria of achieving postmenopausal status, defined as follows:

   - Cessation of regular menses for at least 12 consecutive months with no alternative pathological or physiological cause; [status may be confirmed with/and have] a serum follicle-stimulating hormone (FSH) level confirming the post menopausal state;

   - Have undergone a documented hysterectomy and/or bilateral oophorectomy;

   - Have medically confirmed ovarian failure. All other female participants (including female participants with tubal ligations) are considered to be of childbearing potential.

8. Participants must have at least 1 measurable lesion as defined by RECIST version 1.1 that has not been previously irradiated.

9. ECOG Performance Status PS 0 or 1 (See Appendix 12: Section 10.12).

10. Adequate Bone Marrow Function, including:

    a. ANC ≥1,500/mm3 or ≥1.5 x 109/L;

    b. Platelets ≥100,000/mm3 or ≥100 x 109/L;

    c. Hemoglobin ≥9 g/dL.

11. Adequate Renal Function, including:

    a. Serum creatinine ≤1.5 x ULN or estimated creatinine clearance GFR ≥60 mL/min (≥50 mL/min for Part 2 dose expansion is acceptable) as calculated using the method standard for the institution. In equivocal cases, a 24 hour urine collection test can be used to estimate the creatinine clearance more accurately.

090177e19bd138e0\Approved\Approved On: 14-Nov-2022 20:57 (GMT)

12. Adequate Liver Function, including:

    a. Total serum bilirubin ≤1.5 x ULN unless the participant has documented Gilbert syndrome;

    b. AST and ALT ≤2.5 x ULN; AST and ALT ≤3.0 x ULN if there is liver involvement by the tumor for Part 1 dose escalation. AST and ALT ≤5.0 x ULN if there is liver involvement by the tumor for Part 2 dose expansion.

13. Resolved acute effects of any prior therapy to baseline severity or CTCAE Grade ≤1 except for AEs not constituting a safety risk by investigator judgment.

14. Participants who are willing and able to comply with all scheduled visits, treatment plan, laboratory tests, lifestyle considerations, and other study procedures.

15. Capable of giving signed informed consent as described in Section 10.1.3, which includes compliance with the requirements and restrictions listed in the ICD and in this protocol.

5.2. Exclusion Criteria

Participants are excluded from the study if any of the following criteria apply:

1. Participants with known symptomatic brain metastases requiring steroids. Participants with previously diagnosed brain metastases are eligible if they have completed their treatment and have recovered from the acute effects of radiation therapy or surgery prior to study entry, have discontinued corticosteroid treatment for these metastases for at least 3 weeks and are neurologically stable for 2 months (requires MRI confirmation).

2. Participants with advanced/metastatic, symptomatic, visceral spread, that are at risk of life-threatening complications in the short term (including participants with massive uncontrolled effusions [pleural, pericardial, peritoneal], pulmonary lymphangitis, and over 50% liver involvement). Note: Participants with indwelling catheter for drainage, or requirement for drainage no more frequently than monthly will be allowed.

3. Participants with any other active malignancy within 3 years prior to enrollment, except for adequately treated basal cell or squamous cell skin cancer, or carcinoma in situ. Other indolent cancers that do not interfere with assessment of primary cancer under study may be allowed with prior sponsor approval.

4. Major surgery within 3 weeks prior to study entry.

5. Radiation therapy within 3 weeks prior to study entry.

6. Systemic anti-cancer therapy within 3 weeks prior to study entry. If the last immediate anti-cancer treatment contained an antibody based agent(s) (approved or

090177e19bd138e0\Approved\Approved On: 14-Nov-2022 20:57 (GMT)

investigational), then an interval of 28 days or 5 half-life (whichever is shorter) of the agent(s) prior to receive the study intervention treatment is required.

7. Prior irradiation to >25% of the bone marrow (see Appendix 10: Section 10.10).

8. Participants with active, uncontrolled bacterial, fungal, or viral infection, including (but not limited to) HBV, HCV, known HIV or AIDS related illness. HIV seropositive subjects who are healthy and low risk for AIDS-related outcomes could be considered eligible.

   - Eligibility criteria for HIV-positive subjects should be evaluated and discussed with sponsor's medical monitor and will be based on current and past CD4 and T-cell counts, history (if any) of AIDS-defining conditions (eg, opportunistic infections), and status of HIV treatment. Also, the potential for DDIs will be taken into consideration. In equivocal cases, with positive serology, those participants with a negative viral load are potentially eligible provided the other entry criteria are met.

9. Unmanageable ascites (limited medical treatment to control ascites is permitted, but all participants with ascites require review by sponsor's medical monitor).

10. Baseline 12 -lead ECG that demonstrates clinically relevant abnormalities that may affect participant safety or interpretation of study results (eg, baseline QTc interval >470 msec, complete LBBB, signs of an acute myocardial infarction, ST changes suggestive of active myocardial ischemia, second- or third- degree AV block, or serious bradyarrhythmias or tachyarrhythmias). If the baseline uncorrected QT interval is >470 msec, this interval should be rate corrected using the Fridericia method and the resulting QTcF- should be used for decision making and reporting. If QTcF exceeds 470 msec, or QRS exceeds 120 msec, the ECG should be repeated 2 more times and the average of the 3 QTcF or QRS values should be used to determine the participant's eligibility. Computer -interpreted ECGs should be overread by a physician experienced in reading ECGs before excluding participants. Cases must be discussed in detail with sponsor's medical monitor to judge eligibility.

11. Any of the following in the previous 6 months: myocardial infarction, long QT syndrome, Torsade de Pointes, clinically important atrial or ventricular arrhythmias (including sustained ventricular tachyarrhythmia and ventricular fibrillation), serious conduction system abnormalities (eg, bifascicular block [defined as right bundle branch and left anterior or posterior hemiblock], 3rd degree AV block), unstable angina, coronary/peripheral artery bypass graft, symptomatic CHF, New York Heart Association class III or IV, cerebrovascular accident, transient ischemic attack, symptomatic pulmonary embolism, and/or other clinical significant episode of thrombo embolic disease and ongoing cardiac dysrhythmias of NCI CTCAE $\geq$ Grade 2. For Grade 2 atrial fibrillation, may be considered eligible with sponsor approval (e.g if improved to Grade 1 with non-urgent medical intervention or chronic Grade 2 atrial fibrillation with good rate control with non-urgent medical

090177e19bd138e0\Approved\Approved On: 14-Nov-2022 20:57 (GMT)

[redacted]

intervention). If a participant has a cardiac rhythm device/pacemaker placed and QTcF >470 msec, the participant can be considered eligible. Participants with cardiac rhythm device/pacemaker must be discussed in detail with sponsor's medical monitor to judge eligibility.

12. Therapeutic anticoagulation. [redacted] may allow therapeutic antibogulation with LMWH, Vitamin K antagonists or factor Xa inhibitors following discussion with the Sponsor.

13. Hypertension that cannot be controlled by optimal medical therapy (eg, ≥160/100 mmHg).

14. Participation in other studies involving investigational drug(s) within 3 weeks prior to study entry. Participation in long term follow-up of other studies is allowed if no procedures which may interfere with the interpretation of study results will be performed.

15. Known or suspected hypersensitivity or severe allergy to active ingredient/excipients of study drug(s).

16. Prior treatment with study drug(s).

17. Active inflammatory GI disease, refractory and unresolved chronic diarrhea or previous gastric resection, lap band surgery or other GI conditions and surgeries that may significantly alter the absorption of PF-07248144 [redacted] tablets. Gastroesophageal reflux disease under treatment is allowed.

18. [redacted]

[redacted]

[redacted]

[redacted]

090177e19bd138e0\Approved\Approved On: 14-Nov-2022 20:57 (GMT)

[redacted]

22. [redacted]

[redacted]

26. Positive serum or urine pregnancy test (for females of childbearing potential) at screening.

27. Other medical or psychiatric condition including recent (within the past year) or active suicidal ideation/behavior or laboratory abnormality that may increase the risk of study participation or, in the investigator's judgment, make the participant inappropriate for the study.

28. Investigator site staff or Pfizer employees directly involved in the conduct of the study, site staff otherwise supervised by the investigator, and their respective family members.

Please refer to Appendix 8 (Section 10.8.1) for additional Japan specific requirements.

5.3. Lifestyle Considerations

The following guidelines are provided:

5.3.1. [redacted]

[redacted]

090177e19bd138e0\Approved\Approved On: 14-Nov-2022 20:57 (GMT)

[redacted]

5.3.2. Contraception

The investigator or his or her designee, in consultation with the participant, will confirm that the participant has selected an appropriate method of contraception for the individual participant and his or her partner(s) from the permitted list of contraception methods (see Appendix 4: Section 10.4.4) and will confirm that the participant has been instructed in its consistent and correct use. At time points indicated in the SoA, the investigator or designee will inform the participant of the need to use highly effective contraception consistently and correctly and document the conversation and the participant's affirmation in the participant's chart (participants need to affirm their consistent and correct use of at least 1 of the selected methods of contraception). In addition, the investigator or designee will instruct the participant to call immediately if the selected contraception method is discontinued or if pregnancy is known or suspected in the participant or partner.

5.4. Screen Failures

Screen failures are defined as participants who consent to participate in the clinical study, who do not meet 1 or more criteria required for participation in the study during the screening procedures and are not subsequently assigned to the study intervention. A minimal set of screen failure information is required to ensure transparent reporting of screen failure participants, to meet the CONSORT publishing requirements and to respond to queries from regulatory authorities. Minimal information includes demography, screen failure details, eligibility criteria, and any SAEs.

Individuals who do not meet the criteria for participation in this study (screen failure) may be rescreened no more than 1 time within the screening period. Rescreened participants should be assigned a different participant number as the initial screening and the previous screening data, including initial SSID will be captured in the database.

6. STUDY INTERVENTION

Study intervention is defined as any investigational intervention(s), marketed product(s), placebo, medical device(s), or study procedure(s) intended to be administered to a study participant according to the study protocol.

For the purposes of this protocol, study intervention refers to PF-07248144, [redacted] fulvestrant, [redacted]

090177e19bd138e0\Approved\Approved On: 14-Nov-2022 20:57 (GMT)

6.1. Study Intervention(s) Administered

**Intervention Name**	**PF-07248144**	[redacted]	[redacted]	**fulvestrant**	[redacted]
**ARM Name (group of participants receiving a specific treatment (or no treatment)**	Part 1A, 1B, [redacted] 2A[redacted]	[redacted]	[redacted]	Part 1B, [redacted] 2B, [redacted]	[redacted]
**Type**	Drug	[redacted]	[redacted]	Drug	[redacted]
**Dose Formulation**	Tablet	[redacted]	[redacted]	Injection	[redacted]
**Unit Dose Strength(s)**	0.5, 5 mg	[redacted]	[redacted]	250 mg	[redacted]
**Dosage Level(s)**	TBD	[redacted]	[redacted]	500 mg	[redacted]
**Route of Administration**	Oral	[redacted]	[redacted]	Intramuscular Injection	[redacted]
**Use**	Experimental	[redacted]	[redacted]	Experimental	[redacted]
**IMP or NIMP**	IMP	[redacted]	[redacted]	IMP	[redacted]
**Sourcing**	Supplied by the Sponsor	[redacted]	[redacted]	Supplied by the Sponsor	[redacted]
**Packaging and Labeling**	Study intervention will be provided in bottles. Each bottle will be labeled as required per country requirement.	[redacted]	[redacted].	Study intervention will be provided in commercial packaging, Each package will be labeled as required per country requirement.	[redacted]

[redacted]

6.1.1. Administration

6.1.1.1. PF-07248144

Participants will swallow PF-07248144 whole and will not manipulate or chew the study intervention prior to swallowing. PF-07248144 will initially be administered QD by mouth for all cohorts on a continuous basis. [redacted]

090177e19bd138e0\Approved\Approved On: 14-Nov-2022 20:57 (GMT)

A cycle is defined as 28 days, regardless of missed doses or dose delays.

PF-07248144 will be administered orally on an empty stomach (except during the food effect evaluation) and participants will be instructed to take their medication at approximately the same time each day and to not take more than the prescribed dose at any time.

Oral PF-07248144 will be administered QD with at least 8 oz (240 mL) of water on an empty stomach. No food or liquids other than water will be consumed for 2 hours before and 2 hours following each dose throughout the study for QD dosing.

If a participant misses a dose at the scheduled time, the participant can still take the dose within 3 hours of the scheduled time. If the participant missed taking a dose after 3 hours of the scheduled time, or day of treatment, they must be instructed not to "make it up" but resume subsequent doses as prescribed. This dose will be considered as a missed dose. In addition, if a participant vomits any time after taking a dose; they should not "make it up" but to resume subsequent doses as prescribed. Lastly, if a participant inadvertently takes 1 extra dose during a day, the participant should not take the next dose of PF-07248144. A dosing diary will be given to each participant to support at-home dosing.

6.1.1.2. Fulvestrant

Fulvestrant will be administered intramuscularly into the buttocks (gluteal area) slowly (1-2 minutes per injection) as two 5 mL injections, 1 in each buttock, on C1D1, C1D15, C2D1, and once monthly thereafter according to product labeling and in compliance with its local prescribing information.

[redacted]

[redacted]

[redacted]

[redacted]

[redacted]

[redacted]

090177e19bd138e0\Approved\Approved On: 14-Nov-2022 20:57 (GMT)

[redacted]

6.1.1.6. [redacted]

For all participants in the food effect cohort (a subset of Part 2A), PF-07248144 will be administered following an overnight fast of at least 10 hours on Cycle 1 Day-7 and Cycle 1 Day 1. On Cycle 1 Day -7 (Fed state) a test breakfast meal (described below) will be provided and must be consumed over 30 minutes. PF-07248144 will be administered with approximately 8oz (240 mL) of water 30 minutes after the start of the meal. No additional food will be allowed until at least 4 hours postdose. On Cycle 1 Day 1, patients will receive another single oral dose of PF-07248144 under fasted condition following an overnight fast of at least 10 hours. PF-07248144 will be administered with 8 oz (240 mL) of water. No food will be allowed for an additional 4 hours postdose. For either treatment day, water will be allowed ad libitum except for 1 hr before and 1 hr after drug administration. Starting from Cycle 1 Day 2 onwards, PF-07248144 will be administered QD with at least 8 oz (240 mL) of water on an empty stomach, with no food or liquids other than water for 2 hours before and 2 hours following dosing.

[redacted] Substitutions to this test meal can be made after discussion with the sponsor, as long as the meal provides a similar amount of calories from protein, carbohydrate, and fat and has comparable meal volume and viscosity (if substitutions are made, the contents of the meal will be documented by a dietitian or designate to confirm it matches the FDA requirements for protein, carbohydrate and fat described above). However, it is understood that some participants may not be able to

090177e19bd138e0\Approved\Approved On: 14-Nov-2022 20:57 (GMT)

consume the entire meal. Study staff should record the percent of the test meal breakfast and the time it takes to be consumed.

6.2. Preparation/Handling/Storage/Accountability

1. The investigator or designee must confirm appropriate temperature conditions have been maintained during transit for all study interventions received and any discrepancies are reported and resolved before use of the study intervention.

2. Only participants enrolled in the study may receive study intervention and only authorized site staff may supply or administer study intervention. All study interventions must be stored in a secure, environmentally controlled, and monitored (manual or automated recording) area in accordance with the labeled storage conditions with access limited to the investigator and authorized site staff. At a minimum, daily minimum and maximum temperatures for all site storage locations must be documented and available upon request. Data for nonworking days must indicate the minimum and maximum temperatures since previously documented for all site storage locations upon return to business.

3. Any excursions from the study intervention label storage conditions should be reported to Pfizer upon discovery along with any actions taken. The site should actively pursue options for returning the study intervention to the storage conditions described in the labeling, as soon as possible. Once an excursion is identified, the study intervention must be quarantined and not used until Pfizer provides permission to use the study intervention. Specific details regarding the definition of an excursion and information the site should report for each excursion will be provided to the site in the IP manual.

4. Any storage conditions stated in the SRSD will be superseded by the storage conditions stated on the label.

5. Study interventions should be stored in their original containers.

6. Site staff will instruct participants on the proper storage requirements for take-home study intervention.

7. The investigator, institution, or the head of the medical institution (where applicable) is responsible for study intervention accountability, reconciliation, and record maintenance (ie, receipt, reconciliation, and final disposition records), such as the IPAL or sponsor-approved equivalent. All study interventions will be accounted for using a study intervention accountability form/record. All PF-07248144 [redacted] that is taken home by the participant, both used and unused, must be returned to the investigator by the participant. Returned study intervention must not be re-dispensed to the participants.

8. Further guidance and information for the final disposition of unused study interventions are provided in the IP manual. All destruction must be adequately

090177e19bd138e0\Approved\Approved On: 14-Nov-2022 20:57 (GMT)

documented. If destruction is authorized to take place at the investigator site, the investigator must ensure that the materials are destroyed in compliance with applicable environmental regulations, institutional policy, and any special instructions provided by Pfizer.

9. Upon identification of a product complaint, notify the sponsor within 1 business day of discovery as described in the IP Manual.

6.2.1. Preparation and Dispensing

A qualified staff member will dispense the study intervention in the bottles or packaging provided, in quantities appropriate according to the SoA. A second staff member will verify the dispensing. The participant/caregiver should be instructed to maintain the product in the bottles or packaging provided throughout the course of dosing, keep the study intervention away from children, and return the bottles or packaging to the site at the next study visit. Refer to the IP manual for more detailed instructions.

6.3. Measures to Minimize Bias: Randomization and Blinding

6.3.1. Allocation to Study Intervention

Part 1

Dose level allocation will be performed by the sponsor after participants have given their written informed consent and have completed the necessary baseline assessments. The site staff will email a complete Registration Form to the designated sponsor study team member or designee. The sponsor will assign a participant identification number and supply this number to the site. The participant identification number will be used on all study-related documentation at the site.

No participant will receive study intervention until the investigator or designee has received the following information in writing from the sponsor:

- Confirmation of the participant's enrollment;
- Specification of the dose level for that participant and;
- Permission to proceed with dosing the participant.

Part 2

Open-label using IRT:

This is an open-label study and study intervention dispensed to the participants in Part 2 will be assigned using an IRT system. The site will contact the IRT prior to the start of study intervention administration for each participant in Part 2. The site will record the study intervention assignment on the applicable CRF, if required.

The study-specific IRT reference manual will provide IRT support contact information and further details on the use of the IRT system.

090177e19bd138e0\Approved\Approved On: 14-Nov-2022 20:57 (GMT)

090177e19bd138e0\Approved\Approved On: 14-Nov-2022 20:57 (GMT)

Study intervention will be dispensed at the study visits summarized in the SoA.

Returned study intervention must not be redispensed to the participants.

6.4. Study Intervention Compliance

Participant compliance with investigational product will be assessed at each visit. Compliance will be assessed by direct questioning, counting returned tablets or capsules, and review of the participant diary. Deviation(s) from the prescribed dosage regimen should be recorded in the CRF with >80% of missed doses considered a protocol deviation.

All study intervention will be distributed to the participant by the appropriately designated study staff at the investigational site.

A diary will be provided to the participants to aid in compliance with the dosing instructions. The diary will be maintained by the participant to include missed or changed investigational product doses. Participants will be required to return all investigational product at every cycle and the number of investigational product remaining will be documented and recorded. The participant diary may also be used to support this part of the accountability process via a discussion between the study site staff and the participant. Discrepancies will be documented on the appropriate eCRF.

6.5. Concomitant Therapy

Concomitant treatment considered necessary for the participant's well-being may be given at the discretion of the treating physician.

All concomitant treatments, blood products, as well as nondrug interventions *(eg, paracentesis)* received by participants from screening until the EOT visit will be recorded on the CRF.

6.5.1. [redacted]

[redacted]

[redacted]

[redacted]

[redacted]

- [redacted]
- [redacted]

6.5.2. Fulvestrant, [redacted]

Please refer to the [redacted] product labels of fulvestrant, [redacted] for a list of prohibited concomitant medications.

[redacted]

090177e19bd138e0\Approved\Approved On: 14-Nov-2022 20:57 (GMT)

[redacted]

6.5.3. Other Antitumor/Anticancer or Experimental Drugs

No additional anti-tumor treatment will be permitted while participants are receiving study treatment. Additionally, the concurrent use of vitamins or herbal supplements is not permitted, [redacted]

6.5.4. Supportive Care

Palliative and supportive care for disease related symptoms may be administered at the investigator's discretion and according to the specific supportive care product Prescribing Information or the current ASCO guidelines.

6.5.5. Hematopoietic Growth Factors

Primary prophylactic use of CSF is not permitted during the first 28 days of Cycle 1 Part 1, but they may be used to treat treatment-emergent neutropenia as indicated by the current ASCO guidelines[28]. During the screening window (ie, 28 days prior to Day 1), G-CSF compounds are not permitted to qualify a participant with low WBC counts. For Japan only: since the indication and dosage of G-CSF compounds approved in Japan may differ from ASCO guidelines, please refer to Japanese package insert.

090177e19bd138e0\Approved\Approved On: 14-Nov-2022 20:57 (GMT)

Erythropoietin may be used at the investigator's discretion for the supportive treatment of anemia. (Note: erythropoietin is not approved for anemia caused by chemotherapy in all local regions that includes being not approved in Japan).

6.5.6. Anti-Diarrheal, Anti-Emetic Therapy

Primary prophylactic use of anti-diarrheal and anti-emetic therapy beyond the first cycle is at the investigator's discretion. The choice of the prophylactic drug as well as the duration of treatment is up to the investigator with sponsor approval assuming there is no known or expected drug interaction and assuming the drug is not included in the Concomitant Therapy section.

6.5.7. Anti-inflammatory Therapy

Anti-inflammatory or narcotic analgesic may be offered as needed assuming there is no known or expected drug-drug interaction and assuming the drug is not included in the Concomitant Therapy section.

6.5.8. Corticosteroids

Chronic systemic corticosteroid use (prednisone >10 mg/day or equivalents) for palliative or supportive purposes is not permitted. However, corticosteroid use for a short duration (eg, ≤10 mg/day of prednisone, for 2 weeks) as symptomatic treatment on an individual basis may be considered after discussion with sponsor's medical monitor or designee. For adrenal insufficiency replacement therapy, doses <15 mg/day of prednisone or equivalent will be allowed. Acute emergency administration, topical applications, inhaled sprays, eye drops, or local injections of corticosteroids are allowed.

6.5.9. Surgery

Caution is advised on theoretical grounds for any surgical procedures during the study. The appropriate interval of time between surgery and PF-07248144 required to minimize the risk of impaired wound healing and bleeding has not been determined.

Stopping PF-07248144 is recommended at least 5 days prior to surgery. [redacted] Postoperatively, the decision to reinitiate PF-07248144 [redacted] treatment should be based on a clinical assessment of satisfactory wound healing and recovery from surgery.

6.5.10. Rescue Medicine

There is no rescue therapy to reverse the AEs observed with PF-07248144 [redacted]; standard medical supportive care must be provided to manage the AEs.

6.6. Dose Modification

Every effort should be made to administer PF-07248144 [redacted] on the planned dose and schedule. In the event of significant toxicity, dosing

090177e19bd138e0\Approved\Approved On: 14-Nov-2022 20:57 (GMT)

may be interrupted and/or reduced as described below. In the event of multiple toxicities, dose modification should be based on the worst toxicity observed (and attribution for the combination). Participants are to be instructed to notify investigators at the first occurrence of any adverse symptom.

For fulvestrant, [redacted], dose modification should be made according to product labeling and in compliance with its local prescribing information.

6.6.1. Dosing Interruptions

In the event of significant toxicity, dosing may be interrupted as described in Table 10 and Table 11.

Doses omitted for toxicity are not replaced within the same cycle. The need for a dose reduction at the time of treatment resumption should be based on the criteria defined in Table 10 and Table 11, unless expressly agreed otherwise following discussion between the investigator and the sponsor.

Re-treatment following dose interruption for treatment-related toxicity may not occur until all of the following parameters have been met:

- Platelets count ≥50,000/mm^3.
- Nonhematologic toxicities have returned to baseline or Grade ≤1 severity (or, at the investigator's discretion, Grade ≤2 if not considered a safety risk for the participant).

If treatment(s) interruption was due to worsening of hematologic or biochemical parameters, the frequency of relevant blood tests should be increased as clinically indicated.

If these conditions are met within 4 weeks of treatment(s) interruption, PF-07248144 [redacted] may be resumed. Refer to the Dose Reductions Section 6.6.2 for adverse events requiring dose reduction at the time of treatment(s) resumption.

If participants require discontinuation of PF-07248144 [redacted] for more than 4 weeks at any time during the study, then study treatment should be permanently discontinued, unless the investigator's benefit/risk assessment suggests otherwise after discussion with the Sponsor's medical monitor.

In addition, for combination dose escalation and expansion, dose interruption of 1 or more investigational products may be considered depending on the type and severity of toxicity encountered and the likely attribution based on discussion and consensus of the investigator and Sponsor, see Table 10 and Table 11.

Please see Appendix 13 (Section 10.13) for management of participants who have active confirmed (positive by regulatory authority-approved test) or presumed (test pending/clinical suspicion) SARS-CoV2 infection.

090177e19bd138e0\Approved\Approved On: 14-Nov-2022 20:57 (GMT)

Note: Cycles will not be extended to cover for the missed doses. Refer to Section 6.1.1.1 for information on missed doses.

6.6.2. Dose Reductions

Following dosing interruption due to toxicity, the PF-07248144 [redacted], dose may need to be reduced when treatment is resumed. No specific dose adjustments are recommended for Grade 1 or 2 treatment-related toxicity.

Participants experiencing recurrent and intolerable treatment-related Grade 2 toxicity may resume dosing at the next lower dose level once recovery to Grade ≤1 or baseline is achieved.

Dose reduction of PF-07248144 [redacted] by 1 and, if needed, 2 dose levels will be allowed depending on the type and severity of toxicity encountered.

When a toxicity may be attributed to either PF-07248144 [redacted] and a dose reduction is needed, either the dose for PF-07248144 [redacted] may be reduced based upon the clinical investigator's assessment of the weight of contribution from each study treatment.

Participants requiring more than 2 dose reductions of the PF-07248144 [redacted] will be permanently discontinued from the treatment and enter the follow up phase, unless otherwise agreed between the investigator and the Sponsor. All dose modifications/adjustments must be clearly documented in the participant's source notes and CRF.

Once a dose has been reduced for a given participant, all subsequent cycles should be administered at that dose level, unless further dose reduction is required. Intra-participant dose re-escalation is not allowed unless agreed by both investigator and the sponsor.

Participants experiencing a DLT may resume dosing at the next lower dose level (if applicable) once adequate recovery is achieved, and in the opinion of the investigator and sponsor, the participant is benefiting from therapy.

The following tables provide guidance for dose modifications needed for nonhematologic- (Table 10) and hematologic- (Table 11) treatment related toxicities.

090177e19bd138e0\Approved\Approved On: 14-Nov-2022 20:57 (GMT)

[redacted]

[redacted]	[redacted]	[redacted]	[redacted]	[redacted]
[redacted]	[redacted]	[redacted]	[redacted]	[redacted]

[redacted]

090177e19bd138e0\Approved\Approved On: 14-Nov-2022 20:57 (GMT)

[redacted]

[redacted]	[redacted]	[redacted]	[redacted]	[redacted]
[redacted]	[redacted]	[redacted]	[redacted]	[redacted]
[redacted]	[redacted]	[redacted]	[redacted]	[redacted]

090177e19bd138e0\Approved\Approved On: 14-Nov-2022 20:57 (GMT)

[redacted]

[redacted]	[redacted]	[redacted]	[redacted]	[redacted]
[redacted]	[redacted]	[redacted]	[redacted]	[redacted]
			[redacted]	[redacted]

[redacted]

090177e19bd138e0\Approved\Approved On: 14-Nov-2022 20:57 (GMT)

[redacted]

[redacted]	[redacted]
[redacted]	[redacted]
[redacted]	[redacted]
[redacted]	[redacted]
[redacted]	[redacted]

[redacted]

6.7. Intervention After the End of the Study

No intervention will be provided to study participants at the end of the study.

7. DISCONTINUATION OF STUDY INTERVENTION AND PARTICIPANT DISCONTINUATION/WITHDRAWAL

7.1. Discontinuation of Study Intervention

In rare instances, it may be necessary for a participant to permanently discontinue study intervention (definitive discontinuation). Reasons for definitive discontinuation of study intervention may include the following:

- Objective disease progression;
- Global deterioration of health status requiring discontinuation;
- Unacceptable toxicity;
- Pregnancy;
- Significant protocol violation;
- Lost to follow-up;
- Participant refused further treatment;
- Study terminated by sponsor;

090177e19bd138e0\Approved\Approved On: 14-Nov-2022 20:57 (GMT)

- Death;

Note that discontinuation of study intervention does not represent withdrawal from the study. If study intervention is definitively discontinued, the participant will remain in the study to be evaluated for safety follow-up and survival (Part 2 dose expansion). See the SoA for data to be collected at the time of discontinuation of study intervention and follow-up for any further evaluations that need to be completed.

In the event of discontinuation of study intervention, it must be documented on the appropriate CRF/in the medical records whether the participant is discontinuing further receipt of study intervention or also from study procedures, posttreatment study follow-up, and/or future collection of additional information.

See Appendix 13 (Section 10.13.3.4) for guidance for participants with active or presumed SARS-CoV2 infection.

ECG Changes

A participant who meets the QTc criteria below based on the average of triplicate ECG readings will be discontinued from the study intervention.

- QTcF >500 msec.
- Change from baseline: QTcF >60 msec.

If a clinically significant finding is identified (including, but not limited to, changes from baseline in QTcF after enrollment), the investigator or qualified designee will determine if the participant can continue in the study and if any change in participant management is needed. This review of the ECG printed at the time of collection must be documented. Any new clinically relevant finding should be reported as an AE.

7.1.1. Request to Continue Study Intervention

If the investigator feels the participant is still deriving benefit from the study intervention, the investigator should discuss with the sponsor to elect treatment for the participant at the same dose or 1 dose lower until such benefit no longer exists.

7.2. Participant Discontinuation/Withdrawal From the Study

A participant may withdraw from the study at any time at his/her own request. Reasons for discontinuation from the study may include:

- Completed study follow-up;
- Study terminated by sponsor;
- Lost to follow-up;
- Refused further follow-up;

090177e19bd138e0\Approved\Approved On: 14-Nov-2022 20:57 (GMT)

- Death.

At the time of discontinuing from the study, if possible, an end of treatment visit should be conducted. See the SoA for assessments to be collected at the time of study discontinuation and post-treatment follow-up and for any further evaluations that need to be completed.

If a participant withdraws from the study, he/she may request destruction of any remaining samples taken and not tested, and the investigator must document any such requests in the site study records and notify the sponsor accordingly.

If the participant withdraws from the study and also withdraws consent (see Section 7.2.1) for disclosure of future information, no further evaluations should be performed and no additional data should be collected. The sponsor may retain and continue to use any data collected before such withdrawal of consent.

Lack of completion of all or any of the withdrawal/early termination procedures will not be viewed as protocol deviations so long as the participant's safety was preserved.

7.2.1. Withdrawal of Consent

Participants who request to discontinue receipt of study intervention will remain in the study and must continue to be followed for protocol-specified follow-up procedures. The only exception to this is when a participant specifically withdraws consent for any further contact with him or her or persons previously authorized by the participant to provide this information. Participants should notify the investigator in writing of the decision to withdraw consent from future follow-up, whenever possible. The withdrawal of consent should be explained in detail in the medical records by the investigator, as to whether the withdrawal is only from further receipt of study intervention or also from study procedures and/or posttreatment study follow-up, and entered on the appropriate CRF page. In the event that vital status (whether the participant is alive or dead) is being measured, publicly available information should be used to determine vital status only as appropriately directed in accordance with local law.

7.3. Lost to Follow-up

A participant will be considered lost to follow-up if he or she repeatedly fails to return for scheduled visits and is unable to be contacted by the study site.

The following actions must be taken if a participant fails to return to the clinic for/attend a required study visit:

- The site must attempt to contact the participant and reschedule the missed visit as soon as possible and counsel the participant on the importance of maintaining the assigned visit schedule and ascertain whether or not the participant wishes to and/or should continue in the study.

- Before a participant is deemed lost to follow-up, the investigator or designee must make every effort to regain contact with the participant (where possible, 3 telephone

090177e19bd138e0\Approved\Approved On: 14-Nov-2022 20:57 (GMT)

calls and, if necessary, a certified letter to the participant's last known mailing address or local equivalent methods). These contact attempts should be documented in the participant's medical record.

- Should the participant continue to be unreachable, he/she will be considered to have withdrawn from the study.

8. STUDY ASSESSMENTS AND PROCEDURES

The investigator (or an appropriate delegate at the investigator site) must obtain a signed and dated ICD before performing any study specific procedures.

Study procedures and their timing are summarized in the SoA. Protocol waivers or exemptions are not allowed.

Safety issues should be discussed with the sponsor immediately upon occurrence or awareness to determine whether the participant should continue or discontinue study intervention.

Adherence to the study design requirements, including those specified in the SoA, is essential and required for study conduct.

All screening evaluations must be completed and reviewed to confirm that potential participants meet all eligibility criteria. The investigator will maintain a screening log to record details of all participants screened and to confirm eligibility or record reasons for screening failure, as applicable.

Procedures conducted as part of the participant's routine clinical management (eg, blood count) and obtained before signing of the ICD may be utilized for screening or baseline purposes provided the procedures met the protocol-specified criteria and were performed within the time frame defined in the SoA.

Every effort should be made to ensure that protocol-required tests and procedures are completed as described. However, it is anticipated that from time to time there may be circumstances outside the control of the investigator that may make it unfeasible to perform the test. In these cases, the investigator must take all steps necessary to ensure the safety and well-being of the participant. When a protocol-required test cannot be performed, the investigator will document the reason for the missed test and any corrective and preventive actions that he or she has taken to ensure that required processes are adhered to as soon as possible. The study team must be informed of these incidents in a timely manner.

For samples being collected and shipped, detailed collection, processing, storage, and shipment instructions and contact information will be provided to the investigator site prior to initiation of the study.

During the COVID-19 pandemic, please refer to Appendix 13 (Section 10.13) for safety and efficacy assessments.

090177e19bd138e0\Approved\Approved On: 14-Nov-2022 20:57 (GMT)

8.1. Efficacy Assessments

8.1.1. Tumor Response Assessments

Tumor assessments will include all known or suspected disease sites. Imaging will include contrast enhanced chest, abdomen and pelvis computed tomography or MRI scans; brain computed tomography or MRI scan for participants with known or suspected brain metastases; bone scan and/or bone x-rays for participants with known or suspected bone metastases. For participants with known computed tomography contrast allergy, a non contrast computed tomography of the chest with contrast enhanced abdominal and pelvic MRI can be used. The same imaging technique used to characterize each identified and reported lesion at baseline will be employed in the following tumor assessments.

Anti-tumor activity will be assessed through radiological tumor assessments conducted at baseline, during treatment as specified in the SoA, whenever disease progression is suspected (eg, symptomatic deterioration), and at the time of withdrawal from treatment (if not done in the previous 6 weeks). Assessment of response will be made using RECIST version 1.1 (see Section 10.11).

All participants' files and radiologic images must be available for source verification and for potential IRC review.

For Part 2 only, images will be stored at a third party vendor and may be used for exploratory analysis outside the scope of the study.

8.1.1.1. Determination of Radiographic Progression in Bone

For CRPC participants in PART 1A, radionuclide bone scans will be performed according to the SoA to document bone metastases. The assessment of tumor response will be based on RECIST 1.1 criteria (Section 10.11).

Assessment of metastatic bone disease will be done by whole-body radionuclide bone scan. A bone scan will assess 5 regions of the skeleton, including skull, thorax, spine, pelvis, and extremities. Radiographic progression for bone disease is defined as the appearance of 1 or more metastatic lesions on bone scan. Confirmation with a second imaging modality (plain film, CT, or MRI) will be required when bone lesions are found in a single region on the bone scan. Appearance of metastatic lesions in 2 or more of the 5 regions on a bone scan will not require confirmation with a second imaging modality. PET is not an evaluable imaging modality for this study.

8.1.1.2. Prostate-Specific Antigen

For CRPC participants in Part 1A, blood tumor biomarker PSA testing will be performed according to the SoA to monitor PSA levels.

090177e19bd138e0\Approved\Approved On: 14-Nov-2022 20:57 (GMT)

[redacted]

8.2. Safety Assessments

Planned time points for all safety assessments are provided in the SoA. Unscheduled clinical laboratory measurements may be obtained at any time during the study to assess any perceived safety issues.

Safety assessments will include collection of AEs, SAEs, vital signs and physical examination, ECG (12-lead), laboratory assessments, including pregnancy tests and verification of concomitant treatments.

8.2.1. Physical Examinations

Participants will have a physical examination to include weight, assessment of ECOG performance status and height; height will be measured at baseline only.

A complete physical examination will include more comprehensive assessments of the cardiovascular, respiratory, GI, genitourinary (if needed), musculoskeletal and neurological systems in addition to the assessments for the brief physical exam listed below.

A brief physical examination will include, at a minimum, assessments of the skin, lungs, cardiovascular system, and abdomen.

Findings should be recorded in the source documents, and any change from baseline considered by the investigation to be clinically significant should be recorded as an AE in the CRF.

Investigators should pay special attention to clinical signs related to previous serious illnesses.

8.2.2. Vital Signs

Oral temperature, pulse rate, respiratory rate, BP, and SpO_2 will be assessed.

BP and pulse rate measurements will be assessed in a sitting or semi-recumbent position (the same position should be maintained throughout the study) with a completely automated device. Manual techniques will be used only if an automated device is not available.

BP and pulse rate measurements should be preceded by at least 5 minutes of rest for the participant in a quiet setting without distractions (eg, television, cell phones).

Vital signs (to be taken before blood collection for laboratory tests) will consist of 1 pulse rate and 2 BP measurements (2 consecutive blood pressure readings will be recorded at intervals of at least 1 minute). The average of the 2 BP readings will be recorded on the CRF.

In addition, SpO_2 should be assessed by pulse oximeter as part of the vital signs.

8.2.3. Electrocardiograms

Standard 12-lead ECGs utilizing limb leads (with a 10-second rhythm strip) should be collected at times specified in the SoA section of this protocol using an ECG machine that automatically calculates the heart rate and measures PR, QT, and QTcF intervals and QRS

090177e19bd138e0\Approved\Approved On: 14-Nov-2022 20:57 (GMT)

complex. Alternative lead placement methodology using torso leads (eg, Mason-Likar) is not recommended given the potential risk of discrepancies with ECGs acquired using standard limb lead placement. All scheduled ECGs should be performed after the participant has rested quietly for at least 10 minutes in a supine position.

At each time point (see the SoA), 3 consecutive ECGs will be performed at approximately 2 minutes apart to determine the mean QTcF interval.

To ensure safety of the participants, a qualified individual at the investigator site will make comparisons to baseline measurements. Additional ECG monitoring will occur if a) the mean value from the triplicate measurements for any postdose QTcF interval is increased by ≥60 msec from the baseline **and** is >450 msec; or b) an absolute QTcF value is ≥500 msec for any scheduled ECG. If either of these conditions occurs, then a single ECG measurement must be repeated at least hourly until QTcF values from 2 successive ECGs fall below the threshold value that triggered the repeat measurement. In addition, if verified QTcF values continue to exceed the criteria above, immediate correction for reversible causes including electrolyte abnormalities, hypoxia and concomitant medications for drugs with the potential to prolong the QTcF interval should be performed.

If the QTcF interval reverts to less than the threshold criteria listed above, and in the judgment of the investigator(s) and sponsor, it is determined that the cause(s) of QTcF prolongation is something other than study intervention, treatment may be continued with regular ECG monitoring. If in that timeframe the QTcF intervals rise above the threshold values, the study intervention will be held until the QTcF interval decreases to below the threshold values. Participants will then restart the study intervention at the next lowest dose level. If the QTcF interval has still not decreased to <480 msec after 2 weeks, or if at any time a participant has a QTcF interval >515 msec or becomes symptomatic, the participant will be removed from the study. Additional triplicate ECGs may be performed as clinically indicated.

In some cases, it may be appropriate to repeat abnormal ECGs to rule out improper lead placement as contributing to the ECG abnormality. It is important that leads be placed in the same positions each time in order to achieve precise ECG recordings. If a machine-read QTcF value is prolonged, as defined above, repeat measurements may not be necessary if a qualified medical provider's interpretation determines that the QTcF values are in the acceptable range.

If a participant experiences a cardiac or neurologic AE (specifically syncope, dizziness, seizures, or stroke), an ECG (triplicate) should be obtained at the time of the event.

ECG values of potential clinical concern are listed in Appendix 7 (Section 10.7).

8.2.4. Clinical Safety Laboratory Assessments

See Appendix 2, (Section 10.2) for the list of clinical safety laboratory tests to be performed and the SoA for the timing and frequency. All protocol required- laboratory assessments, as defined in Appendix 2, (Section 10.2), must be conducted in accordance with the laboratory

090177e19bd138e0\Approved\Approved On: 14-Nov-2022 20:57 (GMT)

manual and the SoA. Unscheduled clinical laboratory measurements may be obtained at any time during the study to assess any perceived safety issues.

The investigator must review the laboratory report, document this review, and record any clinically relevant changes occurring during the study in the AE section of the CRF. Clinically significant abnormal laboratory findings are those which are not associated with the underlying disease, unless judged by the investigator to be more severe than expected for the participant's condition.

All laboratory tests with values considered clinically significantly abnormal during participation in the study or within 28 after the last dose of study intervention should be repeated until the values return to normal or baseline or are no longer considered clinically significant by the investigator or medical monitor.

If such values do not return to normal/baseline within a period of time judged reasonable by the investigator, the etiology should be identified and the sponsor notified.

See Appendix 6 (Section 10.6) for suggested actions and follow-up assessments in the event of potential drug-induced liver injury.

8.2.5. Pregnancy Testing

Pregnancy tests may be urine or serum tests, but must have a sensitivity of at least 25 mIU/mL. Pregnancy tests will be performed in WOCBP at the times listed in the SoA. Following a negative pregnancy test result at screening, appropriate contraception must be commenced and a second negative pregnancy test result will be required at the baseline visit prior to the participant receiving PF-07248144. Pregnancy tests will also be done whenever 1 menstrual cycle is missed during the active treatment period (or when potential pregnancy is otherwise suspected) and at the end of the study. Pregnancy tests may also be repeated if requested by IRBs/Ecs or if required by local regulations. If a urine test cannot be confirmed as negative (eg, an ambiguous result), a serum pregnancy test is required. In such cases, the participant must be if the serum pregnancy result is positive.

8.3. Adverse Events and Serious Adverse Events

The definitions of an AE and an SAE can be found in Appendix 3 (Section 10.3).

AEs will be reported by the participant (or, when appropriate, by a caregiver, surrogate, or the participant's legally authorized representative).

The investigator and any qualified designees are responsible for detecting, documenting, and recording events that meet the definition of an AE or SAE and remain responsible to pursue and obtain adequate information both to determine the outcome and to assess whether the event meets the criteria for classification as an SAE or caused the participant to discontinue the study intervention (see Section 7.1).

090177e19bd138e0\Approved\Approved On: 14-Nov-2022 20:57 (GMT)

Each participant will be questioned about the occurrence of AEs in a nonleading manner. In addition, the investigator may be requested by Pfizer Safety to obtain specific follow-up information in an expedited fashion.

8.3.1. Time Period and Frequency for Collecting AE and SAE Information

The time period for actively eliciting and collecting AEs and SAEs ("active collection period") for each participant begins from the time the participant provides informed consent, which is obtained before the participant's participation in the study (ie, before undergoing any study-related procedure and/or receiving study intervention), through and including a minimum of 28 calendar days, except as indicated below, after the last administration of the study intervention.

During the long-term follow-up period in this study for survival, only SAEs will be actively elicited and collected after completion of the active collection period described above. The SAEs identified during long-term follow-up will be reported to Pfizer Safety on the CT SAE Report Form only if considered reasonably related to the study intervention.

Follow-up by the investigator continues throughout and after the active collection period and until the AE or SAE or its sequelae resolve or stabilize at a level acceptable to the investigator and Pfizer concurs with that assessment.

For participants who are screen failures, the active collection period ends when screen failure status is determined.

If the participant withdraws from the study and also withdraws consent for the collection of future information, the active collection period ends when consent is withdrawn.

If a participant definitively discontinues or temporarily discontinues study intervention because of an AE or SAE, the AE or SAE must be recorded on the CRF and the SAE reported using the CT SAE Report Form.

Investigators are not obligated to actively seek AEs or SAEs after the participant has concluded study participation. However, if the investigator learns of any SAE, including a death, at any time after a participant has completed the study, and he/she considers the event to be reasonably related to the study intervention, the investigator must promptly report the SAE to Pfizer using the CT SAE Report Form.

8.3.1.1. Reporting SAEs to Pfizer Safety

All SAEs occurring in a participant during the active collection period as described in Section 8.3.1 are reported to Pfizer Safety on the CT SAE Report Form immediately upon awareness and under no circumstance should this exceed 24 hours, as indicated in Appendix 3. The investigator will submit any updated SAE data to the sponsor within 24 hours of it being available.

090177e19bd138e0\Approved\Approved On: 14-Nov-2022 20:57 (GMT)

If a participant begins a new anticancer therapy, SAEs occurring during the above-indicated active collection period must still be reported to Pfizer Safety irrespective of any intervening treatment.

8.3.1.2. Recording Nonserious AEs and SAEs on the CRF

All nonserious AEs and SAEs occurring in a participant during the active collection period, which begins after obtaining informed consent as described in Section 8.3.1 will be recorded on the AE section of the CRF.

The investigator is to record on the CRF all directly observed and all spontaneously reported AEs and SAEs reported by the participant.

If a participant begins a new anticancer therapy, the recording period for nonserious AEs ends at the time the new treatment is started; however, SAEs must continue to be recorded on the CRF during the above-indicated active collection period. Note that a switch to a commercially available version of the study intervention is considered as a new anticancer therapy for the purposes of SAE reporting.

8.3.2. Method of Detecting AEs and SAEs

The method of recording, evaluating, and assessing causality of AEs and SAEs and the procedures for completing and transmitting SAE reports are provided in Appendix 3.

Care will be taken not to introduce bias when detecting AEs and/or SAEs. Open-ended and nonleading verbal questioning of the participant is the preferred method to inquire about AE occurrences.

8.3.3. Follow-up of AEs and SAEs

After the initial AE/SAE report, the investigator is required to proactively follow each participant at subsequent visits/contacts. For each event, the investigator must pursue and obtain adequate information until resolution, stabilization, the event is otherwise explained, or the participant is lost to follow-up (as defined in Section 7.3).

In general, follow-up information will include a description of the event in sufficient detail to allow for a complete medical assessment of the case and independent determination of possible causality. Any information relevant to the event, such as concomitant medications and illnesses, must be provided. In the case of a participant death, a summary of available autopsy findings must be submitted as soon as possible to Pfizer Safety.

Further information on follow-up procedures is given in Appendix 3 (Section 10.3).

8.3.4. Regulatory Reporting Requirements for SAEs

Prompt notification by the investigator to the sponsor of an SAE is essential so that legal obligations and ethical responsibilities towards the safety of participants and the safety of a study intervention under clinical investigation are met.

090177e19bd138e0\Approved\Approved On: 14-Nov-2022 20:57 (GMT)

The sponsor has a legal responsibility to notify both the local regulatory authority and other regulatory agencies about the safety of a study intervention under clinical investigation. The sponsor will comply with country-specific regulatory requirements relating to safety reporting to the regulatory authority, IRBs/Ecs, and investigators.

Investigator safety reports must be prepared for SUSARs according to local regulatory requirements and sponsor policy and forwarded to investigators as necessary.

An investigator who receives SUSARs or other specific safety information (eg, summary or listing of SAEs) from the sponsor will review and then file it along with the SRSD(s) for the study and will notify the IRB/EC, if appropriate according to local requirements.

8.3.5. Exposure During Pregnancy or Breastfeeding, and Occupational Exposure

Exposure to the study intervention under study during pregnancy or breastfeeding and occupational exposure are reportable to Pfizer Safety within 24 hours of investigator awareness.

8.3.5.1. Exposure During Pregnancy

An EDP occurs if:

- A female participant is found to be pregnant while receiving or after discontinuing study intervention.
- A male participant who is receiving or has discontinued study intervention exposes a female partner prior to or around the time of conception.
- A female is found to be pregnant while being exposed or having been exposed to study intervention due to environmental exposure. Below are examples of environmental exposure during pregnancy:
    - A female family member or healthcare provider reports that she is pregnant after having been exposed to the study intervention by ingestion, inhalation, or skin contact.
    - A male family member or healthcare provider who has been exposed to the study intervention by ingestion, inhalation, or skin contact then exposes his female partner prior to or around the time of conception.

The investigator must report EDP to Pfizer Safety within 24 hours of the investigator's awareness, irrespective of whether an SAE has occurred. The initial information submitted should include the anticipated date of delivery (see below for information related to termination of pregnancy).

- If EDP occurs in a participant or a participant's partner, the investigator must report this information to Pfizer Safety on the CT SAE Report Form and an EDP Supplemental Form, regardless of whether an SAE has occurred. Details of the

090177e19bd138e0\Approved\Approved On: 14-Nov-2022 20:57 (GMT)

pregnancy will be collected after the start of study intervention and until a minimum of 28 calendar days after the last administration of study intervention or until study completion or withdrawal, whichever is longer.

- If EDP occurs in the setting of environmental exposure, the investigator must report information to Pfizer Safety using the CT SAE Report Form and EDP Supplemental Form. Since the exposure information does not pertain to the participant enrolled in the study, the information is not recorded on a CRF; however, a copy of the completed CT SAE Report Form is maintained in the investigator site file.

Follow-up is conducted to obtain general information on the pregnancy and its outcome for all EDP reports with an unknown outcome. The investigator will follow the pregnancy until completion (or until pregnancy termination) and notify Pfizer Safety of the outcome as a follow-up to the initial EDP Supplemental Form. In the case of a live birth, the structural integrity of the neonate can be assessed at the time of birth. In the event of a termination, the reason(s) for termination should be specified and, if clinically possible, the structural integrity of the terminated fetus should be assessed by gross visual inspection (unless preprocedure test findings are conclusive for a congenital anomaly and the findings are reported).

Abnormal pregnancy outcomes are considered SAEs. If the outcome of the pregnancy meets the criteria for an SAE (ie, ectopic pregnancy, spontaneous abortion, intrauterine fetal demise, neonatal death, or congenital anomaly in a live-born baby, a terminated fetus, an intrauterine fetal demise, or a neonatal death), the investigator should follow the procedures for reporting SAEs. Additional information about pregnancy outcomes that are reported to Pfizer Safety as SAEs are as follows:

- Spontaneous abortion including miscarriage and missed abortion;

- Neonatal deaths that occur within 1 month of birth should be reported, without regard to causality, as SAEs. In addition, infant deaths after 1 month should be reported as SAEs when the investigator assesses the infant death as related or possibly related to exposure to the study intervention.

Additional information regarding the EDP may be requested by the sponsor. Further follow-up of birth outcomes will be handled on a case-by-case basis (eg, follow-up on preterm infants to identify developmental delays). In the case of paternal exposure, the investigator will provide the participant with the Pregnant Partner Release of Information Form to deliver to his partner. The investigator must document in the source documents that the participant was given the Pregnant Partner Release of Information Form to provide to his partner.

090177e19bd138e0\Approved\Approved On: 14-Nov-2022 20:57 (GMT)

8.3.5.2. Exposure During Breastfeeding

An exposure during breastfeeding occurs if:

- A female participant is found to be breastfeeding while receiving or after discontinuing study intervention.
- A female is found to be breastfeeding while being exposed or having been exposed to study intervention (ie, environmental exposure). An example of environmental exposure during breastfeeding is a female family member or healthcare provider who reports that she is breastfeeding after having been exposed to the study intervention by inhalation or skin contact.

The investigator must report exposure during breastfeeding to Pfizer Safety within 24 hours of the investigator's awareness, irrespective of whether an SAE has occurred. The information must be reported using the CT SAE Report Form. When exposure during breastfeeding occurs in the setting of environmental exposure, the exposure information does not pertain to the participant enrolled in the study, so the information is not recorded on a CRF. However, a copy of the completed CT SAE Report Form is maintained in the investigator site file.

An exposure during breastfeeding report is not created when a Pfizer drug specifically approved for use in breastfeeding women (eg, vitamins) is administered in accord with authorized use. However, if the infant experiences an SAE associated with such a drug, the SAE is reported together with the exposure during breastfeeding.

8.3.5.3. Occupational Exposure

An occupational exposure occurs when a person receives unplanned direct contact with the study intervention, which may or may not lead to the occurrence of an AE. Such persons may include healthcare providers, family members, and other roles that are involved in the trial participant's care.

The investigator must report occupational exposure to Pfizer Safety within 24 hours of the investigator's awareness regardless of whether there is an associated SAE. The information must be reported using the CT SAE Report Form. Since the information does not pertain to a participant enrolled in the study, the information is not recorded on a CRF; however, a copy of the completed CT SAE Report Form is maintained in the investigator site file.

8.3.6. Cardiovascular and Death Events

Not applicable.

090177e19bd138e0\Approved\Approved On: 14-Nov-2022 20:57 (GMT)

8.3.7. Disease-Related Events and/or Disease Related Outcomes Not Qualifying as AEs or SAEs

The following DREs are common in participants with advanced solids tumors and can be serious/life threatening:

- Disease progression.

Because these events are typically associated with the disease under study, they will not be reported according to the standard process for expedited reporting of SAEs even though the event may meet the definition of an SAE. These events will be recorded on the corresponding CRF page in the participant's CRF within the appropriate time frame.

NOTE: However, if either of the following conditions applies, then the event must be recorded and reported as an SAE (instead of a DRE):

- The event is, in the investigator's opinion, of greater intensity, frequency, or duration than expected for the individual participant.

OR

- The investigator considers that there is a reasonable possibility that the event was related to study intervention.

8.3.8. Adverse Events of Special Interest

Not applicable.

8.3.8.1. Lack of Efficacy

Lack of efficacy is reportable to Pfizer Safety only if associated with an SAE.

8.3.9. Medical Device Deficiencies

Not applicable.

8.3.10. Medication Errors

Medication errors may result from the administration or consumption of the study intervention by the wrong participant, or at the wrong time, or at the wrong dosage strength.

Safety Event	Recorded on the CRF	Reported on the CT SAE Report Form to Pfizer Safety Within 24 Hours of Awareness
Medication errors	All (regardless of whether associated with an AE)	Only if associated with an SAE

090177e19bd138e0\Approved\Approved On: 14-Nov-2022 20:57 (GMT)

Medication errors include:

- Medication errors involving participant exposure to the study intervention;
- Potential medication errors or uses outside of what is foreseen in the protocol that do or do not involve the study participant.

Such medication errors occurring to a study participant are to be captured on the medication error page of the CRF, which is a specific version of the AE page.

In the event of a medication dosing error, the sponsor should be notified within 24 hours.

Whether or not the medication error is accompanied by an AE, as determined by the investigator, the medication error is recorded on the medication error page of the CRF and, if applicable, any associated AE(s), serious and nonserious, are recorded on the AE page of the CRF.

Medication errors should be reported to Pfizer Safety within 24 hours on a CT SAE Report Form **only when associated with an SAE.**

Other examples include, but are not limited to:

- The administration of expired study intervention;
- The administration of an incorrect study intervention;
- The administration of an incorrect dosage;
- The administration of study intervention that has undergone temperature excursion from the specified storage range, unless it is determined by the sponsor that the study intervention under question is acceptable for use.

8.4. Treatment of Overdose

For this study, any dose of either study drug (s) greater than the prescribed regimen of 1 dose within a 24 hour time period (±4 hours) will be considered an overdose.

The Sponsor does not recommend specific treatment for an overdose.

In the event of an overdose, the investigator/treating physician should:

1. Contact the medical monitor within 24 hours.
2. Closely monitor the participant for any AEs/SAEs and laboratory abnormalities for at least 5 half-lives or 28 calendar days after the overdose of study intervention (whichever is longer).

090177e19bd138e0\Approved\Approved On: 14-Nov-2022 20:57 (GMT)

3. Document the quantity of the excess dose as well as the duration of the overdose in the CRF.

4. Overdose is reportable to Safety **only when associated with an SAE.**

5. Obtain a blood sample for PK analysis within 7 days from the date of the last dose of study intervention if requested by the medical monitor (determined on a case-by-case basis).

Decisions regarding dose interruptions or modifications will be made by the investigator in consultation with the medical monitor based on the clinical evaluation of the participant.

8.5. Pharmacokinetics

8.5.1. Plasma for Pharmacokinetic Analysis of PF-07248144, [redacted]

In Parts 1A, 1B, 2A, and 2B, blood samples (2 mL) to provide approximately 1 mL of plasma for PF=-07248144 PK analysis will be collected into appropriately labeled tubes containing EDTA (anticoagulant) as outlined in the SoA. [redacted] The PK sampling schedule may be modified based on emerging PK data. On days when participants have clinical visits where PK assessments are to be obtained, the dose(s) of study intervention (PF-07248144, [redacted] fulvestrant, [redacted] as applicable) should be held (NOT taken) prior to the study visit. On those days, the study intervention dose(s) can be taken after the study procedures required immediately prior to the study intervention dose(s) have been performed.

In addition to samples collected at the scheduled times, an additional blood sample should be collected from patients experiencing unexpected and/or serious AEs and the date and time of blood sample collection and of last dosing prior to PK collection documented on the CRF.

Where noted in the SoA, blood samples for PF-07248144, [redacted]) PK analysis will be collected at approximately the same time as other assessments such as PD samples, and ECGs (first ECG then PK collection), wherever possible.

All efforts will be made to obtain the PK samples at the scheduled nominal time relative to dosing. However, samples collected within the visit windows specified in Table 2 (Pharmacokinetic Sampling, Pharmacodynamic/other Biomarker Sampling, and ECG Assessments (for Parts 1A, 1B, [redacted] and Table 5 (Pharmacokinetic Sampling Schedule in Participants in the food effect subset of Part 2A [Monotherapy Dose Expansion]) will be acceptable, and the exact time of the sample collection will be noted on the CRF. If a

090177e19bd138e0\Approved\Approved On: 14-Nov-2022 20:57 (GMT)

scheduled blood sample collection cannot be completed for any reason, the missed sample time may be re scheduled with agreement of the clinical investigator, patient, and sponsor.

PK samples will be assayed for PF-07248144, [redacted] using a validated analytical method in compliance with Pfizer/vendor SOPs.

The PK samples must be processed and shipped as indicated in the instructions provided to the investigator site to maintain sample integrity. Any deviations from the PK sample handling procedure (eg, sample collection and processing steps, interim storage or shipping conditions), including any actions taken, must be documented and reported to the sponsor. On a case-by-case basis, the sponsor may make a determination as to whether sample integrity has been compromised. Any deviation from the specified sample handling procedure resulting in compromised sample integrity will be considered a protocol deviation.

As part of understanding the PK of the investigational product, samples may be used for the evaluation of the bioanalytical method, as well as for other internal exploratory purposes. These data will not be included in the CSR.

8.5.2. Urine for Analysis of PF-07248144 Concentrations and Metabolite Profiling

For participants in Part 2A dose expansion, urine samples will be collected at screening and for 24 hours after PF-07248144 morning dosing on Cycle 1 Day 15 to measure urinary PF-07248144 concentrations, and thereby determine the renal elimination of PF-07248144 from the body. These urine samples for PK concentrations and metabolite profiling will be collected in approximately 6 participants in Part 2A expansion phase. Urine collection for PF-07248144 PK and metabolite profiling will be collected from the same subset of participants from whom blood samples for metabolite profiling are collected in Part 2A (see Section 8.5.3).

Participants will empty their bladder just prior to morning dosing on Cycle 1 Day 15. The details of sampling of urine for PK and metabolites are provided in the SoA.

At the end of each urine collection period, the total volume will be measured and recorded. Voided urine should be collected in an amber container and protected from direct light. The urine will then be mixed thoroughly and a 10 mL aliquot will be withdrawn for the potential measurement of drug concentrations and a separate 10 mL aliquot will be withdrawn for the potential metabolite profiling. The sample must be processed and shipped to 2 separate labs as indicated in the instructions provided to the investigator site.

The urine samples collected for PF-07248144 concentrations will be assayed using a validated analytical method in compliance with Pfizer/vendor SOPs.

As part of understanding the PK of the investigational product, samples may be used for the evaluation of the bioanalytical method, as well as for other internal exploratory purposes. These data will not be included in the CSR.

090177e19bd138e0\Approved\Approved On: 14-Nov-2022 20:57 (GMT)

8.5.3. Blood for Metabolite Profiling of PF-07248144

For participants in Part 2A dose expansion, blood samples for metabolite profiling will be collected at Screening (or prior to first dose administration) and on Cycle 1 Day 15 at all time points collected for PK analysis (see SoA). Two (2) mL of blood will be collected at each time point to obtain 1 mL of plasma. These blood samples for metabolite profiling will be collected in approximately 6 participants in Part 2A expansion phase in the same subset of participants from whom urine samples are collected in Part 2A (see Section 8.5.2).

Once the metabolite profiling samples have been analyzed and the report completed, the samples will be disposed of. These data will not be included in the CSR.

8.6. Pharmacodynamics

PD parameters will be evaluated in this study. See Section 8.8 Biomarkers.

Blood and/or tumor samples will be collected for measurement of PD markers of PF-07248144 [redacted] including H3K23Ac, [redacted] at the time points in the SoA.

The actual times may change, but the number of samples will remain the same. All efforts will be made to obtain the samples at the exact nominal time relative to dosing. Collection of samples within the sampling time window specified in the Pharmacokinetic and Biomarker Sampling table (see Section 1.3) (collection of samples within 10% of the nominal time relative to dosing (eg, within 6 minutes of a 60-minute sample) will not be captured as a protocol deviation, as long as the exact time of the collection is noted on the source document and the CRF. If a scheduled blood/tumor sample collection cannot be completed for any reason, the missed sample time may be re-scheduled with agreement of the clinical investigator, patient, and sponsor.

As part of understanding the PD of the study intervention, samples may be used for evaluation of the bioanalytical method, as well as for other internal exploratory purposes.

Samples will be tested using analytically validated methods in compliance with applicable SOPs. These data will be used for internal exploratory purposes and will not be included in the CSR.

The PD samples must be processed and shipped as indicated in the instructions provided to the investigator site to maintain sample integrity. Any deviations from the PD sample handling procedure (eg, sample collection and processing steps, interim storage, or shipping conditions), including any actions taken, must be documented and reported to the sponsor. On a case-by-case basis, the sponsor may make a determination as to whether sample integrity has been compromised.

8.7. Genetics

See Appendix 8 (Section 10.8.1.4) for information regarding genetic research for Japan.

090177e19bd138e0\Approved\Approved On: 14-Nov-2022 20:57 (GMT)

8.7.1. Specified Genetics

A 4-mL blood PGx sample for DNA isolation will be collected into plastic K2-EDTA tubes, as defined in the schedule of activities. DNA samples will be analyzed for the purpose of assessing the impact of allelic variants of drug metabolizing enzymes and transporters. Additionally, these samples may also be used for retrospective evaluation of additional genetic variants associated with variation in PK, biomarker response, or to explore AEs should these be observed. Samples will be retained for a period of up to 3 years after regulatory approval. In the event of DNA extraction failure, a replacement genetic blood sample may be requested from the participant.

See Section 10.5 for additional information regarding genetic research. Details on processes for collection and shipment of these samples can be found in sponsor-identified study-specific central laboratory manual.

The PGx sample must be processed and shipped as indicated in the instructions provided to the investigator site, to maintain sample integrity. Any deviations from the PGx processing steps, including any actions taken, must be documented and reported to the sponsor. On a case-by-case basis, the sponsor may make a determination as to whether sample integrity has been compromised. Any sample deemed outside of established stability, or of questionable integrity, will be considered a protocol deviation.

As part of further understanding the biological response to study intervention, samples may be used for evaluation of other related genotyping as well as development and validation of bioanalytical methods.

8.7.2. Banked Biospecimens for Genetics

A 4-mL blood sample optimized for DNA isolation will be collected as local regulations and IRBs/Ecs allow.

Banked Biospecimens may be used for research related to the study intervention(s) and cancer. Genes and other analytes (eg, proteins, RNA, nondrug metabolites) may be studied using the banked samples.

See Section 10.5 for additional information regarding genetic research. Details on processes for collection and shipment of these samples can be found in the laboratory manual.

8.8. Biomarkers

Biomarker samples will be collected from all relevant participants under consent [redacted]

090177e19bd138e0\Approved\Approved On: 14-Nov-2022 20:57 (GMT)

[redacted] s well as hypothesis-free approaches by molecular profiling using baseline and/or progression tumor and/or plasma samples may also be performed.

Biospecimens collected for pharmacodynamic and other biomarker assessments may include peripheral blood, plasma, and tumor tissues, and may be used to analyze DNA, RNA, proteins, or metabolic biomarkers, for achieving planned biomarker objectives. Refer to the SoA for sample collection time points and the laboratory manual for sample processing and shipping. The following biospecimen types are planned to be collected in support of study objectives. Additional biospecimens collected over the course of participant disease management may be submitted for biomarker analyses. The biomarker sampling schedule may be modified based on emerging PK and/or PD data.

[redacted]

A most recent archived tumor specimen from a recurrent tumor or distant metastasis other than bone is required and collected for all participants. If the archived tumor is not available or not sufficient, a de novo (fresh) baseline biopsy (any time before C1D1 treatment once all other general screening procedures have been completed and the participant is eligible for study treatment) is mandatory for all participants. In the event that the participant has bone disease only, or it poses a safety risk to the participant, in the opinion of the investigator and in consultation with the sponsor prior to enrollment, original diagnostic tumor tissue could be used for biomarker analyses. Consultation with the Sponsor prior to participant enrollment is required if this biopsy sample cannot be provided~~.~~

These tumor specimens will be used to analyze candidate nucleic acid and protein biomarkers for their ability to identify those participants who are most likely to benefit from treatment with the study intervention(s). [redacted].

De novo (fresh) tumor paired biopsies obtained pre- and on-treatment are highly encouraged (optional) for participants in the dose escalation cohorts (Part 1A, Part 1B, [redacted], however are mandatory for at least 5 participants in every part of 2A, 2B, [redacted] Paired de novo tumor biopsies will be used to assess modulation of the levels of markers such as H3K23Ac, [redacted] as well as gene expression profiling changes after PF-07248144 treatment. [redacted]. Both baseline and on-treatment biopsies need to be collected if the participant has consented. On-treatment biopsy samples are to be collected on Cycle 1 Day 15 (+/- 2 days), 4 ±2 hours post PF-07248144 dosing. If for safety reasons, the biopsy cannot be collected at this timepoint, it should be collected as soon as medically safe and feasible. In the event of any drug dose interruptions, biopsies should be taken 2 weeks after the patient resumes dosing. Paired tumor biopsies should preferably be taken from the same non-target lesion and from a lesion that has not been previously irradiated. Tumor tissue from cytologic sampling (eg, fine needle aspiration, including FFPE cell pellet material) or bone specimen, is not adequate and

[redacted]

090177e19bd138e0\Approved\Approved On: 14-Nov-2022 20:57 (GMT)

should not be submitted. The EOT biopsy is optional but - encouraged. Additional information on tissue collection procedures can be found in the Laboratory Manual.

[redacted]

Peripheral blood samples will be collected from all participants at the time points outlined in the SoA for measurement of the [redacted] correlate with doses and PK. [redacted] Details regarding the collection, processing, storage and shipping of these samples will be provided in the Laboratory Manual.

In addition, blood samples optimized for [redacted] analyses will be collected from all participants at the time points outlined in the SoA. [redacted] d can be correlated with patient response. Examples of [redacted]

Additional analyses may be warranted based on emerging data.

8.9. Immunogenicity Assessments

Immunogenicity assessments are not included in this study.

8.10. Health Economics

Health economics/medical resource utilization and health economics parameters are not evaluated in this study.

9. STATISTICAL CONSIDERATIONS

Detailed methodology for summary and statistical analyses of the data collected in this study is outlined here and further detailed in a SAP, which will be maintained by the sponsor.

The data will be summarized with respect to demographic and baseline characteristics, efficacy observations and measurements, safety observations and measurements, pharmacokinetic and biomarker measurements.

9.1. Statistical Hypotheses

There will be no formal hypothesis testing in this study.

9.2. Sample Size Determination

The total number of participants is estimated to be approximately 145 to 190.

090177e19bd138e0\Approved\Approved On: 14-Nov-2022 20:57 (GMT)

9.2.1. Part 1 Dose Escalation

Approximately 70 participants will be enrolled in Part 1 including 25 to 30 participants in Part 1A, 6 to 9 participants in Part 1B, [redacted].

The actual number of participants enrolled will depend on the tolerability of PF-07248144 and the number of dose levels required to identify the MTD/RDE as monotherapy or in combination.

9.2.2. Part 2 Dose Expansion

Part 2A: Approximately 30 participants will be enrolled in Part 2A.

Assuming a non-informative prior (ie, Jeffrey's prior) if ≥4 out of 30 participants have tumor response, this would translate into a posterior probability (Beta Binomial) of more than 0.746 that the true response rate is not inferior to target response rate of 10%. Similarly, if ≤2 out of 30 participants have tumor response, this would translate into a posterior probability of more than 0.70 that the true response rate is not superior to the benchmark rate of 10%.

Part 2B: Approximately 30 participants will be enrolled in Part 2B.

Assuming a non-informative prior (ie, Jeffrey's prior) if ≥6 out of 30 participants have tumor response, this would translate into a posterior probability (Beta Binomial) of more than 0.786 that the true response rate is not inferior to target response rate of 15%. Similarly, if ≤2 out of 30 participants have tumor response, this would translate into a posterior probability of more than 0.70 that the true response rate is not superior to the benchmark rate of 10%[29-31].

[redacted]

[redacted]

[redacted]

9.3. Analysis Sets

1. Full analysis set.

   The full analysis set includes all enrolled participants.

2. Safety analysis set.

090177e19bd138e0\Approved\Approved On: 14-Nov-2022 20:57 (GMT)

The safety analysis set includes all enrolled participants who receive at least 1 dose of study intervention. Unless otherwise specified the safety analysis set will be the default analysis set used for all analyses.

3. Per protocol analysis set (evaluable for MTD).

   The per protocol analysis set includes all enrolled participants who had at least 1dose of study treatment and either experienced DLT or do not have major treatment deviations during the DLT observation period.

4. mITT Population.

   The mITT is the analysis population that will follow the ITT principle and include participants receiving at least 1 dose of study medication with baseline assessment and at least 1 post baseline assessment, disease progression, or death before the first tumor assessment. The mITT population may be used for interim analysis and conference presentations when the study is still ongoing.

5. PK analysis sets.

   The PK parameter analysis population is defined as all enrolled participants treated who do not have protocol deviations influencing PK assessment, and have sufficient information to estimate at least 1 of the PK parameters of interest.

   The PK concentration population is defined as all enrolled participants who are treated and have at least 1 analyte concentration.

6. Response Evaluable Set.

   The response evaluable population will include all participants who received at least 1 dose of study treatment and had baseline disease assessment and at least 1 post baseline disease assessment.

7. PD/Biomarker analysis set(s).

   The PD/Biomarker analysis population is defined as all enrolled participants with at least 1 of the PD/Biomarkers evaluated at pre and/or post dose.

9.4. Statistical Analyses

The SAP will be developed and finalized before any analyses are performed and will describe the analyses and procedures for accounting for missing, unused, and spurious data. This section is a summary of the planned statistical analyses of the primary and secondary endpoints.

9.4.1. Maximum Tolerated Dose Determination

Determination of MTD will be performed using a Per protocol analysis set (evaluable for MTD).

090177e19bd138e0\Approved\Approved On: 14-Nov-2022 20:57 (GMT)

Bayesian adaptive approach:

The dose escalation in Part 1A, and dose finding in Part 1B [redacted] of the study will be guided by a Bayesian analysis of Cycle 1 DLT data for PF-07248144 as a monotherapy (Part 1A) or in combination (Part 1B, [redacted]). A traditional 2-parameter BLRM will be used to model the dose/DLT relationship of PF-07248144 monotherapy. A more complex BLRM model specifically designed for combinations will be used to model the dose/DLT relationship of PF-07248144 given in combination with fulvestrant, [redacted]

Using DLT data at all tested dose levels and pre specified prior distribution of model parameters, the posterior distribution for probability of having a DLT will be calculated for all dose levels.

Assessment of participant risk:

After each cohort of participants, the posterior distribution for the risk of DLT for new participants at different doses of interest for PF-07248144 will be evaluated. The posterior distributions will be summarized to provide the posterior probability that the risk of DLT lies within the following intervals:

Under-dosing:	[0, 0.16]
Targeted dosing:	[0.16, 0.33]
Overdosing:	[0.33, 1]

The EWOC principle:

Dosing decisions are guided by the escalation with overdose control principal. A dose may only be used for newly enrolled participants if the risk of excessive toxicity at that dose is less than 25%.

Prior distributions:

Weakly informative prior distributions based on pre-clinical/expert opinion information will be chosen for the logistic parameters for prior distribution in Part 1A, see Appendix 9 (Section 10.9).

A MAP approach might be used to derive the prior distribution for model parameters used in Part 1B, [redacted] based on the data collected in Part 1A and DLT data collected in clinical studies for fulvestrant, [redacted].

The MAP prior for the logistic model parameters for this study is the conditional distribution of the parameters given the historical data. MAP priors are derived from hierarchical models, which take into account possible differences between the studies. A full description of the application of the MAP approach to derive the prior distributions of the model parameters is given in a Technical Supplement to Appendix 9 (Section 10.9).

090177e19bd138e0\Approved\Approved On: 14-Nov-2022 20:57 (GMT)

In case of change of the dosing regimen, DLT data accumulated during the dose escalation with the original regimen may be used to form a prior for further BLRM analysis. Details about derivation of this prior using MAP approach is given in a Technical Supplement to Appendix 9 (Section 10.9).

Starting dose:

The starting dose of Part 1A is 8 mg QD. For this dose the prior risk of overdosing is 10.1%, which satisfies the EWOC criterion. A full assessment of the prior risk to participants is given in Appendix 9 (Section 10.9).

[redacted]

Sensitivity Analysis:

To mitigate the risk of dichotomizing and misclassifying DLTs, a sensitivity analysis that uses weighted DLT/AE data (in equivocal cases) into the BLRM model estimation will also be performed. If all the investigators and the sponsor agree on the equivocal DLT/AE data, the DLT weighting approach could be the primary dose escalation method. See Appendix 9 (Section 10.9) for more details.

9.4.1.1. Stopping Criteria

The trial will be stopped when the following criteria are met:

- At least 6 participants have been treated at the recommended MTD/RDE.
- The dose $\tilde{d}$ satisfies one of the following conditions:
  - The probability of target toxicity at dose $\tilde{d}$ exceeds 50%, ie, $\Pr(0.16 \leq \pi_{\tilde{d}} < 0.33) \geq 50\%$.
  - A minimum of 12 participants have been treated in the trial.
  - These stopping criteria are applicable to Part 1A, Part 1B, [redacted]

9.4.2. Efficacy Analysis

Response Evaluable Set will be used for all response related analyses including BOR, DOR, CBR, PFS, TTP and OS.

Tumor response will be presented in the form of participant data listings that include, but are not limited to tumor type, dose on Day 1, tumor response at each visit, clinical benefit response and best overall response. Proportion of participants responding to the treatment will be presented for each dose level.

090177e19bd138e0\Approved\Approved On: 14-Nov-2022 20:57 (GMT)

For prostate cancer only, PSA and bone scan monitor, report and analysis should follow PCWG3 guidelines[1]. Existing or new bone lesions as assessed by radionuclide bone scan will be described and documented.

PSA data will be presented in form of listings that include dose on Day 1, measurement, and % change from baseline at visit.

Part 1A, Part 1B, [redacted] Progression date, date of first response, last tumor assessment date, and date of last contact will be listed.

Part 2: The Kaplan-Meier methods will be used to analyze all time to event endpoints. Median PFS (if reached) will be calculated. DOR will be listed. Details of these endpoint analyses methods will be included in the SAP. Efficacy data of participants from Part 1A, Part 1B, [redacted] treated at the dose level selected for the corresponding expansion cohort and satisfying inclusion/exclusion criteria might be included in the analysis of efficacy endpoints of Part 2.

PFS is defined as the time from start date to date of first documentation of progression, or death due to any cause. Progression is defined as the appearance of local, regional or distant disease of the same type after complete response or progression of pre-existing lesions. It does not include second primary malignancies of unrelated types.

TTP - defined as the time from date of first dose to the date of the first documentation of PD.

OS is defined as the time from start date to date of death due to any cause.

CBR - defined as the proportion of patients with a best overall response (BOR) of CR, PR or SD lasting for 24 weeks.

The definition of each response category is provided in Appendix 11 (Section 10.11) (RECIST v1.1).

9.4.3. Pharmacokinetic Analysis

9.4.3.1. Single Dose and Steady-State PF-07248144 Pharmacokinetic Analysis (With the Exception of Food Effect Assessment) [redacted]

Following single dose administration, plasma PK parameters including the C_{max}, T_{max}, AUC_{last}, and if data permit, AUC_{inf}, CL/F, V_z/F, and $t_{½}$ will be estimated. Following multiple doses, steady state PK parameters including $C_{max,ss}$, $T_{max,ss}$, $AUC_{\tau,ss}$, $C_{min,ss}$, CL_{ss}/F, and if data permit, V_{ss}/F, $t_{½}$, and R_{ac} ($AUC_{\tau,ss}/AUC_{\tau,sd}$) will be estimated.

The single-dose and steady-state PK parameters will be summarized descriptively (n, mean, standard deviation, CV, median, minimum, maximum, geometric mean and its associated CV) by dose, cycle and day.

090177e19bd138e0\Approved\Approved On: 14-Nov-2022 20:57 (GMT)

PF-07248144 concentrations will be summarized descriptively (n, mean, standard deviation, CV, median, minimum, maximum, geometric mean and its associated CV) by dose, cycle, day and nominal time. Individual participant and median profiles of the concentration-time data will be plotted by dose, cycle and day (single dose and multiple-dose) using nominal times. Individual and median PK profiles will be presented on both linear and log-linear scales for drug concentrations.

Dose normalized C_{max}, AUC_{last}, AUC_{τ} (at steady state) and AUC_{inf} (if estimated), and will be plotted against dose (using a logarithmic scale) by cycle and day. These plots will include individual patient values and the geometric means for each dose. These plots will be used to help understand the relationship between the PK parameters and dose.

The observed accumulation ratio will be summarized descriptively.

[redacted]

[redacted]

[redacted]

[redacted]

[redacted]

[redacted]

[redacted]

090177e19bd138e0\Approved\Approved On: 14-Nov-2022 20:57 (GMT)

[redacted]

9.4.3.5. Pharmacodynamic and Biomarker Analyses

Results from PD and biomarker exploratory analyses will be reported in the CSR where possible. However, given the exploratory nature of exploratory objectives and endpoints, the analyses may not be complete at the time of the CSR. Results from exploratory analyses that are not included in the CSR will be shared with the scientific community through publication at a scientific conference and/or in a peer-reviewed scientific journal.

[redacted].

9.4.5. Safety Analyses

All safety analyses will be performed on the safety population.

Summaries and analyses of safety parameters will include all participants in the safety analysis set.

090177e19bd138e0\Approved\Approved On: 14-Nov-2022 20:57 (GMT)

AEs, ECGs, BP, pulse rate, continuous cardiac monitoring, and safety laboratory data will be reviewed and summarized on an ongoing basis during the study to evaluate the safety of participants. Any clinical laboratory, ECG, BP, and PR abnormalities of potential clinical concern will be described. Safety data will be presented in tabular and/or graphical format and summarized descriptively, where appropriate.

Medical history and physical examination and neurological examination information, as applicable, collected during the course of the study will be considered source data and will not be required to be reported, unless otherwise noted. However, any untoward findings identified on physical and/or neurological examinations conducted during the active collection period will be captured as AEs, if those findings meet the definition of an AE. Data collected at screening that are used for inclusion/exclusion criteria, such as laboratory data, ECGs, and vital signs, will be considered source data, and will not be required to be reported, unless otherwise noted. Demographic data collected at screening will be reported.

9.4.5.1. Electrocardiogram Analyses

Changes from baseline for the following ECG parameters: QT interval, heart rate, QTc interval, PR interval, and QRS complex will be summarized by treatment and time.

The number (%) of participants with maximum postdose QTc values and maximum increases from baseline in the following categories will be tabulated by treatment:

Safety QTc Assessment

Degree of Prolongation	Mild (msec)	Moderate (msec)	Severe (msec)
Absolute value	>450-480	>480-500	>500
Increase from baseline		30-60	>60

In addition, the number of participants with uncorrected QT values >500 msec will be summarized.

If more than 1 ECG is collected at a nominal time after dose administration (for example, triplicate ECGs), the mean of the replicate measurements will be used to represent a single observation at that time point. If any of the 3 individual ECG tracings has a QTc value >500 msec, but the mean of the triplicates is not >500 msec, the data from the participant's individual tracing will be described in a safety section of the CSR in order to place the >500 msec value in appropriate clinical context. However, values from individual tracings within triplicate measurements that are >500 msec will not be included in the categorical analysis unless the average from the triplicate measurements is also >500 msec. Changes from baseline will be defined as the change between the postdose QTc value and the average of the time-matched baseline triplicate values on Day 1, or the average of the predose triplicate values on Day 1.

In addition, an attempt will be made to explore and characterize the relationship between plasma concentration and QT interval length using a PK/PD modeling approach. If a PK/PD relationship is found, the impact of participant factors (covariates) on the relationship will be examined.

090177e19bd138e0\Approved\Approved On: 14-Nov-2022 20:57 (GMT)

[redacted]

The analysis of ECG results will be based on participants in the safety analysis set with baseline and on-treatment ECG data. Baseline is defined as Cycle 1 Day 1 predose.

ECG measurements (an average of the triplicate measurements) will be used for the statistical analysis and all data presentations. Any data obtained from ECGs repeated for safety reasons after the nominal time-points will not be averaged along with the preceding triplicates. Interval measurements from repeated ECGs will be included in the outlier analysis (categorical analysis) as individual values obtained at unscheduled time points.

QT intervals will be corrected for HR (QTc) using standard correction factors (ie, Fridericia's [default correction], Bazett's, and possibly a study specific factor, as appropriate). Data will be summarized and listed for QT, HR, RR, PR, QRS, QTcF (and other correction factors, eg, QTcB as appropriate), and by dose level in Part 1A and by group in Part 2 (Part 2A, [redacted]). Individual QT (all evaluated corrections) intervals will be listed by dose and time in Part 1A and by cohort and time in Part 2. The most appropriate correction factor will be selected and used for the following analyses of central tendency and outliers and used for the study conclusions. Descriptive statistics (n, mean, median, standard deviation, minimum, and maximum) will be used to summarize the absolute corrected QT interval and changes from baseline in corrected QT after treatment by study cohort, dose and time point. Details of additional analysis (if any) will be specified in SAP.

9.4.5.2. Adverse Events

AEs will graded by the investigator according to the CTCAE version 5.0 and coded using MedDRA. AE data will be reported in tables and listings. Summaries of adverse event by mapped terms, appropriate thesaurus level, toxicity grade, and seriousness and relationship to study treatment will be presented, as well as summaries of adverse events leading to death and premature withdrawal from study treatment. The number and percentage of participants who experienced any AE, SAE, treatment related AE, and treatment related SAE will be summarized according to worst toxicity grades. The summaries will present AEs both on the entire study period and by cycle (Cycle 1 and Cycles beyond 1). Listings of DLTs and deaths will be provided.

9.4.5.3. Laboratory Test Abnormalities

The number and percentage of participants who experienced laboratory test abnormalities will be summarized according to worst toxicity grade observed for each laboratory assay. The analyses will summarize laboratory tests both on the entire study period and by cycle (Cycle 1 and Cycles beyond 1). For laboratory tests without CTCAE grade definitions, results will be categorized as normal, abnormal, or not done.

9.4.6. Other Analyse(s)

9.5. Interim Analyses

No formal interim analysis will be conducted for this study. As this is an open-label study, the sponsor may conduct unblinded reviews of the data during the course of the study for the purpose of safety assessment, facilitating dose-escalation decisions, facilitating PK/PD modeling, and/or supporting clinical development.

090177e19bd138e0\Approved\Approved On: 14-Nov-2022 20:57 (GMT)

9.6. Data Monitoring Committee or Other Independent Oversight Committee

This is an open-label, non-randomized Phase 1 study. This study will not use a DMC. Discussions between the investigators and the sponsor regarding safety will occur in an ongoing manner at regular teleconferences and/or meetings to determine the safety profile and risk/benefit ratio and determine if further participant enrollment is appropriate. These individual and summary data would also include participants who are determined to be not applicable for DLT assessment.

10. SUPPORTING DOCUMENTATION AND OPERATIONAL CONSIDERATIONS

10.1. Appendix 1: Regulatory, Ethical, and Study Oversight Considerations

10.1.1. Regulatory and Ethical Considerations

This study will be conducted in accordance with the protocol and with the following:

- Consensus ethical principles derived from international guidelines including the Declaration of Helsinki and CIOMS International Ethical Guidelines;
- Applicable ICH GCP guidelines;
- Applicable laws and regulations, including applicable privacy laws.

The protocol, protocol amendments, ICD, SRSD(s), and other relevant documents (eg, advertisements) must be reviewed and approved by the sponsor and submitted to an IRB/EC by the investigator and reviewed and approved by the IRB/EC before the study is initiated.

Any amendments to the protocol will require IRB/EC approval before implementation of changes made to the study design, except for changes necessary to eliminate an immediate hazard to study participants.

The investigator will be responsible for the following:

- Providing written summaries of the status of the study to the IRB/EC annually or more frequently in accordance with the requirements, policies, and procedures established by the IRB/EC.
- Notifying the IRB/EC of SAEs or other significant safety findings as required by IRB/EC procedures.
- Providing oversight of the conduct of the study at the site and adherence to requirements of 21 CFR, ICH guidelines, the IRB/EC, European regulation 536/2014 for clinical studies (if applicable), and all other applicable local regulations.

10.1.1.1. Reporting of Safety Issues and Serious Breaches of the Protocol or ICH GCP

- In the event of any prohibition or restriction imposed (ie, clinical hold) by an applicable regulatory authority in any area of the world, or if the investigator is aware

090177e19bd138e0\Approved\Approved On: 14-Nov-2022 20:57 (GMT)

of any new information that might influence the evaluation of the benefits and risks of the study intervention, Pfizer should be informed immediately.

- In addition, the investigator will inform Pfizer immediately of any urgent safety measures taken by the investigator to protect the study participants against any immediate hazard, and of any serious breaches of this protocol or of ICH GCP that the investigator becomes aware of.

[redacted]

10.1.3. Informed Consent Process

The investigator or his/her representative will explain the nature of the study to the participant and answer all questions regarding the study. The participant should be given sufficient time and opportunity to ask questions and to decide whether or not to participate in the trial.

Participants must be informed that their participation is voluntary. Participants will be required to sign a statement of informed consent that meets the requirements of 21 CFR 50, local regulations, ICH guidelines, HIPAA requirements, where applicable, and the IRB/EC or study center.

The investigator must ensure that each study participant is fully informed about the nature and objectives of the study, the sharing of data related to the study, and possible risks associated with participation, including the risks associated with the processing of the participant's personal data.

The participant must be informed that his/her personal study-related data will be used by the sponsor in accordance with local data protection law. The level of disclosure must also be explained to the participant.

The participant must be informed that his/her medical records may be examined by Clinical Quality Assurance auditors or other authorized personnel appointed by the sponsor, by appropriate IRB/EC members, and by inspectors from regulatory authorities.

The investigator further must ensure that each study participant is fully informed about his or her right to access and correct his or her personal data and to withdraw consent for the processing of his or her personal data.

The medical record must include a statement that written informed consent was obtained before the participant was enrolled in the study and the date the written consent was obtained. The authorized person obtaining the informed consent must also sign the ICD.

090177e19bd138e0\Approved\Approved On: 14-Nov-2022 20:57 (GMT)

Participants must be reconsented to the most current version of the ICD(s) during their participation in the study.

A copy of the ICD(s) must be provided to the participant.

A participant who is rescreened is not required to sign another ICD if the rescreening occurs within 28 days from the previous ICD signature date.

Unless prohibited by local requirements or IRB/EC decision, the ICD will contain a separate section that addresses the use of samples for optional additional research. The optional additional research does not require the collection of any further samples. The investigator or authorized designee will explain to each participant the objectives of the additional research. Participants will be told that they are free to refuse to participate and may withdraw their consent at any time and for any reason during the storage period. A separate signature will be required to document a participant's agreement to allow specimens to be used for additional research. Participants who decline to participate in this optional additional research will not provide this separate signature.

10.1.4. Data Protection

All parties will comply with all applicable laws, including laws regarding the implementation of organizational and technical measures to ensure protection of participant data.

Participants' personal data will be stored at the study site in encrypted electronic and/or paper form and will be password protected or secured in a locked room to ensure that only authorized study staff have access. The study site will implement appropriate technical and organizational measures to ensure that the personal data can be recovered in the event of disaster. In the event of a potential personal data breach, the study site will be responsible for determining whether a personal data breach has in fact occurred and, if so, providing breach notifications as required by law.

To protect the rights and freedoms of participants with regard to the processing of personal data, participants will be assigned a single, participant-specific numerical code. Any participant records or data sets that are transferred to the sponsor will contain the numerical code; participant names will not be transferred. All other identifiable data transferred to the sponsor will be identified by this single, participant-specific code. The study site will maintain a confidential list of participants who participated in the study, linking each participant's numerical code to his or her actual identity and medical record identification. In case of data transfer, the sponsor will protect the confidentiality of participants' personal data consistent with the clinical study agreement and applicable privacy laws.

[redacted]

[redacted]

090177e19bd138e0\Approved\Approved On: 14-Nov-2022 20:57 (GMT)

[redacted]

[redacted]

[redacted]

[redacted]

[redacted]

[redacted]

[redacted]

[redacted]

[redacted]

[redacted]

[redacted]

[redacted]

090177e19bd138e0\Approved\Approved On: 14-Nov-2022 20:57 (GMT)

[redacted]

[redacted]

[redacted]

[redacted]

[redacted]

[redacted]

[redacted]

[redacted]

[redacted]

[redacted]

090177e19bd138e0\Approved\Approved On: 14-Nov-2022 20:57 (GMT)

[redacted]

[redacted]

[redacted]

[redacted]

[redacted]

[redacted]

[redacted]

[redacted]

[redacted]

[redacted]

[redacted]

[redacted]

090177e19bd138e0\Approved\Approved On: 14-Nov-2022 20:57 (GMT)

[redacted]

090177e19bd138e0\Approved\Approved On: 14-Nov-2022 20:57 (GMT)

[redacted]

[redacted]

[redacted]

090177e19bd138e0\Approved\Approved On: 14-Nov-2022 20:57 (GMT)

10.2. Appendix 2: Clinical Laboratory Tests

The following safety laboratory tests will be performed at times defined in the SoA section of this protocol. Additional laboratory results may be reported on these samples as a result of the method of analysis or the type of analyzer used by the clinical laboratory, or as derived from calculated values. These additional tests would not require additional collection of blood. Unscheduled clinical laboratory measurements may be obtained at any time during the study to assess any perceived safety issues.

Table 13. Safety Laboratory Tests

Hematology	Chemistry	Serology	Coagulation	Urinalysis	Pregnancy Test
Hemoglobin	ALT	HBV*	PT	Urine dipstick for urine blood or leukocyte esterase.<br><br>Urine dipstick:<br>pH<br>Glucose (qual)<br>Protein (qual)<br>Blood (qual)<br>Ketones<br>Nitrites<br>Leukocyte esterase<br>Urobilinogen<br>Urine bilirubin	For female participants of childbearing potential, serum or urine.
Platelets	AST	HCV Ab	PTT or aPTT		
WBC	Bicarbonate **	HIV Ab			
Absolute Neutrophils	CRP	PSA			
Absolute Lymphocytes	Alk Phos				
Absolute Monocytes	Sodium				
Absolute Eosinophils	Potassium				
Absolute Basophils	Magnesium			Microscopy[a]	
	Chloride				
	Total calcium				
	Total bilirubin***				
	Total Protein				
	BUN				
	Creatinine				
	Uric Acid				
	Glucose				
	LDH				
	Albumin				
	Phosphorus or Phosphate				
	Amylase				
	Lipase				

* HbsAg, HbcAb, anti-HBs, HCVAb, and HIV to be conducted by local laboratory where required by local regulations or if warranted by participant history
**CO2 will be used in Korea per clinical practice.
*** For potential Hy's Law cases, in addition to repeating AST and ALT, laboratory tests should include albumin, creatine kinase, total bilirubin, direct and indirect bilirubin, gamma-glutamyl transferase, PT/INR, alkaline phosphatase, total bile acids and acetaminophen drug and/or protein adduct levels.
a. Only if urine dipstick is positive for blood, protein, nitrites, or leukocyte esterase.

Investigators must document their review of each laboratory safety report.

090177e19bd138e0\Approved\Approved On: 14-Nov-2022 20:57 (GMT)

10.3. Appendix 3: Adverse Events: Definitions and Procedures for Recording, Evaluating, Follow-up, and Reporting

10.3.1. Definition of AE

**AE Definition**
• An AE is any untoward medical occurrence in a patient or clinical study participant, temporally associated with the use of study intervention, whether or not considered related to the study intervention.<br><br>• NOTE: An AE can therefore be any unfavorable and unintended sign (including an abnormal laboratory finding), symptom, or disease (new or exacerbated) temporally associated with the use of study intervention.

**Events <u>Meeting</u> the AE Definition**
• Any abnormal laboratory test results (hematology, clinical chemistry, or urinalysis) or other safety assessments (eg, ECG, radiological scans, vital sign measurements), including those that worsen from baseline, considered clinically significant in the medical and scientific judgment of the investigator. Any abnormal laboratory test results that meet any of the conditions below must be recorded as an AE:<br><br>• Is associated with accompanying symptoms;<br><br>• Requires additional diagnostic testing or medical/surgical intervention;<br><br>• Leads to a change in study dosing (outside of any protocol-specified dose adjustments) or discontinuation from the study, significant additional concomitant drug treatment, or other therapy.<br><br>• Exacerbation of a chronic or intermittent preexisting condition including either an increase in frequency and/or intensity of the condition.<br><br>• New conditions detected or diagnosed after study intervention administration even though it may have been present before the start of the study.<br><br>• Signs, symptoms, or the clinical sequelae of a suspected drug -drug interaction.<br><br>• Signs, symptoms, or the clinical sequelae of a suspected overdose of either study intervention or a concomitant medication. Overdose per se will not be reported as an AE/SAE unless it is an intentional overdose taken with possible suicidal/self-harming intent. Such overdoses should be reported regardless of sequelae.

090177e19bd138e0\Approved\Approved On: 14-Nov-2022 20:57 (GMT)

Events NOT Meeting the AE Definition
• Any clinically significant abnormal laboratory findings or other abnormal safety assessments which are associated with the underlying disease, unless judged by the investigator to be more severe than expected for the participant's condition.<br><br>• The disease/disorder being studied or expected progression, signs, or symptoms of the disease/disorder being studied, unless more severe than expected for the participant's condition.<br><br>• Medical or surgical procedure (eg, endoscopy, appendectomy): the condition that leads to the procedure is the AE.<br><br>• Situations in which an untoward medical occurrence did not occur (social and/or convenience admission to a hospital).<br><br>• Anticipated day-to-day fluctuations of preexisting disease(s) or condition(s) present or detected at the start of the study that do not worsen.<br><br>• Worsening of signs and symptoms of the malignancy under study should be recorded as AEs in the appropriate section of the CRF. Disease progression assessed by measurement of malignant lesions on radiographs or other methods should not be reported as AEs.

10.3.2. Definition of SAE

If an event is not an AE per definition above, then it cannot be an SAE even if serious conditions are met (eg, hospitalization for signs/symptoms of the disease under study, death due to progression of disease).

An SAE is defined as any untoward medical occurrence that, at any dose:
a. Results in death
b. Is life threatening<br><br>The term "life threatening" in the definition of "serious" refers to an event in which the participant was at risk of death at the time of the event. It does not refer to an event that hypothetically might have caused death if it were more severe.
c. Requires inpatient hospitalization or prolongation of existing hospitalization<br><br>In general, hospitalization signifies that the participant has been detained (usually involving at least an overnight stay) at the hospital or emergency ward for observation and/or treatment that would not have been appropriate in the physician's office or outpatient setting. Complications that occur during hospitalization are AEs. If a complication prolongs hospitalization or fulfills any other serious criteria, the event is

090177e19bd138e0\Approved\Approved On: 14-Nov-2022 20:57 (GMT)

serious. When in doubt as to whether "hospitalization" occurred or was necessary, the AE should be considered serious.

Hospitalization for elective treatment of a preexisting condition that did not worsen from baseline is not considered an AE.

d. Results in persistent disability/incapacity

- The term disability means a substantial disruption of a person's ability to conduct normal life functions.
- This definition is not intended to include experiences of relatively minor medical significance such as uncomplicated headache, nausea, vomiting, diarrhea, influenza, and accidental trauma (eg, sprained ankle) which may interfere with or prevent everyday life functions but do not constitute a substantial disruption.

e. Is a congenital anomaly/birth defect

f. Other situations:

- Medical or scientific judgment should be exercised in deciding whether SAE reporting is appropriate in other situations such as important medical events that may not be immediately life threatening or result in death or hospitalization but may jeopardize the participant or may require medical or surgical intervention to prevent one of the other outcomes listed in the above definition. These events should usually be considered serious.
- Examples of such events include invasive or malignant cancers, intensive treatment in an emergency room or at home for allergic bronchospasm, blood dyscrasias or convulsions that do not result in hospitalization, or development of drug dependency or drug abuse.
- Progression of the malignancy under study (including signs and symptoms of progression) should not be reported as an SAE unless the outcome is fatal within the active collection period. Hospitalization due to signs and symptoms of disease progression should not be reported as an SAE. If the malignancy has a fatal outcome during the study or within the active collection period, then the event leading to death must be recorded as an AE on the CRF, and as an SAE with CTCAE Grade 5 (see the Assessment of Intensity section).
- Suspected transmission via a Pfizer product of an infectious agent, pathogenic or non-pathogenic, is considered serious. The event may be suspected from clinical symptoms or laboratory findings indicating an infection in a patient exposed to a Pfizer product. The terms "suspected transmission" and "transmission" are considered synonymous. These cases are considered unexpected and handled as serious expedited cases by pharmacovigilance personnel. Such cases are also considered for reporting as product defects, if appropriate.

090177e19bd138e0\Approved\Approved On: 14-Nov-2022 20:57 (GMT)

10.3.3. Recording/Reporting and Follow-up of AEs and/or SAEs

AE and SAE Recording/Reporting

The table below summarizes the requirements for recording adverse events on the CRF and for reporting serious adverse events on the CT SAE Report Form to Pfizer Safety. These requirements are delineated for 3 types of events: (1) SAEs; (2) nonserious adverse events (AEs); and (3) exposure to the study intervention under study during pregnancy or breastfeeding, and occupational exposure.

It should be noted that the CT SAE Report Form for reporting of SAE information is not the same as the AE page of the CRF. When the same data are collected, the forms must be completed in a consistent manner. AEs should be recorded using concise medical terminology and the same AE term should be used on both the CRF and the CT SAE Report Form for reporting of SAE information.

Safety Event	Recorded on the CRF	Reported on the CT SAE Report Form to Pfizer Safety Within 24 Hours of Awareness
SAE	All	All
Nonserious AE	All	None
Exposure to the study intervention under study during pregnancy or breastfeeding, and occupational exposure	All AEs/SAEs associated with exposure during pregnancy or breastfeeding.<br><br>Occupational exposure is not recorded.	All (and EDP supplemental form for EDP).<br>Note: Include all SAEs associated with exposure during pregnancy or breastfeeding. Include all AEs/SAEs associated with occupational exposure.

- When an AE/SAE occurs, it is the responsibility of the investigator to review all documentation (eg, hospital progress notes, laboratory reports, and diagnostic reports) related to the event.
- The investigator will then record all relevant AE/SAE information in the CRF.
- It is **not** acceptable for the investigator to send photocopies of the participant's medical records to Pfizer Safety in lieu of completion of the CT SAE Report Form/AE/SAE CRF page.
- There may be instances when copies of medical records for certain cases are requested by Pfizer Safety. In this case, all participant identifiers, with the exception of the participant number, will be redacted on the copies of the medical records before submission to Pfizer Safety.

090177e19bd138e0\Approved\Approved On: 14-Nov-2022 20:57 (GMT)

- The investigator will attempt to establish a diagnosis of the event based on signs, symptoms, and/or other clinical information. Whenever possible, the diagnosis (not the individual signs/symptoms) will be documented as the AE/SAE.

Assessment of Intensity

The investigator will make an assessment of intensity for each AE and SAE reported during the study and assign it to 1 of the following categories:

An event is defined as "serious" when it meets at least 1 of the predefined outcomes as described in the definition of an SAE, NOT when it is rated as severe.

GRADE	Clinical Description of Severity
1	MILD adverse event
2	MODERATE adverse event
3	SEVERE adverse event
4	LIFE-THREATENING consequences; urgent intervention indicated
5	DEATH RELATED TO adverse event

Assessment of Causality

- The investigator is obligated to assess the relationship between study intervention and each occurrence of each AE/SAE.
- A "reasonable possibility" of a relationship conveys that there are facts, evidence, and/or arguments to suggest a causal relationship, rather than a relationship cannot be ruled out.
- The investigator will use clinical judgment to determine the relationship.
- Alternative causes, such as underlying disease(s), concomitant therapy, and other risk factors, as well as the temporal relationship of the event to study intervention administration, will be considered and investigated.
- The investigator will also consult the IB and/or product information, for marketed products, in his/her assessment.
- For each AE/SAE, the investigator **must** document in the medical notes that he/she has reviewed the AE/SAE and has provided an assessment of causality.

090177e19bd138e0\Approved\Approved On: 14-Nov-2022 20:57 (GMT)

- There may be situations in which an SAE has occurred and the investigator has minimal information to include in the initial report to the sponsor. However, **it is very important that the investigator always make an assessment of causality for every event before the initial transmission of the SAE data to the sponsor.**
- The investigator may change his/her opinion of causality in light of follow-up information and send an SAE follow-up report with the updated causality assessment.
- The causality assessment is one of the criteria used when determining regulatory reporting requirements.
- If the investigator does not know whether or not the study intervention caused the event, then the event will be handled as "related to study intervention" for reporting purposes, as defined by the sponsor. In addition, if the investigator determines that an SAE is associated with study procedures, the investigator must record this causal relationship in the source documents and CRF, and report such an assessment in the dedicated section of the CT SAE Report Form and in accordance with the SAE reporting requirements.

Follow-up of AEs and SAEs

- The investigator is obligated to perform or arrange for the conduct of supplemental measurements and/or evaluations as medically indicated or as requested by the sponsor to elucidate the nature and/or causality of the AE or SAE as fully as possible. This may include additional laboratory tests or investigations, histopathological examinations, or consultation with other healthcare providers.
- If a participant dies during participation in the study or during a recognized follow-up period, the investigator will provide Pfizer Safety with a copy of any postmortem findings including histopathology.
- New or updated information will be recorded in the originally completed CRF.
- The investigator will submit any updated SAE data to the sponsor within 24 hours of receipt of the information.

090177e19bd138e0\Approved\Approved On: 14-Nov-2022 20:57 (GMT)

10.3.4. Reporting of SAEs

SAE Reporting to Pfizer Safety via CT SAE Report Form
• Facsimile transmission of the CT SAE Report Form is the preferred method to transmit this information to Pfizer Safety.<br><br>• In circumstances when the facsimile is not working, notification by telephone is acceptable with a copy of the CT SAE Report Form sent by overnight mail or courier service.<br><br>• Initial notification via telephone does not replace the need for the investigator to complete and sign the CT SAE Report Form pages within the designated reporting time frames.

090177e19bd138e0\Approved\Approved On: 14-Nov-2022 20:57 (GMT)

10.4. Appendix 4: Contraceptive Guidance

10.4.1. Male Participant Reproductive Inclusion Criteria

Male participants are eligible to participate if they agree to the following requirements during the intervention period and for at least 93 days after the last dose of study intervention, which corresponds to the time needed to eliminate reproductive safety risk of the study intervention(s) ***plus*** an additional 90 days (a spermatogenesis cycle):

- Refrain from donating sperm.

  PLUS either**:**

- Be abstinent from heterosexual or homosexual intercourse as their preferred and usual lifestyle (abstinent on a long term and persistent basis) and agree to remain abstinent.

  OR

- Must agree to use contraception/barrier as detailed below:
  - Agree to use a male condom when engaging in any activity that allows for passage of ejaculate to another person.
- Male participants should be advised of the benefit for a female partner to use a highly effective method of contraception, as a condom may break or leak when having sexual intercourse with a WOCBP who is not currently pregnant.

10.4.2. Female Participant Reproductive Inclusion Criteria

- A female participant is eligible to participate if she is not pregnant or breastfeeding (women who are currently breastfeeding and intend to interrupt breastfeeding are excluded from participating), and at least 1 of the following conditions applies:
- Is not a WOCBP (see definitions below in Section 10.4.3).

  OR

- Is a WOCBP and using a contraceptive method that is highly effective (with a failure rate of <1% per year), preferably with low user dependency, as described below during the intervention period and for at least 28 days after the last dose of study intervention, which corresponds to the time needed to eliminate any reproductive safety risk of the study intervention(s). The investigator should evaluate the effectiveness of the contraceptive method in relationship to the first dose of study intervention.
  - Is a WOCBP and using a contraceptive method that is highly effective (with a failure rate of <1% per year), with high user dependency, as described below during the intervention period and for at least 28 days after the last dose of study intervention, which corresponds to the time needed to eliminate any reproductive safety risk of the

090177e19bd138e0\Approved\Approved On: 14-Nov-2022 20:57 (GMT)

study intervention(s). In addition, a second effective method of contraception, as described below, must be used. The investigator should evaluate the effectiveness of the contraceptive method in relationship to the first dose of study intervention.

- A WOCBP agrees not to donate eggs (ova, oocytes) for the purpose of reproduction during this period. The investigator should evaluate the effectiveness of the contraceptive method in relationship to the first dose of study intervention.
- The investigator is responsible for review of medical history, menstrual history, and recent sexual activity to decrease the risk for inclusion of a woman with an early undetected pregnancy.

10.4.3. Woman of Childbearing Potential

A woman is considered fertile following menarche and until becoming postmenopausal unless permanently sterile (see below).

If fertility is unclear (eg, amenorrhea in adolescents or athletes) and a menstrual cycle cannot be confirmed before the first dose of study intervention, additional evaluation should be considered.

Women in the following categories are not considered WOCBP:

1. Premenopausal female with 1 of the following:
   - Documented hysterectomy;
   - Documented bilateral salpingectomy;
   - Documented bilateral oophorectomy.

   For individuals with permanent infertility due to an alternate medical cause other than the above, (eg, mullerian agenesis, androgen insensitivity), investigator discretion should be applied to determining study entry.

   Note: Documentation for any of the above categories can come from the site personnel's review of the participant's medical records, medical examination, or medical history interview. The method of documentation should be recorded in the participant's medical record for the study.

2. Postmenopausal female.
   - A postmenopausal state is defined as no menses for 12 months without an alternative medical cause. In addition, a
     - High FSH level in the postmenopausal range must be used to confirm a postmenopausal state in women under 60 years old and not using hormonal

090177e19bd138e0\Approved\Approved On: 14-Nov-2022 20:57 (GMT)

[redacted]

contraception or HRT. When there is a high FSH level, it should be confirmed that there is no other medical cause.

- Female on HRT and whose menopausal status is in doubt will be required to use one of the non-estrogen hormonal highly effective contraception methods if they wish to continue their HRT during the study. Otherwise, they must discontinue HRT to allow confirmation of postmenopausal status before study enrollment.

10.4.4. Contraception Methods

Contraceptive use by men or women should be consistent with local availability/regulations regarding the use of contraceptive methods for those participating in clinical trials.

<u>Highly Effective Methods That Have Low User Dependency</u>

1. Implantable progestogen only hormone contraception associated with inhibition of ovulation*.
2. Intrauterine device.
3. Intrauterine hormone -releasing system.
4. Bilateral tubal occlusion.
5. Vasectomized partner.
   - Vasectomized partner is a highly effective contraceptive method provided that the partner is the sole sexual partner of the WOCBP and the absence of sperm has been confirmed. If not, an additional highly effective method of contraception should be used. The spermatogenesis cycle is approximately 90 days.
   - * not approved in Japan.

<u>Highly Effective Methods That Are User Dependent</u>

6. Combined (estrogen and progestogen-containing) hormonal contraception associated with inhibition of ovulation.
   - Oral;
   - Intravaginal*;
   - Transdermal*;
   - * not approved in Japan.
7. Progestogen only hormone contraception associated with inhibition of ovulation.

090177e19bd138e0\Approved\Approved On: 14-Nov-2022 20:57 (GMT)

- Oral*;
- Injectable*.
- not approved in Japan.

8. Sexual abstinence.

   - Sexual abstinence is considered a highly effective method only if defined as refraining from heterosexual intercourse during the entire period of risk associated with the study intervention. The reliability of sexual abstinence needs to be evaluated in relation to the duration of the study and the preferred and usual lifestyle of the participant.

One of the following effective barrier methods must be used in addition to the highly effective methods listed above that are user dependent:

- Male or female* condom with or without spermicide;
- Cervical cap*, diaphragm*, or sponge with spermicide*;
- A combination of male condom with either cervical cap*, diaphragm*, or sponge with spermicide* (double-barrier methods).
  - * not approved in Japan.

090177e19bd138e0\Approved\Approved On: 14-Nov-2022 20:57 (GMT)

10.5. Appendix 5: Genetics

Use/Analysis of DNA

- Genetic variation may impact a participant's response to study intervention, susceptibility to, and severity and progression of disease. Therefore, where local regulations and IRBs/Ecs allow, a blood sample will be collected for DNA analysis.

- The scope of the genetic research may be narrow (eg, 1 or more candidate genes) or broad (eg, the entire genome), as appropriate to the scientific question under investigation.

- The samples may be analyzed as part of a multistudy assessment of genetic factors involved in the response to PF-07248144 [redacted]

- The results of genetic analyses may be reported in the CSR or in a separate study summary, or may be used for internal decision making without being included in a study report.

- The sponsor will store the DNA samples in a secure storage space with adequate measures to protect confidentiality.

- The samples will be retained as indicated:

    - Samples for specified genetic analysis (see Section 8.7.1) for a period of up to 3 years after regulatory approval.

    - Samples for banking (see Section 8.7.2) will be stored indefinitely or for another period as per local requirements.

- Participants may withdraw their consent for the storage and/or use of their Banked Biospecimens at any time by making a request to the investigator; in this case, any remaining material will be destroyed. Data already generated from the samples will be retained to protect the integrity of existing analyses.

- Banked Biospecimens will be labeled with a code. The key between the code and the participant's personally identifying information (eg, name, address) will be held at the study site and will not be provided to the sample bank.

090177e19bd138e0\Approved\Approved On: 14-Nov-2022 20:57 (GMT)

10.6. Appendix 6: Liver Safety: Suggested Actions and Follow-up Assessments

Potential Cases of Drug-Induced Liver Injury

Humans exposed to a drug who show no sign of liver injury (as determined by elevations in transaminases) are termed "tolerators," while those who show transient liver injury, but adapt are termed "adaptors." In some participants, transaminase elevations are a harbinger of a more serious potential outcome. These participants fail to adapt and therefore are "susceptible" to progressive and serious liver injury, commonly referred to as DILI. Participants who experience a transaminase elevation above 3 × ULN should be monitored more frequently to determine if they are an "adaptor" or are "susceptible."

In the majority of DILI cases, elevations in AST and/or ALT precede Tbili elevations (>2 × ULN) by several days or weeks. The increase in Tbili typically occurs while AST/ALT is/are still elevated above 3 × ULN (ie, AST/ALT and Tbili values will be elevated within the same laboratory sample). In rare instances, by the time Tbili elevations are detected, AST/ALT values might have decreased. This occurrence is still regarded as a potential DILI. Therefore, abnormal elevations in either AST OR ALT in addition to Tbili that meet the criteria outlined below are considered potential DILI (assessed per Hy's law criteria) cases and should always be considered important medical events, even before all other possible causes of liver injury have been excluded.

The threshold of laboratory abnormalities for a potential DILI case depends on the participant's individual baseline values and underlying conditions. Participants who present with the following laboratory abnormalities should be evaluated further as potential DILI (Hy's law) cases to definitively determine the etiology of the abnormal laboratory values:

- Participants with AST/ALT and Tbili baseline values within the normal range who subsequently present with AST OR ALT values >3 × ULN AND a Tbili value >2 × ULN with no evidence of hemolysis and an alkaline phosphatase value <2 × ULN or not available.

- For participants with baseline AST **OR** ALT **OR** Tbili values above the ULN, the following threshold values are used in the definition mentioned above, as needed, depending on which values are above the ULN at baseline:

  - Preexisting AST or ALT baseline values above the normal range: AST or ALT values >2 times the baseline values AND >3 × ULN; or >8 × ULN (whichever is smaller).

  - Preexisting values of Tbili above the normal range: Tbili level increased from baseline value by an amount of at least 1 × ULN **or** if the value reaches >3 × ULN (whichever is smaller).

Rises in AST/ALT and Tbili separated by more than a few weeks should be assessed individually based on clinical judgment; any case where uncertainty remains as to whether it represents a potential Hy's law case should be reviewed with the sponsor.

[redacted]

090177e19bd138e0\Approved\Approved On: 14-Nov-2022 20:57 (GMT)

The participant should return to the investigator site and be evaluated as soon as possible, preferably within 48 hours from awareness of the abnormal results. This evaluation should include laboratory tests, detailed history, and physical assessment.

In addition to repeating measurements of AST and ALT and Tbili for suspected cases of Hy's law, additional laboratory tests should include albumin, CK, direct and indirect bilirubin, GGT, PT/INR, total bile acids, and alkaline phosphatase. Consideration should also be given to drawing a separate tube of clotted blood and an anticoagulated tube of blood for further testing, as needed, for further contemporaneous analyses at the time of the recognized initial abnormalities to determine etiology. A detailed history, including relevant information, such as review of ethanol, acetaminophen/paracetamol (either by itself or as a co-formulated product in prescription or over-the-counter medications), recreational drug, supplement (herbal) use and consumption, family history, sexual history, travel history, history of contact with a jaundiced person, surgery, blood transfusion, history of liver or allergic disease, and potential occupational exposure to chemicals, should be collected. Further testing for acute hepatitis A, B, C, D, and E infection and liver imaging (eg, biliary tract) and collection of serum samples for acetaminophen/paracetamol drug and/or protein adduct levels may be warranted.

All cases demonstrated on repeat testing as meeting the laboratory criteria of AST/ALT and Tbili elevation defined above should be considered potential DILI (Hy's law) cases if no other reason for the LFT abnormalities has yet been found. **Such potential DILI (Hy's law) cases are to be reported as SAEs, irrespective of availability of all the results of the investigations performed to determine etiology of the LFT abnormalities.**

A potential DILI (Hy's law) case becomes a confirmed case only after all results of reasonable investigations have been received and have excluded an alternative etiology.

090177e19bd138e0\Approved\Approved On: 14-Nov-2022 20:57 (GMT)

10.7. Appendix 7: ECG Findings of Potential Clinical Concern

**ECG Findings That May Qualify as AEs**
• Marked sinus bradycardia (rate <40 bpm) lasting minutes.<br>• New PR interval prolongation >280 msec.<br>• New prolongation of QTcF to >480 msec (absolute) or by ≥60 msec from baseline.<br>• New onset- atrial flutter or fibrillation, with controlled ventricular response rate: ie, rate <120 bpm.<br>• New -onset type I second degree- (Wenckebach) AV block of >30 seconds' duration.<br>• Frequent PVCs, triplets, or short intervals (<30 seconds) of consecutive ventricular complexes.
**ECG Findings That May Qualify as SAEs**
• QTcF prolongation >500 msec.<br>• New STT changes suggestive of myocardial ischemia.<br>• New onset left bundle branch block (QRS >120 msec).<br>• New onset right bundle branch block (QRS >120 msec).<br>• Symptomatic bradycardia.<br>• Asystole:<br>• In awake, symptom-free participants in sinus rhythm, with documented periods of asystole ≥3.0 seconds or any escape rate <40 bpm, or with an escape rhythm that is below the AV node.<br>• In awake, symptom-free participants with atrial fibrillation and bradycardia with 1 or more pauses of at least 5 seconds or longer.<br>• Atrial flutter or fibrillation, with rapid ventricular response rate: rapid = rate >120 bpm.<br>• Sustained supraventricular tachycardia (rate >120 bpm) ("sustained" = short duration with relevant symptoms or lasting >1 minute).<br>• Ventricular rhythms >30 seconds' duration, including idioventricular rhythm (heart rate <40 bpm), accelerated idioventricular rhythm (HR >40 bpm to <100 bpm), and

090177e19bd138e0\Approved\Approved On: 14-Nov-2022 20:57 (GMT)

monomorphic/polymorphic ventricular tachycardia (HR >100 bpm (such as torsades de pointes)).

- Type II second degree (Mobitz II) AV block.
- Complete (third degree) heart block.

ECG Findings That Qualify as SAEs

- Change in pattern suggestive of new myocardial infarction.
- Sustained ventricular tachyarrhythmias (>30 seconds' duration).
- Second- or third -degree AV block requiring pacemaker placement.
- Asystolic pauses requiring pacemaker placement.
- Atrial flutter or fibrillation with rapid ventricular response requiring cardioversion.
- Ventricular fibrillation/flutter.
- At the discretion of the investigator, any arrhythmia classified as an adverse experience.

The enumerated list of major events of potential clinical concern are recommended as "alerts" or notifications from the core ECG laboratory to the investigator and Pfizer study team, and not to be considered as all-inclusive of what to be reported as AEs/SAEs.

090177e19bd138e0\Approved\Approved On: 14-Nov-2022 20:57 (GMT)

10.8. Appendix 8: Country-Specific Requirements.

10.8.1. Japan Specific Requirements

10.8.1.1. Japan Participation, Enrollment and General Safety Monitoring

Conditions for judging whether the participant can be discharged during the DLT evaluation period

When a participant is discharged from the hospital during the DLT evaluation period, the following conditions/status of the patient should be evaluated on the day of the scheduled discharge by the investigators, and the propriety of discharge should be determined. The tests/medical examinations needed to confirm the participant's status will be conducted per clinical practice at the study site by investigator's judgement as appropriate.

- There are currently no clinically significant adverse events or other medical conditions that require monitoring in a hospital setting.
- If a clinically significant adverse event has occurred or continues to be present, the investigator has determined that the event is manageable by appropriate treatment or prophylaxis in an out of the hospital setting. The investigator will ensure the adverse event is followed up according to the protocol requirement.
- Overall physical condition is stable and acceptable.
- In case of emergency, the participant may return to the clinical study site or other medical institution. If participants go to a medical institution other than the clinical study site, the clinical study site asks that the participants contact the study site and study investigator and the doctor at the medical institution will communicate to discuss appropriate treatments. A study site keeps ready for emergency situations and is available even during nights and holidays, and the sponsor will ensure and the selected study site will thoroughly follow all participants according to study procedures.

Japan will be considered to participate in Part 1B, [redacted] and Part 2 expansion only after confirmation of tolerability of PF-07248144 in Japanese participants in Part 1A.

- The enrollment of participants into Parts 1B, [redacted] will be conducted within the range of the dose levels at which PF-07248144 monotherapy is tolerated in Part 1A. The dose of PF-07248144 in Parts 1B, [redacted] will be escalated within the range of the Maximum Tolerated Dose (MTD) levels investigated of monotherapy in Part 1A.

The dose administration interval between the first and second participants

- The interval of the initial administration of PF-07248144 between the first Japanese participant and the second Japanese participant enrolled in the new dose level will be

090177e19bd138e0\Approved\Approved On: 14-Nov-2022 20:57 (GMT)

at least 48 hours within the same dose level cohorts to ensure the safety of participants in Part 1A.

[redacted]

- [redacted]

10.8.1.2. Modification and Addition of Exclusion Criteria

Modification of exclusion criterion for Hepatitis B and the monitoring for Hepatitis B Virus (HBV)

- Participants with active, uncontrolled bacterial, fungal, or viral infection, including (but not limited to) HBV (HbsAg positive), HCV, known HIV or AIDS related illness. HIV seropositive subjects who are healthy and low risk for AIDS-related outcomes could be considered eligible.

- Participants with positive HBs Ab and positive HBc Ab are allowed to participate in the study if they have negative HBV DNA test at screening but HB viral load should be monitored for re-activation every 12 weeks. Participants with HBs Ab positive who get vaccinated with HBV are exempted from the testing of HB viral load.

- Participants who test positive for HBV viral load at any time during the study will interrupt administration of PF-07248144 [redacted], and should be considered for consultation with a hepatologist and initiation of antiviral therapies (eg, nucleoside antagonist) in accordance with the Japan Society of Hepatology (JSH) Guidelines for the management of Hepatitis B Virus infection.

[redacted]

- [redacted]
- [redacted]
- [redacted]
- [redacted]

090177e19bd138e0\Approved\Approved On: 14-Nov-2022 20:57 (GMT)

[redacted]

[redacted]

- [redacted]
- [redacted]

10.8.1.4. Genetics and Biomarkers

Given the genetic testing is for exploratory purposes only and following Pfizer process, there is no expectation to disclose these genetic testing results to the study participants, at any time.

A study participants participation in a clinical study shall not be conditioned on his or her informed consent for the use of biospecimens that is not related to a clinical endpoint, the study intervention or disease being investigated in the trial, or the inclusion/exclusion criteria for the study.

[redacted]

[redacted]

[redacted]

[redacted]

090177e19bd138e0\Approved\Approved On: 14-Nov-2022 20:57 (GMT)

[redacted]

[redacted]	[redacted]
[redacted]	[redacted]
[redacted]	[redacted]
[redacted]	[redacted]
[redacted]	[redacted]
[redacted]	[redacted]
[redacted]	[redacted]
[redacted]	[redacted]
[redacted]	[redacted]

[redacted]

090177e19bd138e0\Approved\Approved On: 14-Nov-2022 20:57 (GMT)

[redacted]

[redacted]	[redacted]	[redacted]																						[redacted]		[redacted]	[redacted]
		[redacted]										[redacted]		[redacted]													
[redacted]		[redacted]		[redacted]			[redacted]	[redacted]	[redacted]	[redacted]						[redacted]			[redacted]	[redacted]		[redacted]			[redacted]		
[redacted]		[redacted]		[redacted]	[redacted]	[redacted]	[redacted]			[redacted]		[redacted]		[redacted]		[redacted]	[redacted]	[redacted]	[redacted]			[redacted]	[redacted]	[redacted]			
[redacted]			[redacted]										[redacted]		[redacted]										[redacted]		
[redacted]		[redacted]		[redacted]	[redacted]	[redacted]	[redacted]	[redacted]	[redacted]	[redacted]	[redacted]	[redacted]		[redacted]		[redacted]	[redacted]	[redacted]	[redacted]	[redacted]	[redacted]	[redacted]	[redacted]	[redacted]		[redacted]	[redacted]
[redacted]	[redacted]	[redacted]																						[redacted]		[redacted]	
[redacted]	[redacted]	[redacted]				[redacted]			[redacted]	[redacted]	[redacted]	[redacted]		[redacted]				[redacted]			[redacted]	[redacted]	[redacted]	[redacted]		[redacted]	
[redacted]	[redacted]																										
[redacted]	[redacted]																										

090177e19bd138e0\Approved\Approved On: 14-Nov-2022 20:57 (GMT)

[redacted]	[redacted]	[redacted]																						[redacted]		[redacted]	[redacted]
		[redacted]								[redacted]				[redacted]													
[redacted]		[redacted]		[redacted]			[redacted]	[redacted]	[redacted]	[redacted]									[redacted]	[redacted]		[redacted]			[redacted]		
[redacted]		[redacted]		[redacted]	[redacted]	[redacted]	[redacted]			[redacted]				[redacted]		[redacted]	[redacted]	[redacted]	[redacted]			[redacted]	[redacted]	[redacted]			
[redacted]	[redacted]																				[redacted]					[redacted]	
[redacted]	[redacted]	[redacted]				[redacted]	[redacted]					[redacted]		[redacted]				[redacted]	[redacted]					[redacted]		[redacted]	

[redacted]

090177e19bd138e0\Approved\Approved On: 14-Nov-2022 20:57 (GMT)

[redacted]

090177e19bd138e0\Approved\Approved On: 14-Nov-2022 20:57 (GMT)

[redacted]

10.8.3. Korean Specific Requirements Inclusion Criterion #3

Part 2A (Dose Expansion, monotherapy) prior lines of therapy

- Part 2A (dose expansion) prior lines of therapy, participant population for Korea will remain with 3L+. For Korea only: Part 2A (ER+HER2- breast cancer 3L+, monotherapy)**:** Histological or cytological diagnosis of locally advanced or metastatic ER+HER2- breast cancer. Participants must have progressed after at least 1 prior line of CDK4/6 inhibitor and 1 line of endocrine therapy.

10.9. Appendix 9: Detailed Dose Escalation/DeEscalation Scheme for BLRM Design

This appendix provides the details of the statistical model, the description of prior distribution. The results of the Bayesian analyses and respective dosing decisions for some hypothetical data scenarios, and a simulation study of the operating characteristics of the model could be found in the separate Technical Supplementary material to this appendix.

[redacted]

090177e19bd138e0\Approved\Approved On: 14-Nov-2022 20:57 (GMT)

[redacted]

[redacted]

[redacted]

[redacted]

[redacted]

[redacted]

[redacted]			
[redacted]	[redacted]	[redacted]	[redacted]
[redacted]	[redacted]	[redacted]	[redacted]

[redacted]

[redacted]	[redacted]			[redacted]	[redacted]	[redacted]		
	[redacted]	[redacted]	[redacted]			[redacted]	[redacted]	[redacted]
[redacted]	[redacted]	[redacted]	[redacted]	[redacted]	[redacted]	[redacted]	[redacted]	[redacted]
[redacted]	[redacted]	[redacted]	[redacted]	[redacted]	[redacted]	[redacted]	[redacted]	[redacted]
[redacted]	[redacted]	[redacted]	[redacted]	[redacted]	[redacted]	[redacted]	[redacted]	[redacted]
[redacted]	[redacted]	[redacted]	[redacted]	[redacted]	[redacted]	[redacted]	[redacted]	[redacted]
[redacted]	[redacted]	[redacted]	[redacted]	[redacted]	[redacted]	[redacted]	[redacted]	[redacted]
[redacted]	[redacted]	[redacted]	[redacted]	[redacted]	[redacted]	[redacted]	[redacted]	[redacted]
[redacted]	[redacted]	[redacted]	[redacted]	[redacted]	[redacted]	[redacted]	[redacted]	[redacted]
[redacted]	[redacted]	[redacted]	[redacted]	[redacted]	[redacted]	[redacted]	[redacted]	[redacted]

090177e19bd138e0\Approved\Approved On: 14-Nov-2022 20:57 (GMT)

[redacted]

[redacted]

[redacted]

[redacted]

[redacted]

[redacted]

090177e19bd138e0\Approved\Approved On: 14-Nov-2022 20:57 (GMT)

[redacted]

090177e19bd138e0\Approved\Approved On: 14-Nov-2022 20:57 (GMT)

10.10. Appendix 10. Bone Marrow Reserve in Adults

***Adapted from R.E. ELLIS**: The Distribution of Active Bone Marrow in the Adult, Phy. Med. Biol. 5, 255-258, 1961*[33]

Marrow Distribution of the Adult

**SITE**		**MARROW wt. (g)**	**FRACTION RED MARROW AGE 40**	**RED MARROW wt. (g) AGE 40**	**% TOTAL RED MARROW**	
CRANIUM	Head :			136.6		
AND	Cranium	165.8	0.75	124.3	13.1	**13.1**
MANDIBLE	Mandible	16.4	0.75	12.3		
	Upper Limb Girdle :			86.7		
HUMERI, SCAPULAE,	2 Humerus, head & neck	26.5	0.75	20.0	8.3	8.3
CLAVICLES	2 Scapulae	67.4	0.75	50.5		
	2 Clavicles	21.6	0.75	16.2		
	Sternum	39.0	0.6	23.4	2.3	
	Ribs :			82.6		
	1 pair	10.2	All 0.4	4.1		
	2	12.6		5.0		
	3	16.0		6.4		
STERNUM	4	18.6		7.4		
AND	5	23.8		9.5	7.9	**10.2**
RIBS	6	23.6		9.4		
	7	25.0		10.0		
	8	24.0		9.6		
	9	21.2		8.5		
	10	16.0		6.4		
	11	11.2		4.5		
	12	4.6		1.8		
	Sacrum	194.0	0.75	145.6	13.9	
PELVIC BONES	2 os coxae	310.6	0.75	233.0	22.3	**36.2**
FEMUR	2 Femoral head and neck	53.0	0.75	40.0		**3.8**

090177e19bd138e0\Approved\Approved On: 14-Nov-2022 20:57 (GMT)

Marrow Distribution of the Adult (cont'd)

**SITE**		**MARROW wt. (g)**	**FRACTION RED MARROW AGE 40**	**RED MARROW wt. (g) AGE 40**	**% TOTAL RED MARROW**	
	Vertebrae (Cervical):			35.8		
	1	6.6	All 0.75	5.0		
	2	8.4		6.3		
	3	5.4		4.1	3.4	
	4	5.7		4.3		
	5	5.8		4.4		
	6	7.0		5.3		
	7	8.5		6.4		
	Vertebrae (Thoracic):			147.9		
	1 pair	10.8	All 0.75	8.1		
	2	11.7		8.8		
	3	11.4		8.5		
	4	12.2		9.1		
**VERTEBRAE**	5	13.4		10.1	14.1	**28.4**
	6	15.3		11.5		
	7	16.1		12.1		
	8	18.5		13.9		
	9	19.7		14.8		
	10	21.2		15.9		
	11	21.7		16.3		
	12	25.0		18.8		
	Vertebrae (Lumbar) :			114.1		
	1 pair	27.8	All 0.75	20.8		
	2	29.1		21.8	10.9	
	3	31.8		23.8		
	4	32.1		24.1		
	5	31.4		23.6		
**TOTAL**		**1497.7**		**1045.7**	**100.0**	**100.0**

090177e19bd138e0\Approved\Approved On: 14-Nov-2022 20:57 (GMT)

10.11. Appendix 11: RECIST (Response Evaluation Criteria In Solid Tumors) version 1.1 Guidelines

Adapted from E.A. Eisenhauer, et al: New response evaluation criteria in solid tumours: Revised RECIST guideline (version 1.1). European Journal of Cancer 45 (2009) 228–247.[34]

CATEGORIZING LESIONS AT BASELINE

Measurable Lesions

- Lesions that can be accurately measured in at least one dimension.
- Lesions with longest diameter twice the slice thickness and at least 10 mm or greater when assessed by CT or MRI (slice thickness 5-8 mm).
- Lesions with longest diameter at least 20 mm when assessed by Chest X-ray.
- Superficial lesions with longest diameter 10 mm or greater when assessed by caliper.
- Malignant lymph nodes with the short axis 15 mm or greater when assessed by CT.

NOTE: The shortest axis is used as the diameter for malignant lymph nodes, longest axis for all other measurable lesions.

Non-measurable disease

Non-measurable disease includes lesions too small to be considered measurable (including nodes with short axis between 10 and 14.9 mm) and truly non-measurable disease such as pleural or pericardial effusions, ascites, inflammatory breast disease, leptomeningeal disease, lymphangitic involvement of skin or lung, clinical lesions that cannot be accurately measured with calipers, abdominal masses identified by physical exam that are not measurable by reproducible imaging techniques.

- Bone disease: Bone disease is non-measurable with the exception of soft tissue components that can be evaluated by CT or MRI and meet the definition of measurability at baseline.
- Previous local treatment: A previously irradiated lesion (or lesion subjected to other local treatment) is non-measurable unless it has progressed since completion of treatment.

Normal sites

- Cystic lesions: Simple cysts should not be considered as malignant lesions and should not be recorded either as target or non-target disease. Cystic lesions thought to represent cystic metastases can be measurable lesions, if they meet the specific definition above. If non-cystic lesions are also present, these are preferred as target lesions.

090177e19bd138e0\Approved\Approved On: 14-Nov-2022 20:57 (GMT)

[redacted]

- Normal nodes: Nodes with short axis <10 mm are considered normal and should not be recorded or followed either as measurable or non-measurable disease.

Recording Tumor Assessments

All sites of disease must be assessed at baseline. Baseline assessments should be done as close as possible prior to study start. For an adequate baseline assessment, all required scans must be done within 28 days prior to randomization and all disease must be documented appropriately. If baseline assessment is inadequate, subsequent statuses generally should be non-evaluable.

Target Lesions

All measurable lesions up to a maximum of 2 lesions per organ, 5 lesions in total, representative of all involved organs, should be identified as target lesions at baseline. Target lesions should be selected on the basis of size (longest lesions) and suitability for accurate repeated measurements. Record the longest diameter for each lesion, except in the case of pathological lymph nodes for which the short axis should be recorded. The sum of the diameters (longest for non-nodal lesions, short axis for nodal lesions) for all target lesions at baseline will be the basis for comparison to assessments performed on study.

- If two target lesions coalesce the measurement of the coalesced mass is used. If a large target lesion splits, the sum of the parts is used.

- Measurements for target lesions that become small should continue to be recorded. If a target lesion becomes too small to measure, 0 mm should be recorded if the lesion is considered to have disappeared; otherwise a default value of 5 mm should be recorded.

NOTE: When nodal lesions decrease to <10 mm (normal), the actual measurement should still be recorded.

Non-target Disease

All non-measurable disease is non-target. All measurable lesions not identified as target lesions are also included as non-target disease. Measurements are not required but rather assessments will be expressed as CR, Non-CR/Non-PD, PD, Non-evaluable (NE). Multiple non-target lesions in one organ may be recorded as a single item on the case report form (eg, ‘multiple enlarged pelvic lymph nodes’or ‘multiple liver metastases’).

OBJECTIVE RESPONSE STATUS AT EACH EVALUATION

Disease sites must be assessed using the same technique as baseline, including consistent administration of contrast and timing of scanning. If a change needs to be made the case must be discussed with the radiologist to determine if substitution is possible. If not, subsequent objective statuses might be non-evaluable.

090177e19bd138e0\Approved\Approved On: 14-Nov-2022 20:57 (GMT)

Target Disease

1. Complete Response (CR): Complete disappearance of all target lesions with the exception of nodal disease. All target nodes must decrease to normal size (short axis <10 mm). All target lesions must be assessed.

2. Partial Response (PR): Greater than or equal to 30% decrease under baseline of the sum of diameters of all target measurable lesions. The short diameter is used in the sum for target nodes, while the longest diameter is used in the sum for all other target lesions. All target lesions must be assessed.

3. Stable: Does not qualify for CR, PR or Progression. All target lesions must be assessed. Stable can follow PR only in the rare case that the sum increases by less than 20% from the nadir, but enough that a previously documented 30% decrease no longer holds.

4. Objective Progression (PD): 20% increase in the sum of diameters of target measurable lesions above the smallest sum observed (over baseline if no decrease in the sum is observed during therapy), with a minimum absolute increase of 5 mm.

5. Non-evaluable (NE): Progression has not been documented, and

   - One or more target measurable lesions have not been assessed; or

   - One or more target lesions cannot be measured accurately (eg, poorly visible unless due to being too small to measure); or

   - One or more target lesions were excised or irradiated.

Non-target disease

6. CR: Disappearance of all non-target lesions and normalization of tumor marker levels. All lymph nodes must be 'normal' in size (<10 mm short axis).

7. Non-CR/Non-PD: Persistence of any non-target lesions and/or tumor marker level above the normal limits.

8. PD: Unequivocal progression of pre-existing lesions. Generally, the overall tumor burden must increase sufficiently to merit discontinuation of therapy. In the presence of SD or PR in target disease, progression due to unequivocal increase in non-target disease should be rare.

9. NE: Progression has not been determined and one or more non-target sites were not assessed or assessment methods were inconsistent with those used at baseline.

090177e19bd138e0\Approved\Approved On: 14-Nov-2022 20:57 (GMT)

New Lesions

The appearance of any new unequivocal malignant lesion indicates PD. If a new lesion is equivocal, for example due to its small size, continued assessment will clarify the etiology. If repeat assessments confirm the lesion, then progression should be recorded on the date of the initial assessment. A lesion identified in an area not previously scanned will be considered a new lesion.

Supplemental Investigations

- If CR determination depends on a residual lesion that decreased in size but did not disappear completely, it is recommended the residual lesion be investigated with biopsy or fine needle aspirate. If no disease is identified, objective status is CR.

- If progression determination depends on a lesion with an increase possibly due to necrosis, the lesion may be investigated with biopsy or fine needle aspirate to clarify status.

Subjective Progression

Patients requiring discontinuation of treatment without objective evidence of disease progression should not be reported as PD on tumor assessment CRFs. This should be indicated on the end of treatment CRF as off treatment due to Global Deterioration of Health Status. Every effort should be made to document objective progression even after discontinuation of treatment.

Objective Response Status at Each Evaluation

Target Lesions	Non-target Disease	New Lesions	Objective status
CR	CR	No	CR
CR	Non-CR/Non-PD	No	PR
CR	NE or Missing	No	PR
PR	Non-CR/Non-PD, NE or Missing	No	PR
SD	Non-CR/Non-PD, NE or Missing	No	Stable
NE or Missing	Non-PD	No	NE
PD	Any	Yes or No	PD
Any	PD	Yes or No	PD
Any	Any	Yes	PD

090177e19bd138e0\Approved\Approved On: 14-Nov-2022 20:57 (GMT)

If the protocol allows enrollment of patients with only non-target disease, the following table will be used:

Objective Response Status at each Evaluation for Patients with Non-Target Disease

Non-target Disease	New Lesions	Objective status
CR	No	CR
Non-CR/Non-PD	No	Non-CR/Non-PD
NE	No	NE
Unequivocal progression	Yes or No	PD
Any	Yes	PD

Best Overall Response

The best overall response (BOR) is the best response recorded from the randomization until disease progression or death due to any cause. This is derived from the sequence of objective statuses. Objective statuses are not considered after objective progression is documented or after start of the first anticancer treatment post discontinuation of protocol treatment. BOR for each patient will be derived as one of the following categories.

- Complete response (CR): At least one objective status of CR documented before progression.
- Partial response (PR): At least one objective status of PR documented before progression.
- Stable disease (SD): At least one objective status of stable documented at 8 weeks (±7 days) after randomization date and before progression but not qualifying as CR, PR.
- Progressive Disease (PD): Objective status of progression within 16 weeks of randomization, not qualifying as CR, PR or SD.
- Non-evaluable (NE): Progression not documented within 16 weeks after randomization and no other response category applies.

090177e19bd138e0\Approved\Approved On: 14-Nov-2022 20:57 (GMT)

[redacted]

10.12. Appendix 12: ECOG Performance Status*[35]

Grade	ECOG
0	Fully active, able to carry on all pre-disease performance without restriction.
1	Restricted in physically strenuous activity but ambulatory and able to carry out work of a light or sedentary nature, eg, light house work, office work.
2	Ambulatory and capable of all self-care but unable to carry out any work activities. Up and about more than 50% of waking hours.
3	Capable of only limited self-care, confined to bed or chair more than 50% of waking hours.
4	Completely disabled. Cannot carry on any self-care. Totally confined to bed or chair.
5	Dead.

*As published in Am J Clin Oncol 5:649-655, 1982.

090177e19bd138e0\Approved\Approved On: 14-Nov-2022 20:57 (GMT)

10.13. Appendix 13: Alternative Measures During Public Emergencies

The alternative study measures described in this section are to be followed during public emergencies, including the COVID-19 pandemic. This appendix applies for the duration of the COVID-19 pandemic globally and will become effective for other public emergencies only upon written notification from Pfizer.

Use of these alternative study measures are expected to cease upon the return of business as usual circumstances (including the lifting of any quarantines and travel bans/advisories).

10.13.1. Eligibility

While SARS-CoV2 testing is not mandated for this study, local clinical practice standards for testing should be followed. A patient should be excluded if he/she has a positive test result for SARS-CoV2 infection, is known to have asymptomatic infection, or is suspected of having SARS-CoV2. Patients with active infections are excluded from study participation as per Exclusion Criterion #8. When the infection resolves, the patient may be considered for re-screening (See Section 5.4 Screen Failures).

10.13.2. Telehealth Visits

In the event that in-clinic study visits cannot be conducted, every effort should be made to follow up on the safety of study participants at scheduled visits per the Schedule of Activities or unscheduled visits. Telehealth visits may be used to continue to assess participant safety and collect data points. Telehealth includes the exchange of healthcare information and services via telecommunication technologies (eg, audio, video, video-conferencing software) remotely, allowing the participant and the investigator to communicate on aspects of clinical care, including medical advice, reminders, education, and safety monitoring. The following assessments must be performed during a telehealth visit:

- Review and record study intervention(s), including compliance and missed doses.
- Review and record any AEs and SAEs since the last contact. Refer to Section 8.3.
- Review and record any new concomitant medications or changes in concomitant medications since the last contact.
- Review and record contraceptive method and results of pregnancy testing. Confirm that the participant is adhering to the contraception method(s) required in the protocol. Refer to Appendix 4 (Section 10.4 and Appendix 13 (Section 10.13.3.1) of this appendix regarding pregnancy tests.
- Review and discuss safety and efficacy imaging assessment results, if applicable.

Study participants must be reminded to promptly notify site staff about any change in their health status.

090177e19bd138e0\Approved\Approved On: 14-Nov-2022 20:57 (GMT)

10.13.3. Alternative Facilities for Safety Assessments

10.13.3.1. Laboratory Testing

If a study participant is unable to visit the site for protocol-specified safety laboratory evaluations, testing may be conducted at a local laboratory if permitted by local regulations. The local laboratory may be a standalone institution or within a hospital. The following safety laboratory evaluations may be performed at a local laboratory:

- Hematology;
- Blood chemistry;
- Coagulation;
- Urinalysis;
- Pregnancy test;
- PSA (for prostate cancer participants only).

If a local laboratory is used, qualified study site personnel must order, receive, and review results. Site staff must collect the local laboratory reference ranges and certifications/ accreditations for filing at the site. Laboratory test results are to be provided to the site staff as soon as possible. The local laboratory reports should be filed in the participant's source documents/medical records. Relevant data from the local laboratory report should be recorded on the CRF.

If a participant requiring pregnancy testing cannot visit a local laboratory for pregnancy testing, a home urine pregnancy testing kit with a sensitivity of at least 25 IU/mL may be used by the participant to perform the test at home, if compliant with local regulatory requirements. The pregnancy test outcome should be documented in the participant's source documents/medical records and relevant data recorded on the CRF. Confirm that the participant is adhering to the contraception method(s) required in the protocol.

10.13.3.2. Imaging

If the participant is unable to visit the study site for safety imaging assessment(s), the participant may visit an alternative facility to have the safety imaging assessment(s) performed. Qualified study site personnel must order, receive, and review results.

10.13.3.3. Electrocardiograms

If the participant is unable to visit the study site for ECGs, the participant may visit an alternative facility to have the ECGs performed. Qualified study site personnel must order, receive, and review results.

090177e19bd138e0\Approved\Approved On: 14-Nov-2022 20:57 (GMT)

10.13.3.4. Study Intervention

If the safety of a trial participant is at risk because they cannot complete required evaluations or adhere to critical mitigation steps, then discontinuing that participant from study intervention must be considered.

PF-07248144 may be shipped by courier to study participants if permitted by local regulations and in accordance with storage and transportation requirements for PF-07248144. Pfizer does not permit the shipment of PF-07248144 by mail. The tracking record of shipments and the chain of custody of PF-07248144 must be kept in the participant's source documents/medical records. Further instructions are provided in the IP manual

The following is recommended for the administration of PF-07248144 for participants who have active confirmed (positive by regulatory authority-approved test) or presumed (test pending/clinical suspicion) SARS-CoV2 infection:

- For symptomatic participants with active SARS-CoV2 infection, PF-07248144 should be delayed for at least 14 days from the start of symptoms. This delay is intended to allow the resolution of symptoms of SARS-CoV2 infection.
- Prior to restarting treatment, the participant should be afebrile for 72 hours, and SARS-CoV2-related symptoms should have recovered to ≤ Grade 1 for a minimum of 72 hours. Notify the study team when treatment is restarted.
- Continue to consider potential drug-drug interactions as described in Section 6.5 for any concomitant medication administered for treatment of SARS-CoV2 infection.

10.13.3.5. Home Health Visits

A home health care service may be considered to facilitate scheduled visits per the Schedule of Activities only after sponsor's approval. Home health visits include a healthcare provider conducting an in-person study visit at the participant's location, rather than an in-person study visit at the site. The following may be performed during a home health visit:

- Physical Exams;
- Vital Signs (including height and weight);
- Safety laboratory blood draws (including hematology, blood chemistry, coagulation);
- Urinalysis;
- Blood draw for PSA analysis (for prostate cancer participants only);
- Blood draw for PK and PD;
- ECGs, if available;

090177e19bd138e0\Approved\Approved On: 14-Nov-2022 20:57 (GMT)

- Also all assessments included in Section 10.13.2 Telehealth Visits.

10.13.4. Adverse Events and Serious Adverse Events

If a participant has COVID-19 during the study, this should be reported as an AE or SAE and appropriate medical intervention provided. Temporary discontinuation of the study intervention may be medically appropriate until the participant has recovered from COVID-19.

It is recommended that the investigator discuss temporary or permanent discontinuation of study intervention with the study medical monitor.

10.13.5. Efficacy Assessments

Please contact the sponsor should a participant need to use alternative measures for efficacy assessments. A plan will be devised in discussion with the sponsor and investigator.

10.13.6. Independent Oversight Committees

This is an open-label, non-randomized Phase 1 study. This study will not use a DMC.

090177e19bd138e0\Approved\Approved On: 14-Nov-2022 20:57 (GMT)

[redacted]

The prohibited concomitant medications listed below should not be taken with PF--07248144 for the period of time at least equal to the required washout period listed in Table 17, and throughout the conduct of the study. The Pfizer study team is to be notified of any prohibited medications taken during the study. After consulting with the sponsor, the investigator will make a judgement on the ongoing participation of any participant with prohibited medication use during the study.

This list of drugs prohibited for potential DDI concerns with the IMP (investigational medicinal product) may be revised during the course of the study with written notification from sponsor, to include or exclude specific drugs or drug categories for various reasons (eg, emerging DDI results for the IMP, availability of new information in literature on the DDI potential of other drugs). This is not an all-inclusive list. [redacted]

[redacted]

[redacted]	[redacted]	[redacted]
[redacted]	[redacted]	[redacted]

090177e19bd138e0\Approved\Approved On: 14-Nov-2022 20:57 (GMT)

[redacted]

[redacted]	[redacted]	[redacted]
[redacted]	[redacted]	[redacted]
[redacted]	[redacted]	[redacted]
[redacted]	[redacted]	[redacted]

[redacted]

090177e19bd138e0\Approved\Approved On: 14-Nov-2022 20:57 (GMT)

[redacted]

[redacted]

[redacted]

[redacted]

[redacted]

[redacted]	[redacted]		[redacted]
[redacted]	[redacted]	[redacted]	
	[redacted]	[redacted]	[redacted]

090177e19bd138e0\Approved\Approved On: 14-Nov-2022 20:57 (GMT)

[redacted]

[redacted]	[redacted]		[redacted]
	[redacted]	[redacted]	
[redacted]	[redacted]	[redacted]	
	[redacted]	[redacted]	[redacted]
[redacted]		[redacted]	[redacted]
[redacted]		[redacted]	[redacted]

090177e19bd138e0\Approved\Approved On: 14-Nov-2022 20:57 (GMT)

[redacted]

[redacted]	[redacted]			[redacted]
		[redacted]		
[redacted]	[redacted]	[redacted]	[redacted]	[redacted]
[redacted]	[redacted]			
[redacted]	[redacted]		[redacted]	
[redacted]	[redacted]			

[redacted]

[redacted]

090177e19bd138e0\Approved\Approved On: 14-Nov-2022 20:57 (GMT)

[redacted]

[redacted]	[redacted]	[redacted]		
[redacted]	[redacted]	[redacted]	[redacted]	[redacted]
[redacted]	[redacted]	[redacted]	[redacted]	[redacted]
[redacted]	[redacted]	[redacted]	[redacted]	[redacted]
[redacted]	[redacted]	[redacted]	[redacted]	[redacted]

090177e19bd138e0\Approved\Approved On: 14-Nov-2022 20:57 (GMT)

[redacted]

[redacted]	[redacted]	[redacted]		
[redacted]	[redacted]	[redacted]	[redacted]	[redacted]
[redacted]	[redacted]		[redacted]	[redacted]
[redacted]		[redacted]	[redacted]	[redacted]

[redacted]

090177e19bd138e0\Approved\Approved On: 14-Nov-2022 20:57 (GMT)

10.16. Appendix 16: Abbreviations

The following is a list of abbreviations that may be used in the protocol.

Abbreviation	Term
1L	first-line
2L	second-line
3L	third-line
4L	forth-line
ADME	absorption, distribution, metabolism, and excretion
AE	adverse event
AE%	percentage of unchanged drug excreted in urine
AIDS	acquired immunodeficiency syndrome
Alk Phos	alkaline phosphatase
ALT	alanine aminotransferase
AML	acute myeloid leukemia
ANC	absolute neutrophil count
anti-HBc	total hepatitis B core antibody
anti-HBs	hepatitis B surface antibody
ASCO	American Society of Clinical Oncology
AST	aspartate aminotransferase
AUC	area under the curve
AUC_{24}	AUC from time zero to 24 hours
AUC_{inf}	AUC from time zero to infinity
AUC_{last}	AUC from time zero to time of last measurable concentration
$AUC_{,ss}$	steady state AUC
$AUC_{\tau,sd}$	stable disease AUC during a dosage interval (τ)
$AUC_{\tau,ss}$	steady state AUC during a dosage interval (τ)
AV	Atrioventricular
BAL	Bronchoalveolar lavage
BCRP	breast cancer resistance protein
BC	breast cancer
BET	Bromodomain and Extra-Terminal motif
BID	twice daily
BLRM	Bayesian Logistic Regression Model
BOR	best overall response
BP	blood pressure
Bpm	beats per minute
BUN	blood urea nitrogen
BVN	bivariate normal
C1D1	Cycle 1 Day 1
C1D8	Cycle 1 day 8
C1D15	Cycle 1 Day 15
C2D1	Cycle 2 Day 1

090177e19bd138e0\Approved\Approved On: 14-Nov-2022 20:57 (GMT)

Abbreviation	Term
C3D1	Cycle 3 day 1
CBC	complete blood count
CBR	clinical benefit rate
CDK	cyclin-dependent kinases
C_{eff}	pharmacologically active concentration
CEP17	chromosome enumeration probe 17
cfDNA	cell-free DNA
CFR	Code of Federal Regulations
CHF	congestive heart failure
CI	confidence interval
CIOMS	Council for International Organizations of Medical Sciences
CISH	chromogenic in situ hybridization
CK	creatine kinase
CL	Clearance
CL/F	apparent total clearance
CL_r	Renal clearance
CL_{ss}/F	steady-state apparent total clearance
Clplasma	plasma clearance
C_{max}	maximum observed concentration
$C_{max,ss}$	steady-state C_{max}
C_{min}	minimum observed concentration
$C_{min,ss}$	steady-state C_{min}
CO2	Carbon dioxide
CoA	coenzyme A
CONSORT	Consolidated Standards of Reporting Trials
COVID-19	coronavirus disease 2019
CR	complete response
CRC	colorectal cancer
CREBBP	cAMP response element-binding protein
CRF	case report form
CRISPR	clustered regularly interspaced short palindromic repeats
CRO	contract research organization
CRP	c-reactive protein
CRPC	castration-resistant prostate cancer
CSF	colony stimulating factor
CSR	clinical study report
CT	clinical trial; computed tomography
CTCAE	Common Terminology Criteria for Adverse Events
ctDNA	circulating tumor DNA
CV	coefficient of variation
Cyclins D1, D2, and D3	D-type cyclins

090177e19bd138e0\Approved\Approved On: 14-Nov-2022 20:57 (GMT)

Abbreviation	Term
[redacted]	[redacted]
DDI	drug-drug interaction
DIL	Dear Investigator Letter
DILI	Drug-induced liver injury
DISH	dual in situ hybridization
DLT	Dose-limiting toxicity
DMC	data monitoring committee
DNA	deoxyribonucleic acid
DOR	duration of response
DRE	disease-related event
EC	ethics committee
ECG	Electrocardiogram
ECOG	Eastern Cooperative Oncology Group
eCRF	electronic case report form
EDP	exposure during pregnancy
EDTA	ethylenediaminetetraacetic acid
EMA	European Medicines Agency
EOT	end of treatment
EP300	adenoviral EIA-associated protein p300
ER	estrogen receptor
ESR1	estrogen receptor 1
ET	endocrine therapy
EU	European Union
EudraCT	European Clinical Trials Database
EWOC	escalation with overdose control
F_a	fraction of drug absorbed.
FDA	Food and Drug Administration (United States)
FFPE	formalin-fixed paraffinembedded
FIH	first-in-human
FISH	fluorescence in situ hybridization
FSH	Follicle stimulating hormone
GALT	gut-associated lymphoid tissue
GCP	Good Clinical Practice
G-CSF	granulocyte colony-stimulating factor
GFR	glomerular filtration rate
GGT	gamma-glutamyl transferase
GI	Gastrointestinal
GLP	Good Laboratory Practice
GNAT	general control non-derepressible 5 (Gcn5) –related N-acetyltransferases
H3K23Ac	KAT6A and KAT6B cell biomarker
HAT	histone acetyltransferases
HbcAb	hepatitis B core antibody

090177e19bd138e0\Approved\Approved On: 14-Nov-2022 20:57 (GMT)

Abbreviation	Term
HbsAg	hepatitis B surface antigen
HBV	hepatitis B virus
HCV	hepatitis C virus
HCVAb	hepatitis C antibody
HDAC	Histone deacetylase
HER2	human epidermal growth factor receptor 2
[redacted]	[redacted]
HIPAA	Health Insurance Portability and Accountability Act
HIV	human immunodeficiency virus
HNSTD	highest non-severely toxic dose
HR	heart rate; hormone replacement; hormone receptor
HRT	hormone replacement therapy
IB	Investigator's Brochure
ICD	informed consent document
ICH	International Council for Harmonisation
IgM anti-HBc	immunoglobulin M antibody to hepatitis B core antigen
IHC	immunohistochemistry
IL	Interleukin
[redacted]	[redacted]
IMP	investigational medicinal product
IND	Investigational New Drug
INR	international normalized ratio
IP manual	investigational product manual
IPAL	Investigational Product Accountability Log
IRB	Institutional Review Board
IRC	Independent Review Committee
JSH	Japan Society of Hepatology
KAT	lysine acetyltransferase
Ki-67	antigen Ki-67
LBBB	left bundle branch block
LDH	lactate dehydrogenase
LFT	liver function test
LHRH	luteinizing hormone releasing hormone
LMWH	low-molecular-weight heparin
MAP	meta-analytic-predictive
mBC	metastatic BC
MD	multiple dose
MEC	molar extinction coefficient
MedDRA	Medical Dictionary for Regulatory Activities
M/E (ratio)	myeloid to erythroid
mITT	modified intent to treat
MDR1	multidrug resistance mutation

090177e19bd138e0\Approved\Approved On: 14-Nov-2022 20:57 (GMT)

Abbreviation	Term
MRD	Minimal residual disease
MRI	magnetic resonance imaging
MTD	maximum tolerated dose
N/A	not applicable
NCCN	National Comprehensive Cancer Network
NCI	National Cancer Institute
NCOA2	nuclear receptor coactivator 2
NCOA3	nuclear receptor coactivator 3
NE	non-evaluable
NHT	novel hormonal therapies
NIMP	non-investigational medicinal product
NOAEL	no-observed-adverse-effect level
NSCLC	non-small cell lung cancer
[redacted]	[redacted]
OR	objective response
ORR	overall response rate
OS	overall survival
PACL	Protocol Administrative Change Letter
PBPK	physiologically based pharmacokinetic
PCWG3	prostate cancer working group 3
PD	pharmacodynamics(s)
PD	progressive disease
PDX	Patient derived xenografts
PET	positron emission tomography
PFS	progression-free survival
[redacted]	[redacted]
PGx	Pharmacogenomics
PI	principal investigator
PK	pharmacokinetic(s)
PMDA	Pharmaceuticals and Medical Devices Agency
PO	per os (by mouth)
[redacted]	[redacted]
PR	partial response
PR	pulse rate
PS	performance status
PSA	prostate specific antigen
PT	prothrombin time
PTT	partial thromboplastin time
aPTT	activated partial thromboplastin time
PVC	premature ventricular contraction/complex
Q2D	every 2 days
QD	every day

090177e19bd138e0\Approved\Approved On: 14-Nov-2022 20:57 (GMT)

Abbreviation	Term
QTc	corrected QT
QTcB	corrected QT (Bazett method)
QTcF	corrected QT (Fridericia method)
R_{ac}	accumulation ratio
[redacted]	[redacted]
RDE	recommended dose for expansion
RECIST	Response Evaluation Criteria in Solid Tumors
RNA	ribonucleic acid
RNAi	RNA interference
RP2D	recommended phase 2 dose
RR	response rate
SAE	serious adverse event
SAP	Statistical Analysis Plan
SARS-CoV-2	severe acute respiratory syndrome coronavirus 2
SC	subcutaneous
SD	stable disease
SERD	selective estrogen receptor degrader
shRNA	short hairpin RNA
SISH	silver-enhanced in situ hybridization
SoA	schedule of activities
SOC	standard of care
SOP	standard operating procedure
[redacted]	[redacted]
SRSD	single reference safety document
STD10	death or irreversible severe toxicity in 10% of rodents (eg 1 out of 10 rats)
SUSAR	suspected unexpected serious adverse reaction
$t_{1/2}$	terminal elimination half-life
TBD	to be determined
TBili	total bilirubin
TBR	tumor background ratio
TEAE	Treatment emergent adverse event
TGI	tumor growth inhibition
TID	three times daily
[redacted]	[redacted]
TNF	tumor necrosis factor
T_{max}	time to maximum concentration
$T_{max,ss}$	steady-state T_{max}
TSC	tumor stasis concentration
TSH	thyroid-stimulating hormone

090177e19bd138e0\Approved\Approved On: 14-Nov-2022 20:57 (GMT)

Abbreviation	Term
TTP	time to progression
UGT	uridine 5' diphosphate-glucuronosyltransferase
ULN	upper limit of normal
US	United States
USPI	United States Package Insert
UVB	ultraviolet B
V_{ss}	steady-state volume of distribution
V_{ss}/F	apparent volume of distribution at steady state
V_{z}/F	apparent volume of distribution during the terminal phase
WBC	white blood cell
WOCBP	woman of childbearing potential

090177e19bd138e0\Approved\Approved On: 14-Nov-2022 20:57 (GMT)

10.17. Appendix 17: Protocol Amendment History

The Protocol Amendment Summary of Changes Table for the current amendment is located directly before the Table of Contents. The protocol amendment summary of changes table for past amendments can be found below:

**Document History:**		
**Document**	**Version Date**	**Summary and Rationale for Changes**
Amendment 3	14 December 2021	Overall Rationale for the Amendment: The purpose of this amendment is to include details of Part 2B combination dose expansion with fulvestrant, update the participant population for Part 2A from at least 3 prior lines of therapy to at least 2 prior lines of therapy, and incorporate prior changes specified in the Protocol Administrative Change Letter (PACL). Additionally, administrative corrections and clarifications were made throughout the protocol to improve internal consistency.<br><br>Specific updates are summarized below.<br>• Section 1.2, Schema: Updated description of Parts 2A and 2B.<br>• Section 1.3, Schedule of Activities: [redacted] Fulvestrant administration information was updated.<br>• Section 1.3, Schedule of Activities: PK Sampling, PD/Other Biomarker Sampling (for Parts 1A, 1B, [redacted]<br>• Section 1.3, Schedule of Activities: Dose Expansion (Part 2): [redacted] Fulvestrant administration information was updated; [redacted] Part 2 (metabolite profiling substudy) clarified that the evaluation for urine PK and metabolite profiling in both blood and urine should be conducted in the same participant; For Part 2: the timing of ECG collection was added to footnote m.

090177e19bd138e0\Approved\Approved On: 14-Nov-2022 20:57 (GMT)

Document History:		
**Document**	**Version Date**	**Summary and Rationale for Changes**
		• Section 1.3, Schedule of Activities: PK Sampling, PD/Other Biomarker Sampling (For Parts 2A [for participants not participating in food effect substudy] and 2B): A separate PK table for Part 2 (non-food effect) was created; Cycle 1 Day 8 blood collection for PK and Biomarker PD sampling was removed; 4 hour time point on Cycle 1 Day 15 for PF-07428144 PK blood plasma sampling in Part 2A was added; Text was added specifying that urine collection for PK and metabolite profiling and blood samples for metabolite profiling are to be collected from the same subset of participants in Part 2A; [redacted].<br>• Section 1.3, Schedule of Activities: PK Sampling Schedule in Participants in the Food Effect Subset of Part 2A (Monotherapy Dose Expansion): [redacted]<br>• Section 2.2.4, Clinical Overview: Updated to include clinical data from the ongoing Part 1A monotherapy dose escalaton study.<br>• Section 1.1 and 3, Objectives and Endpoints: [redacted] Objective and endpoint were added for Part 2A to evaluate urine PK of PF-07248144; [redacted].<br>• Section 4.1, Overall design: [redacted]<br>• Section 5, Inclusion/Exclusion Criteria: Inclusion criteria 1 was updated to include participants enrolled at clinical sites in South Korea: adult participants age ≥19 years; Inclusion criterion 3 was modified for Part 2A to update the participant population from at least 3 prior lines of therapy to at least 2 prior lines of therapy;

090177e19bd138e0\Approved\Approved On: 14-Nov-2022 20:57 (GMT)

Document History:		
**Document**	**Version Date**	**Summary and Rationale for Changes**
		Inclusion criterion 3 was modified to include detailed inclusion criteria for Part 2B; Inclusion criterion 12 was modified to allow higher levels of AST/ALT (≤5x ULN instead of ≤3x) if there is tumor liver involvement for Part 2; Exclusion criterion 6,washout period for systemic anti-cancer therapy was reduced from 4 weeks to 3 weeks.<br>• [redacted]<br>• Section 6.3.1, Allocation to Study Intervention: Details of allocation to study intervention via IRT were added for Part 2<br>• Section 8.2.2, [redacted]<br>• [redacted]<br>• Section 8.5.2, Urine for Analysis of PF-07248144 Concentrations and Metabolite Profiling: Urine analysis at screening and for 24 hours post-dose on Cycle 1 Day 15 was expanded to include a sub set of all participants in Part 2A (including those in the food effect subset); Text was added to clarify that the sub set of participants from whom urine samples for PK and metabolite profiling will be collected are the same as those from whom blood samples for metabolite profiling will be collected; Clarification was added that the urine samples are shipped to 2 separate labs.<br>• Section 8.5.3, Blood for Metabolite Profiling of PF0748144: Blood samples for metabolite profiling at Screening and on

090177e19bd138e0\Approved\Approved On: 14-Nov-2022 20:57 (GMT)

090177e19bd138e0\Approved\Approved On: 14-Nov-2022 20:57 (GMT)

Document History:		
**Document**	**Version Date**	**Summary and Rationale for Changes**
		Cycle 1 Day 15 was expanded to include a sub set of all participants in Part 2A (including those in the food effect subset); Text was added to clarify that the subset of participants from whom blood samples for metabolite profiling will be collected are the same subset from whom urine samples for PK and metabolite profiling will be collected.<br>• Section 9.2.2, Part 2 Dose Expansion: Computation for sample size determination for Part 2 was updated.<br>• [redacted]<br>• Global: Expansion RP2D was changed to RDE where applicable; Alternative dosing regimen examples for PF-07248144 was updated from BID and/or TID to intermittent dosing.
Amendment 2	03 August 2021	The purpose of this amendment is to incorporate changes/clarifications specified in the Japan specific Dear Investigator Letter (DIL) and the PACL into protocol Appendix 10.8.1 Japan Specific Requirements after discussion with the Pharmaceuticals and Medical Devices Agency (PMDA). The changes in Appendix 10.8.1 are only applicable to the Japanese sites and summarized below:<br><br>Additional exclusion criteria (24 and 25).<br><br>Modifications of clinical and laboratory monitoring.<br><br>[redacted]<br><br>Added clarification regarding Japanese participants participation in Part 1B, [redacted] and Part 2.

Document History:		
**Document**	**Version Date**	**Summary and Rationale for Changes**
		Added clarification regarding the administration interval between the first and second Japanese participants.<br><br>[redacted]<br><br>Added clarification regarding the exclusion criteria for Hepatitis B and the monitoring for Hepatitis B Virus (HBV).<br><br>[redacted]
Amendment 1	11 September 2020	The primary purpose of this amendment is to incorporate changes/clarifications requested by the FDA following agency review of the original final protocol dated 13 July 2020.<br><br>In addition, other clarifications, administrative, and typographical modifications were made.
		Section 1.3 Schedules of Activities.<br><br>• Pharmacokinetic (for Parts 1A, 1B, [redacted] and Part 2 [for participants not participating in food effect]) Sampling, Pharmacodynamic/other Biomarker Sampling, and ECG Assessments Table:<br><br>• PF-07248144 urine collection for PK (Part 2A, participants <u>NOT</u> participating in food effect subset) row and Section 8.5.2: Added sample collection at Screening.<br><br>• Blood Sample for metabolite profiling (Part 2A, participants <u>NOT</u> participating in food effect subset) row: Clarified that the sample will be taken at Screening and not Cycle 1 Day 1.

090177e19bd138e0\Approved\Approved On: 14-Nov-2022 20:57 (GMT)

Document History:		
**Document**	**Version Date**	**Summary and Rationale for Changes**
		• Footnote d: Updated to add more clarification around the timing of collection of the PK blood draws.
		Section 2.2.3 Nonclinical Safety and Section 2.3.1 Risk Assessment: Updated text to include reproductive system as a key toxicity of potential clinical importance. Provided details of findings in nonclinical studies increased clarity and added effective contraception as mitigation strategy.
		• Section 4.3.3 Criteria for Dose Escalation<br>• Last paragraph of section: Removed 'unless agreed by both investigator and sponsor' as intra-participant dose escalation will not be permitted in this study.
		• Section 4.3.4 Dose Limiting Toxicity Definition.<br>• Non-Hematologic Dose-Limiting Toxicities, Fourth bullet: Updated DLT as follows: For participants with Grade 2 hepatic transaminase or alkaline phosphatase levels at baseline as a result of liver metastasis or bone metastasis, AST or ALT >8 x ULN or AST or ALT >5 x ULN for ≥14 days will be considered as a DLT.<br>• Added DLT language regarding G5 adverse event: Any Grade 5 AE (death) not clearly due to either the underlying disease or other etiologies is a DLT.
		Section 5.1 Inclusion Criteria.<br><br>Added the following:<br>4. Participants with ER+HER2- advanced or metastatic breast cancer must have documentation

090177e19bd138e0\Approved\Approved On: 14-Nov-2022 20:57 (GMT)

[redacted]

Document History:		
**Document**	**Version Date**	**Summary and Rationale for Changes**
		of ER-positive tumor (≥1% positive stained cells) based on most recent tumor biopsy (unless non-measurable disease where most recent documentation will be provided) utilizing an assay consistent with local standards.<br><br>5. Participants with ER+HER2- advanced or metastatic breast cancer must have documentation of HER2-negative tumor: HER2-negative tumor is determined as immunohistochemistry score 0/1+ or negative by in situ hybridization (FISH/CISH/SISH/DISH) defined as a HER2/CEP17 ratio <2 or for single probe assessment a HER2 copy number <4.<br><br>6. Female participants with ER+HER2- advanced or metastatic breast cancer considered to be of childbearing potential (or have tubal ligations only) must be willing to undergo medically induced menopause by treatment with the approved LHRH agonist such as goserelin, leuprolide or equivalent agents to induce chemical menopause.<br><br>7. Female participants with ER+HER2- advanced or metastatic breast cencer of nonchildbearing potential must meet at least 1 of the following criteria of achieving postmenopausal status, defined as follows:<br><br>• Cessation of regular menses for at least 12 consecutive months with no alternative pathological or physiological cause; [status may be confirmed with/and have] a serum FSH level confirming the post menopausal state;<br><br>• Have undergone a documented hysterectomy and/or bilateral oophorectomy;<br><br>• Have medically confirmed ovarian failure.

090177e19bd138e0\Approved\Approved On: 14-Nov-2022 20:57 (GMT)

[redacted]

Document History:		
**Document**	**Version Date**	**Summary and Rationale for Changes**
		All other female participants (including female participants with tubal ligations) are considered to be of childbearing potential.<br><br>Inclusion criterion # 12 Adequate Liver Function, including, item b. changed the range from ≤5 to ≤3 x ULN for AST and ALT if there is liver involvement by the tumor.
Original protocol	13 July 2020	N/A

090177e19bd138e0\Approved\Approved On: 14-Nov-2022 20:57 (GMT)

11. REFERENCES

1. Scher HI, Morris MJ, Stadler WM, et al. Trial design and objectives for castration-resistant prostate cancer: Updated recommendations from the prostate cancer clinical trials working group 3. J Clin Oncol. 2016;34(12):1402-18.

2. FASLODEX® (fulvestrant) Package Insert. Wilmington, DE; AstraZeneca Pharmaceuticals LP; 2019. Available at: https://www.accessdata.fda.gov/drugsatfda_docs/label/2019/021344Orig1s039lbl.pdf accessed 12 July 2020.

3. Lee KK, Workman JL. Histone acetyltransferase complexes: one size doesn't fit all. Nat Rev Mol Cell Biol. 2007;8(4):284-95.

4. Roth SY, Denu JM, Allis CD. Histone acetyltransferases. Annu Rev Biochem. 2001;70:81-120.

5. Furdas SD, Kannan S, Sippl W, et al. Small molecule inhibitors of histone acetyltransferases as epigenetic tools and drug candidates. Arch Pharm (Weinheim). 2012;345(1):7-21.

6. Voss AK, Thomas T. Histone lysine and genomic targets of histone acetyltransferases in bammals. Bioessays. 2018;40(10):e1800078.

7. Huang F, Abmayr SM, Workman JL. Regulation of KAT6 Acetyltransferases and Their Roles in Cell Cycle Progression, Stem Cell Maintenance, and Human Disease. Mol Cell Biol. 2016;36(14):1900-7.

8. Baell JB, Leaver DJ, Hermans SJ, et al. Inhibitors of histone acetyltransferases KAT6A/B induce senescence and arrest tumour growth. Nature. 2018;560(7717):253-57.

9. Sheikh BN, Phipson B, El-Saafin F, et al. MOZ (MYST3, KAT6A) inhibits senescence via the INK4A-ARF pathway. Oncogene 2015;34(47):5807-20.

10. Hu Z, Zhou J, Jiang J, et al. Genomic characterization of genes encoding histone acetylation modulator proteins identifies therapeutic targets for cancer treatment. Nat Commun. 2019;10(1):733.

11. Borrow J, Stanton VP, Jr., Andresen JM, et al. The translocation t(8;16)(p11;p13) of acute myeloid leukaemia fuses a putative acetyltransferase to the CREB-binding protein. Nat Genet. 1996;14(1):33-41.

12. Shima H, Yamagata K, Aikawa Y, et al. Bromodomain-PHD finger protein 1 is critical for leukemogenesis associated with MOZ-TIF2 fusion. Int J Hematol. 2014;99(1):21-31.

090177e19bd138e0\Approved\Approved On: 14-Nov-2022 20:57 (GMT)

13. Adelaide J, Chaffanet M, Imbert A, et al. Chromosome region 8p11-p21: refined mapping and molecular alterations in breast cancer. Genes Chromosomes Cancer. 1998;22(3):186-99.

14. Turner-Ivey B, Guest ST, Irish JC, et al. KAT6A, a chromatin modifier from the 8p11-p12 amplicon is a candidate oncogene in luminal breast cancer. Neoplasia. 2014;16(8):644-55.

15. Yu L, Liang Y, Cao X, et al. Identification of MYST3 as a novel epigenetic activator of ERalpha frequently amplified in breast cancer. Oncogene. 2017;36(20):2910-18.

16. Tsherniak A, Vazquez F, Montgomery PG, et al. Defining a cancer dependency map. Cell. 2017;170(3):564-76 e16.

17. Zack TI, Schumacher SE, Carter SL, et al. Pan-cancer patterns of somatic copy number alteration. Nat Genet. 2013;45(10):1134-40.

18. Northcott PA, Nakahara Y, Wu X, et al. Multiple recurrent genetic events converge on control of histone lysine methylation in medulloblastoma. Nat Genet. 2009;41(4):465-72.

[redacted]

[redacted]

21. Yan F, Li J, Milosevic J, et al. KAT6A and ENL Form an Epigenetic Transcriptional Control Module to Drive Critical Leukemogenic Gene-Expression Programs. Cancer Discov. 2022 Mar 1;12(3):792-811. doi: 10.1158/2159-8290.CD-20-1459. PMID: 34853079; PMCID: PMC8916037.

[redacted]

23. National Cancer Society. Cancer Facts and Figures 2020. Available from: https://www.cancer.org/research/cancer-facts-statistics/all-cancer-facts-figures/cancer-facts-figures-2020.html#:~:text=Cancer%20Facts%20%26%20Figures%202020%20is%20an%20educational,States.%29%20Current%20cancer%20incidence%2C%20mortality%2C%20and%20survival%20statistics Accessed on: 02 July 2020.

24. de Bono JS, Logothetis CJ, Molina A, et al. Abiraterone and increased survival in metastatic prostate cancer. The New England journal of medicine. 2011;364(21):1995-2005.

090177e19bd138e0\Approved\Approved On: 14-Nov-2022 20:57 (GMT)

25. Scher HI, Fizazi K, Saad F, et al. Increased Survival with Enzalutamide in Prostate Cancer after Chemotherapy. 2012;367(13):1187-97.

26. NCCN. National Comprehensive Cancer Network: NCCN Guielines - Non-Small Cell Lung Cancer. Version 6.2020. June 15 2020.

27. Department of Pharmaceutics University of Washington. (2020). Metabolism and Transport, Drug Interaction Database. Available from: https://www.druginteractionsolutions.org/solutions/drug-interaction-database/. Accessed on: 11 June 2020.

28. Smith TJ, Bohlke K, Lyman GH, et al. Recommendations for the Use of WBC Growth Factors: American Society of Clinical Oncology Clinical Practice Guideline Update. J Clin Oncol. 2015;33(28):3199-212.

29. Lindeman GJ, Bowen R, Joanna Jerzak K, et al. Results from VERONIA: A randomized, Phase II study of second-/third-line venetoclax (VEN) + fulvestrant (F) versus F alone in estrogen receptor (ER)-positive, Her2-negative, locally advanced, or metastatic breast cancer (LA/MBC). J Clin Oncol. 2021;39(15_suppl):1004.

30. Bardia A, Hurvitz SA, DeMichele A, et al. Triplet therapy (continuous ribociclib, everolimus, exemestane) in HR+/HER2− advanced breast cancer postprogression on a CDK4/6 inhibitor (TRINITI-1): Efficacy, safety, and biomarker results. 2019;37(15_suppl):1016-16.

31. Rugo HS, Lerebours F, Ciruelos E, et al. Alpelisib plus fulvestrant in PIK3CA-mutated, hormone receptor-positive advanced breast cancer after a CDK4/6 inhibitor (BYLieve): one cohort of a phase 2, multicentre, open-label, non-comparative study. Lancet Oncol. 2021;22(4):489-98.

32. Neuenschwander B, Matano A, Tang Z, Roychoudhury S, Wandel S and Bailey S. A Bayesian Industry Approach to Phase I Combination Trials in Oncology. In Statistical Methods in Drug Combination Studies. Zhao W and Yang H (eds), Chapman & Hall/CRC, 2014:96-132.

33. R.E. Eliss. The distribution of active bone marrow in the adult, Phy Med Biol. 1961; 5, (255)258.

34. E.A. Eisenhauer, et al: New response evaluation criteria in solid tumours: Revised RECIST guideline (version 1.1). European Journal of Cancer. 2009;(45):228–247.

35. Oken, MM, Creech RH, Tormey, D, et al: Toxicity and response criteria of the Eastern Cooperative Oncology Group. Amer J of Clin Oncol. 1982;(5):649-656.

090177e19bd138e0\Approved\Approved On: 14-Nov-2022 20:57 (GMT)

[redacted]

Protocol C4551001

A PHASE 1 DOSE ESCALATION AND EXPANSION STUDY TO EVALUATE SAFETY, TOLERABILITY, PHARMACOKINETIC, PHARMACODYNAMIC, AND ANTI-TUMOR ACTIVITY OF PF-07248144 IN PARTICIPANTS WITH ADVANCED OR METASTATIC SOLID TUMORS

Statistical Analysis Plan
(SAP)

Version: 2

Date: 22 MAY 2023

090177e19dab896e\Approved\Approved On: 07-Jun-2023 12:23 (GMT)

TABLE OF CONTENTS

LIST OF TABLES 5

VERSION HISTORY 6

1. INTRODUCTION 6

1.1. Study Objectives 6

1.1.1. Primary Objective(s) 6

1.1.1.1. Part 1A: Monotherapy Dose Escalation 6

1.1.1.2. Part 1B: Combination Dose Escalation 7

[redacted]

1.1.3. Secondary Objective(s) 8

1.1.3.1. Part 1: PF-07248144 Monotherapy (Part 1A) and Combination Dose Escalation (Part 1B[redacted] 8

[redacted]

1.2. Study Design 9

2. ENDPOINTS AND BASELINE VARIABLES: DEFINITIONS AND CONVENTIONS 13

2.1. Primary Endpoint(s) 13

2.1.1. Part 1: PF-07248144 Monotherapy (Part 1A) and Combination Dose Escalation (Parts 1B[redacted] 13

2.1.2. Part 2: PF-07248144 Monotherapy (Part 2A) and Combinations in Dose Expansion (Parts 2B[redacted] 14

2.2. Secondary Endpoint(s) 14

2.2.1. Part 1: PF-07248144 Monotherapy (Part 1A) and Combination Dose Escalation (Parts 1B[redacted] 14

2.2.2. Part 2: PF-07248144 Monotherapy (Part 2A) and Combinations in Dose Expansion (Parts 2B[redacted] 14

2.3. Exploratory Endpoint(s) 15

090177e19dab896e\Approved\Approved On: 07-Jun-2023 12:23 (GMT)

090177e19dab896e\Approved\Approved On: 07-Jun-2023 12:23 (GMT)

2.3.1. Part 1: PF-07248144 Monotherapy (Part 1A) and Combination Dose Escalation (Parts 1B, [redacted])BOR as assessed by investigator based on RECIST v1.1 15

2.3.2. Part 2: PF-07248144 Monotherapy (Part 2A) and Combinations in Dose Expansion (Parts 2B [redacted]) 15

2.3.3. Baseline Variables 16

2.4. Safety Endpoints 16

2.4.1. Adverse Events 16

2.4.2. Laboratory Data 16

3. ANALYSIS SETS (POPULATIONS FOR ANALYSIS) 17

4. GENERAL METHODOLOGY AND CONVENTIONS 18

4.1. Hypotheses and Decision Rules 18

4.1.1. Maximum Tolerated Dose Determination 18

4.2. Sample Size Determination 20

4.2.1. Part 1 Dose Escalation 20

4.2.2. Part 2 Dose Expansion 20

4.3. General Methods 21

4.3.1. Analyses for Binary Endpoints 21

4.3.2. Analyses for Continuous Endpoints 21

4.3.3. Analyses for Categorical Endpoints 21

4.3.4. Analyses for Time-to-Event Endpoints 21

4.4. Methods to Manage Missing Data 22

4.4.1. Missing Dates 22

4.4.2. Efficacy Analysis 22

[redacted]

4.4.6. QTc 23

4.5. Statistical Considerations of COVID-19 Impacted Data 23

5. ANALYSES AND SUMMARIES 24

5.1. Primary Endpoint(s) 24
5.1.1. Dose-Limiting Toxicities (DLTs) 24
5.2. Safety Endpoint(s) 24
5.2.1. Adverse Events 25
5.2.2. Laboratory Test Abnormalities 25
5.2.3. Vital Sign Abnormalities 25
5.2.4. Electrocardiogram Parameters 25
5.3. Secondary Endpoint(s) 27
[redacted]
5.4. Efficacy Endpoint(s) 29
5.5. Exploratory Endpoint(s) 33
5.6. Subset Analyses 33
5.7. Baseline and Other Summaries and Analyses 33
5.7.1. Baseline Summaries 33
5.7.2. Study Conduct and Participant Disposition 33
5.7.3. Study Treatment Exposure 33
5.7.4. Concomitant Medications and Nondrug Treatments 37
5.7.5. Patient Follow-up Summary for Time-to-Event 37
5.7.5.1. Patient follow-up summary for OS 37
5.7.5.2. Patient follow-up summary for PFS 38
5.7.6. Prior Therapy 38
5.8. Data Monitoring Committee or Other Independent Oversight Committee 40

090177e19dab896e\Approved\Approved On: 07-Jun-2023 12:23 (GMT)

6. INTERIM ANALYSES 40
7. REFERENCES 40
8. APPENDICES 41
8.1. Appendix 1: Time to Event Data Analysis Censoring Rules 41
8.2. Appendix 2: List of Abbreviations...42

LIST OF TABLES

Table 1. Summary of Changes...6
Table 2. Progression Free Survival Outcome and Event Dates..............................41
Table 3. Titme to Progression Outcome and Event Dates.....................................41

090177e19dab896e\Approved\Approved On: 07-Jun-2023 12:23 (GMT)

VERSION HISTORY

Table 1. Summary of Changes

Version/ Date	Associated Protocol Amendment	Rationale	Specific Changes
1<br>16 Oct 2020	Amendment 1<br>11 Sep 2020	N/A	N/A
2<br>22 May 2023	Amendement 4<br>14 Nov 2022	To update the Statistical Analysis Plan corresponding to the recent protocol amendment	Introduction of Part 1D and 2D in Sections 2, 3, 5, and 6.<br><br>Updated study schema<br><br>Addition of analyses related to dose exposures, follow-up summary, and prior therapy summary in Section 6.<br>Updated<br><br>Updated analysis set definitions in Section 4.

1. INTRODUCTION

PF-07248144 is an orally available small-molecule inhibitor of human KAT6 histone acetyltransferases, KAT6A and KAT6B that is being investigated in participants with locally advanced or metastatic ER+ HER2- breast cancer, CRPC, or NSCLC who progress on or are intolerant to standard therapy.

This statistical analysis plan (SAP) provides the detailed methodology for summary and statistical analyses of the data collected in Study C4551001. This document may modify the plans outlined in the protocol; however, any major modifications of the primary endpoint definition or its analysis will also be reflected in a protocol amendment.

1.1. Study Objectives

1.1.1. Primary Objective(s)

1.1.1.1. Part 1A: Monotherapy Dose Escalation

- *To assess safety and tolerability of escalating dose levels of PF-07248144 in successive cohorts of participants with locally advanced or metastatic ER+ HER2 breast cancer, CRPC, or NSCLC to determine the monotherapy MTD and to select the monotherapy RDE.*

090177e19dab896e\Approved\Approved On: 07-Jun-2023 12:23 (GMT)

1.1.1.2. Part 1B: Combination Dose Escalation

- *To assess safety and tolerability of PF-07248144 in combination with fulvestrant in participants with locally advanced or metastatic ER+HER2 breast cancer who have progressed after at least 1 prior line of treatment with an endocrine therapy and CDK4/6 inhibitor to determine the combination MTD and to select the combination RDE.*

[redacted]

- [redacted]
- [redacted]

[redacted]

- [redacted]
- [redacted]
- [redacted]
- [redacted]

[redacted]

- [redacted]

090177e19dab896e\Approved\Approved On: 07-Jun-2023 12:23 (GMT)

[redacted]

1.1.3. Secondary Objective(s)

1.1.3.1. Part 1: PF-07248144 Monotherapy (Part 1A) and Combination Dose Escalation (Part 1B, [redacted]

- *To evaluate the single- and multiple-dose PK of PF-07248144 when given as monotherapy (Part 1A), in combination with fulvestrant (Part 1B);* [redacted]
- [redacted]
- [redacted] Part 2: PF-07248144 Monotherapy (Part 2A) and Combinations in Dose Expansion (Parts 2B [redacted])• *To evaluate antitumor activity of PF-07248144 monotherapy and in combination with fulvestrant (Part 2B)* [redacted]
- To evaluate PK of PF-07248144 monotherapy (at RDE from Part 1A) and in combination with fulvestrant (at combination RDE from Part 1B) [redacted]
- [redacted]
- *To evaluate the effect of food on the PK of PF-07248144 administered (at Part 1A RDE) in a subset of participants in Part 2A (approximately 6 participants).*
- *To evaluate urine PK of PF-07248144 in a subset of participants in Part 2A (at least 6 participants).*

[redacted]

- [redacted]
- [redacted]

090177e19dab896e\Approved\Approved On: 07-Jun-2023 12:23 (GMT)

- [redacted]
- [redacted]
- [redacted]

  [redacted]
- [redacted]
- [redacted]
- [redacted]
- [redacted]
- [redacted]

[redacted]

- [redacted]
- [redacted]
- [redacted]

1.2. Study Design

This is an open-label, multi center Phase 1 study in adult participants to evaluate safety, tolerability, PK, and PD of PF 07248144 in locally advanced or metastatic selected solid tumors (ER+HER2- breast cancer, CRPC, or NSCLC) and early signs of clinical activity of

090177e19dab896e\Approved\Approved On: 07-Jun-2023 12:23 (GMT)

PF 07248144 as a single agent and in combination in ER+HER2 breast cancer. The overall study design is depicted in the schema **Study Schema:**

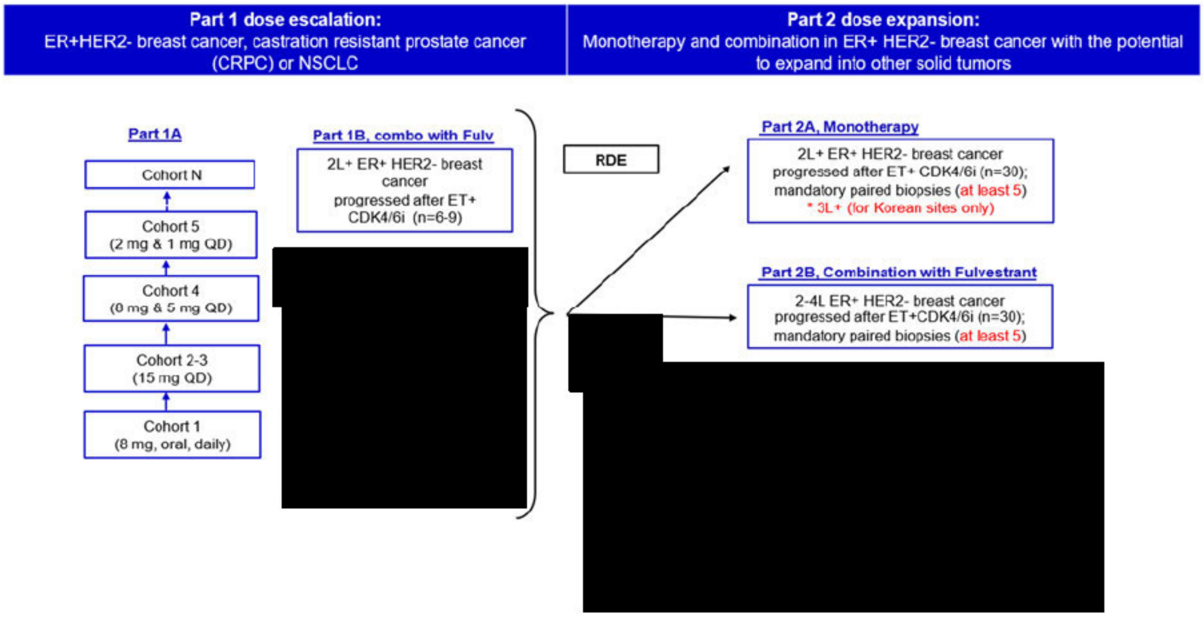

Part 1 *dose escalation consists of: Part 1A, Part 1B,* [redacted].

Part 1A *contains dose escalation as monotherapy in participants with locally advanced or metastatic ER+HER2- breast cancer, CRPC, or NSCLC that are resistant or intolerant to standard therapy or for whom no standard therapy is available, to determine the MTD and select the RDE. Participants will receive escalating doses of PF-07248144. BLRM guided by EWOC principle will be used to guide dose escalation process and determine the MTD. The first dose level in Part 1A will be 8 mg QD. Maximum allowable PF-07248144 dose increment is 100% unless in circumstance defined in protocol. DLT will be assessed during Cycle 1 (the first 28 days with the inclusion of C2D1 laboratory assessments). Each dose level group will be approximately 3 participants, with at least 1 DLT-evaluable participant per cohort in the first 2 cohorts and at least 2 DLT evaluable participants per dose level group in the remaining cohorts for Part 1A. Per BLRM design, expanding additional participants at lower dose levels is allowed to assess safety. Additional dosing frequency such as intermittent dosing may be considered if supported by emerging clinical data.*

In Part 1B*, PF-07248144 in combination with fulvestrant, will be evaluated for dose finding in participants with locally advanced or metastatic ER+HER2- breast cancer (2L+) who have progressed after at least 1 line of treatment with an endocrine therapy and CDK4/6*

090177e19dab896e\Approved\Approved On: 07-Jun-2023 12:23 (GMT)

inhibitor to determine the MTD and RDE for this combination. The definitions of RDE and RP2D are provided in protocol. Combination RDE may be different from monotherapy RDE due to potential toxicity overlap or drug-drug interaction.

[redacted]

Additional dosing frequency such as intermittent dosing may be considered after the second dose group in the study if supported by emerging clinical data. Treatment will continue until PD, unacceptable toxicity, or participant refusal, whichever occurs first.

BLRM specifically developed for double and triple combinations will be used for dose finding in Part 1B, [redacted]*. PF-07248144 may start at 1 dose level below the monotherapy RDE (RDE-1) with fixed doses of fulvestrant* [redacted]*. The definitions of MTD and RDE are provided in protocol. In addition, depending on the safety findings in Part 1A, and whether significant overlapping toxicities are expected in combination, the starting dose of PF-07248144 in combination can be further modified to a lower dose. The sponsor may choose to advance a lower dose to start the combination dose finding Part 1B,* [redacted] *prior to the monotherapy MTD being reached. This decision will be made based on emerging preliminary safety, PK, PD, and/or activity data during Part 1A. At least 2 DLT evaluable participants will be required for dose level groups in Part 1B,* [redacted]

After the determination of the monotherapy expansion RDE in Part 1A, PF-07248144 will be evaluated in a dose expansion cohort as a monotherapy in locally advanced or metastatic ER+HER2- breast cancer (2L+) who have progressed after at least 1 prior line of CDK4/6 inhibitor and 1 line of endocrine therapy (Part 2A). The definitions of RDE and RP2D are provided in protocol. Paired tumor biopsies will be required in at least 5 participants in Part 2A.

A food effect assessment will take place in a subset of participants (approximately 6) in Part 2A. [redacted]

[redacted]

090177e19dab896e\Approved\Approved On: 07-Jun-2023 12:23 (GMT)

After determination of the combination RDE from Part 1B, PF-07248144 in combination with fulvestrant will be evaluated in a dose-expansion combination cohort in participants with advanced or metastatic 2-4L ER+HER2- breast cancer whose disease has progressed after at least 1 prior line of a CDK4/6 inhibitor and at least 1 prior line of endocrine therapy and who must not have received more than 3 lines of systemic therapies in advanced or metastatic setting (Part 2B). Paired tumor biopsies will be required from at least 5 participants in Part 2B.

[redacted]

Furthermore, to better characterize safety, PK and potential efficacy, each dose expansion may investigate more than one dose level(s) of the study intervention(s) in up to an additional 30 participants. The dose levels to be evaluated will be informed by available data from dose escalation (including safety, PK/PD, and activity) and will not exceed the monotherapy MTD unless the observed exposure in combination significantly lower than monotherapy.

The evaluation of an alternative dosing regimen (eg, different daily frequency or intermittent dosing) may be considered during dose escalation or after determination of the MTD and/or RDE based on emerging and available preliminary clinical data, including safety/tolerability, laboratory, PK, and PD findings. If an alternative dosing regimen (eg, intermittent dosing) occurs, it will be initiated with a dose level at a comparable total daily dose determined from the MTD and/or RDE of the QD/BID regimen and satisfying EWOC criteria. PF-07248144 [redacted] *intermittent dosing schedules may also be evaluated, if indicated based on emerging clinical data.*

[redacted]

Number of Participants

The total number of participants is estimated to be approximately 140 to 200

Part 1 Dose Escalation: *Approximately 70 participants will be enrolled in Part 1 including 25 to 30 participants in Part 1A, 6 to 9 participants in Part 1B,* [redacted]

The actual number of participants enrolled will depend on the tolerability of PF-07248144 and the number of dose levels required to identify the MTD/RDE as monotherapy or in combination.

090177e19dab896e\Approved\Approved On: 07-Jun-2023 12:23 (GMT)

Part 2: Dose Expansion: *Approximately 90-120 participants are expected to be enrolled in Part 2. Approximately 30 participants each will be enrolled in Parts 2A, 2B* [redacted]*, with up to an additional approximately 30 patients at an alternative dose(s) as discussed above.*

All participants will undergo up to 28 days of screening prior to study entry. Eligible participants will then receive study intervention for up to 2 years, or until disease progression, unacceptable toxicities, a decision by the participant (withdrawal of consent or no longer willing to participate) or investigator to discontinue treatment, or study termination. Any additional treatment beyond 2 years shall be discussed and approved by the sponsor. After EOT, all participants will complete a 28 day post-treatment follow up visit for AEs. Participants in Part 2 dose expansion cohorts will be contacted by telephone approximately every 3 months for survival data collection until end of trial (2 years from last participant first dose), unless otherwise notified by the sponsor.

A participant is considered to have completed the study if he/she has completed all phases of the study including the last scheduled procedure shown in the schedule of activities, including overall survival (OS). OS follow-up should not continue after end of the study (protocol Section 4.4).

During treatment with study intervention, all cycles will be 28 days in length. Every effort should be made to administer study intervention on the planned dose and schedule. In the event of significant toxicities, dosing may be interrupted, modified, or discontinued.

[redacted]

2. ENDPOINTS AND BASELINE VARIABLES: DEFINITIONS AND CONVENTIONS

2.1. Primary Endpoint(s)

2.1.1. Part 1: PF-07248144 Monotherapy (Part 1A) and Combination Dose Escalation (Parts 1B [redacted]

- *DLTs.*
- *AEs as characterized by type, frequency, severity (as graded by NCI CTCAE version 5.0), timing, seriousness, and relationship to study therapy.*
- *Laboratory abnormalities as characterized by type, frequency, severity (as graded by NCI CTCAE version 5.0), and timing*

[redacted]

[redacted]

090177e19dab896e\Approved\Approved On: 07-Jun-2023 12:23 (GMT)

2.1.2. Part 2: PF-07248144 Monotherapy (Part 2A) and Combinations in Dose Expansion (Parts 2B [redacted]

- *AEs as characterized by type, frequency, severity (as graded by NCI CTCAE version 5.0), timing, seriousness, and relationship to study therapy.*
- *Laboratory abnormalities as characterized by type, frequency, severity (as graded by NCI CTCAE version 5.0), and timing.*

2.2. Secondary Endpoint(s)

2.2.1. Part 1: PF-07248144 Monotherapy (Part 1A) and Combination Dose Escalation (Parts 1B [redacted]

PK parameters of PF-07248144:

- [redacted]
- [redacted]
- [redacted]
- [redacted]
- [redacted]

2.2.2. Part 2: PF-07248144 Monotherapy (Part 2A) and Combinations in Dose Expansion (Parts 2B [redacted]

- *BOR, DOR, and CBR as assessed by investigator based on RECIST v1.1.*
- *PFS as assessed by investigator based on RECIST v1.1.*
- *TTP as assessed by investigator based on RECIST v1.1.*
- *Overall Survival.*
- *Monotherapy and combination cohorts: Trough concentrations of PF-07248144 for selected cycles.*
- [redacted]

090177e19dab896e\Approved\Approved On: 07-Jun-2023 12:23 (GMT)

- [redacted]

[redacted]

2.3. Exploratory Endpoint(s)

2.3.1. Part 1: PF-07248144 Monotherapy (Part 1A) and Combination Dose Escalation (Parts 1B[redacted]BOR as assessed by investigator based on RECIST v1.1.

- *DOR and CBR as assessed by investigator based on RECIST v1.1.*
- *PFS as assessed by investigator based on RECIST v1.1.Part 1A CRPC Participants Only:*

[redacted]

- *Genomic, transcriptomic, and/or protein analyses of baseline and/or on-treatment tumor tissues and their relationship to clinical response.*
- *Changes in peripheral blood biomarkers of cfDNA mutations, cytokine, and chemokine soluble proteins that may be related to response or resistance to treatment.*

2.3.2. Part 2: PF-07248144 Monotherapy (Part 2A) and Combinations in Dose Expansion (Parts 2B[redacted]

- *Changes from baseline levels of H3K23Ac, [redacted] in pre- and on treatment tumor biopsies, and/or peripheral blood.*
- [redacted]

090177e19dab896e\Approved\Approved On: 07-Jun-2023 12:23 (GMT)

- *Genomic, transcriptomic, and protein analyses of baseline and/or on-treatment tumor tissues and their relationship to clinical response.*
- *Changes in peripheral blood biomarkers of cfDNA mutations,* [redacted] *that may be related to response or resistance to treatment.*
- [redacted]

2.3.3. Baseline Variables

Baseline characteristics will be collected according to Schedule of Activities as specified in the protocol. No baseline variable will be used for stratification or as covariates for the primary statistical analysis. Unless otherwise specified, the baseline value is defined as the value collected at the time closest to, but prior to, starting the study intervention administration in the first cycle.

2.4. Safety Endpoints

For safety summaries, unless other specified, safety data are summarized for the data collected during "on-treatment period", which is defined as the period that starts with the first dose date and ends at min(last dose date + 28 days, start of new anti-cancer therapy – 1 day). Anti-cancer radiation or surgery is not considered as a start of new anti-cancer therapy.

2.4.1. Adverse Events

The definitions of an AE and an SAE can be found in Appendix 3 (Section 10.3) of the protocol.

AEs will be reported by the participant (or, when appropriate, by a caregiver, surrogate, or the participant's legally authorized representative).

The investigator and any qualified designees are responsible for detecting, documenting, and recording events that meet the definition of an AE or SAE and remain responsible to pursue and obtain adequate information both to determine the outcome and to assess whether the event meets the criteria for classification as an SAE or caused the participant to discontinue the study intervention.

Each participant will be questioned about the occurrence of AEs in a nonleading manner. In addition, the investigator may be requested by Pfizer Safety to obtain specific follow up information in an expedited fashion.

2.4.2. Laboratory Data

Further details of the laboratory tests can be found in Appendix 10.2 of the protocol.

090177e19dab896e\Approved\Approved On: 07-Jun-2023 12:23 (GMT)

3. ANALYSIS SETS (POPULATIONS FOR ANALYSIS)

Data for all participants will be assessed to determine if participants meet the criteria for inclusion in each analysis population.

Population	Description
Full analysis set	The full analysis set includes all enrolled participants who will receive C1D1 dose. Unless otherwise specified the full analysis set will be the default analysis set used for all efficacy analyses.
Safety analysis set.	The safety analysis set includes all enrolled participants who receive at least 1 dose of study intervention (including C1D-7). Unless otherwise specified the safety analysis set will be the default analysis set used for all safety analyses.
Per protocol analysis set (evaluable for MTD)	*The per protocol analysis set includes all enrolled participants who had at least 1dose of study treatment and either experienced DLT or do not have major treatment deviations during the DLT observation period.*
mITT Population	*The mITT is the analysis population that will follow the ITT principle and include participants receiving at least 1 dose of study medication with baseline assessment and at least 1 post baseline assessment, discontinuing due to disease progression, death or any other reason before the first tumor assessment. The mITT population may be used for interim analysis and conference presentations when the study is still ongoing.*
PK analysis sets	*The PK parameter analysis population is defined as all enrolled participants treated who do not have protocol deviations influencing PK assessment, and have sufficient information to estimate at least 1 of the PK parameters of interest.*<br>*The PK concentration population is defined as all enrolled participants who are treated and have at least 1 analyte concentration.*
Response Evaluable Set	*The response evaluable population will include all participants who received at least 1 dose of study treatment and had baseline disease assessment and at least 1 post baseline disease assessment.*
PD/Biomarker analysis set(s)	*The PD/Biomarker analysis population is defined as all enrolled participants with at least 1 of the PD/Biomarkers evaluated at pre and/or post dose.*

090177e19dab896e\Approved\Approved On: 07-Jun-2023 12:23 (GMT)

4. GENERAL METHODOLOGY AND CONVENTIONS

4.1. Hypotheses and Decision Rules

There will be no formal hypothesis testing in this study.

4.1.1. Maximum Tolerated Dose Determination

Determination of MTD will be performed using a Per-protocol analysis set (evaluable for MTD).

<u>Bayesian adaptive approach:</u>

The dose escalation in Part 1A, and dose finding in Part 1B [redacted] *of the study will be guided by a Bayesian analysis of Cycle 1 DLT data for PF 07248144 as a monotherapy (Part 1A) or in combination (Part 1B,* [redacted]*). A traditional 2-parameter BLRM will be used to model the dose/DLT relationship of PF 07248144 monotherapy. A more complex BLRM model specifically designed for combinations will be used to model the dose/DLT relationship of PF-07248144 given in combination with fulvestrant,* [redacted]

Using DLT data at all tested dose levels and pre specified prior distribution of model parameters, the posterior distribution for probability of having a DLT will be calculated for all dose levels.The dose escalation in Part 1A, Part 1B [redacted] *of the study will be guided by a Bayesian analysis of Cycle 1 DLT data for PF-07248144 as a monotherapy (Part 1A) or in combination (Part 1B* [redacted]*). A traditional 2-parameter BLRM* (Neuenschwander, et al. 2015)[1] *will be used to model the DLT relationship of PF-07248144* monotherapy *and a more complex BLRM model specifically designed for double* [redacted] *combinations will be used to model the dose toxicity relationship of PF-07248144 given in combination with fulvestrant* [redacted] *Using DLT data at all tested dose levels and pre specified prior distribution of model parameters, the posterior distribution for probability of having a DLT will be calculated for all dose levels.*

<u>Assessment of participant risk:</u>

After each cohort of participants, the posterior distribution for the risk of DLT for new participants at different doses of interest for PF-07248144 will be evaluated. The posterior distributions will be summarized to provide the posterior probability that the risk of DLT lies within the following intervals:

Under-dosing:	[0, 0.16]
Targeted dosing:	[0.16, 0.33]
Overdosing:	[0.33, 1]

090177e19dab896e\Approved\Approved On: 07-Jun-2023 12:23 (GMT)

<u>The EWOC principle:</u>

Dosing decisions are guided by the escalation with overdose control principal. A dose may only be used for newly enrolled participants if the risk of excessive toxicity at that dose [redacted].

<u>Prior distributions:</u>

Weakly informative prior distributions based on pre-clinical/expert opinion information will be chosen for the logistic parameters for prior distribution in Part 1A, see Appendix 9 of the protocol.

A MAP approach might be used to derive the prior distribution for model parameters used in Part 1B, [redacted] *based on the data collected in Part 1A and DLT data collected in clinical studies for fulvestrant,* [redacted]*. The MAP prior for the logistic model parameters for this study is the predictive posterior distribution of the parameters given the historical data. MAP priors are derived from hierarchical models, which take into account possible differences between the studies. A full description of the application of the MAP approach to derive the prior distributions of the model parameters is given in a Technical Supplement.*

In case of change of the dosing regimen, DLT data accumulated during the dose escalation with the original regimen may be used to form a prior for further BLRM analysis. Details about derivation of this prior using MAP approach is given in a Technical Supplement.

<u>Starting dose:</u>

The starting dose of Part 1A is 8 mg QD. For this dose the prior risk of overdosing is 10.1%, which satisfies the EWOC criterion. A full assessment of the prior risk to participants is given in Appendix 9 of the protocol.

[redacted]

<u>Sensitivity Analysis:</u>

To mitigate the risk of dichotomizing and misclassifying DLTs, a sensitivity analysis that uses weighted DLT/AE data (in equivocal cases) into the BLRM model estimation will also be performed. If all the investigators and the sponsor agree on the equivocal DLT/AE data, the DLT weighting approach could be the primary dose escalation method. See Appendix 9 of the proptocol for more details.

<u>Stopping Criteria:</u>

The maximum number of participants in dose escalation part of the trial is set to 40. The trial will be stopped when the following criteria are met:

090177e19dab896e\Approved\Approved On: 07-Jun-2023 12:23 (GMT)

- *At least 6 participants have been treated at the recommended MTD/RP2D.*
- *The dose $\tilde{d}$ satisfies one of the following conditions:*
  - *The probability of target toxicity at dose $\tilde{d}$ exceeds 50%, ie, $Pr(0.16 \leq \pi_{\tilde{d}} < 0.33) \geq 50\%$.*
  - *A minimum of 12 participants have been treated in the trial.*

These stopping criteria are applicable to Part 1A, Part 1B, [redacted]

4.2. Sample Size Determination

The total number of participants is estimated to be approximately 145 to 190.

4.2.1. Part 1 Dose Escalation

Approximately 70 participants will be enrolled in Part 1 including 25 to 30 participants in Part 1A, 6 to 9 participants in Part 1B, [redacted]*. The actual number of participants enrolled will depend on the tolerability of PF-07248144 and the number of dose levels required to identify the MTD/RDE as monotherapy or in combination.*

4.2.2. Part 2 Dose Expansion

Part 2A: *Approximately 30 participants will be enrolled in Part 2A.*

Assuming a non informative prior (ie, Jeffrey's prior) if 9 out of 30 participants have tumor response, this would predict a posterior probability (Beta Binomial) equal to [redacted] *that the true response is not inferior to target response rate of* [redacted] *and a posterior probability equal to* [redacted] *that the true response is inferior to benchmark rate of* [redacted].

Part 2B: *Approximately 30 participants will be enrolled in Part 2B.*

Assuming a non informative prior (ie, Jeffrey's prior) if 9 out of 30 participants have tumor response, this would predict a posterior probability (Beta Binomial) equal to [redacted] *that the true response is not inferior to target response rate of* [redacted] *and a posterior probability equal to* [redacted] *that the true response is inferior to benchmark rate of* [redacted].

Participants from Part 1A and Part 1B [redacted] *who were treated at the dose level selected for Part 2 and fulfilling Part 2 inclusion/exclusion criteria may be counted towards the sample size of Part 2 at the corresponding regimen (monotherapy and combination).*

[redacted]

[redacted]

090177e19dab896e\Approved\Approved On: 07-Jun-2023 12:23 (GMT)

[redacted]

Participants from Part 1A, Part 1B, [redacted] *who were treated at the dose level selected for Part 2 and fulfilling Part 2 inclusion/exclusion criteria may be counted towards the sample size of Part 2 at the corresponding regimen (monotherapy and combination).*

4.3. General Methods

The data will be summarized by dose level, defined by the initial dose of the study intervention administered to participants. If a dose level has more than 1 cohort, data from these cohorts will be combined. DLT rates at the study dose levels will be presented via mean and medians and a Bayesian credible interval based on the posterior density from the full probability model. This information will also be used for the dose level review meetings (DLRM) to guide the dose escalation.

4.3.1. Analyses for Binary Endpoints

Binary data will be summarized using number of unique participant incidence, proportion in the analysis set, and the 2-sided 95% exact confidence interval for the proportions. The confidence interval will be based on the Clopper-Pearson exact method.

4.3.2. Analyses for Continuous Endpoints

Continuous data will be summarized with the mean, median, minimum, maximum, standard deviation, and 2-sided 95% confidence interval of the mean if the sample size permits and deemed necessary.

4.3.3. Analyses for Categorical Endpoints

Categorical data will be summarized by number of unique participant incidence and proportion of participant in each category, and 2-sided 95% confidence interval of the proportion if the sample size permits and deemed necessary.

4.3.4. Analyses for Time-to-Event Endpoints

The time-to-event endpoints will be summarized using the Kaplan-Meier method and estimated survival curves may be displayed graphically when needed. Graphs will describe the number of participants at risk over time. The median, quartiles, and probabilities of an event at particular points in time will be estimated by the Kaplan-Meier method, when possible based on the number of observed events. Confidence intervals for medians and quartiles, based on the Brookmeyer-Crowley method (Brookmeyer and Crowley 1982),[2] may be presented. Confidence intervals for the estimated probability of an event at a particular time point may be generated using the Greenwood formula.

090177e19dab896e\Approved\Approved On: 07-Jun-2023 12:23 (GMT)

4.4. Methods to Manage Missing Data

For the analysis of safety endpoints, the sponsor data standard rules for imputation will be applied based on Pfizer Safety Rulebook.

4.4.1. Missing Dates

In compliance with Pfizer standards, if the day of the month is missing for any date used in a calculation, the 1st of the month will be used to replace the missing date unless the calculation results in a negative time duration (eg, date of onset cannot be prior to day one date). In this case, the date resulting in 0 time duration will be used. Pfizer standards are also used if both month and day are missing (Jan 1 unless negative time duration). This excludes the pharmacokinetic and ECG analyses, which will only use the actual date collected or if date not available deem the data missing.

4.4.2. Efficacy Analysis

For binary efficacy endpoint related to tumor assessment , every effort will be made to retrieve data in the CRF, however missing data will be left as is, no imputation will be performed. The reasons for missing tumor assessment will be collected.

For the time-to-event endpoints, the missing data handling method will be censoring. Censoring rules for time-to-event endpoints are detailed in Section 8.1.

[redacted]

[redacted]

[redacted]

[redacted]

[redacted]

[redacted]

- [redacted]
- [redacted]

[redacted]

[redacted]

090177e19dab896e\Approved\Approved On: 07-Jun-2023 12:23 (GMT)

090177e19dab896e\Approved\Approved On: 07-Jun-2023 12:23 (GMT)

[redacted]

[redacted]

[redacted]

[redacted]

[redacted]

4.4.6. QTc

For the corrected QT (QTc) analyses, no values will be imputed for missing data.

4.5. Statistical Considerations of COVID-19 Impacted Data

In March 2020, the World Health Organization (WHO) announced a global pandemic of the virus SARS-CoV-2 and the resulting disease COVID-19. During the conduct of this trial, if any participant's data is impacted by this pandemic, the following considerations will be given in the data analyses:

a. If a participant dropped out of the study during the DLT evaluation window in Parts 1A, 1B, [redacted] due to COVID-19 and does not meet the DLT evaluation criteria, a replacement participant may be added.

b. Death caused by COVID-19 is still considered as an "event" in the analysis of PFS and OS. If deemed necessary, a sensitivity analysis may be performed where COVID-19 driven death is censored at the death date.

c. If a scheduled tumor radiographic scan is *delayed* out of the Schedule of Activity allowable window, or is *missing* (ie, participant skipped a scheduled tumor radiographic scan) due to any reasons related to the pandemic, this delay or missingness does not alter the censoring rules for PFS or TTP. A censoring reason of "COVID-19" may be added to the PFS or TTP summary if the specific reason of

tumor scan delay or missing can be attributed to COVID-19. If deemed necessary, a sensitivity analysis may be performed where participants would be censored on the date of COVID-19 diagnosis.

d. Any COVID-19 related symptoms are to be captured as adverse events in the case report form. Those adverse events will be summarized in the same manner as other adverse events. If a label or phrase of COVID-19 can be identified in the investigator provided adverse event term, then a separate AE listing may be provided for just the COVID-19 related events.

e. If identifiable, the COVID-19 related data points, including missing data where the reason of missing is identified as COVID-19 related (site closure hence data could not captured; participants skipped a visit because of concern over the pandemic), protocol deviations driven by COVID-19, safety events caused by COVID-19 may be separately listed.

5. ANALYSES AND SUMMARIES

5.1. Primary Endpoint(s)

5.1.1. Dose-Limiting Toxicities (DLTs)

The DLT events will be summarized by dose level. A listing of the DLTs events will also be provided in which the participant primary diagnosis (malignancy), dose level the participant was enrolled to, DLT event start day and stop day relative to the cycle 1 day 1 dose date, the DLT event term, NCI CTCAE grade, relatedness to the investigational product (PF-07248144), or Fulvestrant (Part 1B), [redacted] outcome of the event, along with other variables deemed important, will be included.

5.2. Safety Endpoint(s)

All safety analyses will be performed on the safety population.

Summaries and analyses of safety parameters will include all participants in the safety analysis set.

AEs, ECGs, BP, pulse rate, continuous cardiac monitoring, and safety laboratory data will be reviewed and summarized on an ongoing basis during the study to evaluate the safety of participants. Any clinical laboratory, ECG, BP, and PR abnormalities of potential clinical concern will be described. Safety data will be presented in tabular and/or graphical format and summarized descriptively, where appropriate.

Medical history and physical examination and neurological examination information, as applicable, collected during the course of the study will be considered source data and will not be required to be reported, unless otherwise noted. However, any untoward findings identified on physical and/or neurological examinations conducted during the active collection period will be captured as AEs, if those findings meet the definition of an AE.

090177e19dab896e\Approved\Approved On: 07-Jun-2023 12:23 (GMT)

Data collected at screening that are used for inclusion/exclusion criteria, such as laboratory data, ECGs, and vital signs, will be considered source data, and will not be required to be reported, unless otherwise noted. Demographic data collected at screening will be reported.

5.2.1. Adverse Events

AEs will graded by the investigator according to the CTCAE version 5.0 and coded using MedDRA. AE data will be reported in tables and listings. Summaries of adverse event by mapped terms, appropriate thesaurus level, toxicity grade, and seriousness and relationship to study treatment will be presented, as well as summaries of adverse events leading to death and premature withdrawal from study treatment. The number and percentage of participants who experienced any AE, SAE, treatment related AE, and treatment related SAE will be summarized according to worst toxicity grades. The summaries will present AEs on the entire study period. Listings of DLTs and deaths will be provided.

5.2.2. Laboratory Test Abnormalities

The number and percentage of participants who experienced laboratory test abnormalities will be summarized according to worst toxicity grade observed for each laboratory assay. The analyses will summarize laboratory tests on the entire study. For laboratory tests without CTCAE grade definitions, results will be categorized as high, normal, or low. .

5.2.3. Vital Sign Abnormalities

Vital signs including temperature, pulse rate, respiratory rate, and blood pressure, will be assessed.

The vital signs will be generally considered as continuous endpoints. However the summaries of vital signs as continuous variables will not be provided as they may not be clinically meaningful. Instead, vital signs during the on-treatment period will be summazried by the categories of abnormality as specified in Appendix 1. Shift tables will not be provided unless deemed necessary.

5.2.4. Electrocardiogram Parameters

Changes from baseline for the following ECG parameters: QT interval, heart rate, QTc interval, PR interval, and QRS complex will be summarized by treatment and time.

The number (%) of participants with maximum postdose QTc values and maximum increases from baseline in the following categories will be tabulated by treatment:

Safety QTc Assessment

Degree of Prolongation	Mild (msec)	Moderate (msec)	Severe (msec)
Absolute value	>450-480	>480-500	>500
Increase from baseline		30-60	>60

In addition, the number of participants with uncorrected QT values >500 msec will be summarized.

[redacted]

[redacted]

090177e19dab896e\Approved\Approved On: 07-Jun-2023 12:23 (GMT)

If more than 1 ECG is collected at a nominal time after dose administration (for example, triplicate ECGs), the mean of the replicate measurements will be used to represent a single observation at that time point. If any of the 3 individual ECG tracings has a QTc value >500 msec, but the mean of the triplicates is not >500 msec, the data from the participant's individual tracing will be described in a safety section of the CSR in order to place the >500-msec value in appropriate clinical context. However, values from individual tracings within triplicate measurements that are >500 msec will not be included in the categorical analysis unless the average from the triplicate measurements is also >500 msec. Changes from baseline will be defined as the change between the postdose QTc value and the average of the time-matched baseline triplicate values on Day -1, or the average of the predose triplicate values on Day 1.

In addition, an attempt will be made to explore and characterize the relationship between plasma concentration and QT interval length using a PK/PD modeling approach. If a PK/PD relationship is found, the impact of participant factors (covariates) on the relationship will be examined.

The analysis of ECG results will be based on participants in the safety analysis set with baseline and on-treatment ECG data. Baseline is defined as Cycle 1 Day 1 predose.

ECG measurements (an average of the triplicate measurements) will be used for the statistical analysis and all data presentations. Any data obtained from ECGs repeated for safety reasons after the nominal time-points will not be averaged along with the preceding triplicates. Interval measurements from repeated ECGs will be included in the outlier analysis (categorical analysis) as individual values obtained at unscheduled time points.

QT intervals will be corrected for HR (QTc) using standard correction factors (ie, Fridericia's [default correction], Bazett's, and possibly a study-specific factor, as appropriate). Data will be summarized and listed for QT, HR, RR, PR, QRS, QTcF (and other correction factors, eg, QTcB as appropriate), and by dose level in Part 1A and by group in Part 2 (Part 2A and Part 2B). Individual QT (all evaluated corrections) intervals will be listed by dose and time in Part 1A and by cohort and time in Part 2. The most appropriate correction factor will be selected and used for the following analyses of central tendency and outliers and used for the study conclusions. Descriptive statistics (n, mean, median, standard deviation, minimum, and maximum) will be used to summarize the absolute corrected QT interval and changes from baseline in corrected QT after treatment by study cohort, dose and time point.

090177e19dab896e\Approved\Approved On: 07-Jun-2023 12:23 (GMT)

5.3. Secondary Endpoint(s)

5.3.1. Pharmacokinetic Parameters

5.3.1.1. Single Dose and Steady-State PF-07248144 Pharmacokinetic Analysis (With the Exception of Food Effect Assessment) [redacted]

5.3.1.2.

Following single dose administration, plasma PK parameters [redacted] *ill be estimated.*

[redacted]

[redacted]

[redacted]

[redacted]

[redacted]

[redacted]

[redacted]

[redacted]

090177e19dab896e\Approved\Approved On: 07-Jun-2023 12:23 (GMT)

[redacted]

[redacted]

[redacted]

[redacted]

[redacted]

[redacted]

[redacted]

5.3.1.3. Effect of Food on PF-07248144 Pharmacokinetics (Part 2A)

[redacted]

[redacted]

[redacted]

5.3.1.5. Pharmacodynamic and Biomarker Analyses

Results from PD and biomarker exploratory analyses will be reported in the CSR where possible. However, given the exploratory nature of exploratory objectives and endpoints, the

090177e19dab896e\Approved\Approved On: 07-Jun-2023 12:23 (GMT)

analyses may not be complete at the time of the CSR. Results from exploratory analyses that are not included in the CSR will be shared with the scientific community through publication at a scientific conference and/or in a peer-reviewed scientific journal.

5.3.2. Population Pharmacokinetic Analysis [redacted]

Results from PD and biomarker exploratory analyses will be reported in the CSR where possible. However, given the exploratory nature of exploratory objectives and endpoints, the analyses may not be complete at the time of the CSR. Results from exploratory analyses that are not included in the CSR will be shared with the scientific community through publication at a scientific conference and/or in a peer-reviewed scientific journal.

5.4. Efficacy Endpoint(s)

BOR, DOR, CBR, and PFS as assessed by investigator based on RECIST v1.1, are exploratory endpoints in Part1 to evaluate the anti-tumor activity of PF-07248144. They, along with TTP, as assessed by investigator based on RECIST version 1.1, and Overall Survival are secondary endpoints in Part 2.

Full Analysis Set will be used for all response related analyses including BOR, PFS, TTP, and OS. DOR will be analyzed only on responders.

Tumor response will be presented in the form of participant data listings that include, but are not limited to tumor type, dose on Day 1, tumor response at each visit, and best overall response. Proportion of participants responding to the treatment will be presented for each dose level.

For prostate cancer only, PSA and bone scan monitor, report and analysis should follow PCWG3 guidelines (see Protocol) Existing or new bone lesions as assessed by radionuclide bone scan will be described and documented.

PSA data will be presented in form of listings that include dose on Day 1, measurement, and % change from baseline at visit.

Part 1A, Part 1B, [redacted] *Progression date, date of first response, last tumor assessment date, and date of last contact will be listed.*

Part 2: The Kaplan-Meier method will be used to analyze all time to event endpoints. Median PFS (if reached) will be calculated. DOR will be listed. Efficacy data of participants from Part 1A, Part 1B, [redacted] *treated at the dose level selected for the corresponding expansion cohort and satisfying inclusion/exclusion criteria might be included in the analysis of efficacy endpoints of Part 2.*

Part 1 and Part 2 data will be summarized separately, and may also be pooled together for analysis if deemed necessary, eg, to account for food effects subgroups. Specifically, the key tables in Part 2 include:

090177e19dab896e\Approved\Approved On: 07-Jun-2023 12:23 (GMT)

1. BOR is the best response recorded from the randomization until disease progression or death due to any cause. This is derived from the sequence of objective statuses. Objective statuses are not considered after objective progression is documented or after start of the first anticancer treatment post discontinuation of protocol treatment. BOR for each patient will be derived as one of the following categories.

   - Complete Response (CR): Two objective statuses of CR a minimum of 4 weeks apart documented before progression.

   - Partial Response (PR) (applicable only to patients with measurable disease at baseline): Two objective statuses of PR or better (PR followed by PR or PR followed by CR) a minimum of 4weeks apart documented before progression, but not qualifying as CR. Sequences of PR- Stable- PR are considered PRs as long as the 2 PR responses are observed at a minimum of 4 weeks apart.

   - Stable Disease (SD) (applicable only to patients with measurable disease at baseline): At least 1 objective status of stable disease or better documented at least 6 weeks after ‘start date’ and before progression but not qualifying as CR or PR. In this document, ‘start date’ for a patient is the date of first treatment (if study treatment start date is not available, date of enrollment is used) for non-randomized trials and the date of randomization for randomized trials.

   - Progressive Diseas (PD): Progression documented within 17 weeks after ‘start date’ and not qualifying as CR, PR or SD.

   - Non-CR/non-PD (applicable only to patients with non-measurable disease at baseline): At least 1 non-CR/non-PD assessment (or better) documented at least 6 weeks after ‘start date’ and before first documentation of progression (and not qualifying for CR or PR).

   -

   - Not Evaluable (NE): All other cases. Note that reasons for NE should be summarized and the following reasons could be used:

     - No adequate baseline assessment

     - No evidence of disease at baseline

     - No post-baseline assessments due to early death (ie, death prior to 6 weeks after ‘start date’)

     - No post-baseline assessments due to other reasons

     - All post-baseline assessments have overall response NE

090177e19dab896e\Approved\Approved On: 07-Jun-2023 12:23 (GMT)

    - New anticancer therapy started before first post-baseline assessment
    - SD of insufficient duration (< 6 weeks after 'start date' without further evaluable tumor assessments)
    - PD too late (> 17 weeks after 'start date')

- Special and rare cases where BOR is NE due to both SD of insufficient duration ('too early') and late PD will be classified as 'SD of insufficient duration'.
- An objective status of PR, SD, or Non-CR/Non-PD cannot follow one of CR. SD can follow PR only in the rare case that tumor increases by less than 20% from the nadir, but enough that a previously documented 30% decrease from baseline no longer holds. If this occurs, the sequence PR-SD-PR is considered a confirmed PR. A sequence of PR – SD – SD – PD would be a best response of SD if the window for SD definition has been met.
- Unconfirmed CR (uCR) is defined as one objective status of CR documented before PD, while confirmed CR requires two objective statuses of CR a minimum of four weeks apart documented before PD. Sequences of CR - Non-evaluable - CR are considered confirmed CR as long as the two CR responses are observed at a minimum of 4 weeks apart. Similarly, unconfirmed PR (uPR) is defined as one objective status of PR documented before PD but not qualifying as uCR. Confirmed PR is defined as two objective statuses of PR or better (PR followed by PR or PR followed by CR) a minimum of four weeks apart documented before PD, but not qualifying as CR. Sequences of PR - Stable Disease or Non-evaluable - PR are considered PRs as long as the two PR responses are observed at a minimum of 4 weeks apart. Based on these definitions, the unconfirmed ORR analysis will include both confirmed CR or PR and unconfirmed CR or PR as responders, whereas the confirmed ORR analysis will only include confirmed CR or PR as responders.

Descriptive statistics (frequency and percentage) and 95% confidence interval will be provided.

2. DOR is defined as the time from first documentation of CR or PR to date of first documentation of PD or death due to any cause. The responders who have not disease progressed at the time of analysis will be censored at the last available tumor scan date. Both confirmed DOR and unconfirmed DOR (uDOR) will be determined separately for the subset of participants with a confirmed and unconfirmed objective response of CR or PR.

3. *CBR is defined as the proportion of patients with a best overall response (BOR) of CR, PR or SD lasting for 24 weeks.*

090177e19dab896e\Approved\Approved On: 07-Jun-2023 12:23 (GMT)

4. *PFS is defined as the time from start date to date of first documentation of progression as per RECIST 1.1, or death due to any cause. Progression is defined as the appearance of local, regional or distant disease of the same type after complete response or progression of pre-existing lesions. It does not include second primary malignancies of unrelated types*.

   PFS will be summarized using the Kaplan-Meier method, as descrbied in Section 4.3.4. and may also be displayed graphically when appropriate.

5. TTP is the time from start date to the date of the first documentation of PD per RECIST 1.1. Start date is the start of treatment.

   TTP will be summarized using the Kaplan-Meier method, as descrbied in Section 4.3.4. TTP may also be displayed graphically when appropriate.

6. *OS is defined as the time from start date to date of death due to any cause*. Start date is the start of treatment.

   OS will be summarized using the Kaplan-Meier method, as descrbied in Section 4.3.4. OS may also be displayed graphically when appropriate. Participants last known to be alive are censored at date of last contact. The date of last contact will be derived for participants not known to have died at the analysis data cutoff date using the latest complete date (non-imputed) among AE collection date, vital sign date, tumor assessment date, date of follow up anti-cancer therpay etc. This is not an exclusive list of possible dates. Any retrievable last contact date from the clinical database will be used.

   Tumor response data in Part 1, Part 2 and across the two Parts may be summarized with descriptive statistics (frequency and percentage) in the following groups by visit and then best overall response across all visits:

- Overall summary for all doses and all tumor types;
- By tumor type regardless of dose;
- By dose regardless of tumor type;
- By dose and tumor type if data permit.

Summary tables of BOR, DOR, PFS, TTP, and OS may be provided by the groups aforementioned when deemed necessary (eg, if there are ≥5 patients in a specific group).

Efficacy listings (tumor measurements listings and tumor response listings) will be provided that include the investigator provided tumor measurement data, tumor response, BOR, DOR, first CR/PR date, last date with CR or PR, most recent date without progression, progression date, death date, and last tumor assessment date, etc.

090177e19dab896e\Approved\Approved On: 07-Jun-2023 12:23 (GMT)

Swimmer plot for individual clinical response and time on treatment, waterfall plot for individual tumor size percent change from baseline, and spider plot for individual tumor size percent change from baseline over time will be presented.

5.5. Exploratory Endpoint(s)

[redacted]

5.6. Subset Analyses

In general, Parts 1A, 1B[redacted] and Part 2A, 2B[redacted]data will be summarized and analyzed separately, and may also be pooled together for analysis if deemed necessary. Other specific subset analyses may be determined during the trial if necessary.

5.7. Baseline and Other Summaries and Analyses

5.7.1. Baseline Summaries

Baseline characteristics will be summarized and/or listed in participant level data listings:

- Demographics: will be summarized by dose level (Part 1) and overall across all dose levels, or by arm (Part 2). This will be based on the Full analysis set. Demographic data will also be listed in a data listing.
- Primary diagnosis: will be listed for all enrolled participants.
- Baseline signs and symptoms: will be summarized by dose level (Part 1) or by arm (Part 2) using the full analysis set. This data will also be listed for all enrolled participants.
- ECOG performance status: will be summarized by dose level (Part 1) or by arm (Part 2) using the full analysis set.

Prior medication, medical history, physical examinations will be tabulated and listed.

5.7.2. Study Conduct and Participant Disposition

An accounting of the study participants will be tabulated. The participant dose level cohort will be listed. The Full Analysis Set will be used.

Participant discontinuation from treatment and study will be tabulated and listed for each participant separately with their reason for discontinuation. The Safety Analysis Set will be used.

5.7.3. Study Treatment Exposure

The Safety Analysis Set will be used for the analysis of treatment exposure.

Listings and tables by dose level will be provided. Day 1 of a cycle is the first date of dose within that cycle.

090177e19dab896e\Approved\Approved On: 07-Jun-2023 12:23 (GMT)

Treatment exposure will be assessed based on Duration of Treatment (DOT) that is defined as the last active dose date minus the first active dose date + 1. DOT will be summarized, as a continuous variable, by dose level for Part 1 and overall across all dose levels and all study interventions; or by arm for Part 2. DOT may also be categorized into different intervals (≥1 cycle; ≥2 cycles; ≥3 cycles etc.) if deemed necessary, frequency and percentage of participants for each interval will be descriptively summarized by dose level for Part 1 and overall across all dose levels; or by arm for Part 2.

Dose modifications may occur in the following ways:

- Cycle delay – Day 1 of current cycle starts later than 28 (+2) days from Day 1 of the previous cycle (only applies to cycle 2 and above), will be used in determining cycle delay. For example, after cycle 1 ended for a patient in Part 1, a new cycle didn't start until 28 (+2) days after (but before 56 days after) cycle 1 day 1, the newly started cycle will be considered as cycle 2, and cycle 2 is considered delayed.

- Cycle skip – Day 1 of current cycle starts later than 56 (+4) days from Day 1 of the previous cycle (only applies to cycle 2 and above). For example, After cycle 1 ended for a patient in Part 2, a new cycle didn't start until 56 (+4) days after cycle 1 day 1, the newly started cycle will be considered as cycle 3, and cycle 2 is considered skipped for this patient.

Dose reduction – a decrease in the administered total daily dose (non-zero) compared to the planned total daily dose upon enrollment.
Dose Reduction is defined as the newly prescribed planned dose (by the investigator) at a visit being less than the immediate previous planned dose. Summaries of dose reductions will only focus on dose reductions which are due to adverse events. If the reason can't be identified based on the CRF data, AE would be assumed as the underlying reason. Accidental dose reduction or planned dose reductions (reductions as planned in the protocol, not due to AE) will not be summarized, and will only be listed. Such dose reductions will be reflected in the summary of overall relative dose intensity. Dose reduction is determined by comparing the current PLANNED dose (as prescribed by the investigator) and the previous PLANNED dose (as prescribed by the investigator). For example, if a patient is enrolled to and dosed at 100 mg at Cycle 1 Day 1 as planned, at the next scheduled dose visit (e.g., Cycle 1 Day 15 or Cycle 2 Day 1), the new planned dose is reduced to 50 mg by the investigator because of any adverse event the patient had experienced, this is an episode of dose reduction.

When the planned dose for a visit post Cycle 1 Day 1 has changed because of investigator's decision, study sites are instructed to capture the newly prescribed planned dose in the case report form. Dose reduction will be programmatically determined through this data.

Number of dose reductions may be summarized by dose level (treatment), if deemed necessary. Dose reduction will be listed and reasons for dose reduction (e.g., AE) if available, will be included in the listing.

-

090177e19dab896e\Approved\Approved On: 07-Jun-2023 12:23 (GMT)

Dose interruption – a skip in the planned administered daily dose upon enrollment.
The actual dose being zero (e.g., dose holding regardless of reason, missed doses or did not complete the planned dose due to adverse event) is considered as dose interruption. Dose interruption must due to adverse event. If the reason can't be identified based on the CRF data, AE would be assumed as the underlying reason.

If there is a planned dosing holiday in the treatment plan (e.g., 3 weeks on and 1 week off), an event of non-dosing days after the dosing holiday and before the next cycle start is considered as a dose interruption if it is driven by adverse event.

Dosing period is defined as either a day when a dose is scheduled to be administered to patient (e.g., injection, infusion) or a range of days when the investigational product is scheduled to be taken by patient (e.g., pill, tablets, capsules). The summation of all dosing periods will be the entire "treatment period" of a patient.

Number of dose interruptions may be summarized by dose level (treatment), if deemed necessary. Dose interruption will be listed, reasons for dose interruption (e.g., specific AE(s)) if available, will be included in the listing.

-

The following will be summarized by subject for overall and each dose level:

- Number of subjects per dose level;
- Median and range of number of cycles started per subject;
- Number (%) of subjects starting a cycle (1, 2, 3…);
- Number (%) of subjects with cycle delays and cycle skips;
- Number (%) of dose interruptions;
- Number (%) of subjects with dose reductions;
- Number (%) of each reason (drug related AE vs AE vs. Other) for cycle delays, dose interruptions and dose reductions;
- Time on treatment (median, range).

The following will be summarized by cycle received for overall and each dose level:

- Total number of cycles started;
- Number of cycles started per subject (median, range);

090177e19dab896e\Approved\Approved On: 07-Jun-2023 12:23 (GMT)

- Number of cycles before 1st delay (median, range);
- Number of cycles before 1st reduction (median, range);
- Number of cycles before 1st interruption (median, range).
- Number of cycles before 1st dose skip (median, range).

The following will be summarized for cumulative dose by dose level and cycle:

- Summary statistics (mean, median, standard deviation and range) of cumulative dose and percent of starting dose (compared to Day 1 dose of each cycle).

Listings by subject (ordered by dose level): cycle number, start date and stop date of each dosing period within each cycle (including records with 0 mg), administered total daily dose for each period, any missed doses with unknown dates (Y/N), number of missed doses with unknown dates, reason for any dosing changes, total planned dose, total actual dose received, percentage of planned dose, dose delay (yes/no), dose reduction (yes/no), and dose interruption (yes/no).

Listings by subject and each cycle (ordered by dose level): cycle length, administered total daily dose for each period, any missed doses with unknown dates (Y/N), number of missed doses with unknown dates, reason for any dosing changes, total planned dose, total actual dose received, percentage of planned dose, dose delay (yes/no), dose reduction (yes/no), and dose interruption (yes/no).

Intended (Planned) Dose Intensity -- Intended dose intensity is defined as the dose a patient is intended to receive (based on the initial dose a patient was assigned to at enrollment) for a cycle divided by the intended length of a cycle (e.g., week).

Ovberall Actual Dose Intensity -- Overall actual dose intensity is defined as the sum of actual doses a patient received during the entire actual treatment period divided by the intended treatment period in time unit (e.g., week).

Overall Relative Dose Intensity -- Exposure may be summarized per cycle and/or overall. For phase 1 oncology trials, in addition to treatment compliance calculation for the DLT observational period, the recommendation is that only overall exposure across the entire treatment period is summarized through Overall Relative Dose Intensity, i.e., Overall RDI, for each dose level of the investigational product (IP)

When the IP is is monotherapy, Overall RDI is calculated for the IP only. When the treatment is a combination of the IP with other agents, overall RDI should be calculated for the IP at a minimum. It is up to the study team whether overall RDI needs to be calculated for the combination agents.

Overall RDI (%) = [Overall Actual Dose Intensity] / [Intended Dose Intensity] * 100%.

090177e19dab896e\Approved\Approved On: 07-Jun-2023 12:23 (GMT)

Note in the RDI calculation, the Intended Dose and the Intended DI remain constant for all cycles. The intended dose level for a patient is fixed at the dose a patient was assigned to at enrollment, rather than the start of a cycle (this should not be confused with the new planned dose when there is a dose reduction or dose increase). The intended cycle length is the same for the entire treatment period, including the last cycle.

5.7.4. Concomitant Medications and Nondrug Treatments

Prior, concomitant, and further therapies (drug and non-drug treatments) will be coded by the World Health Organization (WHO) medical dictionary. Listings of prior, concomitant, and further therapies will be provided separately.

5.7.5. Patient Follow-up Summary for Time-to-Event

5.7.5.1. Patient follow-up summary for OS

Overall survival is the time from "start date" (i.e., first dose date or the randomization date) to date of death due to any cause. Patients last known to be alive are censored at date of last contact and the date of last contact will be derived for patients not known to have died at the analysis data cutoff date using the latest complete date (non-imputed) among the following:

- *All patient assessment dates (eg, blood draws [laboratory, PK], vital signs, performance status, electrocardiogram (ECG), tumor assessments, concomitant radiation, surgery)*
- *Start and end dates of follow-up anti-cancer therapies*
- *AE start and end dates*
- *Last date of contact where "Subject Remains in Follow-up" or "Subject No Longer Being Followed for Survival" collected on the "Survival Follow-up" eCRF*
- *Study drug start and end dates*
- [redacted]
- *Withdrawal of consent date*
- *Date of discontinuation on disposition eCRF pages (do not use if reason for discontinuation is lost to follow-up or death).*

The following 2 methods may be used to summarize the extent of follow-up for OS:

1. Reversing the censoring and event indicators and estimating follow-up time with the method of Kaplan and Meier (referred to as the "Kaplan-Meier potential follow-up or reversed Kaplan-Meier method")

090177e19dab896e\Approved\Approved On: 07-Jun-2023 12:23 (GMT)

2. Calculating the "observation time" ("start date" to date of death or last contact date) and presenting descriptively.

5.7.5.2. Patient follow-up summary for PFS

Patients who did not progress or die are censored at the date of the last adequate radiological tumor scan. The following 2 methods may be used to summarize the extent of follow-up for PFS:

1. Reversing the censoring and event indicators and estimating follow-up time with the method of Kaplan and Meier (referred to as the "Kaplan-Meier potential follow-up or reversed Kaplan-Meier method")

2. Calculating the "observation time" ("start date" to event date (progression or death) or the date of the last adequate radiological tumor scan for censored observations) and presenting descriptively.

5.7.6. Prior Therapy

Summary of prior therapy and regimens of patients will be presented. The data will be summarized by dose level and treatment categories. The therapies entered by the sites are coded to and categorized by ATC2 codes. Unless otherwise metioned, the therapies and regimens summarized should be in metastatic or locoregional setting only.

The following will be presented in the summary:

- Prior anti-cancer surgery
- Prior radiotherapy
- Prior adjuvant/neoadjuvant therapy
- Number of prior systemic line of therapy in any setting (1,2,3, >3, mean, median(range))
- Number of prior systemic line of therapy in advanced/metastatc setting (1,2,3, >3, mean, median(range))
- Number of prior lines containing chemotherapy in advanced/metastatic setting (1,2,3, >3, mean, median(range))
- Prior therapy in advanced/metastatic setting listed by relevant types of therapies and specific drug names.
  - Chemotherapies
    - ATC Level 3 Codes: L01A, L01B, L01C, L01D and ATC Level 4 codes: L01XA, L01XX, L01XY.
  - Immunotherapy/Biologics
    - ATC Level 3 Codes L01F and (ATC Level 4 Code L01XX and cmdecod='BMS 986301')
  - PARP inhibitors
    - ATC Level 4 Code L01XK

- Recommendations for breast cancer:
  - Number of prior lines containing endocrine therapy in advanced/metastatic setting (1,2,3, >3, mean, median(range))

090177e19dab896e\Approved\Approved On: 07-Jun-2023 12:23 (GMT)

090177e19dab896e\Approved\Approved On: 07-Jun-2023 12:23 (GMT)

- Prior therapies of interest
  - Any prior endocrine therapy (ATC Level 2 Code: L02):
    - Aromatase Inhibitors (ATC Level 4 Code: L02BG)
      - Letrozole (ATC4CD = "L02BG" and cmdecod= "LETROZOLE")
      - Exemestane (ATC4CD = "L02BG" and cmdecod= "EXEMESTANE")
      - Anastrozole (ATC4CD = "L02BG" and cmdecod= "ANASTROZOLE")
      - Others (ATC4CD = "L02BG" and cmdecod not in ("LETROZOLE", "EXEMESTANE", "ANASTROZOLE"),
    - SERD
      - Fulvestrant cmdecod = "FULVESTRANT"
      - Elacestrant cmdecod = "ELACESTRANT"
    - Others (ATC Level 2 Code L02 and not belong to AI or SERD
  - Any prior CDK46 inhibitor
    - ATC Level 4 Code: L01EF
  - Other targeted therapies
    - mTOR: ATC Level 4 Code: L01EG
    - PI3K: ATC Level 4 Code: L01EM

- Recommendations for prostate cancer
  - Prior therapies of interest
  - The number of prior systemic line of therapy, the number of prior lines containing endocrine therapy, and the number of prior lines containing chemotherapy in advanced and metastatic setting will be summarized (1,2,3, >3, mean, median(range)).

- Recommendations for lung cancer
  - The number of prior systemic line of therapy, the number of prior lines containing endocrine therapy, and the number of prior lines containing chemotherapy in advanced and metastatic setting will be summarized (1,2,3, >3, mean, median(range)).

  - The prior systemic medications in advanced and metastatic setting will be categorized to endocrine therapy, CDK46 inhibitor, chemotherapy, immunotherapy/biologics, and other targeted therapeies, frequency and percentage of patients in each category and at each medication within a category will be presented.

  - Prior systemic therapy, immunotherapy, targeted therapy, chemotherapy (look at Sasanlimab)

5.8. Data Monitoring Committee or Other Independent Oversight Committee

This is an open-label, non-randomized Phase 1 study. This study will not use a DMC. Discussions between the investigators and the sponsor regarding safety will occur in an ongoing manner at regular teleconferences and/or meetings to determine the safety profile and risk/benefit ratio and determine if further participant enrollment is appropriate. These individual and summary data would also include participants who are determined to be not applicable for DLT assessment.

6. INTERIM ANALYSES

No formal interim analysis will be conducted for this study. As this is an open-label study, the sponsor may conduct unblinded reviews of the data during the course of the study for the purpose of safety assessment, facilitating dose-escalation decisions, facilitating PK/PD modeling, and/or supporting clinical development.

7. REFERENCES

1. Neuenschwander, Beat, Alessandro Matano, Zhongwen Tang, Satrajit Roychoudhury, Simon Wandel, and Stuart Bailey. 2015. "A Bayesian Industry Approach to Phase I Combination Trials in Oncology." In *Statistical Methods in Drug Combination Studies*, by Wei Zhao and Harry Yang, 95 -- 135. Boca raton, Florida: Chapman & Hall/CRC Press.

2. Brookmeyer, R, and JJ Crowley. 1982. "A Confidence Interval for the Median Survival Time." *Biometrics* 38: 29 – 41.

090177e19dab896e\Approved\Approved On: 07-Jun-2023 12:23 (GMT)

8. APPENDICES

8.1. Appendix 1: Time to Event Data Analysis Censoring Rules

Table 2. Progression Free Survival Outcome and Event Dates

**Situation**	**Date of Event/Censoring**	**Outcome**
No adequate baseline assessment	'start date'[a]	Censored[a]
PD or death<br>- after at most one missing or inadequate post-baseline tumor assessment, or<br>- ≤ 17 weeks after 'start date'.	Date of PD or death	Event
PD or death<br>- after 2 or more missing or inadequate tumor assessments (> 17 weeks) .	Date of last adequate tumor assessment[b] documenting no PD prior to new anti-cancer therapy or missed tumor assessments	Censored
No PD		
New anti-cancer therapy given prior to PD or death		

[a] If the patient dies ≤*17 weeks* after 'start date' and did not initiate new anti-cancer therapy, the death is an event with date on death date.

[b] If there are no adequate post-baseline tumor assessments prior to the PD or death, then the time without adequate assessment should be measured from the 'start date'; if the criteria were met the censoring will be on the 'start date'.

Note: If no adequate tumor assessment within *17 weeks* after 'start date' then censor at 'start date'.

Table 3. Titme to Progression Outcome and Event Dates

**Situation**	**Date of Progression/Censoring**	**Outcome**
No adequate baseline assessment	'start date'	Censored
PD<br>- after at most one missing or inadequate post-baseline tumor assessment, or<br>- ≤ 17 weeks after 'start date'.	Date of PD	Event
PD<br>- after 2 or more missing or inadequate tumor assessments (> 17 weeks).[a]	Date of last adequate tumor assessment[a] documenting no PD prior to new anti-cancer therapy or missed tumor assessments.	Censored
No PD		
New anti-cancer therapy given prior to PD		
Death due to any cause		

[a] If there are no adequate post-baseline assessments prior to the PD, then the time without adequate assessment should be measured from the 'start date'; if the criteria were met the censoring will be on the 'start date'.

Note: If no adequate tumor assessment within *17 weeks* after 'start date' then censor at 'start date'.

090177e19dab896e\Approved\Approved On: 07-Jun-2023 12:23 (GMT)

8.2. Appendix 2: List of Abbreviations

The following is a list of abbreviations that may be used in the SAP.

Abbreviation	Term
1L	first line
2L	second line
3L	third line
ADME	absorption, distribution, metabolism, and excretion
AE%	percentage of unchanged drug excreted in urine
AIDS	acquired immunodeficiency syndrome
Alk Phos	alkaline phosphatase
ALT	alanine aminotransferase
AML	acute myeloid leukemia
ANC	absolute neutrophil count
anti-HBc	total hepatitis B core antibody
anti-HBs	hepatitis B surface antibody
ASCO	American Society of Clinical Oncology
AST	aspartate aminotransferase
AUC	area under the curve
[redacted]	[redacted]
AV	atrioventricular
BCRP	breast cancer resistance protein
BET	Bromodomain and Extra-Terminal motif
BID	twice daily
BLRM	Bayesian Logistic Regression Model
BOR	best overall response
BP	blood pressure
Bpm	beats per minute
BUN	blood urea nitrogen
BVN	bivariate normal
C1D1	Cycle 1 Day 1
C1D15	Cycle 1 Day 15
C2D1	Cycle 2 Day 1
CDK	cyclin-dependent kinases
cfDNA	cell-free DNA
CFR	Code of Federal Regulations
CHF	congestive heart failure
CI	confidence interval
CIOMS	Council for International Organizations of Medical Sciences
CK	creatine kinase

090177e19dab896e\Approved\Approved On: 07-Jun-2023 12:23 (GMT)

Abbreviation	Term
CL	clearance
CL/F	apparent total clearance
[redacted]	[redacted]
[redacted]	[redacted]
C_{max}	maximum observed concentration
[redacted]	[redacted]
[redacted]	[redacted]
[redacted]	[redacted]
CoA	coenzyme A
CONSORT	Consolidated Standards of Reporting Trials
COVID-19	coronavirus disease 2019
CR	complete response
CRC	colorectal cancer
CREBBP	cAMP response element-binding protein
CRF	case report form
CRISPR	clustered regularly interspaced short palindromic repeats
CRO	contract research organization
CRP	c-reactive protein
CRPC	castration-resistant prostate cancer
CSF	colony stimulating factor
CSR	clinical study report
CT	clinical trial; computed tomography
CTCAE	Common Terminology Criteria for Adverse Events
ctDNA	circulating tumor DNA
CV	coefficient of variation
CYP	Cytochrome P450
DDI	drug-drug interaction
DILI	drug-induced liver injury
DLT	dose-limiting toxicity
DMC	data monitoring committee
DNA	deoxyribonucleic acid
DOR	duration of response
DRE	disease-related event
EC	ethics committee
ECG	electrocardiogram
ECOG	Eastern Cooperative Oncology Group
eCRF	electronic case report form
EDP	exposure during pregnancy
EDTA	ethylenediaminetetraacetic acid
EMA	European Medicines Agency
EOT	end of treatment
[redacted]	[redacted]

090177e19dab896e\Approved\Approved On: 07-Jun-2023 12:23 (GMT)

Abbreviation	Term
ER	estrogen receptor
ESR1	estrogen receptor 1
EU	European Union
EudraCT	European Clinical Trials Database
EWOC	escalation with overdose control
[redacted]	[redacted]
FDA	Food and Drug Administration (United States)
FFPE	formalin-fixed paraffin-embedded
FIH	first-in-human
FSH	follicle-stimulating hormone
GALT	gut-associated lymphoid tissue
GCP	Good Clinical Practice
[redacted]	[redacted]
GFR	glomerular filtration rate
GGT	gamma-glutamyl transferase
GI	gastrointestinal
GLP	Good Laboratory Practice
[redacted]	[redacted]
HAT	histone acetyltransferases
HBcAb	hepatitis B core antibody
HbsAg	hepatitis B surface antigen
HBV	hepatitis B virus
HCV	hepatitis C virus
HCVAb	hepatitis C antibody
HDAC	Histone deacetylase
HER2	human epidermal growth factor receptor 2
HIPAA	Health Insurance Portability and Accountability Act
HIV	human immunodeficiency virus
HNSTD	highest non-severely toxic dose
HR	heart rate; hormone replacement; hormone receptor
HRT	hormone replacement therapy
IB	Investigator's Brochure
ICD	informed consent document
ICH	International Council for Harmonisation
IgM anti-HBc	immunoglobulin M antibody to hepatitis B core antigen
IL	interleukin
ILD	interstitial lung disease
IMP	investigational medicinal product
IND	Investigational New Drug
INR	international normalized ratio
IP manual	investigational product manual

090177e19dab896e\Approved\Approved On: 07-Jun-2023 12:23 (GMT)

Abbreviation	Term
IPAL	Investigational Product Accountability Log
IRB	Institutional Review Board
KAT	lysine acetyltransferase
LBBB	left bundle branch block
LDH	lactate dehydrogenase
LFT	liver function test
MAP	meta-analytic-predictive
MD	multiple dose
MEC	molar extinction coefficient
MedDRA	Medical Dictionary for Regulatory Activities
M/E (ratio)	myeloid to erythroid
mITT	modified intent to treat
MRI	magnetic resonance imaging
MTD	maximum tolerated dose
N/A	not applicable
NCCN	National Comprehensive Cancer Network
NCI	National Cancer Institute
NCOA2	nuclear receptor coactivator 2
NCOA3	nuclear receptor coactivator 3
NE	non-evaluable
NHT	novel hormonal therapies
NIMP	non-investigational medicinal product
NOAEL	no-observed-adverse-effect level
NSCLC	non-small cell lung cancer
OAT3	organic anion transporter 3
OR	objective response
ORR	overall response rate
OS	overall survival
PBPK	physiologically based pharmacokinetic
PCWG3	prostate cancer working group 3
PD	pharmacodynamics(s)
PD	progressive disease
PET	positron emission tomography
PFS	progression-free survival
PGx	pharmacogenomics
PI	principal investigator
PK	pharmacokinetic(s)
PO	per os (by mouth)
PR	partial response
PR	pulse rate
PS	performance status
PSA	prostate specific antigen

090177e19dab896e\Approved\Approved On: 07-Jun-2023 12:23 (GMT)

**Abbreviation**	**Term**
PT	prothrombin time
PTT	partial thromboplastin time
aPTT	activated partial thromboplastin time
PVC	premature ventricular contraction/complex
Q2D	every 2 days
QD	every day
QTc	corrected QT
QTcB	corrected QT (Bazett method)
QTcF	corrected QT (Fridericia method)
R_{ac}	accumulation ratio
RECIST	Response Evaluation Criteria in Solid Tumors
RNA	ribonucleic acid
RNAi	RNA interference
RP2D	recommended phase 2 dose
RR	response rate
SAE	serious adverse event
SAP	Statistical Analysis Plan
SARS-CoV-2	severe acute respiratory syndrome coronavirus 2
SD	stable disease
shRNA	short hairpin RNA
SoA	schedule of activities
SOC	standard of care
SOP	standard operating procedure
SRSD	single reference safety document
[redacted]	[redacted]
SUSAR	suspected unexpected serious adverse reaction
$t_{1/2}$	terminal elimination half-life
TBD	to be determined
TBili	total bilirubin
TBR	tumor background ratio
TGI	tumor growth inhibition
TID	three times daily
TNF	tumor necrosis factor
T_{max}	time to maximum concentration
Tmax,ss	steady-state T_{max}
TSC	tumor stasis concentration
TSH	thyroid-stimulating hormone
TTP	time to progression
ULN	upper limit of normal
US	United States
USPI	United States Package Insert

090177e19dab896e\Approved\Approved On: 07-Jun-2023 12:23 (GMT)

Abbreviation	Term
UVB	ultraviolet B
[redacted]	[redacted]
WBC	white blood cell
WOCBP	woman of childbearing potential

090177e19dab896e\Approved\Approved On: 07-Jun-2023 12:23 (GMT)
