# Supplementary material for: Inhibition of lysine acetyltransferase KAT6 in ER+HER2− metastatic breast cancer: a phase 1 trial
Source: Nat Med. 2024 Jun 1;30(8):2242–50. doi: 10.1038/s41591-024-03060-0 (PMC11333285; doi:10.1038/s41591-024-03060-0)
Supplement: Supplementary file 2 — Reporting Summary [file 41591_2024_3060_MOESM2_ESM.pdf]

nature portfolio

Corresponding author(s): Patricia M. LoRusso; Meng Li

Last updated by author(s): May 9, 2024

Reporting Summary

Nature Portfolio wishes to improve the reproducibility of the work that we publish. This form provides structure for consistency and transparency in reporting. For further information on Nature Portfolio policies, see our Editorial Policies and the Editorial Policy Checklist.

Statistics

For all statistical analyses, confirm that the following items are present in the figure legend, table legend, main text, or Methods section.

n/a	Confirmed	
☐	☒	The exact sample size (*n*) for each experimental group/condition, given as a discrete number and unit of measurement
☐	☒	A statement on whether measurements were taken from distinct samples or whether the same sample was measured repeatedly
☐	☒	The statistical test(s) used AND whether they are one- or two-sided<br>*Only common tests should be described solely by name; describe more complex techniques in the Methods section.*
☒	☐	A description of all covariates tested
☒	☐	A description of any assumptions or corrections, such as tests of normality and adjustment for multiple comparisons
☐	☒	A full description of the statistical parameters including central tendency (e.g. means) or other basic estimates (e.g. regression coefficient) AND variation (e.g. standard deviation) or associated estimates of uncertainty (e.g. confidence intervals)
☒	☐	For null hypothesis testing, the test statistic (e.g. *F*, *t*, *r*) with confidence intervals, effect sizes, degrees of freedom and *P* value noted<br>*Give P values as exact values whenever suitable.*
☒	☐	For Bayesian analysis, information on the choice of priors and Markov chain Monte Carlo settings
☒	☐	For hierarchical and complex designs, identification of the appropriate level for tests and full reporting of outcomes
☒	☐	Estimates of effect sizes (e.g. Cohen's *d*, Pearson's *r*), indicating how they were calculated

Our web collection on statistics for biologists contains articles on many of the points above.

Software and code

Policy information about availability of computer code

Data collection	No software was used for data collection.
Data analysis	SAS version 9.4 and R version 4.2.1 (2022-06-23)

For manuscripts utilizing custom algorithms or software that are central to the research but not yet described in published literature, software must be made available to editors and reviewers. We strongly encourage code deposition in a community repository (e.g. GitHub). See the Nature Portfolio guidelines for submitting code & software for further information.

Data

Policy information about availability of data

All manuscripts must include a data availability statement. This statement should provide the following information, where applicable:

- Accession codes, unique identifiers, or web links for publicly available datasets
- A description of any restrictions on data availability
- For clinical datasets or third party data, please ensure that the statement adheres to our policy

Upon request, and subject to review, Pfizer will provide the data that support the findings of this study. Subject to certain criteria, conditions, and exceptions, Pfizer may also provide access to the related individual de-identified participant data. See https://www.pfizer.com/science/clinical-trials/trial-data-and-results for more information.

Research involving human participants, their data, or biological material

Policy information about studies with human participants or human data. See also policy information about sex, gender (identity/presentation), and sexual orientation and race, ethnicity and racism.

Reporting on sex and gender	Information on sex was obtained for all study participants (as self-reported) and is detailed in Table 1. No information about gender was collected. No analysis was performed based on sex.
Reporting on race, ethnicity, or other socially relevant groupings	Information on race was obtained for all study participants and is detailed in Table 1. No analysis was performed based on race.
Population characteristics	No covariate analysis was conducted. The baseline characteristics of study participants are detailed in Table 1.
Recruitment	Patients were recruited by principal investigators as described here. Principal investigators selected patients based on their clinical judgment and their ability to ensure that patients could meet study specific requirements and inclusion/exclusion criteria as described in clinical study protocol. Investigators obtained written informed consent from each participating patient before any study-specific activity was performed. This study has 27 investigative centers (12 in the USA, 5 in Australia, 6 in the Republic of Korea, 4 in Japan). Enrollment started in November 2020 and the study is ongoing. Due to the geographical distribution of the study centers, participants may not represent the global general population. No other bias emerging from recruitment is expected.
Ethics oversight	This study was conducted in accordance with the Declaration of Helsinki and the Council for International Organizations of Medical Sciences International Ethical Guidelines. It followed all applicable guidelines, laws, and regulations. The protocol was approved by the ethics committee or the institutional review board. All patients provided written informed consent. Patient safety was monitored jointly by investigators and a safety assessment committee established by the sponsor.<br><br>The following independent ethics committee or the institutional review board provided approval of the study: Bellberry Human Research Ethics Committee, Eastwood, South Australia, Australia; Royal Melbourne Hospital Human Research Ethics Committee, Parkville, VIC, Australia; St John of God Health Care Human Research Ethics Committee, Perth, Western Australia, Australia; National Cancer Center IRBc, Chuo-ku, Tokyo, Japan; Kanagawa Cancer Center IRB, Yokohama, Kanagawa, Japan; Aichi Cancer Center Hospital Institutional Review Board, Nagoya, Aichi, Japan; Seoul National University Bundang Hospital Institutional Review Board, Seongnam, Gyeonggi-do, Republic of Korea; IRB of Samsung Medical Center, Seoul, Seoul-Teukbyeolsi [Seoul], Republic of Korea; Seoul National University Hospital IRB/IEC, Seoul, Seoul-Teukbyeolsi [Seoul], Republic of Korea; Asan Medical Center Institutional Review Board, Seoul, Republic of Korea; Severance Hospital, Yonsei University Health System IRB, Seoul, Seoul-Teukbyeolsi [Seoul], Republic of Korea; IRB of Kyungpook National University Chilgok Hospital, Daegu, Taegu-Kwangyŏkshi, Republic of Korea; Western Institutional Review Board (WIRB), Puyallup, Washington, United States; Salus IRB, Austin, Texas, United States; Advarra, Inc, Columbia, Maryland, United States; UCSF Human Research Protection Program, San Francisco, California, United States; U.T. M. D. Anderson Cancer Center Institutional Review Board, Houston, Texas, United States; University of Louisville IRB #1 – Biomedical, Louisville, Kentucky, United States.

Note that full information on the approval of the study protocol must also be provided in the manuscript.

Field-specific reporting

Please select the one below that is the best fit for your research. If you are not sure, read the appropriate sections before making your selection.

☒ Life sciences ☐ Behavioural & social sciences ☐ Ecological, evolutionary & environmental sciences

For a reference copy of the document with all sections, see nature.com/documents/nr-reporting-summary-flat.pdf

Life sciences study design

All studies must disclose on these points even when the disclosure is negative.

Sample size	The sample size for part 1A was planned to be approximately 25-30 patients (and approximately 6-9 for part 1B) - it was based on the number of doses planned to be tested and assuming at least 3 patients per dose would be evaluable DLTs assessment. For Part 2A and 2B, 30 patients were planned to be enrolled in each cohort. it was deemed sufficient given that, even though ORR was a secondary endpoint, observing at least 4 responders out of 30 participants in part 2a would translate into a 0.746 posterior probability for the true response rate to be higher than 10%. Similarly for part 2B, observing at least 6 responders out of 30 participants would translate into a 0.786 posterior probability for the true response rate to be higher than 15%.
Data exclusions	No data excluded for the cohorts reported (part 1A mono dose escalation, part 2A mono expansion at RDE, fulvestrant combo at RDE (part 1B and part 2B at 5 mg QD).
Replication	This was a non-randomized phase 1 study reporting safety, pharmacokinetics, pharmacodynamics, and efficacy data. Replication of the results will be reported with follow-up studies.
Randomization	This was a non-randomized phase 1 study.

Blinding	This was an open-label study. Blinding is not applicable.

Reporting for specific materials, systems and methods

We require information from authors about some types of materials, experimental systems and methods used in many studies. Here, indicate whether each material, system or method listed is relevant to your study. If you are not sure if a list item applies to your research, read the appropriate section before selecting a response.

Materials & experimental systems

n/a	Involved in the study
☐	☒ Antibodies
☒	☐ Eukaryotic cell lines
☒	☐ Palaeontology and archaeology
☒	☐ Animals and other organisms
☐	☒ Clinical data
☒	☐ Dual use research of concern
☒	☐ Plants

Methods

n/a	Involved in the study
☒	☐ ChIP-seq
☒	☐ Flow cytometry
☒	☐ MRI-based neuroimaging

Antibodies

Antibodies used	Rabbit Anti-Acetyl-Histone H3(Lys23): Catalog No. 07-355, Lot No. 3468685, from Millipore Sigma; and Mouse Anti Histone H3 (N terminus): Catalog No. 39763, Lot No. 20418023, from Active Motif; were used for the PBMC H3K23Ac PD assay. Acetyl-Histone H3 (Lys23) (D6Y7M) Rabbit mAb: Catalog No. 14932 from Cell Signaling Technology was used for the tumor H3K23Ac IHC assay.
Validation	The H3K23Ac MSD and IHC assays used in the manuscript were customary established based on the antibody validation notes provided by the suppliers. Rabbit Anti-Acetyl-Histone H3(Lys23) Catalog No. 07-355, from Millipore Sigma validated in ChIP, Western blot; Mouse Anti Histone H3 (N terminus): Catalog No. 39763, from Active Motif validated in CHIP-Seq, CHIP and ICC/IF; Acetyl-Histone H3 (Lys23) (D6Y7M) Rabbit mAb: Catalog No. 14932 from Cell Signaling Technology validated in Western blot.

Clinical data

Policy information about clinical studies

All manuscripts should comply with the ICMJE guidelines for publication of clinical research and a completed CONSORT checklist must be included with all submissions.

Clinical trial registration	NCT04606446
Study protocol	Information can be accessed at: https://www.clinicaltrials.gov/study/NCT04606446 or by reviewing the study protocol in the Supporting Information.
Data collection	Enrollment of participating patients started in November 2020 and is ongoing; the data cutoff was September 30, 2023. Data were collected by clinical investigative sites as described in study specific clinical protocol. This study has 27 investigative centers (12 in the USA, 5 in Australia, 6 in the Republic of Korea, 4 in Japan).
Outcomes	The primary objective was to assess safety per Common Terminology Criteria for Adverse Events (CTCAE) 5.0 and tolerability for both dose escalation and expansion parts. PK profile of PF-07248144 was a secondary objective for both parts, while antitumor activity per Response Evaluation Criteria in Solid Tumors (RECIST) 1.1 was an exploratory objective for the dose escalation part and a secondary objective for the dose expansion parts. Other exploratory objectives included pharmacodynamics (PD) and predictive biomarkers for both parts.<br>Assessments for safety included dose-limiting toxicity (DLTs; Cycle 1), treatment-emergent and treatment-related adverse events (TEAEs and TRAEs), as well as laboratory abnormalities. Concentrations used to generate PK parameters were quantified using validated bioanalytical methods. Antitumor activity was assessed by investigator based on RECIST v1.1 including best overall response (BOR), objective response rate (defined as the proportion of patients with a BOR of complete response [CR] or partial response [PR]), duration of response, disease control rate (including CR, PR, stable disease [SD], and non-CR/non-progressive disease), clinical benefit rate (defined as the proportion of patients with a BOR of CR, PR, or SD lasting for at least 24 weeks), and progression-free survival.<br>H3K23Ac PD biomarker of KAT6 modulation in pre- and on-treatment peripheral blood mononuclear cell samples and tumor biopsies was evaluated by Meso Scale Discovery and immunohistochemistry assays, respectively. Circulating tumor DNA (ctDNA) and gene mutations were evaluated by Guardant360® assay (74 gene-panel based, Guardant Health Inc. Redwood City, CA). Variant allele frequencies (VAFs) were defined as percentages of the variant mutant reads over the total number of reads from cfDNA in a sample. Percentage changes in ctDNA from baseline were analyzed only for patients with detected mutations (defined as having any missense, nonsense, or frameshift mutations or splice site alterations either at baseline and/or on-treatment). Mean VAF and percent change in mean VAF values were provided by Guardant Health Inc.

Plants

Seed stocks	Not applicable.
Novel plant genotypes	Not applicable.
Authentication	Not applicable.
